# Reconstruction of Bacterial and Viral Genomes from Multiple Metagenomes

**DOI:** 10.3389/fmicb.2016.00469

**Published:** 2016-04-12

**Authors:** Ankit Gupta, Sanjiv Kumar, Vishnu P. K. Prasoodanan, K. Harish, Ashok K. Sharma, Vineet K. Sharma

**Affiliations:** ^1^Metagenomics and Systems Biology Group, Department of Biological Sciences, Indian Institute of Science Education and ResearchBhopal, India; ^2^Department of Medicine, University of Connecticut Health CenterFarmington, CT, USA

**Keywords:** binning, genome assembly, metagenome, spiking, bacterial draft genomes, viral draft genomes

## Abstract

Several metagenomic projects have been accomplished or are in progress. However, in most cases, it is not feasible to generate complete genomic assemblies of species from the metagenomic sequencing of a complex environment. Only a few studies have reported the reconstruction of bacterial genomes from complex metagenomes. In this work, Binning-Assembly approach has been proposed and demonstrated for the reconstruction of bacterial and viral genomes from 72 human gut metagenomic datasets. A total 1156 bacterial genomes belonging to 219 bacterial families and, 279 viral genomes belonging to 84 viral families could be identified. More than 80% complete draft genome sequences could be reconstructed for a total of 126 bacterial and 11 viral genomes. Selected draft assembled genomes could be validated with 99.8% accuracy using their ORFs. The study provides useful information on the assembly expected for a species given its number of reads and abundance. This approach along with spiking was also demonstrated to be useful in improving the draft assembly of a bacterial genome. The Binning-Assembly approach can be successfully used to reconstruct bacterial and viral genomes from multiple metagenomic datasets obtained from similar environments.

## Introduction

The complete genome sequences of bacteria abundant in different environmental systems are essential to uncover the genetic diversity present on this planet. However, the fact that 98% bacteria cannot be cultured using traditional methodologies is a limiting factor for their genomic sequencing using the available sequencing technologies. In this scenario, metagenomics has emerged as a culture independent methodology to directly sequence the microbial genomes from their environments. The main objective of the metagenomic projects is to access the genetic information of the inherent microbes irrespective of the fact that whether the individual complete genomic sequences are achievable or not. In most cases, it is not feasible to generate complete genomic assemblies of species from the metagenomic sequencing of a sample obtained from a complex environment. It is mainly due to the inherent enormous microbial diversity which requires massive sequencing and involves substantial cost to obtain a reasonable coverage for each species.

In the last decade, several large and small-scale metagenomic projects have been accomplished and a large number of projects are currently under progress. Due to the unprecedented improvements in next generation sequencing (NGS) technologies, the amount of data generated from the recent metagenomic projects has shown a logarithmic upward trend compared to the initial metagenomic projects. Resultantly, sequence data from multiple samples sequenced from the same environment or from similar environments has been gradually accumulating. In this scenario, the reconstruction of bacterial genomes from a mixture of metagenomic reads obtained from multiple samples of similar origin appears feasible. At present, only a few studies have reported the reconstruction of genomes from complex metagenomic samples (Luo et al., 2011; Iverson et al., 2012; Sharon and Banfield, 2013; Nielsen et al., 2014) and from using a mixture of multiple metagenomes (Albertsen et al., 2013). The availability of only a few such reports point toward the technical difficulties and limitations of the existing approaches.

The sequence data obtained from a metagenomic project contains a mixture of short reads derived from the microbial species present in that environment, but lacks the information on their taxonomic origin. Therefore, as a first step the taxonomic binning of metagenomic reads into their respective genomic bins is aimed. The factors influencing the precise taxonomic binning includes the read length (Patil et al., 2011), community complexity in terms of distinct phylotypes of similar origin (Dröge and McHardy, 2012), availability of reference data from taxa closely related to the origin of sequences (Qin et al., 2010; Dröge and McHardy, 2012) and, requirement of large computational resources (Sharma et al., 2012).

The taxonomic binning is commonly carried out using a homology-based or a composition-based approach, or a combination of these two approaches (Sharma et al., 2012). Among the two approaches, the homology-based methods, such as MEGAN (Huson et al., 2007) and MetaBin (Sharma et al., 2012), are more sensitive and accurate, but suffer primarily due to the time required to generate the BLAST or BLAT alignments. The composition-based or marker-gene based approaches, such as Phylopythia (McHardy et al., 2007), TACOA (Diaz et al., 2009), PhymmBL (Brady and Salzberg, 2009), and MetaPhlAn (Segata et al., 2012) are several times faster in execution but provide limited classification accuracy and efficiency. However, given the magnitude of the metagenomic sequence data, it is impractical to implement a homology-based approach. A recent program Kraken uses exact alignment of k-mers for binning and offers genus-level sensitivity and precision similar to that of BLAST (Wood and Salzberg, 2014) and is 909 times faster compared to Megablast. Therefore, Kraken appears to be a method of choice for carrying out the taxonomic binning of large datasets given its speed, precision, and accuracy.

After carrying out the taxonomic binning, the next step is the reconstruction of genomes for which the currently used methods either perform the alignment of reads against the available reference genomes or carry out *de-novo* assembly. Commonly used alignment based methods such as BOWTIE (Langmead et al., 2009), BWA (Li and Durbin, 2009), MAQ (Li et al., 2008), SOAP/SOAP2 (Li R. et al., 2009) etc., are limited by the required computational time, and unavailability of reference genome sequences. In addition to the above methods, there are several *de novo* assemblers available for metagenomic data such as, Genovo (Laserson et al., 2011), MEGAHIT (Li et al., 2015), MetaVelvet (Namiki et al., 2012), SOAPdenovo (Luo et al., 2012), IDBA-UD (Peng et al., 2012), META-IDBA (Peng et al., 2011), Bambus 2 (Koren et al., 2011). Among these, MEGAHIT and SOAPdenovo2 are *de novo* assemblers for assembling large metagenomic data using *de Bruijn* graph, whereas, Genovo assembler uses a generative probabilistic model of read generation algorithm and can deal with high levels of taxonomic heterogeneity and can construct sequences even for the low coverage species. Thus, the recent developments in computational methods allow greater accuracy in binning of metagenomic reads and offer improved assembly. In addition to these methods, recently, a few tools have also been developed, such as CONCOCT (Alneberg et al., 2014), GroopM (Imelfort et al., 2014), MaxBIN (Wu et al., 2014), MetaBAT (Kang et al., 2015), which aim to reconstruct the genomes from metagenomes, however, majority of these tools struggles in coping up with large metagenomic datasets and provide limited flexibility in parameters selection and have their own limitations.

Deep sequencing data has been used to partially assemble multiple genomes from rumen metagenome with varying levels of completeness (Hess et al., 2011). Reassembly of a genome of single genotype (or species) has been reported to be possible provided ample coverage (20 ×) is achieved from a complex metagenome (Luo et al., 2011). At lower coverage, errors during assembly may occur due to chimeric sequences which form because of the presence of closely related species. Attempts have also been made with partial success to assemble individual genomes from a complex metagenomic data set by spiking the metagenomic data with the target species (Luo et al., 2011).

Reconstruction of genomes with varying level of completeness has been shown for 49 genomes by Wrighton et al. for at least five phyla for which there were no previous genomic information available (Wrighton et al., 2012). Construction of bacterial genomes constituting about 1% of the community has been attempted from various metagenomes including seawater (Iverson et al., 2012), human gut (Di Rienzi et al., 2013), and sediments (Castelle et al., 2013). Recently, a sequence composition-independent method has been proposed based on the differences in the relative population abundance of similar community as a primary binning approach followed by post binning refinement (using composition-dependent binning) of bins representing potential genomes in a metagenome (Albertsen et al., 2013). The authors used this approach to obtain 31 genome bins of rare bacterial species, of which 12 were refined into complete (or near complete) genome sequences for the candidate phylum TM7 from activated sludge bioreactor (Albertsen et al., 2013). The method relies on the presumption that the abundance of each gene from a bacterial chromosome will be similar in abundance to any other gene from that chromosome. Though the method could segregate abundant species in a metagenome, ample segregation could not be achieved for the taxonomically related species in a complex metagenome.

Nielsen et al. used strategy based on binning co-abundant genes across various metagenomic samples from human gut without requiring reference genome sequences (Nielsen et al., 2014). Using this approach 238 bacterial species were assembled, of which the sequences of 181 were not known earlier. Additionally, they were able to assign a taxonomic group to 10% of the total genes from 396 samples at species level and 161 “reference species gene sets” were created using these genes. However, it was observed that the abundance profiles of these gene sets were highly inconsistent. For 88 of these “reference species gene sets,” >25% of the genes had Pearson Correlation Coefficient < 0.5. The genes within 56 of these gene sets belonged to multiple distinct metagenomic species (MGS) and for 12 of these gene sets no internal correlation was found. The reasons for this inconsistency is not known and appears to be a limitation since the two approaches, taxonomic assignment and co-abundance profiles, are not in consensus.

The objective of the present study is to propose and evaluate the combination of binning and assembly “Binning-Assembly” (BA) approach to construct individual bacterial genomes from metagenomic reads. Since, in a single metagenome, the genomic coverage expected for a single species is generally insufficient to construct a reasonable draft, the taxonomically assigned reads from multiple metagenomes have been used cumulatively. Therefore, in this work, a two-step methodology is followed in which the reads are first assigned into taxonomy bins, followed by their assembly based on reference genomes to reconstruct draft genome sequences of related bacterial species present in human gut.

## Materials and methods

### Metagenomic data, binning, and reference assembly

High quality human gut metagenomic data for 124 individuals generated using Illumina sequencing technology was retrieved from BGI website (ftp://public.genomics.org.cn/BGI/gutmeta/High_quality_reads/) (Qin et al., 2010). Out of 124 individuals, 70 healthy individuals for which the paired-end sequence data with read length of 75 bp was available were considered for further analysis. The paired-end reads were assembled into a single read using FLASH v 1.2.10 (Magoč and Salzberg, 2011) with an overlap size of 4 bp and the other parameters were set to default (Since, it was known that the two sequences are mate pairs, hence, the minimum criteria of 4 bp was used). The 75 bp paired-end reads were assembled using FLASH to generate a single read (average length 131.2 bp). Only those samples for which >55% of the paired-end reads could be assembled in a single read were selected (Table S1). Therefore, 72 datasets from 67 individuals (for five individuals, there were two datasets) were used in the present study. These paired-end assembled reads are referred to as “reads” in the manuscript text. The taxonomic binning of reads was performed using Kraken (Wood and Salzberg, 2014). The reads assigned to each genus were aligned with all the available reference bacterial genomes (NCBI, ftp://ftp.ncbi.nlm.nih.gov/genomes/) of that genus using Burrows-Wheeler Aligner (BWA) using default parameters (Li and Durbin, 2009). An assembly was considered to be good, or referred to as “reasonable assembly,” if it covers 85% of the reference genome. Similarly, the reads classified into virus domain were pooled together and aligned against all available reference viral genome sequences (NCBI, ftp://ftp.ncbi.nlm.nih.gov/genomes/Viruses/) using “BWA-MEM” and SAMtools (version 0.1.19) (Li H. et al., 2009) with default parameters. Metagenemark (Zhu et al., 2010) was used to predict ORFs in the selected assembled draft genomes.

### *De-novo* metagenomic assembly and alignment of contigs

Assembly of reads was performed using Genovo with 50 iterations (Laserson et al., 2011). The general assembly statistics of contigs were calculated using assemblathon_stats.pl. The percentage of assembly was calculated by aligning contigs to the genomic sequences of the selected genomes with default parameters using “BWA-MEM” (version 0.5.9) and SAMtools (version 0.1.19).

### Construction of spiked metagenome

The next-generation sequencing reads were simulated using ART Software (Huang et al., 2012) to attain a 200 × genomic coverage of a genome and were further used to spike the metagenomic data for that genome followed by assembly using Genovo with 50 iterations and SOAPdenovo2 at k-mer length of 63.

### Comparative genomics of the assembled genomes

BLAST Ring Image Generator (BRIG) (Alikhan et al., 2011) was used to construct the circular genome map and compare the assembled genome with the available complete reference genome using BLAST with default parameters and *e* < 10^−5^.

### Standard and high quality draft assemblies

In the current study, a draft genome with 80% genomic assembly is termed as standard draft and a draft genome with 90% genomic assembly is termed as high quality draft as defined by Chain et al. (2009). The term “percentage assembly” used in the manuscript refers to the completeness of the genomes reconstructed using different approaches. The term “coverage” used in the manuscript symbolize sequencing depth, which represents average number of times a given region has been sequenced by independent reads.

## Results

**Figure 1:**
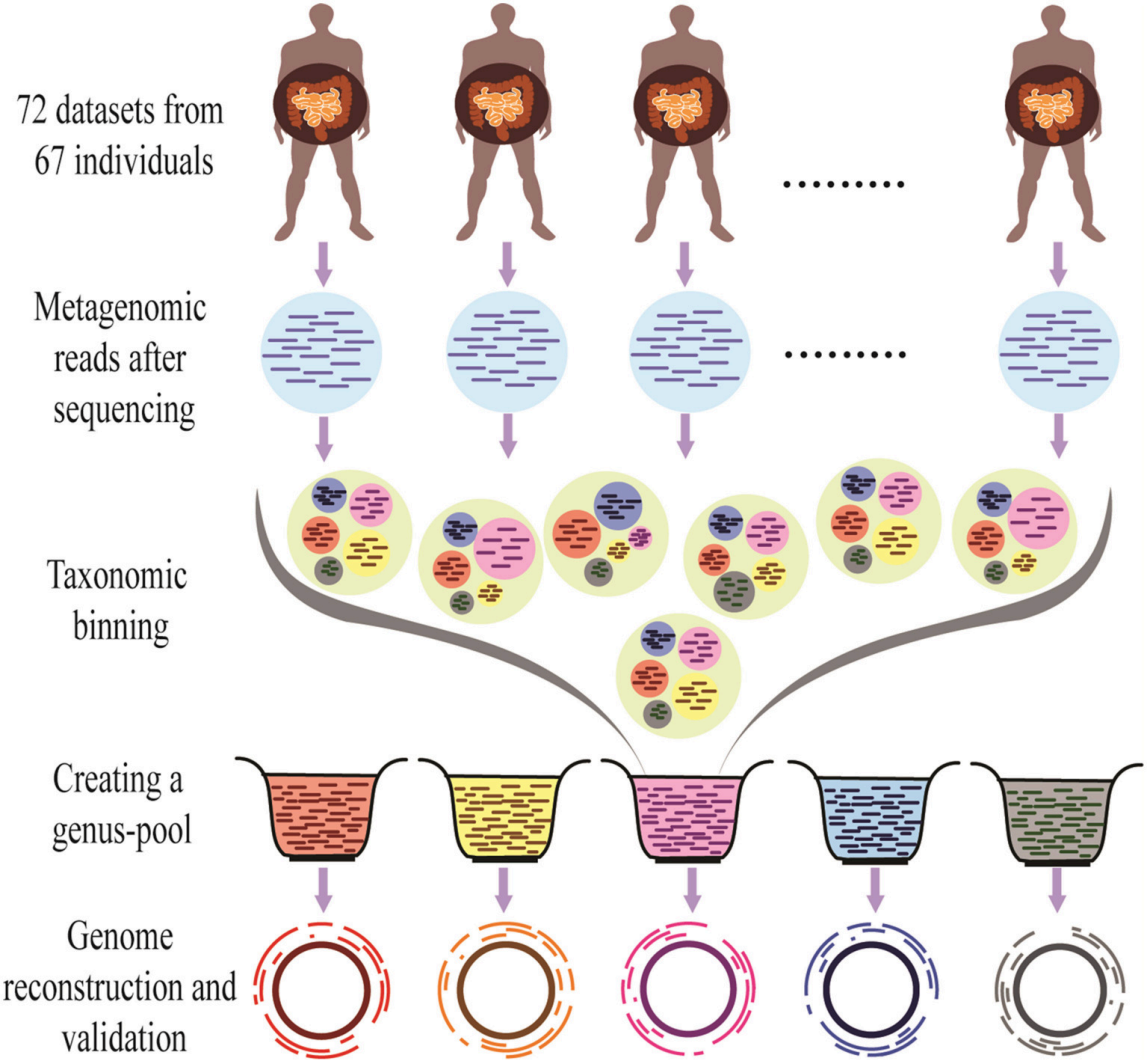
**Outline of the Binning-Assembly approach used in this study**. Metagenomic reads from similar metagenomes were used for taxonomic binning to make genus-pool, which is further used for the reconstruction and validation of the genomes.

The overall methodology of the present work is shown in Figure 1.

### Taxonomic binning

For the reconstruction of genome sequences, the first task in this approach is the assignment of reads into different taxonomic bins to estimate the diversity and abundance of various species present in the metagenome. The taxonomic assignment of the reads was performed using Kraken (using k-mer 24), since it is a faster and accurate classifier for small read lengths (>100 bp) compared to the available binning tools. Kraken could classify an average of 32.56% of the total number of reads from each metagenome at different levels of taxonomy (Figure S1). These results concurs with its previously demonstrated classification efficiency on Human Microbiome Project data and the observed low classification efficiency is due to the significant differences between the unclassified reads and the genomic sequences present in the training database (Wood and Salzberg, 2014).

A total of 219 bacterial families could be identified from the 72 metagenomic datasets (Tables S2, S3) and all of these were also found to be present in all individual metagenomic datasets. It is interesting to note that out of the total number of reads assigned at the family level, >80% of the reads belonged to only eight families (Figure S2). With respect to the total percentage of reads mapped to each family across all 72 datasets, the five most abundant bacterial families were *Bacteroidaceae* (31.37%), *Ruminococcaceae* (12.19%), *Eubacteriaceae* (11.65%), *Lachnospiraceae* (10.05%), *Porphyromonadaceae* (5.71%) (Table S2). A total of 606 genera were identified from 219 families. Similar results have also been reported by other studies (Canny and McCormick, 2008; Qin et al., 2010; Stearns et al., 2011; Maier et al., 2015; Newton et al., 2015).

Though viruses are an important component of human gut flora, their abundance and distribution in human gut has been comparatively less explored (Mokili et al., 2012). Even in the study by Qin et al. from which the data was retrieved for this work, the sequencing or identification of viral metagenome was not an objective of the study. However, while the sequencing of human gut bacterial metagenome was performed, the bacteria-associated viruses (bacteriophages, 215 out of 279) and human associated viruses (64 out of 279) were also sequenced and their reads were found in the selected datasets. Therefore, a total of 50 viral families (containing at least 100 reads) could be identified from the selected metagenomes, out of which 12 families were present across all 72 datasets and eight viral families were present in less than five datasets. The reads belonging to viral domain were combined together from all metagenomes to create a pool of virus reads. Among all viral families, *Siphoviridae* (34.77%), *Poxviridae* (12.03%), *Myoviridae* (11.57%), *Herpesviridae* (7.49%), *Baculoviridae* (6.97%) were found as the five most abundant families (Table S4). *Siphoviridae* and *Myoviridae* were also found abundant in human gut in previous studies (Minot et al., 2011; Nielsen et al., 2014; Waller et al., 2014).

### Alignment based reconstruction of genomes

After the taxonomic classification of reads, the different strategies for the reconstruction of bacterial genomic sequences were examined. If the reconstruction of a bacterial genome is attempted from a single metagenome, in most cases a reasonable draft assembly may not be achieved due to the lack of sufficient number of reads (coverage) of that genome. Therefore, an apparently better strategy would be to combine multiple metagenomes having similar origin to increase the representation of reads of different species present in the metagenomes. However, the resultant mixture of reads is likely to increase the data size tremendously to be handled by computationally intensive currently used assemblers, genomic complexity, and time required for the assembly algorithms. Therefore, in the present study, instead of combining all the reads from all metagenomes, the reads belonging to only a single genus from 72 metagenomes were pooled together to create a pool of reads specific for each genus referred to as “genus-pool” in the manuscript. The genus-pool for each individual genus is likely to facilitate the assembly of the genomes belonging only to that genus, thereby, reducing the chances of errors which may result by inclusion of reads from other genus, furthermore, reducing the data size and decreasing the time required for the assembly process. It should be noted that assembly using a genus pool of reads belonging to closely related species might also result in chimeric assembly, however, it can be countered by performing additional steps of verifying the completeness of the ORFs to validate the accuracy of the reconstructed genomes.

The genus-pool of each individual genus was aligned using BWA-MEM with the complete bacterial genomes known in the respective genus (Table S5). A total of 1156 bacterial genomes could be identified with ≥1% assembly. More than 80% (standard draft) and 90% (high-quality draft) complete assemblies could be reconstructed for a total of 126 and 90 bacterial genomes, respectively. The percentage assembly of the most represented genomes found in the eight most abundant genera is shown in Table 1 and were used for further analysis in the manuscript as “selected genomes.” The maximum (99.17%) assembly was achieved for *Odoribacter splanchnicus* DSM 20712 genome belonging to the genus *Odoribacter* (Table 1). It is to be noted that the reconstructed bacterial chromosomal sequences were closely (92–97% identity) related to the reference genome and could possibly represent the different strains of the same species or a closely related species. The validation of these reconstructed genomes was carried out by verifying the completeness of their ORFs and 99.8% ORFs (including ORF's with single N's) were found to be completely present with 99.8% identity (Table 1 and Table S6). The sequence of these selected genomes are made publicly available and can be accessed from (http://metagenomics.iiserb.ac.in/m_data/GFM/genomes.php).

**Table 1:** **Assembly and validation of eight selected genomes**.

**Family**	**Genus**	**Reference Genome**	**% Assembly**	**% Identity**	**% Complete ORFs**
*Porphyromonadaceae*	*Odoribacter*	*Odoribacter splanchnicus* DSM 20712	99.17	97	99.94
*Bacteroidaceae*	*Bacteroides*	*Bacteroides thetaiotaomicron* VPI 5482	98.89	92	99.93
*Akkermansiaceae*	*Akkermansia*	*Akkermansia muciniphila* ATCC BAA 835	98.71	93	99.95
*Porphyromonadaceae*	*Parabacteroides*	*Parabacteroides distasonis* ATCC 8503	98.69	97	99.81
*Lachnospiraceae*	*Roseburia*	*Roseburia hominis* A2 183	97.79	94	99.56
*Bifidobacteriaceae*	*Bifidobacterium*	*Bifidobacterium longum* JCM 1217	97.14	94	99.80
*Enterobacteriaceae*	*Escherichia*	*Escherichia coli* K 12 substr MDS42	95.72	95	99.84
*Eubacteriaceae*	*Eubacterium*	*Eubacterium siraeum* V10Sc8a	95.07	93	99.84

This suggests that standard draft genomes and high quality draft genomes (as defined by Chain et al., 2009) of the related species or strains of the same species, which was used as reference, can be reconstructed using the present BA approach. It is significant since the genetic variation present within the same microbial species in different metagenomic samples obtained from the same or similar environments, such as human gut in this study, is low. Thus, the genomes of these variants of the same species can be easily reconstructed, without de-novo sequence assembly (which is not only computationally intensive but also the currently available assemblers fail on such huge amount of data), if the reference strain is available.

### Number of reads required to achieve reasonable draft assembly

In the above section, the genus-pool from 72 metagenomes was used to generate 1156 bacterial genomic assemblies. However, it would be interesting to estimate the minimum number of reads which can yield the similar percentage of assembly, as achieved in the above section, for a particular genome. A metagenome harbors several species with different relative abundances which may vary from sample to sample. Therefore, the number of reads in each metagenome which are required for achieving reasonable (≥85%) assembly for a particular species is likely to vary depending upon the abundance of that species in the metagenomes. Therefore, in the scenario where multiple metagenomes of similar origin are available, the following two strategies were evaluated for the reconstruction of a genome.

#### Strategy-I: reconstruction of an abundant species from metagenomes

To evaluate the first strategy, incremental assembly was performed for the eight selected bacterial genomes (Figure S3 and Table S7). The reconstruction of a genome was first attempted by using the reads from a metagenome having the maximum abundance of that genus followed by addition of metagenomes in decreasing order of abundance of that genus. In the case of *Akkermansia muciniphila* ATCC BAA 835, belonging to family *Akkermansiaceae*, the first metagenome (MH0060_081222) selected for the assembly was the one which contained the maximum number of reads for the genus *Akkermansia*, and subsequently the next metagenome (MH0054_081222) which contained the next highest number of reads of that genus was added, and the process was continued for all 72 metagenomes. An assembly of 91.02% was achieved by just using the reads from the first metagenome which had 164,835 reads of *Akkermansia* genus. The assembly increased gradually and reached near saturation (98.03%) with the addition of 789,932 reads from eight metagenomes, and a maximum assembly of 98.71% was achieved on addition of reads from all 72 datasets. It is to be noted that on using the first metagenome, almost 99.67% of the reads (164,296 out of 164,835) corresponding to only 8.11 × coverage of the genome was sufficient to achieve 91% assembly. This shows that a reasonably good assembly can be achieved for an abundant species from a single metagenome where a higher representation of reads only from that species is expected, as compared to pooling reads from multiple metagenomes where the reads might belong to closely related species. Similar trend was observed for *O. splanchnicus* DSM 20712, *Bacteroides thetaiotaomicron* VPI 5482, *Parabacteroides distasonis* ATCC 8503, *Eubacterium siraeum* V10Sc8a, and *Escherichia coli* K 12 MDS42.

However, in the case of *Bifidobacterium longum* JCM 1217 (family *Bifidobacteriaceae*), only 43.22% assembly was achieved from addition of 90,369 reads from the first metagenome which increased to 83.99% on addition of the second metagenome, which contained the next higher number of reads, and reached near saturation (94.72%) after addition of 606,716 reads from 13 metagenomes. In this case, on addition of first metagenome, only 26.14% reads were used for the assembly (23,630 out of 90,369) corresponding to a low coverage of 4.9 ×, and as a result only 43.22% assembly was achieved. Similar pattern was observed in case of *Roseburia hominis* A2 183. In these cases, the observed low percentage assembly achieved from the first metagenomes, which contained largest number of reads for the genus to which the genome belonged, could be due to the low number of species specific reads in these metagenomes.

#### Strategy II: coverage required for reconstruction of a species

**Figure 2:**
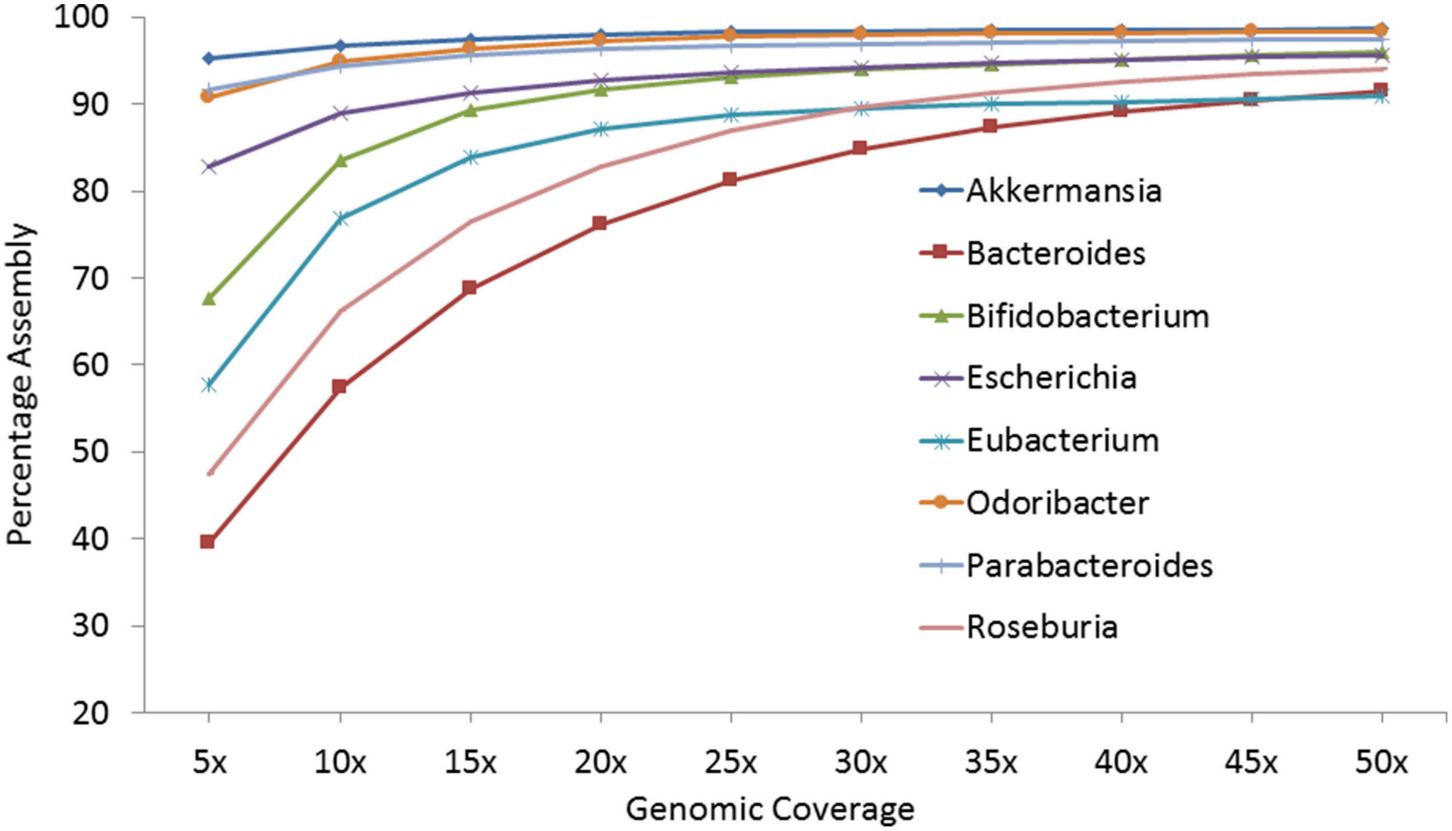
**Percentage of assembly achieved for eight selected genomes at different genomic coverage**.

The previous strategy demonstrated the assembly of an abundant species from a single metagenome or on pooling multiple metagenomes sequentially. However, in this strategy, the average number of reads required to attain a reasonable (>85%) assembly, independent of the genus abundance, is estimated. For the selected top eight genomes, sets of reads representing 5–50 × genomic coverage, calculated according to the size of the selected reference genome, were created from the corresponding genus-pool. The sets of reads for each coverage were aligned against the reference genomes and the percentage of reconstructed reference genome was calculated (Figure 2 and Table S8). It was observed that at a genomic coverage of 25 ×, 7 out of the 8 genomes could be 85% assembled and at a coverage of 30 ×, >90% genomic assembly could be achieved in all cases, except *Bacteroides* (84.72%). These results suggest that 25–30 × coverage is sufficient to generate >85% genomic assembly on using the genus-pool of reads from multiple metagenomes.

### Assembly of viral genomes

Since, the number of reads assigned to any viral genus-pool was too low for performing assembly, all the reads which belonged to viruses were pooled together from all 72 datasets and were aligned with the available viral genomes (Table S9). A total of 279 viral genomes could be identified with ≥1% genome assembly, of which 215 were bacteriophage genomes. The maximum (88.4–97.86%) assembly was observed for the 11 different *Lactococcus* phages. The other 56 viruses showed reasonable (10–80%) coverage with most of them being bacteriophages of common human gut bacteria, and the remaining 211 viruses showed low (1–10%) percentage assembly. As expected, the bacteriophages, having more than 10% of coverage, were associated with bacteria belonging to the most abundant bacterial families. The maximum numbers of reads belonged to the *Lactococcus* phage (family *Siphoviridae*) and is harbored by *Streptococcaceae* family, which is among the top 10 bacterial families found in the data. The *Lactococcus* phage has also been reported as highly abundant in human gut datasets (Waller et al., 2014) in earlier studies. The maximum percentage of assembly was obtained for the *Lactococcus* phage P008 (97.86%) associated with *Lactococcus lactis*, which sustains high temperature affecting milk industry (Chen et al., 2013).

### Comparison of assembly using reads with assembly using contigs

**Figure 3:**
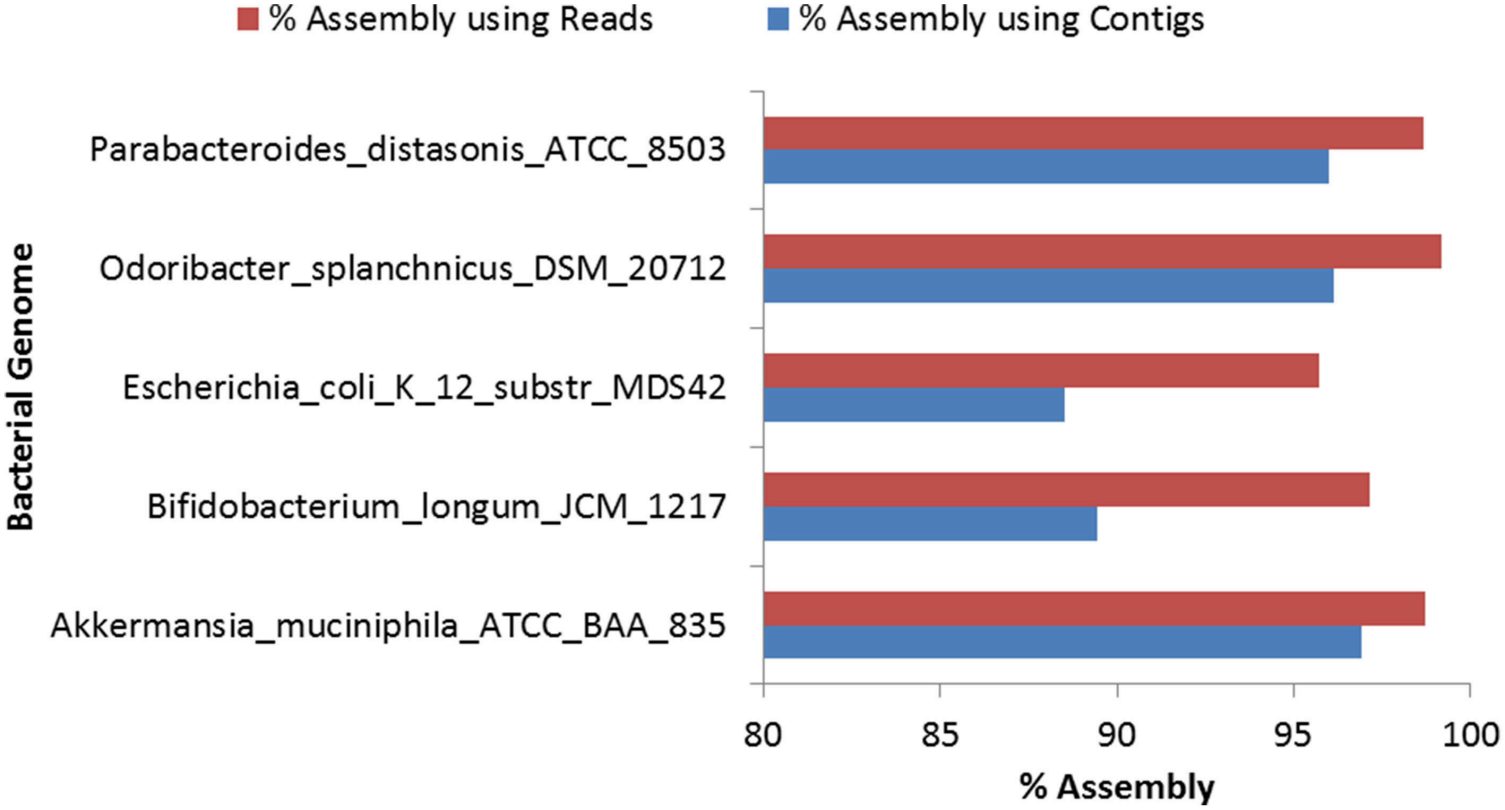
**Comparison of percentage of assembly achieved by aligning reads and contigs for bacterial genomes**.

Assembly of the reads for the eight selected bacterial genomes was carried out using Genovo because of its high accuracy, ability to use maximum number of reads to generate large assembled contigs, along with high N50 values (Vázquez-Castellanos et al., 2014). Genovo, uses an iterative algorithm which estimates the number of genomes in the population and de-noises the metagenomic data (Laserson et al., 2011). However, Genovo assembler is time consuming and memory intensive and hence the assembly was performed for only five genus-pools out of the selected eight genera. Since, the degree of chimericity depends on the contig length, with shorter contigs having a much higher degree of chimericity, only those contigs with length ≥500 bp were considered for further analyses (Mende et al., 2012). Highest N50 (5063) was achieved in the case of *O. splanchnicus* DSM_20712. The detailed statistics of the assembly process are provided in Table S10. The alignment of resultant contigs was carried out using BWA with the most abundant bacterial genome in that genus as predicted by Kraken. The alignment of draft genome (constructed using contigs) with the respective reference genome showed a high (95–99%) identity. In a recent study, assembly using Genovo has been shown to be prone to some chimerism (Vázquez-Castellanos et al., 2014), therefore, 50 iterations were performed to minimize the possibility of formation of chimeric contigs. Furthermore, the high alignment identity and N50 values achieved for the above assemblies attests to high level of accuracy in the assembly and thus, indicate low possibility of existence of chimeric contigs. 96.91% assembly could be achieved by the alignment of the contigs with the *A. muciniphila* ATCC BAA 835, as compared to 98.71% assembly achieved when the reads were aligned directly with the same genome (Table S11). Similarly, for the other remaining genomes, higher (95.7–99.2%) percentage of assembly was obtained when the reads were directly used for the alignment as compared to the coverage (88.5–96.9%) achieved using contigs for alignment with the respective reference genome (Figure 3). These results indicate that the percentage of assembly achieved on using the direct alignment of reads is comparable to the alignment of contigs, however, a marginally higher percentage of assembly could be achieved when the reads are directly used.

Similarly, the assembly of reads belonging to the viruses from all 72 metagenomic datasets for top five viral genomes (one from each genus) shows that the percentage assembly of the respective viral genome was found to be lower (10.5–94.5%) when the contigs were used for the alignment as compared to the alignment carried out by directly using the reads (49.6–97.9%) (Figure S4 and Table S12).

### Improving the draft assembly using spiking

In public databases, the number of draft assemblies far exceeds the number of completely sequenced bacterial species identified from human gut and from other metagenomic datasets. The main reasons for the inability to achieve a complete genomic sequence in most cases is the absence of reads from some genomic regions after sequencing which remain as gaps in the draft genomes, and due to repeated regions in a genome which are bigger than the read length. Though, the latter problem cannot be resolved without longer read lengths along with high sequencing depths, however, for the former scenario, it would be interesting to see if the human gut metagenomic reads could be used to fill up these gaps to improve the draft assemblies of genomes available from human gut. To examine this hypothesis, a simulated draft genome was constructed using the complete genome sequence of *O. splanchnicus* DSM 20712, which is a bacteria found in human gut. A total of 3,993,300 bases were substituted by stretches of “n,” each stretch of “*n*” = 700 bp, at 570 different sites in the complete genome. The draft genome constructed using this approach showed an alignment of 90.91% with the complete genome. The simulated reads were generated at 200 × coverage from this draft genome and were added with the genus-pool of reads for the genus *Odoribacter* resulting in a spiked metagenomic dataset for *O. splanchnicus DSM 20712*. *De novo* assembly of this spiked set of reads was performed using Genovo with 50 iterations and the resulting contigs were mapped on the complete reference genome. It was observed that the spiked set of metagenomic reads could improve the percentage of assembly from 90.91 to 98.45% with the reference genome. The gaps (n's) in the simulated draft genome were replaced with nucleotide bases with 98.9% accuracy in the final constructed draft genome (Table S13 and Figure S5). *De novo* assembly was also performed using SOAPdenovo2 for this spiked metagenomic dataset, however, the percentage assembly (96.99%) achieved and the accuracy (98.5%) of the replaced n's with the nucleotide bases was lower as compared to Genovo based *de novo* assembly.

## Discussion

While the current work was in progress, a different approach of cumulating metagenomes to assemble new microbial species from multiple metagenomes was carried out by Nielsen et al. (as described in the Introduction Section). However, a completely different and novel Binning-Assembly (BA) approach is demonstrated in the present work to reconstruct the bacterial genomes from multiple metagenomes. Using the BA approach, a total of 31 phylum, 219 families, 584 genera and 446 bacterial species and 279 viral species were identified from 72 human gut datasets, whereas, the MGS approach reported the presence of 741 MGS including bacterial and viral species from 396 datasets. The number of reported species is higher in the later study as it was carried out using much larger number of datasets. The major difference in the two approaches is that in the present study the reference genomes have been used to reconstruct the genomic assemblies, whereas, in the study by Nielsen et al., MGS were constructed from gut without using any reference genomes.

Out of the 1156 bacterial genomes identified in this study, >50% assembly could be achieved for 181 genomes. Furthermore, 126 bacterial genomes and 11 viral genomes could be reconstructed with >80% assembly which asserts the usability of this approach to reconstruct genomes from a metagenomic mixture of reads. The acceptance of metagenome-derived genomes may be arguable due to the assembly of regions of a bacterial species using metagenomic mix of reads obtained from multiple samples of same environment, such as human gut in this study. Therefore, in this work, multiple steps were taken to ensure high accuracy of the reconstructed genomes. At the first step, the reads belonging only to bacterial kingdom were selected after the taxonomic assignment of all reads by Kraken which ensures that the eukaryotic and viral reads are removed before proceeding for assembly. Furthermore, consideration of reads only from a single genus by constructing the genus-pool for each genus removes the possibility of the presence of reads from other genera which makes the assembly process more specific and less complex.

Though, it could be argued that the mixing of reads from multiple metagenomes to form a genus-pool might result in some chimerism during the assembly, however, in the case of all eight resultant draft genomes in this manuscript, the observed high (>95%) identity of the assembled genomes with the respective reference genome attests to the accuracy of the assembly. Further verification of the assembled genomes was performed by examining the completeness of the ORF's of the reconstructed genomes, and this analysis revealed 99.8% of the ORF's to be completely present with 99.8% identity. In case of *de novo* assembly, Genovo assembler was used with 50 iterations in place of commonly used 40 iterations (Smits et al., 2014), to achieve high accuracy (95–99%) in the resultant contigs. Taken together, these results underscore the validity and accuracy of the reconstructed genomes.

It is apparent that the achievable percentage assembly of a genome depends upon its abundance (number of reads) in the metagenome. The strategy-I reveals that an abundant genomic species can be easily assembled up to 91% with a minimal 8 × coverage using reads from a single metagenome. Therefore, for abundant species, the reconstruction should first be attempted using only a single metagenome. However, in general, for the assembly of any genomic species (irrespective of its abundance), the strategy-II shows that a sequencing depth of 25 × –30 × of that species is sufficient to achieve >85% assembly of that genome which also concurs with previous reports (Chitsaz et al., 2011; Liu et al., 2012).

Promising results were also achieved by spiking the “genus-pool” of reads with the reads of a simulated draft genome belonging to that genus. The gap regions “n” in the simulated draft genome could be replaced by nucleotides with 98.5% accuracy and could improve the assembly from 90.9 to 96.9%. This appears to be a useful strategy to improve the assembly of the incomplete draft genomes which outnumbers the completed genomes in the public databases.

An apparent limitation of the current approach is the dependence on the classification accuracy and efficiency of binning algorithm which is limited at this point mainly due to the lower read lengths and unavailability of reference genomic sequences in the public databases, which is expected to improve with time. It is to be noted that only 1/3rd of the total reads (32.56%) could be classified into taxonomic groups using Kraken and on using only these reads, 90 high quality draft genomes with >90% assembly could be reconstructed by using BA approach. Furthermore, no improvement was observed in the assemblies after the addition of leftover reads (Text S1). These results attest the accuracy of taxonomic classification by Kraken, but also point toward its limited classification ability, which however will improve with the availability of number of reference genomes for training and improvements in the binning methodology. Furthermore, the read lengths obtained in different sequencing technologies are also becoming longer which is likely to improve the classification ability and hence, would further improve the reconstruction of genomes using the BA approach.

Another limitation of this approach is its dependence on reference genomes for alignment to reconstruct the genome sequences. This limits its usability for those bacterial genomes for which a closely related reference genome is available. However, more and more bacterial genomes are being sequenced worldwide at a rapid rate and in this scenario, the main advantage of this approach is the rapid and reliable reconstruction of strains of the known species or closely related members of the known species which are likely to be present in different populations or environments.

The analysis presented in this study demonstrates the merits and limitations of binning and assembly based approach and thus it is likely to act as a reference for the reconstruction of bacterial genomes from metagenomic reads.

## Author contributions

SK, AG, and VS developed the idea. AG, SK, VP, KH performed the analysis. AG and AS developed the scripts. SK, AG, and VS wrote the manuscript.

## Funding

We thank the intramural funding received from IISER Bhopal for carrying out this work. AG is a recipient of DST-INSPIRE Fellowship and thanks the Department of Science and Technology for the fellowship.

### Conflict of interest statement

The authors declare that the research was conducted in the absence of any commercial or financial relationships that could be construed as a potential conflict of interest.
