# Supplementary material for: Reconstruction of Bacterial and Viral Genomes from Multiple Metagenomes
Source: Front Microbiol. 2016 Apr 12;7:469. doi: 10.3389/fmicb.2016.00469 (PMC4828583; doi:10.3389/fmicb.2016.00469)
Supplement: Supplementary file 1 [file Table1.DOCX]

**Table S1. Detailed description of the raw and processed data of all the metagenomic datasets used for this study and the percentage classification shown by Kraken for each metagenome.** The metagenomes selected for the analysis are shown in bold. 'Unique in forward' and 'Unique in reverse' represents 'Initially rejected reads'. 'Flash not combined' represents 'Flash rejected reads'.

| **Metagenome labeled as per technical replicates** | **Total Count of Raw Reads** | | **Read Count after Sorting Metagenomic Reads for FLASH Input** | | | **Read Count After Combining Using FLASH** | | **% FLASH Reads combined** | **% Kraken Classification** |
| --- | --- | --- | --- | --- | --- | --- | --- | --- | --- |
| **Metagenomic Data** | **Forward** | **Reverse** | **Common Reads in Forward & Reverse** | **Unique in Forward** | **Unique in Reverse** | **FLASH Combined** | **FLASH Not Combined** |  |  |
| **MH0002_081224** | 11166973 | 11167277 | 11165183 | 1790 | 2094 | 8862690 | 2302493 | 79.38 | 41.91 |
| **MH0003_081203** | 4477830 | 4477934 | 4477312 | 518 | 622 | 2733307 | 1744005 | 61.05 | 36.98 |
| **MH0003_081224** | 10856726 | 10856618 | 10856019 | 707 | 599 | 8825085 | 2030934 | 81.29 | 36.49 |
| **MH0009_081224** | 11285597 | 11286038 | 11283995 | 1602 | 2043 | 7233081 | 4050914 | 64.10 | 21.3 |
| **MH0011_081203** | 4957206 | 4957434 | 4956118 | 1088 | 1316 | 4282768 | 673350 | 86.41 | 25.82 |
| **MH0011_081224** | 11091156 | 11091909 | 11088635 | 2521 | 3274 | 7593269 | 3495366 | 68.48 | 25.19 |
| **MH0012_081203** | 5074588 | 5074552 | 5074119 | 469 | 433 | 4935394 | 138725 | 97.27 | 79.34 |
| **MH0012_081224** | 10688612 | 10688557 | 10687883 | 729 | 674 | 9199323 | 1488560 | 86.07 | 79.24 |
| **MH0014_081203** | 4779090 | 4779411 | 4778196 | 894 | 1215 | 4389501 | 388695 | 91.87 | 38.9 |
| **MH0014_081224** | 10030943 | 10032618 | 10029059 | 1884 | 3559 | 7537929 | 2491130 | 75.16 | 38.5 |
| **MH0016_081203** | 4840407 | 4840503 | 4840022 | 385 | 481 | 4593571 | 246451 | 94.91 | 36.83 |
| **MH0016_081224** | 9034646 | 9035871 | 9033892 | 754 | 1979 | 8288896 | 744996 | 91.75 | 36.68 |
| **MH0020_081224** | 10103617 | 10101992 | 10094227 | 9390 | 7765 | 6967439 | 3126788 | 69.02 | 44.47 |
| **MH0021_081124** | 8960070 | 8960899 | 8958733 | 1337 | 2166 | 7784047 | 1174686 | 86.89 | 34.19 |
| **MH0024_081224** | 10712799 | 10713358 | 10710932 | 1867 | 2426 | 9620204 | 1090728 | 89.82 | 25.66 |
| **MH0025_090120** | 11438262 | 11438357 | 11437582 | 680 | 775 | 8699143 | 2738439 | 76.06 | 36.98 |
| **MH0028_081223** | 10318197 | 10317884 | 10317483 | 714 | 401 | 1667373 | 833012 | 91.93 | 30.14 |
| **MH0030_081224** | 10133195 | 10132455 | 10126724 | 6471 | 5731 | 7870473 | 2256251 | 77.72 | 21.36 |
| **MH0031_081223** | 10774731 | 10777088 | 10768394 | 6337 | 8694 | 9711428 | 1056966 | 90.18 | 18.51 |
| **MH0032_081224** | 9138885 | 9139241 | 9133455 | 5430 | 5786 | 7022386 | 2111069 | 76.89 | 22.97 |
| **MH0033_081224** | 9130715 | 9131145 | 9129898 | 817 | 1247 | 5656541 | 3473357 | 61.96 | 36.84 |
| **MH0035_081223** | 10932571 | 10936598 | 10922014 | 10557 | 14584 | 9946067 | 975947 | 91.06 | 31.15 |
| **MH0036_081226** | 10485163 | 10484660 | 10483020 | 2143 | 1640 | 8634441 | 1848579 | 83.37 | 32.61 |
| **MH0037_081226** | 8644291 | 8644091 | 8643128 | 1163 | 963 | 4993677 | 3649451 | 57.78 | 29.33 |
| **MH0038_081226** | 9575758 | 9572557 | 9567382 | 8376 | 5175 | 5664620 | 3902762 | 59.21 | 28.54 |
| **MH0039_081126** | 8839640 | 8839455 | 8838893 | 747 | 562 | 6003485 | 2835408 | 67.92 | 37.54 |
| **MH0040_081223** | 8680047 | 8679877 | 8679313 | 734 | 564 | 7835129 | 844184 | 90.27 | 31.69 |
| **MH0041_081222** | 9036377 | 9036779 | 9032917 | 3460 | 3862 | 7584332 | 1448585 | 83.96 | 37.27 |
| **MH0042_081226** | 8807526 | 8811721 | 8802750 | 4776 | 8971 | 6370709 | 2432041 | 72.37 | 27.02 |
| **MH0043_081214** | 11618517 | 11621067 | 11610294 | 8223 | 10773 | 9510440 | 2099854 | 81.91 | 35.27 |
| **MH0044_081222** | 9172000 | 9171922 | 9171391 | 609 | 531 | 7953538 | 1217853 | 86.72 | 34.69 |
| **MH0045_081223** | 11545785 | 11545689 | 11544188 | 1597 | 1501 | 10652457 | 891731 | 92.28 | 36.81 |
| **MH0046_081230** | 6052897 | 6054628 | 6052183 | 714 | 2445 | 3724091 | 2328092 | 61.53 | 24.72 |
| **MH0047_081223** | 10770637 | 10770672 | 10769772 | 865 | 900 | 9553406 | 1216366 | 88.71 | 20.28 |
| **MH0048_081226** | 10565677 | 10565318 | 10563584 | 2093 | 1734 | 6914173 | 3649411 | 65.45 | 26.35 |
| **MH0049_081223** | 10935828 | 10935774 | 10935278 | 550 | 496 | 9971154 | 964124 | 91.18 | 25.8 |
| **MH0050_081223** | 11557102 | 11557125 | 11556341 | 761 | 784 | 9023108 | 2533233 | 86.70 | 24.17 |
| **MH0051_081226** | 10154856 | 10154884 | 10151349 | 3507 | 3535 | 6997599 | 3153750 | 75.46 | 34.83 |
| **MH0052_090103** | 10593717 | 10593302 | 10592713 | 1004 | 589 | 8820190 | 1772523 | 83.27 | 23.64 |
| **MH0053_081222** | 8624989 | 8625063 | 8624377 | 612 | 686 | 7667658 | 956719 | 88.91 | 20.11 |
| **MH0054_081222** | 8494281 | 8494229 | 8493115 | 1166 | 1114 | 7376166 | 1116949 | 86.85 | 32.71 |
| **MH0055_081223** | 10019796 | 10020033 | 10018598 | 1198 | 1435 | 9027031 | 991567 | 90.10 | 25.6 |
| **MH0057_081222** | 8046876 | 8046890 | 8046008 | 868 | 882 | 6683306 | 1362702 | 83.06 | 24.54 |
| **MH0058_081230** | 11596992 | 11601827 | 11595122 | 1870 | 6705 | 8232949 | 3362173 | 71.00 | 36.12 |
| **MH0059_081230** | 10014810 | 10020361 | 10012573 | 2237 | 7788 | 7170979 | 2841594 | 71.62 | 34.85 |
| **MH0060_081222** | 8422904 | 8422795 | 8422281 | 623 | 514 | 6853421 | 1568860 | 81.37 | 23.64 |
| **MH0061_090103** | 11079516 | 11073400 | 11046354 | 33162 | 27046 | 9483439 | 1562915 | 85.85 | 40.54 |
| **MH0062_081223** | 9418946 | 9434289 | 9350300 | 68646 | 83989 | 7811421 | 1538879 | 83.54 | 36.01 |
| **MH0063_090103** | 11839551 | 11838888 | 11837789 | 1762 | 1099 | 9977286 | 1860503 | 84.28 | 19.94 |
| **MH0064_081223** | 11169216 | 11169553 | 11167478 | 1738 | 2075 | 9586528 | 1580950 | 85.84 | 25.19 |
| **MH0065_081223** | 11219529 | 11219522 | 11217122 | 2407 | 2400 | 9408609 | 1808513 | 83.88 | 24.15 |
| **MH0066_081223** | 10944699 | 10944425 | 10943896 | 803 | 529 | 8366227 | 2577669 | 76.45 | 32.14 |
| **MH0067_090103** | 11600809 | 11600392 | 11600193 | 616 | 199 | 9903827 | 1696366 | 85.38 | 33.08 |
| **MH0068_081223** | 11098691 | 11098721 | 11097318 | 1373 | 1403 | 9013254 | 2084064 | 81.22 | 58.43 |
| **MH0069_090103** | 11960071 | 11959700 | 11959007 | 1064 | 693 | 7319023 | 4639984 | 61.20 | 32.53 |
| **MH0070_090104** | 11322886 | 11324394 | 11320404 | 2482 | 3990 | 9435807 | 1884597 | 83.35 | 27.68 |
| **MH0071_090104** | 11197082 | 11197711 | 11194636 | 2446 | 3075 | 10210439 | 984197 | 91.20 | 28.24 |
| **MH0072_081223** | 12102801 | 12103046 | 12098158 | 4643 | 4888 | 8527509 | 3570649 | 70.49 | 50.54 |
| **MH0073_081223** | 11029318 | 11029432 | 11027848 | 1470 | 1584 | 7527257 | 3500591 | 86.65 | 38.12 |
| **MH0074_081223** | 11172758 | 11172374 | 11172112 | 646 | 262 | 8666605 | 2505507 | 77.57 | 33.93 |
| **MH0075_090104** | 11674285 | 11673980 | 11673692 | 593 | 288 | 10563923 | 1109769 | 90.49 | 26.75 |
| **MH0076_081223** | 11204459 | 11205419 | 11202008 | 2451 | 3411 | 10433157 | 768851 | 93.14 | 32.48 |
| **MH0077_081223** | 11034859 | 11035407 | 11031485 | 3374 | 3922 | 10023832 | 1007653 | 90.87 | 28.67 |
| **MH0078_081222** | 8576035 | 8579185 | 8559662 | 16373 | 19523 | 5926467 | 2633195 | 69.24 | 19.08 |
| **MH0079_090104** | 10412151 | 10411988 | 10411670 | 481 | 318 | 9715242 | 696428 | 93.31 | 22.19 |
| **MH0080_090109** | 13040255 | 13042473 | 13038927 | 1328 | 3546 | 11034374 | 2004553 | 84.63 | 38.7 |
| **MH0081_090105** | 10866700 | 10866495 | 10866009 | 691 | 486 | 10004209 | 861800 | 92.07 | 29.09 |
| **MH0082_090105** | 11382136 | 11382431 | 11380428 | 1708 | 2003 | 9941460 | 1438968 | 87.36 | 32.66 |
| **MH0083_090105** | 11684284 | 11684012 | 11683722 | 562 | 290 | 10878026 | 805696 | 93.10 | 35.37 |
| **MH0084_090109** | 13733793 | 13735105 | 13732684 | 1109 | 2421 | 11730312 | 2002372 | 85.42 | 19.8 |
| **MH0085_081223** | 11429782 | 11429697 | 11429041 | 741 | 656 | 10288486 | 1140555 | 90.02 | 34.91 |
| **MH0086_081223** | 11217184 | 11216960 | 11216331 | 853 | 629 | 8115944 | 3100387 | 72.36 | 36.76 |
| MH0009_081203 | 4876830 | 4877229 | 4876142 | 688 | 1087 | 2468998 | 2407144 | 50.63 | Not done |
| MH0034_081213 | 4807230 | 4807030 | 4806843 | 387 | 187 | 2332480 | 2474363 | 48.52 | Not done |
| MH0069_081209 | 10854240 | 10861072 | 10853791 | 449 | 7281 | 4094422 | 6759369 | 37.72 | Not done |
| MH0056_081230 | 11327680 | 11389275 | 11324515 | 3165 | 64760 | 3987997 | 7336518 | 35.22 | Not done |
| MH0026_081224 | 10894176 | 10893938 | 10893200 | 976 | 738 | 3707442 | 7185758 | 34.03 | Not done |
| MH0002_081203 | 4189859 | 4189961 | 4189308 | 551 | 653 | 1267095 | 2922213 | 30.25 | Not done |
| MH0079_090130 | 3296600 | 3298160 | 3295805 | 795 | 2355 | 365717 | 2930088 | 11.10 | Not done |
| MH0076_090130 | 4119851 | 4120377 | 4119175 | 676 | 1202 | 447260 | 3671915 | 10.86 | Not done |
| MH0065_090126 | 11070459 | 11070285 | 11069860 | 599 | 425 | 1193609 | 9876251 | 10.78 | Not done |
| MH0052_090201 | 3693833 | 3694184 | 3693580 | 253 | 604 | 383683 | 3309897 | 10.39 | Not done |
| MH0021_090201 | 4168661 | 4168696 | 4168325 | 336 | 371 | 427469 | 3740856 | 10.26 | Not done |
| MH0082_090130 | 12791674 | 12791713 | 12790727 | 947 | 986 | 1305257 | 11485470 | 10.20 | Not done |
| MH0062_090104 | 8962070 | 8993919 | 8905846 | 56224 | 88073 | 907535 | 7998311 | 10.19 | Not done |
| MH0066_090126 | 7112923 | 7112950 | 7112559 | 364 | 391 | 724747 | 6387812 | 10.19 | Not done |
| MH0080_090130 | 10253842 | 10254179 | 10253124 | 718 | 1055 | 1044217 | 9208907 | 10.18 | Not done |
| MH0078_090130 | 4447780 | 4448537 | 4446341 | 1439 | 2196 | 451843 | 3994498 | 10.16 | Not done |
| MH0077_090130 | 13794955 | 13795294 | 13793388 | 1567 | 1906 | 1399479 | 12393909 | 10.15 | Not done |
| MH0069_090125 | 12043764 | 12043373 | 12043015 | 749 | 358 | 1219215 | 10823800 | 10.12 | Not done |
| MH0081_090130 | 13059443 | 13059259 | 13059070 | 373 | 189 | 1321654 | 11737416 | 10.12 | Not done |
| MH0048_090201 | 2920851 | 2921124 | 2920218 | 633 | 906 | 294938 | 2625280 | 10.10 | Not done |
| MH0035_090122 | 13709781 | 13710289 | 13705983 | 3798 | 4306 | 1369239 | 12336744 | 9.99 | Not done |
| MH0047_090201 | 2695125 | 2695630 | 2694802 | 323 | 828 | 268642 | 2426160 | 9.97 | Not done |
| MH0059_090105 | 10495359 | 10495076 | 10494611 | 748 | 465 | 1040413 | 9454198 | 9.91 | Not done |
| MH0083_090126 | 12267910 | 12267600 | 12267365 | 545 | 235 | 1215096 | 11052269 | 9.91 | Not done |
| MH0070_090125 | 12637416 | 12636898 | 12636427 | 989 | 471 | 1250821 | 11385606 | 9.90 | Not done |
| MH0067_090126 | 10641841 | 10641595 | 10641529 | 312 | 66 | 1049799 | 9591730 | 9.87 | Not done |
| MH0056_090125 | 13251423 | 13251093 | 13242905 | 8518 | 8188 | 1305099 | 11937806 | 9.86 | Not done |
| MH0053_090125 | 13017068 | 13016444 | 13016035 | 1033 | 409 | 1280057 | 11735978 | 9.85 | Not done |
| MH0054_090125 | 12637397 | 12636841 | 12636094 | 1303 | 747 | 1244633 | 11391461 | 9.85 | Not done |
| MH0086_090325 | 16500404 | 16499119 | 16498965 | 1439 | 154 | 1624217 | 14874748 | 9.84 | Not done |
| MH0063_090126 | 11141408 | 11141182 | 11140970 | 438 | 212 | 1095044 | 10045926 | 9.83 | Not done |
| MH0055_090125 | 13536703 | 13535775 | 13535415 | 1288 | 360 | 1329041 | 12206374 | 9.82 | Not done |
| MH0011_090120 | 13202808 | 13202845 | 13201615 | 1193 | 1230 | 1294693 | 11906922 | 9.81 | Not done |
| MH0075_090125 | 11858630 | 11858208 | 11858110 | 520 | 98 | 1159082 | 10699028 | 9.77 | Not done |
| MH0064_090126 | 11196850 | 11196678 | 11196413 | 437 | 265 | 1091626 | 10104787 | 9.75 | Not done |
| MH0042_090125 | 12902190 | 12902615 | 12900414 | 1776 | 2201 | 1254802 | 11645612 | 9.73 | Not done |
| MH0033_090122 | 13367314 | 13366959 | 13366786 | 528 | 173 | 1298328 | 12068458 | 9.71 | Not done |
| MH0060_090125 | 12133394 | 12133134 | 12132695 | 699 | 439 | 1178361 | 10954334 | 9.71 | Not done |
| MH0058_090125 | 13312472 | 13311707 | 13311136 | 1336 | 571 | 1288680 | 12022456 | 9.68 | Not done |
| MH0043_090125 | 13060920 | 13061749 | 13058081 | 2839 | 3668 | 1263098 | 11794983 | 9.67 | Not done |
| MH0044_090125 | 13260049 | 13260109 | 13259545 | 504 | 564 | 1282256 | 11977289 | 9.67 | Not done |
| MH0061_090126 | 11180598 | 11181968 | 11175648 | 4950 | 6320 | 1081179 | 10094469 | 9.67 | Not done |
| MH0028_090121 | 17256930 | 17259508 | 17256242 | 688 | 3266 | 9484471 | 15588869 | 9.66 | Not done |
| MH0036_090122 | 13368541 | 13368271 | 13367401 | 1140 | 870 | 1291046 | 12076355 | 9.66 | Not done |
| MH0038_090122 | 13163624 | 13163452 | 13162165 | 1459 | 1287 | 1271300 | 11890865 | 9.66 | Not done |
| MH0039_090122 | 12519215 | 12518874 | 12518664 | 551 | 210 | 1209137 | 11309527 | 9.66 | Not done |
| MH0073_090125 | 12588097 | 12587354 | 12586444 | 1653 | 910 | 1215279 | 11371165 | 9.66 | Not done |
| MH0068_090126 | 10835126 | 10834996 | 10834690 | 436 | 306 | 1044487 | 9790203 | 9.64 | Not done |
| MH0040_090125 | 13445033 | 13444775 | 13444486 | 547 | 289 | 1294076 | 12150410 | 9.63 | Not done |
| MH0049_090201 | 4391565 | 4391675 | 4391268 | 297 | 407 | 422696 | 3968572 | 9.63 | Not done |
| MH0050_090201 | 12339056 | 12338750 | 12338464 | 592 | 286 | 1188371 | 11150093 | 9.63 | Not done |
| MH0030_090121 | 15676620 | 15680533 | 15672721 | 3899 | 7812 | 1505401 | 14167320 | 9.61 | Not done |
| MH0002_090117 | 7928251 | 7927774 | 7927536 | 715 | 238 | 760882 | 7166654 | 9.60 | Not done |
| MH0037_090122 | 12373035 | 12372682 | 12372278 | 757 | 404 | 1187451 | 11184827 | 9.60 | Not done |
| MH0045_090125 | 13171382 | 13171445 | 13170508 | 874 | 937 | 1264810 | 11905698 | 9.60 | Not done |
| MH0024_090121 | 16743506 | 16746322 | 16742504 | 1002 | 3818 | 1606309 | 15136195 | 9.59 | Not done |
| MH0025_090121 | 16362156 | 16365816 | 16361297 | 859 | 4519 | 1568936 | 14792361 | 9.59 | Not done |
| MH0016_090120 | 13322252 | 13322342 | 13321829 | 423 | 513 | 1276666 | 12045163 | 9.58 | Not done |
| MH0031_090121 | 16992262 | 16996182 | 16987719 | 4543 | 8463 | 1621151 | 15366568 | 9.54 | Not done |
| MH0071_090125 | 11923933 | 11923547 | 11922800 | 1133 | 747 | 1136293 | 10786507 | 9.53 | Not done |
| MH0003_090107 | 9858042 | 9857719 | 9857500 | 542 | 219 | 938916 | 8918584 | 9.52 | Not done |
| MH0012_090120 | 13150674 | 13150815 | 13150141 | 533 | 674 | 1252133 | 11898008 | 9.52 | Not done |
| MH0046_090125 | 11845298 | 11845281 | 11844870 | 428 | 411 | 1126830 | 10718040 | 9.51 | Not done |
| MH0051_090201 | 2826513 | 2826851 | 2825973 | 540 | 878 | 57577 | 2768396 | 9.50 | Not done |
| MH0009_090109 | 13063446 | 13063364 | 13062929 | 517 | 435 | 1239963 | 11822966 | 9.49 | Not done |
| MH0074_090125 | 11842894 | 11842479 | 11842386 | 508 | 93 | 1122351 | 10720035 | 9.48 | Not done |
| MH0041_090125 | 13154146 | 13155178 | 13151792 | 2354 | 3386 | 1244079 | 11907713 | 9.46 | Not done |
| MH0014_090120 | 13341364 | 13341340 | 13340573 | 791 | 767 | 1260884 | 12079689 | 9.45 | Not done |
| MH0020_090120 | 12803883 | 12804154 | 12803061 | 822 | 1093 | 1197322 | 11605739 | 9.35 | Not done |
| MH0084_090126 | 13048496 | 13048421 | 13047840 | 656 | 581 | 1220508 | 11827332 | 9.35 | Not done |
| MH0085_090126 | 13287702 | 13287310 | 13287131 | 571 | 179 | 1240003 | 12047128 | 9.33 | Not done |
| MH0072_090125 | 12540605 | 12540220 | 12539905 | 700 | 315 | 1166947 | 11372958 | 9.31 | Not done |
| MH0057_090125 | 13478346 | 13477916 | 13477358 | 988 | 558 | 1249442 | 12227916 | 9.27 | Not done |
| MH0032_090121 | 15914320 | 15917621 | 15913587 | 733 | 4034 | 1473870 | 14439717 | 9.26 | Not done |
| MH0034_090122 | 13893487 | 13893150 | 13893002 | 485 | 148 | 1286135 | 12606867 | 9.26 | Not done |
| MH0026_090117 | 7848259 | 7847693 | 7847511 | 748 | 182 | 710880 | 7136631 | 9.06 | Not done |
