# Supplementary material for: Reconstruction of Bacterial and Viral Genomes from Multiple Metagenomes
Source: Front Microbiol. 2016 Apr 12;7:469. doi: 10.3389/fmicb.2016.00469 (PMC4828583; doi:10.3389/fmicb.2016.00469)
Supplement: Supplementary file 2 [file Table2.DOCX]

**Table S2. Total number of reads and their percentage abundance in the identified bacterial families and genera in all 72 metagenomes.** Percentage abundance of a family is calculated by dividing the total reads in the family with the total reads classified in all families.

| **GENUS** | **# Reads** | **% Abundance** | **% Cumulative Abundance** |  | **FAMILY** | **# Reads** | **% Abundance** | **% Cumulative Abundance** |
| --- | --- | --- | --- | --- | --- | --- | --- | --- |
| *Bacteroides* | 53089988 | 31.649 | 31.649 |  | *Bacteroidaceae* | 53089988 | 31.371 | 31.371 |
| *Eubacterium* | 18834513 | 11.228 | 42.877 |  | *Ruminococcaceae* | 20631793 | 12.192 | 43.563 |
| *Faecalibacterium* | 14324227 | 8.539 | 51.417 |  | *Eubacteriaceae* | 19710192 | 11.647 | 55.210 |
| *Alistipes* | 9079576 | 5.413 | 56.830 |  | *Lachnospiraceae* | 17004059 | 10.048 | 65.258 |
| *Roseburia* | 8031335 | 4.788 | 61.617 |  | *Porphyromonadaceae* | 9668730 | 5.713 | 70.971 |
| *Parabacteroides* | 6260450 | 3.732 | 65.350 |  | *Rikenellaceae* | 9086210 | 5.369 | 76.340 |
| *Ruminococcus* | 5433123 | 3.239 | 68.588 |  | *Clostridiaceae* | 5018175 | 2.965 | 79.305 |
| *Blautia* | 4065893 | 2.424 | 71.012 |  | *Enterobacteriaceae* | 3726117 | 2.202 | 81.507 |
| *Clostridium* | 3418988 | 2.038 | 73.051 |  | *Prevotellaceae* | 3273521 | 1.934 | 83.442 |
| *Coprococcus* | 3401750 | 2.028 | 75.079 |  | *Bifidobacteriaceae* | 1315206 | 0.777 | 84.219 |
| *Prevotella* | 3253696 | 1.940 | 77.018 |  | *Streptococcaceae* | 1280565 | 0.757 | 84.975 |
| *Odoribacter* | 2866067 | 1.709 | 78.727 |  | *Verrucomicrobiaceae* | 1276640 | 0.754 | 85.730 |
| *Escherichia* | 1571615 | 0.937 | 79.664 |  | *Bacillaceae* | 915460 | 0.541 | 86.271 |
| *Candidatus_Arthromitus* | 1510327 | 0.900 | 80.564 |  | *Burkholderiaceae* | 707056 | 0.418 | 86.689 |
| *Bifidobacterium* | 1295716 | 0.772 | 81.336 |  | *Flavobacteriaceae* | 685496 | 0.405 | 87.094 |
| *Akkermansia* | 1276640 | 0.761 | 82.098 |  | *Oscillospiraceae* | 675010 | 0.399 | 87.492 |
| *Streptococcus* | 1184653 | 0.706 | 82.804 |  | *Coriobacteriaceae* | 647408 | 0.383 | 87.875 |
| *Acetobacterium* | 874544 | 0.521 | 83.325 |  | *Veillonellaceae* | 624531 | 0.369 | 88.244 |
| *Ethanoligenens* | 812897 | 0.485 | 83.810 |  | *Pseudomonadaceae* | 594014 | 0.351 | 88.595 |
| *Bacillus* | 699221 | 0.417 | 84.227 |  | *Streptomycetaceae* | 525000 | 0.310 | 88.905 |
| *Oscillibacter* | 675010 | 0.402 | 84.629 |  | *Peptostreptococcaceae* | 503987 | 0.298 | 89.203 |
| *Cellulosilyticum* | 613050 | 0.365 | 84.994 |  | *Peptococcaceae* | 499571 | 0.295 | 89.498 |
| *Pseudomonas* | 554064 | 0.330 | 85.325 |  | *Lactobacillaceae* | 499114 | 0.295 | 89.793 |
| *Streptomyces* | 472046 | 0.281 | 85.606 |  | *Erysipelotrichaceae* | 448539 | 0.265 | 90.058 |
| *Lactobacillus* | 459313 | 0.274 | 85.880 |  | *Spirochaetaceae* | 441096 | 0.261 | 90.319 |
| *Burkholderia* | 449025 | 0.268 | 86.148 |  | *Pasteurellaceae* | 429605 | 0.254 | 90.573 |
| *Peptoclostridium* | 431399 | 0.257 | 86.405 |  | *Desulfovibrionaceae* | 419742 | 0.248 | 90.821 |
| *Butyrivibrio* | 413482 | 0.246 | 86.651 |  | *Comamonadaceae* | 400890 | 0.237 | 91.058 |
| *Desulfovibrio* | 400420 | 0.239 | 86.890 |  | *Acidaminococcaceae* | 361852 | 0.214 | 91.272 |
| *Acidaminococcus* | 361852 | 0.216 | 87.106 |  | *Rhizobiaceae* | 341123 | 0.202 | 91.473 |
| *Mycoplasma* | 313184 | 0.187 | 87.292 |  | *Paenibacillaceae* | 341008 | 0.202 | 91.675 |
| *Haemophilus* | 280931 | 0.167 | 87.460 |  | *Mycoplasmataceae* | 336137 | 0.199 | 91.873 |
| *Mycobacterium* | 265221 | 0.158 | 87.618 |  | *Cytophagaceae* | 323822 | 0.191 | 92.065 |
| *Paenibacillus* | 259615 | 0.155 | 87.773 |  | *Rhodobacteraceae* | 321546 | 0.190 | 92.255 |
| *Tannerella* | 238227 | 0.142 | 87.915 |  | *Campylobacteraceae* | 317148 | 0.187 | 92.442 |
| *Klebsiella* | 233843 | 0.139 | 88.054 |  | *Alcaligenaceae* | 283409 | 0.167 | 92.609 |
| *Enterococcus* | 217386 | 0.130 | 88.184 |  | *Bradyrhizobiaceae* | 276002 | 0.163 | 92.773 |
| *Staphylococcus* | 213121 | 0.127 | 88.311 |  | *Sphingobacteriaceae* | 274999 | 0.162 | 92.935 |
| *Treponema* | 208184 | 0.124 | 88.435 |  | *Mycobacteriaceae* | 274675 | 0.162 | 93.097 |
| *Geobacter* | 207170 | 0.124 | 88.559 |  | *Rhodospirillaceae* | 267937 | 0.158 | 93.256 |
| *Brachyspira* | 203250 | 0.121 | 88.680 |  | *Enterococcaceae* | 265374 | 0.157 | 93.413 |
| *Megasphaera* | 201912 | 0.120 | 88.800 |  | *Thermoanaerobacterales_Family_III._Incertae_Sedis* | 237209 | 0.140 | 93.553 |
| *Porphyromonas* | 189508 | 0.113 | 88.913 |  | *Myxococcaceae* | 234984 | 0.139 | 93.692 |
| *Campylobacter* | 178441 | 0.106 | 89.019 |  | *Xanthomonadaceae* | 234908 | 0.139 | 93.830 |
| *Enterobacter* | 177942 | 0.106 | 89.125 |  | *Staphylococcaceae* | 232966 | 0.138 | 93.968 |
| *Corynebacterium* | 173906 | 0.104 | 89.229 |  | *Geobacteraceae* | 207170 | 0.122 | 94.090 |
| *Selenomonas* | 173269 | 0.103 | 89.332 |  | *Micromonosporaceae* | 204168 | 0.121 | 94.211 |
| *Desulfotomaculum* | 166133 | 0.099 | 89.431 |  | *Brachyspiraceae* | 203250 | 0.120 | 94.331 |
| *Shewanella* | 165481 | 0.099 | 89.530 |  | *Pseudonocardiaceae* | 202845 | 0.120 | 94.451 |
| *Flavobacterium* | 154571 | 0.092 | 89.622 |  | *Thermoanaerobacteraceae* | 201639 | 0.119 | 94.570 |
| *Adlercreutzia* | 152285 | 0.091 | 89.713 |  | *Helicobacteraceae* | 198290 | 0.117 | 94.687 |
| *Methylobacterium* | 139056 | 0.083 | 89.796 |  | *Sphingomonadaceae* | 177227 | 0.105 | 94.792 |
| *Deinococcus* | 138428 | 0.083 | 89.878 |  | *Chlorobiaceae* | 176839 | 0.104 | 94.897 |
| *Eggerthella* | 136405 | 0.081 | 89.960 |  | *Vibrionaceae* | 175090 | 0.103 | 95.000 |
| *Rhizobium* | 134767 | 0.080 | 90.040 |  | *Corynebacteriaceae* | 173906 | 0.103 | 95.103 |
| *Bradyrhizobium* | 131188 | 0.078 | 90.118 |  | *Thermotogaceae* | 173878 | 0.103 | 95.206 |
| *Vibrio* | 128576 | 0.077 | 90.195 |  | *Shewanellaceae* | 165481 | 0.098 | 95.303 |
| *Veillonella* | 125356 | 0.075 | 90.270 |  | *Caulobacteraceae* | 164275 | 0.097 | 95.400 |
| *Caldicellulosiruptor* | 124576 | 0.074 | 90.344 |  | *Rhodocyclaceae* | 151657 | 0.090 | 95.490 |
| *Actinoplanes* | 122204 | 0.073 | 90.417 |  | *Micrococcaceae* | 140546 | 0.083 | 95.573 |
| *Megamonas* | 118617 | 0.071 | 90.488 |  | *Nocardiaceae* | 139220 | 0.082 | 95.655 |
| *Arcobacter* | 116890 | 0.070 | 90.557 |  | *Methylobacteriaceae* | 139056 | 0.082 | 95.738 |
| *Serratia* | 114388 | 0.068 | 90.625 |  | *Deinococcaceae* | 138428 | 0.082 | 95.819 |
| *Cupriavidus* | 113035 | 0.067 | 90.693 |  | *Fusobacteriaceae* | 136669 | 0.081 | 95.900 |
| *Listeria* | 108737 | 0.065 | 90.758 |  | *Alteromonadaceae* | 131394 | 0.078 | 95.978 |
| *Azospirillum* | 107997 | 0.064 | 90.822 |  | *Listeriaceae* | 129813 | 0.077 | 96.054 |
| *Fusobacterium* | 107108 | 0.064 | 90.886 |  | *Acidobacteriaceae* | 126702 | 0.075 | 96.129 |
| *Geobacillus* | 106370 | 0.063 | 90.949 |  | *Leptotrichiaceae* | 123826 | 0.073 | 96.202 |
| *Desulfitobacterium* | 104611 | 0.062 | 91.012 |  | *Nostocaceae* | 123309 | 0.073 | 96.275 |
| *Gordonibacter* | 103997 | 0.062 | 91.074 |  | *Aeromonadaceae* | 116104 | 0.069 | 96.344 |
| *Frankia* | 103273 | 0.062 | 91.135 |  | *Oxalobacteraceae* | 113877 | 0.067 | 96.411 |
| *Helicobacter* | 101449 | 0.060 | 91.196 |  | *Halomonadaceae* | 113373 | 0.067 | 96.478 |
| *Rhodopseudomonas* | 100987 | 0.060 | 91.256 |  | *Thermaceae* | 109826 | 0.065 | 96.543 |
| *Sorangium* | 97619 | 0.058 | 91.314 |  | *Acetobacteraceae* | 105516 | 0.062 | 96.605 |
| *Ralstonia* | 95441 | 0.057 | 91.371 |  | *Frankiaceae* | 103273 | 0.061 | 96.666 |
| *Thermoanaerobacterium* | 94527 | 0.056 | 91.427 |  | *Moraxellaceae* | 99976 | 0.059 | 96.726 |
| *Anaeromyxobacter* | 94466 | 0.056 | 91.484 |  | *Ectothiorhodospiraceae* | 97814 | 0.058 | 96.783 |
| *Synechococcus* | 94225 | 0.056 | 91.540 |  | *Polyangiaceae* | 97619 | 0.058 | 96.841 |
| *Paludibacter* | 93260 | 0.056 | 91.595 |  | *Desulfobacteraceae* | 95628 | 0.057 | 96.898 |
| *Myxococcus* | 92588 | 0.055 | 91.651 |  | *Neisseriaceae* | 95375 | 0.056 | 96.954 |
| *Lactococcus* | 91085 | 0.054 | 91.705 |  | *Phyllobacteriaceae* | 95160 | 0.056 | 97.010 |
| *Sinorhizobium* | 90871 | 0.054 | 91.759 |  | *Propionibacteriaceae* | 90652 | 0.054 | 97.064 |
| *Acidovorax* | 88891 | 0.053 | 91.812 |  | *Cyclobacteriaceae* | 90383 | 0.053 | 97.117 |
| *Borrelia* | 86454 | 0.052 | 91.864 |  | *Leuconostocaceae* | 88983 | 0.053 | 97.170 |
| *Prochlorococcus* | 85630 | 0.051 | 91.915 |  | *Prochlorococcaceae* | 85630 | 0.051 | 97.220 |
| *Xanthomonas* | 84781 | 0.051 | 91.965 |  | *Synergistaceae* | 82972 | 0.049 | 97.269 |
| *Pedobacter* | 83717 | 0.050 | 92.015 |  | *Spiroplasmataceae* | 81952 | 0.048 | 97.318 |
| *Spiroplasma* | 81952 | 0.049 | 92.064 |  | *Chromatiaceae* | 80374 | 0.047 | 97.365 |
| *Thermoanaerobacter* | 79048 | 0.047 | 92.111 |  | *Planctomycetaceae* | 80240 | 0.047 | 97.413 |
| *Mesorhizobium* | 78917 | 0.047 | 92.158 |  | *Hyphomicrobiaceae* | 79590 | 0.047 | 97.460 |
| *Alkaliphilus* | 78627 | 0.047 | 92.205 |  | *Deferribacteraceae* | 79253 | 0.047 | 97.507 |
| *Arthrobacter* | 78617 | 0.047 | 92.252 |  | *Clostridiales_Family_XI._Incertae_Sedis* | 79170 | 0.047 | 97.553 |
| *Spirochaeta* | 78455 | 0.047 | 92.299 |  | *Halanaerobiaceae* | 78838 | 0.047 | 97.600 |
| *Desulfosporosinus* | 78151 | 0.047 | 92.345 |  | *Acholeplasmataceae* | 78504 | 0.046 | 97.646 |
| *Aeromonas* | 77843 | 0.046 | 92.392 |  | *Alicyclobacillaceae* | 76791 | 0.045 | 97.692 |
| *Rhodococcus* | 77305 | 0.046 | 92.438 |  | *Chromobacteriaceae* | 76242 | 0.045 | 97.737 |
| *Agrobacterium* | 77187 | 0.046 | 92.484 |  | *Xanthobacteraceae* | 75997 | 0.045 | 97.782 |
| *Bordetella* | 76133 | 0.045 | 92.529 |  | *Blattabacteriaceae* | 75425 | 0.045 | 97.826 |
| *Propionibacterium* | 75144 | 0.045 | 92.574 |  | *Desulfobulbaceae* | 75302 | 0.044 | 97.871 |
| *Buchnera* | 74859 | 0.045 | 92.619 |  | *Methylococcaceae* | 72868 | 0.043 | 97.914 |
| *Dickeya* | 74190 | 0.044 | 92.663 |  | *Rickettsiaceae* | 71519 | 0.042 | 97.956 |
| *Rhodobacter* | 73099 | 0.044 | 92.706 |  | *Cryomorphaceae* | 68332 | 0.040 | 97.996 |
| *Slackia* | 72843 | 0.043 | 92.750 |  | *Microbacteriaceae* | 67306 | 0.040 | 98.036 |
| *Variovorax* | 71698 | 0.043 | 92.793 |  | *Rhodothermaceae* | 64434 | 0.038 | 98.074 |
| *Cyanothece* | 71643 | 0.043 | 92.835 |  | *Carnobacteriaceae* | 63788 | 0.038 | 98.112 |
| *Salmonella* | 71103 | 0.042 | 92.878 |  | *Geodermatophilaceae* | 62739 | 0.037 | 98.149 |
| *Filifactor* | 71058 | 0.042 | 92.920 |  | *Saprospiraceae* | 61311 | 0.036 | 98.185 |
| *Chlorobium* | 69574 | 0.041 | 92.961 |  | *Chloroflexaceae* | 61202 | 0.036 | 98.221 |
| *Acinetobacter* | 68408 | 0.041 | 93.002 |  | *Anaplasmataceae* | 61172 | 0.036 | 98.258 |
| *Candidatus_Carsonella* | 67706 | 0.040 | 93.043 |  | *Nocardiopsaceae* | 57024 | 0.034 | 98.291 |
| *Rhodospirillum* | 67212 | 0.040 | 93.083 |  | *Leptospiraceae* | 56097 | 0.033 | 98.324 |
| *Sphaerochaeta* | 66891 | 0.040 | 93.123 |  | *Methylophilaceae* | 55586 | 0.033 | 98.357 |
| *Achromobacter* | 66758 | 0.040 | 93.162 |  | *Bartonellaceae* | 53343 | 0.032 | 98.389 |
| *Olsenella* | 66736 | 0.040 | 93.202 |  | *Rivulariaceae* | 51323 | 0.030 | 98.419 |
| *Blattabacterium* | 66518 | 0.040 | 93.242 |  | *Pelobacteraceae* | 50816 | 0.030 | 98.449 |
| *Chitinophaga* | 64758 | 0.039 | 93.280 |  | *Francisellaceae* | 50232 | 0.030 | 98.479 |
| *Carnobacterium* | 63788 | 0.038 | 93.318 |  | *Nitrosomonadaceae* | 49377 | 0.029 | 98.508 |
| *Leuconostoc* | 61309 | 0.037 | 93.355 |  | *Syntrophaceae* | 46393 | 0.027 | 98.535 |
| *Nocardia* | 60846 | 0.036 | 93.391 |  | *Piscirickettsiaceae* | 45880 | 0.027 | 98.562 |
| *Rickettsia* | 60465 | 0.036 | 93.427 |  | *Clostridiales_Family_XVIII._Incertae_Sedis* | 45830 | 0.027 | 98.590 |
| *Capnocytophaga* | 59083 | 0.035 | 93.463 |  | *Cellulomonadaceae* | 45558 | 0.027 | 98.616 |
| *Caulobacter* | 58139 | 0.035 | 93.497 |  | *Nitrospiraceae* | 44867 | 0.027 | 98.643 |
| *Amycolatopsis* | 57964 | 0.035 | 93.532 |  | *Heliobacteriaceae* | 42871 | 0.025 | 98.668 |
| *Halanaerobium* | 57631 | 0.034 | 93.566 |  | *Kofleriaceae* | 42633 | 0.025 | 98.694 |
| *Pantoea* | 56936 | 0.034 | 93.600 |  | *Nocardioidaceae* | 42120 | 0.025 | 98.718 |
| *Erwinia* | 56235 | 0.034 | 93.634 |  | *Halobacteroidaceae* | 41757 | 0.025 | 98.743 |
| *Candidatus_Pelagibacter* | 55691 | 0.033 | 93.667 |  | *Candidatus_Midichloriaceae* | 41649 | 0.025 | 98.768 |
| *Pectobacterium* | 54567 | 0.033 | 93.699 |  | *Fibrobacteraceae* | 40590 | 0.024 | 98.792 |
| *Candidatus_Phytoplasma* | 53959 | 0.032 | 93.731 |  | *Hydrogenothermaceae* | 40581 | 0.024 | 98.816 |
| *Bartonella* | 53343 | 0.032 | 93.763 |  | *Clostridiales_Family_XVII._Incertae_Sedis* | 40481 | 0.024 | 98.840 |
| *Stenotrophomonas* | 53335 | 0.032 | 93.795 |  | *Solibacteraceae* | 40125 | 0.024 | 98.863 |
| *Magnetospirillum* | 52139 | 0.031 | 93.826 |  | *Desulfarculaceae* | 39207 | 0.023 | 98.886 |
| *Niastella* | 52131 | 0.031 | 93.857 |  | *Hyphomonadaceae* | 39088 | 0.023 | 98.910 |
| *Thermobacillus* | 52110 | 0.031 | 93.888 |  | *Gordoniaceae* | 39069 | 0.023 | 98.933 |
| *Dyadobacter* | 51732 | 0.031 | 93.919 |  | *Brucellaceae* | 37807 | 0.022 | 98.955 |
| *Yersinia* | 51610 | 0.031 | 93.950 |  | *Dehalococcoidaceae* | 37111 | 0.022 | 98.977 |
| *Thermus* | 51152 | 0.030 | 93.980 |  | *Acidithiobacillaceae* | 36748 | 0.022 | 98.999 |
| *Pelobacter* | 50816 | 0.030 | 94.011 |  | *Oceanospirillaceae* | 35047 | 0.021 | 99.019 |
| *Sphingobium* | 50357 | 0.030 | 94.041 |  | *Catenulisporaceae* | 34921 | 0.021 | 99.040 |
| *Kitasatospora* | 49903 | 0.030 | 94.070 |  | *Streptosporangiaceae* | 34862 | 0.021 | 99.061 |
| *Francisella* | 49840 | 0.030 | 94.100 |  | *Bdellovibrionaceae* | 34485 | 0.020 | 99.081 |
| *Thioalkalivibrio* | 49186 | 0.029 | 94.129 |  | *Legionellaceae* | 34229 | 0.020 | 99.101 |
| *Nostoc* | 49088 | 0.029 | 94.159 |  | *Alcanivoracaceae* | 33851 | 0.020 | 99.121 |
| *Marinobacter* | 49064 | 0.029 | 94.188 |  | *Gallionellaceae* | 33665 | 0.020 | 99.141 |
| *Exiguobacterium* | 48446 | 0.029 | 94.217 |  | *Conexibacteraceae* | 33562 | 0.020 | 99.161 |
| *Neisseria* | 48295 | 0.029 | 94.246 |  | *Hydrogenophilaceae* | 33306 | 0.020 | 99.181 |
| *Hyphomicrobium* | 48281 | 0.029 | 94.274 |  | *Cystobacteraceae* | 32652 | 0.019 | 99.200 |
| *Cellulophaga* | 48211 | 0.029 | 94.303 |  | *Promicromonosporaceae* | 31625 | 0.019 | 99.219 |
| *Sebaldella* | 47914 | 0.029 | 94.332 |  | *Desulfomicrobiaceae* | 31505 | 0.019 | 99.237 |
| *Symbiobacterium* | 45830 | 0.027 | 94.359 |  | *Dictyoglomaceae* | 30663 | 0.018 | 99.255 |
| *Haliscomenobacter* | 45570 | 0.027 | 94.386 |  | *Erythrobacteraceae* | 30196 | 0.018 | 99.273 |
| *Cellulomonas* | 45558 | 0.027 | 94.413 |  | *Beijerinckiaceae* | 28325 | 0.017 | 99.290 |
| *Fretibacterium* | 45349 | 0.027 | 94.440 |  | *Aquificaceae* | 28290 | 0.017 | 99.307 |
| *Azoarcus* | 45293 | 0.027 | 94.467 |  | *Syntrophomonadaceae* | 27732 | 0.016 | 99.323 |
| *Fibrella* | 45271 | 0.027 | 94.494 |  | *Thermomonosporaceae* | 27582 | 0.016 | 99.339 |
| *Polaromonas* | 45181 | 0.027 | 94.521 |  | *Pseudoalteromonadaceae* | 27347 | 0.016 | 99.355 |
| *Leptospira* | 45097 | 0.027 | 94.548 |  | *Elusimicrobiaceae* | 27335 | 0.016 | 99.372 |
| *Thermotoga* | 45089 | 0.027 | 94.575 |  | *Thermodesulfobacteriaceae* | 27247 | 0.016 | 99.388 |
| *Nocardiopsis* | 45063 | 0.027 | 94.602 |  | *Opitutaceae* | 26998 | 0.016 | 99.404 |
| *Sphingomonas* | 44928 | 0.027 | 94.629 |  | *Psychromonadaceae* | 26213 | 0.015 | 99.419 |
| *Solitalea* | 44019 | 0.026 | 94.655 |  | *Flammeovirgaceae* | 26207 | 0.015 | 99.435 |
| *Anabaena* | 43982 | 0.026 | 94.681 |  | *Chlamydiaceae* | 26056 | 0.015 | 99.450 |
| *Cronobacter* | 43810 | 0.026 | 94.707 |  | *Planococcaceae* | 26034 | 0.015 | 99.465 |
| *Delftia* | 42896 | 0.026 | 94.733 |  | *Ignavibacteriaceae* | 25236 | 0.015 | 99.480 |
| *Heliobacterium* | 42871 | 0.026 | 94.758 |  | *Glycomycetaceae* | 25154 | 0.015 | 99.495 |
| *Finegoldia* | 42767 | 0.025 | 94.784 |  | *Nautiliaceae* | 24820 | 0.015 | 99.510 |
| *Haliangium* | 42633 | 0.025 | 94.809 |  | *Acidimicrobiaceae* | 24521 | 0.014 | 99.524 |
| *Paracoccus* | 42392 | 0.025 | 94.835 |  | *Phycisphaeraceae* | 24365 | 0.014 | 99.539 |
| *Runella* | 42373 | 0.025 | 94.860 |  | *Thermoanaerobacterales_Family_IV._Incertae_Sedis* | 23830 | 0.014 | 99.553 |
| *Dehalobacter* | 42115 | 0.025 | 94.885 |  | *Melioribacteraceae* | 23648 | 0.014 | 99.567 |
| *Sphingobacterium* | 41828 | 0.025 | 94.910 |  | *Ferrimonadaceae* | 23477 | 0.014 | 99.581 |
| *Rhodothermus* | 41593 | 0.025 | 94.935 |  | *Chrysiogenaceae* | 23273 | 0.014 | 99.594 |
| *Alteromonas* | 41070 | 0.024 | 94.959 |  | *Kineosporiaceae* | 23121 | 0.014 | 99.608 |
| *Thermosipho* | 40849 | 0.024 | 94.984 |  | *Actinomycetaceae* | 22697 | 0.013 | 99.621 |
| *Flexibacter* | 40767 | 0.024 | 95.008 |  | *Nakamurellaceae* | 22696 | 0.013 | 99.635 |
| *Fibrobacter* | 40590 | 0.024 | 95.032 |  | *Caldilineaceae* | 21983 | 0.013 | 99.648 |
| *Corallococcus* | 40462 | 0.024 | 95.056 |  | *Entomoplasmataceae* | 20047 | 0.012 | 99.660 |
| *Granulicella* | 39641 | 0.024 | 95.080 |  | *Syntrophobacteraceae* | 20025 | 0.012 | 99.672 |
| *Desulfarculus* | 39207 | 0.023 | 95.103 |  | *Sphaerobacteraceae* | 19907 | 0.012 | 99.683 |
| *Coriobacterium* | 39122 | 0.023 | 95.127 |  | *Parachlamydiaceae* | 19853 | 0.012 | 99.695 |
| *Gordonia* | 39069 | 0.023 | 95.150 |  | *Gemmatimonadaceae* | 19565 | 0.012 | 99.707 |
| *Riemerella* | 39001 | 0.023 | 95.173 |  | *Desulfurobacteriaceae* | 19434 | 0.011 | 99.718 |
| *Leptotrichia* | 38573 | 0.023 | 95.196 |  | *Dermabacteraceae* | 18899 | 0.011 | 99.729 |
| *Roseiflexus* | 38447 | 0.023 | 95.219 |  | *Hahellaceae* | 18553 | 0.011 | 99.740 |
| *Pediococcus* | 38383 | 0.023 | 95.242 |  | *Thermodesulfobiaceae* | 18429 | 0.011 | 99.751 |
| *Spirosoma* | 38356 | 0.023 | 95.265 |  | *Rubrobacteraceae* | 18302 | 0.011 | 99.762 |
| *Emticicia* | 38281 | 0.023 | 95.288 |  | *Natranaerobiaceae* | 18274 | 0.011 | 99.773 |
| *Nitrosomonas* | 38037 | 0.023 | 95.310 |  | *Anaerolineaceae* | 17153 | 0.010 | 99.783 |
| *Novosphingobium* | 37294 | 0.022 | 95.332 |  | *Rhodobiaceae* | 17026 | 0.010 | 99.793 |
| *Terriglobus* | 37222 | 0.022 | 95.355 |  | *Colwelliaceae* | 16784 | 0.010 | 99.803 |
| *Pseudoxanthomonas* | 37171 | 0.022 | 95.377 |  | *Coxiellaceae* | 16681 | 0.010 | 99.813 |
| *Chlorobaculum* | 36787 | 0.022 | 95.399 |  | *Beutenbergiaceae* | 16592 | 0.010 | 99.823 |
| *Syntrophobotulus* | 36785 | 0.022 | 95.421 |  | *Desulfurellaceae* | 16218 | 0.010 | 99.832 |
| *Streptobacillus* | 36733 | 0.022 | 95.443 |  | *Methylocystaceae* | 16051 | 0.009 | 99.842 |
| *Anaerococcus* | 36253 | 0.022 | 95.464 |  | *Sanguibacteraceae* | 15483 | 0.009 | 99.851 |
| *Citrobacter* | 36161 | 0.022 | 95.486 |  | *Trueperaceae* | 14910 | 0.009 | 99.860 |
| *Pelodictyon* | 36059 | 0.021 | 95.507 |  | *Dermacoccaceae* | 14014 | 0.008 | 99.868 |
| *Actinobacillus* | 35894 | 0.021 | 95.529 |  | *Tsukamurellaceae* | 13690 | 0.008 | 99.876 |
| *Alicyclobacillus* | 35734 | 0.021 | 95.550 |  | *Desulfohalobiaceae* | 13502 | 0.008 | 99.884 |
| *Marinitoga* | 35135 | 0.021 | 95.571 |  | *Amoebophilaceae* | 13126 | 0.008 | 99.892 |
| *Catenulispora* | 34921 | 0.021 | 95.592 |  | *Bacteriovoracaceae* | 12529 | 0.007 | 99.899 |
| *Streptosporangium* | 34862 | 0.021 | 95.612 |  | *Intrasporangiaceae* | 12434 | 0.007 | 99.906 |
| *Bdellovibrio* | 34485 | 0.021 | 95.633 |  | *Herpetosiphonaceae* | 12132 | 0.007 | 99.914 |
| *Legionella* | 34229 | 0.020 | 95.653 |  | *Chthonomonadaceae* | 12065 | 0.007 | 99.921 |
| *Cyanobacterium* | 34170 | 0.020 | 95.674 |  | *Magnetococcaceae* | 11902 | 0.007 | 99.928 |
| *Simiduia* | 34013 | 0.020 | 95.694 |  | *Halothiobacillaceae* | 11078 | 0.007 | 99.934 |
| *Acidithiobacillus* | 33963 | 0.020 | 95.714 |  | *Puniceicoccaceae* | 10633 | 0.006 | 99.941 |
| *Zobellia* | 33846 | 0.020 | 95.735 |  | *Aerococcaceae* | 10249 | 0.006 | 99.947 |
| *Edwardsiella* | 33798 | 0.020 | 95.755 |  | *Parvularculaceae* | 10204 | 0.006 | 99.953 |
| *Clavibacter* | 33790 | 0.020 | 95.775 |  | *Caldisericaceae* | 10142 | 0.006 | 99.959 |
| *Gloeobacter* | 33769 | 0.020 | 95.795 |  | *Segniliparaceae* | 9745 | 0.006 | 99.964 |
| *Leadbetterella* | 33669 | 0.020 | 95.815 |  | *Cardiobacteriaceae* | 8997 | 0.005 | 99.970 |
| *Conexibacter* | 33562 | 0.020 | 95.835 |  | *Idiomarinaceae* | 8289 | 0.005 | 99.975 |
| *Micavibrio* | 33195 | 0.020 | 95.855 |  | *Thermomicrobiaceae* | 8191 | 0.005 | 99.979 |
| *Zunongwangia* | 33132 | 0.020 | 95.875 |  | *Acidothermaceae* | 7942 | 0.005 | 99.984 |
| *Actinosynnema* | 33126 | 0.020 | 95.894 |  | *Waddliaceae* | 7599 | 0.004 | 99.989 |
| *Rubrivivax* | 33014 | 0.020 | 95.914 |  | *Jonesiaceae* | 7353 | 0.004 | 99.993 |
| *Candidatus_Solibacter* | 32672 | 0.019 | 95.933 |  | *Methylacidiphilaceae* | 7011 | 0.004 | 99.997 |
| *Stigmatella* | 32652 | 0.019 | 95.953 |  | *Simkaniaceae* | 4858 | 0.003 | 100.000 |
| *Echinicola* | 32086 | 0.019 | 95.972 |  |  |  |  |  |
| *Ruegeria* | 31966 | 0.019 | 95.991 |  |  |  |  |  |
| *Chromobacterium* | 31755 | 0.019 | 96.010 |  |  |  |  |  |
| *Rahnella* | 31536 | 0.019 | 96.029 |  |  |  |  |  |
| *Desulfomicrobium* | 31505 | 0.019 | 96.048 |  |  |  |  |  |
| *Tistrella* | 31243 | 0.019 | 96.066 |  |  |  |  |  |
| *Aliivibrio* | 31174 | 0.019 | 96.085 |  |  |  |  |  |
| *Robiginitalea* | 30945 | 0.018 | 96.103 |  |  |  |  |  |
| *Dictyoglomus* | 30663 | 0.018 | 96.122 |  |  |  |  |  |
| *Thermaerobacter* | 30396 | 0.018 | 96.140 |  |  |  |  |  |
| *Saccharothrix* | 30198 | 0.018 | 96.158 |  |  |  |  |  |
| *Sulfurihydrogenibium* | 30122 | 0.018 | 96.176 |  |  |  |  |  |
| *Cytophaga* | 30117 | 0.018 | 96.194 |  |  |  |  |  |
| *Glaciecola* | 29972 | 0.018 | 96.211 |  |  |  |  |  |
| *Ornithobacterium* | 29745 | 0.018 | 96.229 |  |  |  |  |  |
| *Phaeobacter* | 29656 | 0.018 | 96.247 |  |  |  |  |  |
| *Cyclobacterium* | 29618 | 0.018 | 96.265 |  |  |  |  |  |
| *Aggregatibacter* | 29610 | 0.018 | 96.282 |  |  |  |  |  |
| *Ilyobacter* | 29429 | 0.018 | 96.300 |  |  |  |  |  |
| *Erysipelothrix* | 29308 | 0.017 | 96.317 |  |  |  |  |  |
| *Desulfatibacillum* | 29099 | 0.017 | 96.335 |  |  |  |  |  |
| *Micromonospora* | 28929 | 0.017 | 96.352 |  |  |  |  |  |
| *Ehrlichia* | 28635 | 0.017 | 96.369 |  |  |  |  |  |
| *Marinomonas* | 28349 | 0.017 | 96.386 |  |  |  |  |  |
| *Belliella* | 28156 | 0.017 | 96.403 |  |  |  |  |  |
| *Azorhizobium* | 27941 | 0.017 | 96.419 |  |  |  |  |  |
| *Wigglesworthia* | 27936 | 0.017 | 96.436 |  |  |  |  |  |
| *Candidatus_Blochmannia* | 27806 | 0.017 | 96.452 |  |  |  |  |  |
| *Kinetoplastibacterium* | 27799 | 0.017 | 96.469 |  |  |  |  |  |
| *Candidatus_Sulcia* | 27698 | 0.017 | 96.486 |  |  |  |  |  |
| *Meiothermus* | 27604 | 0.016 | 96.502 |  |  |  |  |  |
| *Herbaspirillum* | 27586 | 0.016 | 96.518 |  |  |  |  |  |
| *Thermomonospora* | 27582 | 0.016 | 96.535 |  |  |  |  |  |
| *Rothia* | 27559 | 0.016 | 96.551 |  |  |  |  |  |
| *Dehalococcoides* | 27422 | 0.016 | 96.568 |  |  |  |  |  |
| *Elusimicrobium* | 27335 | 0.016 | 96.584 |  |  |  |  |  |
| *Photorhabdus* | 27296 | 0.016 | 96.600 |  |  |  |  |  |
| *Candidatus_Azobacteroides* | 27205 | 0.016 | 96.616 |  |  |  |  |  |
| *Leptothrix* | 27053 | 0.016 | 96.633 |  |  |  |  |  |
| *Azospira* | 27001 | 0.016 | 96.649 |  |  |  |  |  |
| *Opitutus* | 26998 | 0.016 | 96.665 |  |  |  |  |  |
| *Saccharopolyspora* | 26868 | 0.016 | 96.681 |  |  |  |  |  |
| *Brevibacillus* | 26589 | 0.016 | 96.697 |  |  |  |  |  |
| *Melissococcus* | 26491 | 0.016 | 96.712 |  |  |  |  |  |
| *Alicycliphilus* | 26371 | 0.016 | 96.728 |  |  |  |  |  |
| *Sulfurimonas* | 26357 | 0.016 | 96.744 |  |  |  |  |  |
| *Salinispora* | 26318 | 0.016 | 96.760 |  |  |  |  |  |
| *Oscillatoria* | 26307 | 0.016 | 96.775 |  |  |  |  |  |
| *Marivirga* | 26207 | 0.016 | 96.791 |  |  |  |  |  |
| *Polaribacter* | 26200 | 0.016 | 96.806 |  |  |  |  |  |
| *Alcanivorax* | 26058 | 0.016 | 96.822 |  |  |  |  |  |
| *Fluviicola* | 26038 | 0.016 | 96.838 |  |  |  |  |  |
| *Rivularia* | 25951 | 0.015 | 96.853 |  |  |  |  |  |
| *Tepidanaerobacter* | 25551 | 0.015 | 96.868 |  |  |  |  |  |
| *Solibacillus* | 25492 | 0.015 | 96.883 |  |  |  |  |  |
| *Calothrix* | 25284 | 0.015 | 96.898 |  |  |  |  |  |
| *Gramella* | 25242 | 0.015 | 96.914 |  |  |  |  |  |
| *Ignavibacterium* | 25236 | 0.015 | 96.929 |  |  |  |  |  |
| *Pseudonocardia* | 25234 | 0.015 | 96.944 |  |  |  |  |  |
| *Psychromonas* | 25226 | 0.015 | 96.959 |  |  |  |  |  |
| *Stackebrandtia* | 25154 | 0.015 | 96.974 |  |  |  |  |  |
| *Desulfobulbus* | 25079 | 0.015 | 96.989 |  |  |  |  |  |
| *Thiomonas* | 24996 | 0.015 | 97.004 |  |  |  |  |  |
| *Methylovorus* | 24811 | 0.015 | 97.018 |  |  |  |  |  |
| *Planctomyces* | 24773 | 0.015 | 97.033 |  |  |  |  |  |
| *Xenorhabdus* | 24742 | 0.015 | 97.048 |  |  |  |  |  |
| *Comamonas* | 24740 | 0.015 | 97.063 |  |  |  |  |  |
| *Modestobacter* | 24692 | 0.015 | 97.077 |  |  |  |  |  |
| *Lysinibacillus* | 24650 | 0.015 | 97.092 |  |  |  |  |  |
| *Pseudoalteromonas* | 24462 | 0.015 | 97.107 |  |  |  |  |  |
| *Desulfobacula* | 24454 | 0.015 | 97.121 |  |  |  |  |  |
| *Xanthobacter* | 24428 | 0.015 | 97.136 |  |  |  |  |  |
| *Psychrobacter* | 24372 | 0.015 | 97.150 |  |  |  |  |  |
| *Muricauda* | 24174 | 0.014 | 97.165 |  |  |  |  |  |
| *Candidatus_Liberibacter* | 24137 | 0.014 | 97.179 |  |  |  |  |  |
| *Candidatus_Cloacimonas* | 24072 | 0.014 | 97.193 |  |  |  |  |  |
| *Aequorivita* | 24004 | 0.014 | 97.208 |  |  |  |  |  |
| *Phycisphaera* | 23994 | 0.014 | 97.222 |  |  |  |  |  |
| *Ramlibacter* | 23894 | 0.014 | 97.236 |  |  |  |  |  |
| *Halobacteroides* | 23886 | 0.014 | 97.250 |  |  |  |  |  |
| *Pelotomaculum* | 23841 | 0.014 | 97.265 |  |  |  |  |  |
| *Mahella* | 23830 | 0.014 | 97.279 |  |  |  |  |  |
| *Gluconobacter* | 23789 | 0.014 | 97.293 |  |  |  |  |  |
| *Acholeplasma* | 23772 | 0.014 | 97.307 |  |  |  |  |  |
| *Psychroflexus* | 23760 | 0.014 | 97.321 |  |  |  |  |  |
| *Zymomonas* | 23703 | 0.014 | 97.336 |  |  |  |  |  |
| *Melioribacter* | 23648 | 0.014 | 97.350 |  |  |  |  |  |
| *Deferribacter* | 23523 | 0.014 | 97.364 |  |  |  |  |  |
| *Owenweeksia* | 23500 | 0.014 | 97.378 |  |  |  |  |  |
| *Rhodanobacter* | 23480 | 0.014 | 97.392 |  |  |  |  |  |
| *Shigella* | 23370 | 0.014 | 97.406 |  |  |  |  |  |
| *Roseobacter* | 23282 | 0.014 | 97.419 |  |  |  |  |  |
| *Desulfurispirillum* | 23273 | 0.014 | 97.433 |  |  |  |  |  |
| *Kineococcus* | 23121 | 0.014 | 97.447 |  |  |  |  |  |
| *Verminephrobacter* | 23051 | 0.014 | 97.461 |  |  |  |  |  |
| *Thauera* | 22844 | 0.014 | 97.475 |  |  |  |  |  |
| *Candidatus_Accumulibacter* | 22786 | 0.014 | 97.488 |  |  |  |  |  |
| *Oceanobacillus* | 22775 | 0.014 | 97.502 |  |  |  |  |  |
| *Kribbella* | 22699 | 0.014 | 97.515 |  |  |  |  |  |
| *Nakamurella* | 22696 | 0.014 | 97.529 |  |  |  |  |  |
| *Trichodesmium* | 22669 | 0.014 | 97.542 |  |  |  |  |  |
| *Caldanaerobacter* | 22450 | 0.013 | 97.556 |  |  |  |  |  |
| *Salinibacter* | 22428 | 0.013 | 97.569 |  |  |  |  |  |
| *Chloroflexus* | 22306 | 0.013 | 97.582 |  |  |  |  |  |
| *Lacinutrix* | 22191 | 0.013 | 97.596 |  |  |  |  |  |
| *Candidatus_Hamiltonella* | 22084 | 0.013 | 97.609 |  |  |  |  |  |
| *Phenylobacterium* | 22022 | 0.013 | 97.622 |  |  |  |  |  |
| *Starkeya* | 21985 | 0.013 | 97.635 |  |  |  |  |  |
| *Caldilinea* | 21983 | 0.013 | 97.648 |  |  |  |  |  |
| *Moorella* | 21810 | 0.013 | 97.661 |  |  |  |  |  |
| *Amphibacillus* | 21750 | 0.013 | 97.674 |  |  |  |  |  |
| *Ferrimonas* | 21743 | 0.013 | 97.687 |  |  |  |  |  |
| *Nitrobacter* | 21594 | 0.013 | 97.700 |  |  |  |  |  |
| *Ketogulonicigenium* | 21533 | 0.013 | 97.713 |  |  |  |  |  |
| *Oceanimonas* | 21442 | 0.013 | 97.725 |  |  |  |  |  |
| *Collimonas* | 21439 | 0.013 | 97.738 |  |  |  |  |  |
| *Verrucosispora* | 21385 | 0.013 | 97.751 |  |  |  |  |  |
| *Laribacter* | 21292 | 0.013 | 97.764 |  |  |  |  |  |
| *Atopobium* | 21202 | 0.013 | 97.776 |  |  |  |  |  |
| *Acidobacterium* | 21166 | 0.013 | 97.789 |  |  |  |  |  |
| *Desulfococcus* | 21156 | 0.013 | 97.802 |  |  |  |  |  |
| *Halothermothrix* | 21123 | 0.013 | 97.814 |  |  |  |  |  |
| *Wolbachia* | 21082 | 0.013 | 97.827 |  |  |  |  |  |
| *Acidiphilium* | 21081 | 0.013 | 97.839 |  |  |  |  |  |
| *Thermincola* | 21062 | 0.013 | 97.852 |  |  |  |  |  |
| *Desulfurivibrio* | 21047 | 0.013 | 97.864 |  |  |  |  |  |
| *Sulfurospirillum* | 20837 | 0.012 | 97.877 |  |  |  |  |  |
| *Pandoraea* | 20820 | 0.012 | 97.889 |  |  |  |  |  |
| *Maribacter* | 20808 | 0.012 | 97.902 |  |  |  |  |  |
| *Pseudogulbenkiania* | 20744 | 0.012 | 97.914 |  |  |  |  |  |
| *Nitrosococcus* | 20677 | 0.012 | 97.926 |  |  |  |  |  |
| *Nautilia* | 20661 | 0.012 | 97.939 |  |  |  |  |  |
| *Cyanobium* | 20660 | 0.012 | 97.951 |  |  |  |  |  |
| *Thiocystis* | 20658 | 0.012 | 97.963 |  |  |  |  |  |
| *Fervidobacterium* | 20533 | 0.012 | 97.975 |  |  |  |  |  |
| *Dechloromonas* | 20462 | 0.012 | 97.988 |  |  |  |  |  |
| *Taylorella* | 20452 | 0.012 | 98.000 |  |  |  |  |  |
| *Leisingera* | 20390 | 0.012 | 98.012 |  |  |  |  |  |
| *Tetragenococcus* | 20388 | 0.012 | 98.024 |  |  |  |  |  |
| *Methylophaga* | 20296 | 0.012 | 98.036 |  |  |  |  |  |
| *Desulfobacterium* | 20292 | 0.012 | 98.048 |  |  |  |  |  |
| *Octadecabacter* | 20264 | 0.012 | 98.060 |  |  |  |  |  |
| *Candidatus_Koribacter* | 20228 | 0.012 | 98.073 |  |  |  |  |  |
| *Ureaplasma* | 20148 | 0.012 | 98.085 |  |  |  |  |  |
| *Asticcacaulis* | 20140 | 0.012 | 98.097 |  |  |  |  |  |
| *Mesoplasma* | 20047 | 0.012 | 98.109 |  |  |  |  |  |
| *Nitratifractor* | 20030 | 0.012 | 98.120 |  |  |  |  |  |
| *Syntrophobacter* | 20025 | 0.012 | 98.132 |  |  |  |  |  |
| *Sphaerobacter* | 19907 | 0.012 | 98.144 |  |  |  |  |  |
| *Nonlabens* | 19895 | 0.012 | 98.156 |  |  |  |  |  |
| *Brucella* | 19713 | 0.012 | 98.168 |  |  |  |  |  |
| *Thermacetogenium* | 19627 | 0.012 | 98.180 |  |  |  |  |  |
| *Calditerrivibrio* | 19600 | 0.012 | 98.191 |  |  |  |  |  |
| *Gemmatimonas* | 19565 | 0.012 | 98.203 |  |  |  |  |  |
| *Rhodoferax* | 19519 | 0.012 | 98.215 |  |  |  |  |  |
| *Aromatoleum* | 19508 | 0.012 | 98.226 |  |  |  |  |  |
| *Croceibacter* | 19486 | 0.012 | 98.238 |  |  |  |  |  |
| *Sulfuricurvum* | 19435 | 0.012 | 98.249 |  |  |  |  |  |
| *Allochromatium* | 19418 | 0.012 | 98.261 |  |  |  |  |  |
| *Nocardioides* | 19357 | 0.012 | 98.272 |  |  |  |  |  |
| *Gluconacetobacter* | 19327 | 0.012 | 98.284 |  |  |  |  |  |
| *Azotobacter* | 19114 | 0.011 | 98.295 |  |  |  |  |  |
| *Brachybacterium* | 18899 | 0.011 | 98.307 |  |  |  |  |  |
| *Methylibium* | 18887 | 0.011 | 98.318 |  |  |  |  |  |
| *Pseudovibrio* | 18870 | 0.011 | 98.329 |  |  |  |  |  |
| *Geodermatophilus* | 18824 | 0.011 | 98.340 |  |  |  |  |  |
| *Singulisphaera* | 18750 | 0.011 | 98.352 |  |  |  |  |  |
| *Gardnerella* | 18738 | 0.011 | 98.363 |  |  |  |  |  |
| *Candidatus_Desulforudis* | 18716 | 0.011 | 98.374 |  |  |  |  |  |
| *Ilumatobacter* | 18597 | 0.011 | 98.385 |  |  |  |  |  |
| *Raoultella* | 18572 | 0.011 | 98.396 |  |  |  |  |  |
| *Hahella* | 18553 | 0.011 | 98.407 |  |  |  |  |  |
| *Halobacillus* | 18394 | 0.011 | 98.418 |  |  |  |  |  |
| *Alkalilimnicola* | 18334 | 0.011 | 98.429 |  |  |  |  |  |
| *Rubrobacter* | 18302 | 0.011 | 98.440 |  |  |  |  |  |
| *Natranaerobius* | 18274 | 0.011 | 98.451 |  |  |  |  |  |
| *Lawsonia* | 18246 | 0.011 | 98.462 |  |  |  |  |  |
| *Thermodesulfovibrio* | 18174 | 0.011 | 98.473 |  |  |  |  |  |
| *Thermodesulfobacterium* | 18159 | 0.011 | 98.483 |  |  |  |  |  |
| *Thioflavicoccus* | 18065 | 0.011 | 98.494 |  |  |  |  |  |
| *Krokinobacter* | 18051 | 0.011 | 98.505 |  |  |  |  |  |
| *Stanieria* | 17986 | 0.011 | 98.516 |  |  |  |  |  |
| *Carboxydothermus* | 17958 | 0.011 | 98.526 |  |  |  |  |  |
| *Desulfomonile* | 17919 | 0.011 | 98.537 |  |  |  |  |  |
| *Shimwellia* | 17850 | 0.011 | 98.548 |  |  |  |  |  |
| *Macrococcus* | 17838 | 0.011 | 98.558 |  |  |  |  |  |
| *Oceanithermus* | 17816 | 0.011 | 98.569 |  |  |  |  |  |
| *Thermosediminibacter* | 17711 | 0.011 | 98.579 |  |  |  |  |  |
| *Acetohalobium* | 17710 | 0.011 | 98.590 |  |  |  |  |  |
| *Chamaesiphon* | 17702 | 0.011 | 98.601 |  |  |  |  |  |
| *Sulfurovum* | 17696 | 0.011 | 98.611 |  |  |  |  |  |
| *Denitrovibrio* | 17534 | 0.010 | 98.622 |  |  |  |  |  |
| *Blastococcus* | 17351 | 0.010 | 98.632 |  |  |  |  |  |
| *Brevundimonas* | 17293 | 0.010 | 98.642 |  |  |  |  |  |
| *Leifsonia* | 17288 | 0.010 | 98.653 |  |  |  |  |  |
| *Dinoroseobacter* | 17283 | 0.010 | 98.663 |  |  |  |  |  |
| *Ochrobactrum* | 17208 | 0.010 | 98.673 |  |  |  |  |  |
| *Anaerolinea* | 17153 | 0.010 | 98.683 |  |  |  |  |  |
| *Sphingopyxis* | 17147 | 0.010 | 98.694 |  |  |  |  |  |
| *Methylomicrobium* | 17134 | 0.010 | 98.704 |  |  |  |  |  |
| *Weeksella* | 17042 | 0.010 | 98.714 |  |  |  |  |  |
| *Chromohalobacter* | 17030 | 0.010 | 98.724 |  |  |  |  |  |
| *Chlamydia* | 16998 | 0.010 | 98.734 |  |  |  |  |  |
| *Sideroxydans* | 16993 | 0.010 | 98.744 |  |  |  |  |  |
| *Parvibaculum* | 16912 | 0.010 | 98.754 |  |  |  |  |  |
| *Methylomonas* | 16907 | 0.010 | 98.764 |  |  |  |  |  |
| *Thiobacillus* | 16890 | 0.010 | 98.775 |  |  |  |  |  |
| *Methylococcus* | 16846 | 0.010 | 98.785 |  |  |  |  |  |
| *Halomonas* | 16787 | 0.010 | 98.795 |  |  |  |  |  |
| *Hyphomonas* | 16751 | 0.010 | 98.805 |  |  |  |  |  |
| *Thermobispora* | 16712 | 0.010 | 98.815 |  |  |  |  |  |
| *Syntrophus* | 16616 | 0.010 | 98.824 |  |  |  |  |  |
| *Morganella* | 16536 | 0.010 | 98.834 |  |  |  |  |  |
| *Beutenbergia* | 16509 | 0.010 | 98.844 |  |  |  |  |  |
| *Hippea* | 16218 | 0.010 | 98.854 |  |  |  |  |  |
| *Cellvibrio* | 16178 | 0.010 | 98.863 |  |  |  |  |  |
| *Flexistipes* | 16148 | 0.010 | 98.873 |  |  |  |  |  |
| *Methylotenera* | 16083 | 0.010 | 98.883 |  |  |  |  |  |
| *Chelativorans* | 15964 | 0.010 | 98.892 |  |  |  |  |  |
| *Chroococcidiopsis* | 15906 | 0.009 | 98.902 |  |  |  |  |  |
| *Rhodomicrobium* | 15875 | 0.009 | 98.911 |  |  |  |  |  |
| *Chloroherpeton* | 15858 | 0.009 | 98.921 |  |  |  |  |  |
| *Xylanimonas* | 15831 | 0.009 | 98.930 |  |  |  |  |  |
| *Candidatus_Zinderia* | 15812 | 0.009 | 98.939 |  |  |  |  |  |
| *Geitlerinema* | 15781 | 0.009 | 98.949 |  |  |  |  |  |
| *Spiribacter* | 15722 | 0.009 | 98.958 |  |  |  |  |  |
| *Methylocystis* | 15721 | 0.009 | 98.968 |  |  |  |  |  |
| *Saprospira* | 15689 | 0.009 | 98.977 |  |  |  |  |  |
| *Frateuria* | 15605 | 0.009 | 98.986 |  |  |  |  |  |
| *Proteus* | 15486 | 0.009 | 98.996 |  |  |  |  |  |
| *Sulfuricella* | 15462 | 0.009 | 99.005 |  |  |  |  |  |
| *Microbacterium* | 15451 | 0.009 | 99.014 |  |  |  |  |  |
| *Gallionella* | 15424 | 0.009 | 99.023 |  |  |  |  |  |
| *Methylocella* | 15403 | 0.009 | 99.032 |  |  |  |  |  |
| *Isoptericola* | 15360 | 0.009 | 99.041 |  |  |  |  |  |
| *Anoxybacillus* | 15301 | 0.009 | 99.051 |  |  |  |  |  |
| *Histophilus* | 15258 | 0.009 | 99.060 |  |  |  |  |  |
| *Acetobacter* | 15207 | 0.009 | 99.069 |  |  |  |  |  |
| *Microlunatus* | 15179 | 0.009 | 99.078 |  |  |  |  |  |
| *Syntrophomonas* | 15107 | 0.009 | 99.087 |  |  |  |  |  |
| *Pelagibacterium* | 15014 | 0.009 | 99.096 |  |  |  |  |  |
| *Sanguibacter* | 15011 | 0.009 | 99.105 |  |  |  |  |  |
| *Mobiluncus* | 14929 | 0.009 | 99.114 |  |  |  |  |  |
| *Tolumonas* | 14920 | 0.009 | 99.123 |  |  |  |  |  |
| *Truepera* | 14910 | 0.009 | 99.131 |  |  |  |  |  |
| *Sodalis* | 14768 | 0.009 | 99.140 |  |  |  |  |  |
| *Desulfocapsa* | 14571 | 0.009 | 99.149 |  |  |  |  |  |
| *Desulfotalea* | 14493 | 0.009 | 99.158 |  |  |  |  |  |
| *Pusillimonas* | 14383 | 0.009 | 99.166 |  |  |  |  |  |
| *Petrotoga* | 14323 | 0.009 | 99.175 |  |  |  |  |  |
| *Candidatus_Chloracidobacterium* | 14182 | 0.008 | 99.183 |  |  |  |  |  |
| *Micrococcus* | 14177 | 0.008 | 99.192 |  |  |  |  |  |
| *Herminiimonas* | 14176 | 0.008 | 99.200 |  |  |  |  |  |
| *Kyrpidia* | 14176 | 0.008 | 99.208 |  |  |  |  |  |
| *Jannaschia* | 14126 | 0.008 | 99.217 |  |  |  |  |  |
| *Advenella* | 14109 | 0.008 | 99.225 |  |  |  |  |  |
| *Prosthecochloris* | 14000 | 0.008 | 99.234 |  |  |  |  |  |
| *Kytococcus* | 13985 | 0.008 | 99.242 |  |  |  |  |  |
| *Hydrogenobaculum* | 13971 | 0.008 | 99.250 |  |  |  |  |  |
| *Thermanaerovibrio* | 13902 | 0.008 | 99.259 |  |  |  |  |  |
| *Candidatus_Methylomirabilis* | 13760 | 0.008 | 99.267 |  |  |  |  |  |
| *Microcoleus* | 13757 | 0.008 | 99.275 |  |  |  |  |  |
| *Leptospirillum* | 13732 | 0.008 | 99.283 |  |  |  |  |  |
| *Halorhodospira* | 13702 | 0.008 | 99.291 |  |  |  |  |  |
| *Oligotropha* | 13693 | 0.008 | 99.300 |  |  |  |  |  |
| *Tsukamurella* | 13690 | 0.008 | 99.308 |  |  |  |  |  |
| *Photobacterium* | 13664 | 0.008 | 99.316 |  |  |  |  |  |
| *Weissella* | 13649 | 0.008 | 99.324 |  |  |  |  |  |
| *Desulfohalobium* | 13502 | 0.008 | 99.332 |  |  |  |  |  |
| *Thermodesulfobium* | 13177 | 0.008 | 99.340 |  |  |  |  |  |
| *Erythrobacter* | 13168 | 0.008 | 99.348 |  |  |  |  |  |
| *Isosphaera* | 13141 | 0.008 | 99.356 |  |  |  |  |  |
| *Cryptobacterium* | 13052 | 0.008 | 99.363 |  |  |  |  |  |
| *Gallibacterium* | 12983 | 0.008 | 99.371 |  |  |  |  |  |
| *Nitrospira* | 12958 | 0.008 | 99.379 |  |  |  |  |  |
| *Kocuria* | 12948 | 0.008 | 99.387 |  |  |  |  |  |
| *Oenococcus* | 12842 | 0.008 | 99.394 |  |  |  |  |  |
| *Desulfurobacterium* | 12794 | 0.008 | 99.402 |  |  |  |  |  |
| *Beijerinckia* | 12736 | 0.008 | 99.409 |  |  |  |  |  |
| *Candidatus_Symbiobacter* | 12595 | 0.008 | 99.417 |  |  |  |  |  |
| *Ammonifex* | 12553 | 0.007 | 99.424 |  |  |  |  |  |
| *Bacteriovorax* | 12529 | 0.007 | 99.432 |  |  |  |  |  |
| *Acaryochloris* | 12518 | 0.007 | 99.439 |  |  |  |  |  |
| *Granulibacter* | 12432 | 0.007 | 99.447 |  |  |  |  |  |
| *Maricaulis* | 12372 | 0.007 | 99.454 |  |  |  |  |  |
| *Basfia* | 12340 | 0.007 | 99.461 |  |  |  |  |  |
| *Janthinobacterium* | 12167 | 0.007 | 99.469 |  |  |  |  |  |
| *Intrasporangium* | 12159 | 0.007 | 99.476 |  |  |  |  |  |
| *Herpetosiphon* | 12132 | 0.007 | 99.483 |  |  |  |  |  |
| *Rhodopirellula* | 12090 | 0.007 | 99.490 |  |  |  |  |  |
| *Thermobaculum* | 12075 | 0.007 | 99.498 |  |  |  |  |  |
| *Chthonomonas* | 12065 | 0.007 | 99.505 |  |  |  |  |  |
| *Komagataeibacter* | 11999 | 0.007 | 99.512 |  |  |  |  |  |
| *Syntrophothermus* | 11963 | 0.007 | 99.519 |  |  |  |  |  |
| *Mannheimia* | 11924 | 0.007 | 99.526 |  |  |  |  |  |
| *Crinalium* | 11911 | 0.007 | 99.533 |  |  |  |  |  |
| *Cylindrospermum* | 11909 | 0.007 | 99.540 |  |  |  |  |  |
| *Magnetococcus* | 11902 | 0.007 | 99.547 |  |  |  |  |  |
| *Desulfobacca* | 11836 | 0.007 | 99.555 |  |  |  |  |  |
| *Pasteurella* | 11817 | 0.007 | 99.562 |  |  |  |  |  |
| *Polynucleobacter* | 11748 | 0.007 | 99.569 |  |  |  |  |  |
| *Trichormus* | 11708 | 0.007 | 99.576 |  |  |  |  |  |
| *Thermobifida* | 11634 | 0.007 | 99.582 |  |  |  |  |  |
| *Colwellia* | 11515 | 0.007 | 99.589 |  |  |  |  |  |
| *Teredinibacter* | 11458 | 0.007 | 99.596 |  |  |  |  |  |
| *Pleurocapsa* | 11434 | 0.007 | 99.603 |  |  |  |  |  |
| *Xylella* | 11351 | 0.007 | 99.610 |  |  |  |  |  |
| *Arthrospira* | 11330 | 0.007 | 99.617 |  |  |  |  |  |
| *Candidatus_Profftella* | 11172 | 0.007 | 99.623 |  |  |  |  |  |
| *Marinithermus* | 11119 | 0.007 | 99.630 |  |  |  |  |  |
| *Candidatus_Portiera* | 11086 | 0.007 | 99.636 |  |  |  |  |  |
| *Halothiobacillus* | 11078 | 0.007 | 99.643 |  |  |  |  |  |
| *Saccharophagus* | 11013 | 0.007 | 99.650 |  |  |  |  |  |
| *Candidatus_Protochlamydia* | 10992 | 0.007 | 99.656 |  |  |  |  |  |
| *Microcystis* | 10930 | 0.007 | 99.663 |  |  |  |  |  |
| *Orientia* | 10863 | 0.006 | 99.669 |  |  |  |  |  |
| *Providencia* | 10845 | 0.006 | 99.676 |  |  |  |  |  |
| *Pirellula* | 10759 | 0.006 | 99.682 |  |  |  |  |  |
| *Turneriella* | 10749 | 0.006 | 99.688 |  |  |  |  |  |
| *Methylobacillus* | 10747 | 0.006 | 99.695 |  |  |  |  |  |
| *Coraliomargarita* | 10633 | 0.006 | 99.701 |  |  |  |  |  |
| *Thermosynechococcus* | 10437 | 0.006 | 99.707 |  |  |  |  |  |
| *Persephonella* | 10381 | 0.006 | 99.714 |  |  |  |  |  |
| *Leptolyngbya* | 10267 | 0.006 | 99.720 |  |  |  |  |  |
| *Aerococcus* | 10249 | 0.006 | 99.726 |  |  |  |  |  |
| *Parvularcula* | 10204 | 0.006 | 99.732 |  |  |  |  |  |
| *Coxiella* | 10159 | 0.006 | 99.738 |  |  |  |  |  |
| *Caldisericum* | 10142 | 0.006 | 99.744 |  |  |  |  |  |
| *Gloeocapsa* | 10081 | 0.006 | 99.750 |  |  |  |  |  |
| *Sulfobacillus* | 10079 | 0.006 | 99.756 |  |  |  |  |  |
| *Segniliparus* | 9745 | 0.006 | 99.762 |  |  |  |  |  |
| *Hirschia* | 9732 | 0.006 | 99.768 |  |  |  |  |  |
| *Dehalogenimonas* | 9689 | 0.006 | 99.773 |  |  |  |  |  |
| *Nitrosospira* | 9650 | 0.006 | 99.779 |  |  |  |  |  |
| *Cycloclasticus* | 9561 | 0.006 | 99.785 |  |  |  |  |  |
| *Amycolicicoccus* | 9323 | 0.006 | 99.790 |  |  |  |  |  |
| *Synechocystis* | 9289 | 0.006 | 99.796 |  |  |  |  |  |
| *Nitratiruptor* | 9191 | 0.005 | 99.801 |  |  |  |  |  |
| *Saccharomonospora* | 9165 | 0.005 | 99.807 |  |  |  |  |  |
| *Dichelobacter* | 8997 | 0.005 | 99.812 |  |  |  |  |  |
| *Candidatus_Atelocyanobacterium* | 8956 | 0.005 | 99.818 |  |  |  |  |  |
| *Candidatus_Saccharimonas* | 8933 | 0.005 | 99.823 |  |  |  |  |  |
| *Thermodesulfatator* | 8919 | 0.005 | 99.828 |  |  |  |  |  |
| *Parachlamydia* | 8733 | 0.005 | 99.833 |  |  |  |  |  |
| *Pseudanabaena* | 8663 | 0.005 | 99.839 |  |  |  |  |  |
| *Bibersteinia* | 8573 | 0.005 | 99.844 |  |  |  |  |  |
| *Candidatus_Puniceispirillum* | 8549 | 0.005 | 99.849 |  |  |  |  |  |
| *Kosmotoga* | 8431 | 0.005 | 99.854 |  |  |  |  |  |
| *Mesotoga* | 8218 | 0.005 | 99.859 |  |  |  |  |  |
| *Thermomicrobium* | 8191 | 0.005 | 99.864 |  |  |  |  |  |
| *Aminobacterium* | 7964 | 0.005 | 99.868 |  |  |  |  |  |
| *Acidothermus* | 7942 | 0.005 | 99.873 |  |  |  |  |  |
| *Kangiella* | 7777 | 0.005 | 99.878 |  |  |  |  |  |
| *Thiomicrospira* | 7769 | 0.005 | 99.882 |  |  |  |  |  |
| *Arcanobacterium* | 7720 | 0.005 | 99.887 |  |  |  |  |  |
| *Idiomarina* | 7706 | 0.005 | 99.892 |  |  |  |  |  |
| *Wolinella* | 7699 | 0.005 | 99.896 |  |  |  |  |  |
| *Halothece* | 7690 | 0.005 | 99.901 |  |  |  |  |  |
| *Waddlia* | 7599 | 0.005 | 99.905 |  |  |  |  |  |
| *Chlamydophila* | 7502 | 0.004 | 99.910 |  |  |  |  |  |
| *Anaerobaculum* | 7448 | 0.004 | 99.914 |  |  |  |  |  |
| *Dactylococcopsis* | 7401 | 0.004 | 99.919 |  |  |  |  |  |
| *Candidatus_Amoebophilus* | 7332 | 0.004 | 99.923 |  |  |  |  |  |
| *Methylacidiphilum* | 7011 | 0.004 | 99.927 |  |  |  |  |  |
| *Thermovirga* | 6997 | 0.004 | 99.931 |  |  |  |  |  |
| *Moraxella* | 6948 | 0.004 | 99.935 |  |  |  |  |  |
| *Thalassolituus* | 6693 | 0.004 | 99.939 |  |  |  |  |  |
| *Thioalkalimicrobium* | 6473 | 0.004 | 99.943 |  |  |  |  |  |
| *Candidatus_Nasuia* | 6366 | 0.004 | 99.947 |  |  |  |  |  |
| *Anaplasma* | 6210 | 0.004 | 99.951 |  |  |  |  |  |
| *Candidatus_Midichloria* | 6011 | 0.004 | 99.954 |  |  |  |  |  |
| *Acidimicrobium* | 5922 | 0.004 | 99.958 |  |  |  |  |  |
| *Candidatus_Cardinium* | 5769 | 0.003 | 99.961 |  |  |  |  |  |
| *Renibacterium* | 5708 | 0.003 | 99.965 |  |  |  |  |  |
| *Thermovibrio* | 5701 | 0.003 | 99.968 |  |  |  |  |  |
| *Coprothermobacter* | 5252 | 0.003 | 99.971 |  |  |  |  |  |
| *Hydrogenobacter* | 5180 | 0.003 | 99.974 |  |  |  |  |  |
| *Candidatus_Riesia* | 5169 | 0.003 | 99.977 |  |  |  |  |  |
| *Aquifex* | 5126 | 0.003 | 99.981 |  |  |  |  |  |
| *Candidatus_Endolissoclinum* | 5121 | 0.003 | 99.984 |  |  |  |  |  |
| *Jonesia* | 5113 | 0.003 | 99.987 |  |  |  |  |  |
| *Neorickettsia* | 4995 | 0.003 | 99.990 |  |  |  |  |  |
| *Simkania* | 4858 | 0.003 | 99.992 |  |  |  |  |  |
| *Thermocrinis* | 3804 | 0.002 | 99.995 |  |  |  |  |  |
| *Candidatus_Moranella* | 3231 | 0.002 | 99.997 |  |  |  |  |  |
| *Tropheryma* | 3099 | 0.002 | 99.999 |  |  |  |  |  |
| *Candidatus_Tremblaya* | 2029 | 0.001 | 100.000 |  |  |  |  |  |
| *Candidatus_Hodgkinia* | 444 | 0.000 | 100.000 |  |  |  |  |  |
