# Supplementary material for: Reconstruction of Bacterial and Viral Genomes from Multiple Metagenomes
Source: Front Microbiol. 2016 Apr 12;7:469. doi: 10.3389/fmicb.2016.00469 (PMC4828583; doi:10.3389/fmicb.2016.00469)
Supplement: Supplementary file 4 [file Table4.DOCX]

**Table S4. Total number of reads and their percentage abundance in the identified viral families in all 72 metagenomes.**

| **Viral families** | **# Reads** | **% Abundance** |
| --- | --- | --- |
| *Siphoviridae* | 57569 | 34.775 |
| *Poxviridae* | 19915 | 12.030 |
| *Myoviridae* | 19147 | 11.566 |
| *Herpesviridae* | 12401 | 7.491 |
| *Baculoviridae* | 11528 | 6.963 |
| *Phycodnaviridae* | 7637 | 4.613 |
| *Polydnaviridae* | 4665 | 2.818 |
| *Podoviridae* | 4611 | 2.785 |
| *Iridoviridae* | 3755 | 2.268 |
| *Adenoviridae.seq* | 2541 | 1.535 |
| *Alloherpesviridae* | 1665 | 1.006 |
| *Hytrosaviridae* | 1614 | 0.975 |
| *Ascoviridae* | 1152 | 0.696 |
| *Marseillevirus_family* | 1140 | 0.689 |
| *Coronaviridae* | 861 | 0.520 |
| *Rudiviridae* | 823 | 0.497 |
| *Potyviridae* | 806 | 0.487 |
| *Malacoherpesviridae* | 793 | 0.479 |
| *Lipothrixviridae* | 716 | 0.433 |
| *Nimaviridae* | 697 | 0.421 |
| *Geminiviridae* | 670 | 0.405 |
| *Caulimoviridae* | 665 | 0.402 |
| *Parvoviridae* | 627 | 0.379 |
| *Inoviridae* | 625 | 0.378 |
| *Reoviridae* | 542 | 0.327 |
| *Flaviviridae* | 538 | 0.325 |
| *Closteroviridae* | 525 | 0.317 |
| *Bunyaviridae* | 479 | 0.289 |
| *Retroviridae* | 475 | 0.287 |
| *Papillomaviridae* | 467 | 0.282 |
| *Rhabdoviridae* | 442 | 0.267 |
| *Anelloviridae* | 428 | 0.259 |
| *Bicaudaviridae* | 399 | 0.241 |
| *Asfarviridae* | 389 | 0.235 |
| *Ophioviridae* | 363 | 0.219 |
| *Paramyxoviridae* | 326 | 0.197 |
| *Orthomyxoviridae* | 292 | 0.176 |
| *Microviridae* | 287 | 0.173 |
| *Tymoviridae* | 216 | 0.130 |
| *Secoviridae* | 188 | 0.114 |
| *Narnaviridae* | 169 | 0.102 |
| *Polyomaviridae* | 148 | 0.089 |
| *Tectiviridae* | 139 | 0.084 |
| *Virgaviridae* | 138 | 0.083 |
| *Totiviridae* | 122 | 0.074 |
| *Dicistroviridae* | 118 | 0.071 |
| *Arenaviridae* | 117 | 0.071 |
| *Globuloviridae* | 110 | 0.066 |
| *Betaflexiviridae* | 108 | 0.065 |
| *Astroviridae* | 104 | 0.063 |
| *Fuselloviridae* | 92 | 0.056 |
| *Alphaflexiviridae* | 78 | 0.047 |
| *Hepadnaviridae* | 78 | 0.047 |
| *Caliciviridae* | 76 | 0.046 |
| *Endornaviridae* | 75 | 0.045 |
| *Chrysoviridae* | 68 | 0.041 |
| *Luteoviridae* | 68 | 0.041 |
| *Bromoviridae* | 61 | 0.037 |
| *Hypoviridae* | 60 | 0.036 |
| *Tombusviridae* | 60 | 0.036 |
| *Alphatetraviridae* | 55 | 0.033 |
| *Circoviridae* | 55 | 0.033 |
| *Corticoviridae* | 53 | 0.032 |
| *Picornaviridae* | 50 | 0.030 |
| *Roniviridae* | 46 | 0.028 |
| *Iflaviridae* | 45 | 0.027 |
| *Nanoviridae* | 43 | 0.026 |
| *Togaviridae* | 40 | 0.024 |
| *Birnaviridae* | 35 | 0.021 |
| *Cystoviridae* | 26 | 0.016 |
| *Alvernaviridae* | 20 | 0.012 |
| *Partitiviridae* | 19 | 0.011 |
| *Filoviridae* | 16 | 0.010 |
| *Picobirnaviridae* | 16 | 0.010 |
| *Ampullaviridae* | 15 | 0.009 |
| *Nodaviridae* | 13 | 0.008 |
| *Arteriviridae* | 11 | 0.007 |
| *Plasmaviridae* | 10 | 0.006 |
| *Gammaflexiviridae* | 4 | 0.002 |
| *Leviviridae* | 3 | 0.002 |
| *Marnaviridae* | 3 | 0.002 |
| *Barnaviridae* | 1 | 0.001 |
| *Hepeviridae* | 1 | 0.001 |
| *Megabirnaviridae* | 1 | 0.001 |
