# Supplementary material for: Reconstruction of Bacterial and Viral Genomes from Multiple Metagenomes
Source: Front Microbiol. 2016 Apr 12;7:469. doi: 10.3389/fmicb.2016.00469 (PMC4828583; doi:10.3389/fmicb.2016.00469)
Supplement: Supplementary file 5 [file Table5.DOCX]

**Table S5. Percentage of assembly achieved after alignment of genus-pool reads with 2,576 genomes available at NCBI.** One those genome for which complete genome sequence was available and achieved >95% assembly was selected for the further analysis (shown in bold).

| **GENUS** | **GENOME** | **% Assembly** |
| --- | --- | --- |
| ***Odoribacter*** | **Odoribacter_splanchnicus_DSM_20712_uid63397** | **99.174** |
| ***Bacteroides*** | **Bacteroides_thetaiotaomicron_VPI_5482_uid62913** | **98.887** |
| *Bacteroides* | Bacteroides_fragilis_638R_uid84217 | 98.874 |
| ***Akkermansia*** | **Akkermansia_muciniphila_ATCC_BAA_835_uid58985** | **98.710** |
| ***Parabacteroides*** | **Parabacteroides_distasonis_ATCC_8503_uid58301** | **98.689** |
| *Bacteroides* | Bacteroides_vulgatus_ATCC_8482_uid58253 | 98.508 |
| *Alistipes* | Alistipes_finegoldii_DSM_17242_uid168180 | 98.486 |
| *Bacteroides* | Bacteroides_fragilis_YCH46_uid58195 | 98.045 |
| *Bacteroides* | Bacteroides_fragilis_NCTC_9343_uid57639 | 97.858 |
| ***Roseburia*** | **Roseburia_hominis_A2_183_uid73419** | **97.786** |
| *Streptococcus* | Streptococcus_salivarius_57_I_uid162151 | 97.222 |
| ***Bifidobacterium*** | **Bifidobacterium_longum_JCM_1217_uid62695** | **97.135** |
| *Streptococcus* | Streptococcus_thermophilus_ND03_uid162015 | 96.779 |
| *Bifidobacterium* | Bifidobacterium_longum_NCC2705_uid57939 | 96.647 |
| *Bifidobacterium* | Bifidobacterium_longum_KACC_91563_uid158861 | 96.642 |
| *Bifidobacterium* | Bifidobacterium_longum_F8_uid197184 | 96.594 |
| *Bacteroides* | Bacteroides_xylanisolvens_XB1A_uid197168 | 96.328 |
| *Ruminococcus* | Ruminococcus_bromii_uid197158 | 96.327 |
| *Eubacterium* | Eubacterium_rectale_uid197161 | 96.29 |
| *Streptococcus* | Streptococcus_thermophilus_MN_ZLW_002_uid166827 | 96.209 |
| *Roseburia* | Roseburia_intestinalis_uid197164 | 96.128 |
| *Streptococcus* | Streptococcus_thermophilus_LMD_9_uid58327 | 96.074 |
| *Streptococcus* | Streptococcus_salivarius_CCHSS3_uid70481 | 95.998 |
| *Bifidobacterium* | Bifidobacterium_longum_DJO10A_uid58833 | 95.991 |
| *Ruminococcus* | Ruminococcus_uid197156 | 95.941 |
| *Faecalibacterium* | Faecalibacterium_prausnitzii_uid197157 | 95.814 |
| ***Escherichia*** | **Escherichia_coli_K_12_substr__MDS42_uid193705** | **95.719** |
| *Eubacterium* | Eubacterium_siraeum_uid197160 | 95.58 |
| *Streptococcus* | Streptococcus_thermophilus_LMG_18311_uid58219 | 95.342 |
| *Bifidobacterium* | Bifidobacterium_longum_BBMN68_uid60163 | 95.315 |
| *Streptococcus* | Streptococcus_salivarius_JIM8777_uid162145 | 95.232 |
| ***Eubacterium*** | **Eubacterium_siraeum_V10Sc8a_uid197178** | **95.079** |
| *Alistipes* | Alistipes_shahii_WAL_8301_uid197175 | 95.034 |
| *Streptococcus* | Streptococcus_thermophilus_CNRZ1066_uid58221 | 94.936 |
| *Eubacterium* | Eubacterium_eligens_ATCC_27750_uid59171 | 94.638 |
| *Bifidobacterium* | Bifidobacterium_longum_infantis_157F_uid62693 | 94.606 |
| *Eubacterium* | Eubacterium_rectale_ATCC_33656_uid59169 | 94.60 |
| *Escherichia* | Escherichia_coli_SE15_uid161939 | 94.519 |
| *Roseburia* | Roseburia_intestinalis_XB6B4_uid197179 | 94.487 |
| *Escherichia* | Escherichia_coli_S88_uid62979 | 94.276 |
| *Escherichia* | Escherichia_coli_O83_H1_NRG_857C_uid161987 | 94.091 |
| *Escherichia* | Escherichia_coli_PMV_1_uid219679 | 93.971 |
| *Escherichia* | Escherichia_coli_LF82_uid161965 | 93.943 |
| *Escherichia* | Escherichia_coli_BL21_DE3__uid161947 | 93.924 |
| *Escherichia* | Escherichia_coli_BL21_DE3__uid161949 | 93.924 |
| *Bifidobacterium* | Bifidobacterium_adolescentis_ATCC_15703_uid58559 | 93.868 |
| *Escherichia* | Escherichia_coli__BL21_Gold_DE3_pLysS_AG__uid59245 | 93.795 |
| *Escherichia* | Escherichia_coli_APEC_O78_uid187277 | 93.72 |
| *Escherichia* | Escherichia_coli_B_REL606_uid58803 | 93.718 |
| *Escherichia* | Escherichia_coli_APEC_O1_uid58623 | 93.536 |
| *Escherichia* | Escherichia_coli_BW2952_uid59391 | 93.521 |
| *Escherichia* | Escherichia_coli_DH1_uid161951 | 93.515 |
| *Escherichia* | Escherichia_coli_K_12_substr__MG1655_uid57779 | 93.465 |
| *Escherichia* | Escherichia_coli_DH1_uid162051 | 93.413 |
| *Escherichia* | Escherichia_coli_K_12_substr__W3110_uid161931 | 93.38 |
| *Streptococcus* | Streptococcus_parasanguinis_ATCC_15912_uid49313 | 93.236 |
| *Ruminococcus* | Ruminococcus_champanellensis_18P13_uid197169 | 93.204 |
| *Escherichia* | Escherichia_coli_UM146_uid162043 | 93.195 |
| *Escherichia* | Escherichia_coli_IAI1_uid59377 | 93.193 |
| *Escherichia* | Escherichia_coli_UTI89_uid58541 | 92.94 |
| *Escherichia* | Escherichia_coli_IHE3034_uid162007 | 92.878 |
| *Streptococcus* | Streptococcus_parasanguinis_FW213_uid163997 | 92.704 |
| *Escherichia* | Escherichia_coli_K_12_substr__DH10B_uid58979 | 92.658 |
| *Escherichia* | Escherichia_coli_NA114_uid162139 | 92.498 |
| *Escherichia* | Escherichia_coli__clone_D_i2__uid162047 | 92.393 |
| *Escherichia* | Escherichia_coli__clone_D_i14__uid162049 | 92.393 |
| *Escherichia* | Escherichia_coli_HS_uid58393 | 92.233 |
| *Escherichia* | Escherichia_coli_ATCC_8739_uid58783 | 92.181 |
| *Escherichia* | Escherichia_coli_JJ1886_uid226103 | 92.167 |
| *Eubacterium* | Eubacterium_rectale_uid197162 | 92.089 |
| *Streptococcus* | Streptococcus_thermophilus_JIM_8232_uid162157 | 92.073 |
| *Escherichia* | Escherichia_coli_536_uid58531 | 91.957 |
| *Escherichia* | Escherichia_coli_W_uid162011 | 91.811 |
| *Escherichia* | Escherichia_coli_W_uid162101 | 91.752 |
| *Escherichia* | Escherichia_coli_SMS_3_5_uid58919 | 91.658 |
| *Escherichia* | Escherichia_coli_KO11FL_uid52593 | 91.612 |
| *Escherichia* | Escherichia_coli_UMN026_uid62981 | 91.587 |
| *Escherichia* | Escherichia_coli_ABU_83972_uid161975 | 91.471 |
| *Escherichia* | Escherichia_coli_CFT073_uid57915 | 91.417 |
| *Escherichia* | Escherichia_coli_LY180_uid219461 | 91.398 |
| *Acidaminococcus* | Acidaminococcus_intestini_RyC_MR95_uid74445 | 91.244 |
| *Escherichia* | Escherichia_coli_SE11_uid59425 | 91.178 |
| *Veillonella* | Veillonella_parvula_DSM_2008_uid41927 | 91.177 |
| *Haemophilus* | Haemophilus_parainfluenzae_T3T1_uid72801 | 91.141 |
| *Escherichia* | Escherichia_coli_042_uid161985 | 90.44 |
| *Klebsiella* | Klebsiella_pneumoniae_CG43_uid223021 | 90.375 |
| *Coprococcus* | Coprococcus_ART55_1_uid197176 | 90.363 |
| *Escherichia* | Escherichia_coli_E24377A_uid58395 | 90.236 |
| *Escherichia* | Escherichia_coli_O7_K1_CE10_uid162115 | 90.228 |
| *Coprococcus* | Coprococcus_catus_GD_7_uid197174 | 90.21 |
| *Klebsiella* | Klebsiella_pneumoniae_KCTC_2242_uid162147 | 89.85 |
| *Escherichia* | Escherichia_coli_IAI39_uid59381 | 89.477 |
| *Klebsiella* | Klebsiella_pneumoniae_MGH_78578_uid57619 | 89.432 |
| *Escherichia* | Escherichia_coli_P12b_uid162061 | 88.964 |
| *Escherichia* | Escherichia_coli_55989_uid59383 | 88.919 |
| *Klebsiella* | Klebsiella_pneumoniae_JM45_uid215235 | 88.918 |
| *Escherichia* | Escherichia_coli_ETEC_H10407_uid161993 | 88.514 |
| *Klebsiella* | Klebsiella_pneumoniae_NTUH_K2044_uid59073 | 88.261 |
| *Escherichia* | Escherichia_coli_UMNK88_uid161991 | 88.245 |
| *Escherichia* | Escherichia_coli_O104_H4_2009EL_2050_uid175905 | 88.143 |
| *Lactobacillus* | Lactobacillus_ruminis_ATCC_27782_uid73417 | 88.101 |
| *Klebsiella* | Klebsiella_pneumoniae_HS11286_uid84387 | 87.861 |
| *Escherichia* | Escherichia_coli_O104_H4_2011C_3493_uid176127 | 87.813 |
| *Escherichia* | Escherichia_coli_O104_H4_2009EL_2071_uid176128 | 87.678 |
| *Escherichia* | Escherichia_coli_KO11FL_uid162099 | 87.676 |
| *Escherichia* | Escherichia_coli_O127_H6_E2348_69_uid59343 | 87.373 |
| *Escherichia* | Escherichia_coli_ED1a_uid59379 | 87.274 |
| *Escherichia* | Escherichia_coli_O55_H7_RM12579_uid162153 | 86.429 |
| *Megamonas* | Megamonas_hypermegale_uid197163 | 86.307 |
| *Bacteroides* | Bacteroides_salanitronis_DSM_18170_uid63269 | 86.236 |
| *Escherichia* | Escherichia_coli_O55_H7_CB9615_uid46655 | 86.138 |
| *Klebsiella* | Klebsiella_pneumoniae_1084_uid174151 | 86.079 |
| *Megasphaera* | Megasphaera_elsdenii_DSM_20460_uid71135 | 85.863 |
| *Bifidobacterium* | Bifidobacterium_longum_JDM301_uid49131 | 85.023 |
| *Escherichia* | Escherichia_coli_O103_H2_12009_uid41013 | 84.939 |
| *Escherichia* | Escherichia_coli_O111_H__11128_uid41023 | 84.42 |
| *Escherichia* | Escherichia_coli_Xuzhou21_uid163995 | 84.332 |
| *Acidaminococcus* | Acidaminococcus_fermentans_DSM_20731_uid43471 | 83.644 |
| *Klebsiella* | Klebsiella_pneumoniae_rhinoscleromatis_SB3432_uid203334 | 83.348 |
| *Escherichia* | Escherichia_coli_O26_H11_11368_uid41021 | 83.339 |
| *Escherichia* | Escherichia_coli_O157_H7_uid57781 | 83.25 |
| *Escherichia* | Escherichia_coli_O157_H7_TW14359_uid59235 | 83.125 |
| *Escherichia* | Escherichia_coli_O157_H7_EDL933_uid57831 | 82.807 |
| *Escherichia* | Escherichia_coli_O157_H7_EC4115_uid59091 | 82.563 |
| *Bacteroides* | Bacteroides_uniformis_uid13130 | 80.966 |
| *Klebsiella* | Klebsiella_variicola_At_22_uid42113 | 80.862 |
| *Klebsiella* | Klebsiella_pneumoniae_342_uid59145 | 79.206 |
| *Streptococcus* | Streptococcus_I_G2_uid224251 | 78.389 |
| *Streptococcus* | Streptococcus_I_P16_uid224252 | 76.449 |
| *Clostridium* | Clostridium_cf__saccharolyticum_K10_uid197201 | 76.44 |
| *Adlercreutzia* | Adlercreutzia_equolifaciens_DSM_19450_uid223286 | 75.448 |
| *Bifidobacterium* | Bifidobacterium_longum_infantis_ATCC_15697_uid159865 | 71.073 |
| *Bifidobacterium* | Bifidobacterium_longum_infantis_ATCC_15697_uid58677 | 70.974 |
| *Lactobacillus* | Lactobacillus_salivarius_UCC118_uid58233 | 68.405 |
| *Lactobacillus* | Lactobacillus_salivarius_CECT_5713_uid162005 | 68.315 |
| *Streptococcus* | Streptococcus_anginosus_C1051_uid218003 | 65.095 |
| *Escherichia* | Escherichia_fergusonii_ATCC_35469_uid59375 | 63.99 |
| *Gordonibacter* | Gordonibacter_pamelaeae_7_10_1_b_uid197167 | 63.033 |
| *Streptococcus* | Streptococcus_gordonii_Challis_substr__CH1_uid57667 | 61.114 |
| *Streptococcus* | Streptococcus_sanguinis_SK36_uid58381 | 58.531 |
| *Streptococcus* | Streptococcus_mitis_B6_uid46097 | 58.239 |
| *Streptococcus* | Streptococcus_oralis_Uo5_uid65449 | 57.033 |
| *Streptococcus* | Streptococcus_anginosus_C238_uid218004 | 56.976 |
| *Eggerthella* | Eggerthella_lenta_DSM_2243_uid59079 | 56.961 |
| *Bifidobacterium* | Bifidobacterium_bifidum_BGN4_uid167988 | 56.872 |
| *Streptococcus* | Streptococcus_pneumoniae_R6_uid57859 | 56.796 |
| *Streptococcus* | Streptococcus_pneumoniae_D39_uid58581 | 56.641 |
| *Streptococcus* | Streptococcus_pneumoniae_SPN034156_uid197185 | 56.412 |
| *Streptococcus* | Streptococcus_pneumoniae_G54_uid59167 | 56.343 |
| *Streptococcus* | Streptococcus_pneumoniae_SPN994038_uid197187 | 56.336 |
| *Streptococcus* | Streptococcus_pneumoniae_SPN994039_uid197188 | 56.329 |
| *Bifidobacterium* | Bifidobacterium_bifidum_PRL2010_uid59883 | 56.327 |
| *Streptococcus* | Streptococcus_pneumoniae_OXC141_uid162037 | 56.171 |
| *Streptococcus* | Streptococcus_pneumoniae_SPN034183_uid197186 | 56.131 |
| *Streptococcus* | Streptococcus_pneumoniae_gamPNI0373_uid175861 | 55.825 |
| *Streptococcus* | Streptococcus_pneumoniae_TCH8431_19A_uid49735 | 55.771 |
| *Streptococcus* | Streptococcus_pneumoniae_A026_uid226114 | 55.751 |
| *Streptococcus* | Streptococcus_pneumoniae_P1031_uid59123 | 55.481 |
| *Bifidobacterium* | Bifidobacterium_bifidum_S17_uid59545 | 55.405 |
| *Streptococcus* | Streptococcus_pneumoniae_INV200_uid162035 | 55.338 |
| *Streptococcus* | Streptococcus_pneumoniae_Taiwan19F_14_uid59119 | 55.323 |
| *Bifidobacterium* | Bifidobacterium_breve_ACS_071_V_Sch8b_uid158863 | 55.246 |
| *Streptococcus* | Streptococcus_pneumoniae_JJA_uid59121 | 55.009 |
| *Bifidobacterium* | Bifidobacterium_breve_UCC2003_uid193702 | 54.914 |
| *Streptococcus* | Streptococcus_pneumoniae_SPN032672_uid197189 | 54.521 |
| *Streptococcus* | Streptococcus_pneumoniae_SPN033038_uid197190 | 54.477 |
| *Streptococcus* | Streptococcus_pneumoniae_INV104_uid162039 | 54.459 |
| *Streptococcus* | Streptococcus_pneumoniae_ST556_uid162191 | 54.443 |
| *Streptococcus* | Streptococcus_pneumoniae_AP200_uid52453 | 54.395 |
| *Streptococcus* | Streptococcus_pneumoniae_SPNA45_uid174986 | 54.077 |
| *Streptococcus* | Streptococcus_pneumoniae_CGSP14_uid59181 | 53.951 |
| *Streptococcus* | Streptococcus_pneumoniae_TIGR4_uid57857 | 53.893 |
| *Streptococcus* | Streptococcus_pneumoniae_ATCC_700669_uid59287 | 53.737 |
| *Streptococcus* | Streptococcus_pneumoniae_70585_uid59125 | 53.632 |
| *Streptococcus* | Streptococcus_pneumoniae_670_6B_uid52533 | 53.398 |
| *Streptococcus* | Streptococcus_pneumoniae_Hungary19A_6_uid59117 | 52.759 |
| *Streptococcus* | Streptococcus_pseudopneumoniae_IS7493_uid71153 | 52.5 |
| *Pediococcus* | Pediococcus_pentosaceus_SL4_uid227215 | 51.488 |
| *Streptococcus* | Streptococcus_constellatus_pharyngis_C1050_uid218002 | 51.268 |
| *Streptococcus* | Streptococcus_constellatus_pharyngis_C818_uid218001 | 50.715 |
| *Streptococcus* | Streptococcus_constellatus_pharyngis_C232_uid217998 | 50.711 |
| *Pediococcus* | Pediococcus_pentosaceus_ATCC_25745_uid57981 | 49.756 |
| *Streptococcus* | Streptococcus_intermedius_C270_uid217999 | 47.136 |
| *Streptococcus* | Streptococcus_intermedius_B196_uid218000 | 46.293 |
| *Streptococcus* | Streptococcus_intermedius_JTH08_uid168614 | 45.159 |
| *Bifidobacterium* | Bifidobacterium_dentium_Bd1_uid43091 | 44.009 |
| *Lactococcus* | Lactococcus_lactis_CV56_uid160253 | 43.226 |
| *Lactococcus* | Lactococcus_lactis_Il1403_uid57671 | 43.126 |
| *Enterobacter* | Enterobacter_cloacae_EcWSU1_uid80739 | 43.027 |
| *Lactococcus* | Lactococcus_lactis_KLDS_4_0325_uid225028 | 41.808 |
| *Klebsiella* | Klebsiella_oxytoca_KCTC_1686_uid83159 | 41.546 |
| *Klebsiella* | Klebsiella_oxytoca_E718_uid170256 | 41.446 |
| *Lactococcus* | Lactococcus_lactis_IO_1_uid192185 | 40.436 |
| *Enterobacter* | Enterobacter_cloacae_ENHKU01_uid172463 | 39.559 |
| *Lactococcus* | Lactococcus_lactis_cremoris_UC509_9_uid179384 | 38.995 |
| *Bacteroides* | Bacteroides_helcogenes_P_36_108_uid62135 | 38.488 |
| *Lactococcus* | Lactococcus_lactis_KF147_uid42831 | 38.432 |
| *Lactococcus* | Lactococcus_lactis_cremoris_A76_uid160937 | 37.995 |
| *Lactococcus* | Lactococcus_lactis_cremoris_SK11_uid57983 | 37.639 |
| *Enterobacter* | Enterobacter_cloacae_NCTC_9394_uid197202 | 37.535 |
| *Lactococcus* | Lactococcus_lactis_cremoris_MG1363_uid58837 | 37.134 |
| *Lactococcus* | Lactococcus_lactis_cremoris_NZ9000_uid167481 | 37.091 |
| *Streptococcus* | Streptococcus_oligofermentans_AS_1_3089_uid201429 | 36.59 |
| *Lactococcus* | Lactococcus_lactis_cremoris_KW2_uid219629 | 35.358 |
| *Enterobacter* | Enterobacter_cloacae_dissolvens_SDM_uid168997 | 34.566 |
| *Haemophilus* | Haemophilus_influenzae_KR494_uid219323 | 34.122 |
| *Haemophilus* | Haemophilus_influenzae_PittEE_uid58591 | 34.021 |
| *Haemophilus* | Haemophilus_influenzae_R2846_uid161921 | 33.872 |
| *Haemophilus* | Haemophilus_influenzae_R2866_uid161923 | 33.714 |
| *Haemophilus* | Haemophilus_influenzae_PittGG_uid58593 | 33.46 |
| *Haemophilus* | Haemophilus_influenzae_Rd_KW20_uid57771 | 33.427 |
| *Haemophilus* | Haemophilus_influenzae_86_028NP_uid58093 | 33.146 |
| *Haemophilus* | Haemophilus_influenzae_10810_uid86647 | 32.568 |
| *Rothia* | Rothia_mucilaginosa_uid43093 | 32.451 |
| *Enterobacter* | Enterobacter_cloacae_ATCC_13047_uid48363 | 32.164 |
| *Haemophilus* | Haemophilus_influenzae_F3031_uid62123 | 30.618 |
| *Lactobacillus* | Lactobacillus_reuteri_I5007_uid208677 | 30.281 |
| *Haemophilus* | Haemophilus_influenzae_F3047_uid62097 | 30.133 |
| *Enterobacter* | Enterobacter_asburiae_LF7a_uid72793 | 28.912 |
| *Lactobacillus* | Lactobacillus_reuteri_DSM_20016_uid58471 | 26.451 |
| *Lactobacillus* | Lactobacillus_reuteri_JCM_1112_uid58875 | 26.385 |
| *Lactobacillus* | Lactobacillus_reuteri_TD1_uid213089 | 24.649 |
| *Lactobacillus* | Lactobacillus_reuteri_SD2112_uid55357 | 23.752 |
| *Enterobacter* | Enterobacter_638_uid58727 | 21.471 |
| *Ruminococcus* | _Ruminococcus__obeum_uid197165 | 20.596 |
| *Streptococcus* | Streptococcus_infantarius_CJ18_uid87033 | 20.528 |
| *Bifidobacterium* | Bifidobacterium_animalis_lactis_ATCC_27673_uid222803 | 20.521 |
| *Streptococcus* | Streptococcus_macedonicus_ACA_DC_198_uid81631 | 20.484 |
| *Porphyromonas* | Porphyromonas_asaccharolytica_DSM_20707_uid66603 | 19.993 |
| *Bifidobacterium* | Bifidobacterium_thermophilum_RBL67_uid193770 | 19.774 |
| *Bifidobacterium* | Bifidobacterium_animalis_lactis_AD011_uid58911 | 19.617 |
| *Bifidobacterium* | Bifidobacterium_animalis_lactis_DSM_10140_uid59357 | 19.506 |
| *Bifidobacterium* | Bifidobacterium_animalis_lactis_Bl_04_uid59359 | 19.488 |
| *Bifidobacterium* | Bifidobacterium_animalis_lactis_Bi_07_uid163693 | 19.487 |
| *Bifidobacterium* | Bifidobacterium_animalis_lactis_BB_12_uid158871 | 19.479 |
| *Bifidobacterium* | Bifidobacterium_animalis_lactis_CNCM_I_2494_uid158869 | 19.475 |
| *Bifidobacterium* | Bifidobacterium_animalis_lactis_B420_uid163691 | 19.473 |
| *Bifidobacterium* | Bifidobacterium_animalis_lactis_BLC1_uid158867 | 19.469 |
| *Bifidobacterium* | Bifidobacterium_animalis_lactis_Bl12_uid210081 | 19.463 |
| *Bifidobacterium* | Bifidobacterium_animalis_lactis_V9_uid158865 | 19.459 |
| *Bifidobacterium* | Bifidobacterium_animalis_ATCC_25527_uid162513 | 19.26 |
| *Lactobacillus* | Lactobacillus_delbrueckii_bulgaricus_2038_uid161929 | 19.236 |
| *Lactobacillus* | Lactobacillus_delbrueckii_bulgaricus_ATCC_11842_uid58647 | 19.193 |
| *Lactobacillus* | Lactobacillus_delbrueckii_bulgaricus_ATCC_BAA_365_uid57987 | 19.033 |
| *Streptococcus* | Streptococcus_lutetiensis_033_uid213397 | 18.43 |
| *Prevotella* | Prevotella_dentalis_DSM_3688_uid184818 | 18.269 |
| *Lactobacillus* | Lactobacillus_sakei_23K_uid58281 | 16.997 |
| *Streptococcus* | Streptococcus_pasteurianus_ATCC_43144_uid68019 | 16.906 |
| *Lactobacillus* | Lactobacillus_delbrueckii_bulgaricus_ND02_uid60621 | 16.487 |
| *Streptococcus* | Streptococcus_mutans_UA159_uid57947 | 16.456 |
| *Streptococcus* | Streptococcus_mutans_LJ23_uid162197 | 16.415 |
| *Streptococcus* | Streptococcus_mutans_NN2025_uid46353 | 16.162 |
| *Streptococcus* | Streptococcus_mutans_GS_5_uid169223 | 16.095 |
| *Oscillibacter* | Oscillibacter_valericigenes_Sjm18_20_uid73895 | 16.04 |
| *Prevotella* | Prevotella_denticola_F0289_uid65091 | 15.714 |
| *Streptococcus* | Streptococcus_pyogenes_MGAS2096_uid58573 | 15.661 |
| *Enterobacter* | Enterobacter_cloacae_SCF1_uid59969 | 15.527 |
| *Atopobium* | Atopobium_parvulum_DSM_20469_uid59195 | 15.463 |
| *Fusobacterium* | Fusobacterium_4_8_uid205051 | 15.406 |
| *Streptococcus* | Streptococcus_gallolyticus_ATCC_43143_uid162103 | 15.156 |
| *Streptococcus* | Streptococcus_gallolyticus_ATCC_BAA_2069_uid63617 | 15.126 |
| *Desulfovibrio* | Desulfovibrio_desulfuricans_ATCC_27774_uid59213 | 15.08 |
| *Eggerthella* | Eggerthella_YY7918_uid68707 | 15.018 |
| *Streptococcus* | Streptococcus_suis_GZ1_uid161937 | 14.902 |
| *Fusobacterium* | Fusobacterium_nucleatum_ATCC_25586_uid57885 | 14.893 |
| *Streptococcus* | Streptococcus_gallolyticus_UCN34_uid46061 | 14.843 |
| *Fusobacterium* | Fusobacterium_3_1_36A2_uid55995 | 14.765 |
| *Enterobacter* | Enterobacteriaceae_bacterium_FGI_57_uid185181 | 14.668 |
| *Streptococcus* | Streptococcus_suis_ST1_uid167482 | 14.294 |
| *Prevotella* | Prevotella_oral_taxon_299_F0039_uid45899 | 14.204 |
| *Lactobacillus* | Lactobacillus_fermentum_IFO_3956_uid58865 | 14.1 |
| *Enterococcus* | Enterococcus_faecalis_OG1RF_uid54927 | 14.094 |
| *Lactobacillus* | Lactobacillus_fermentum_F6_uid203391 | 14.068 |
| *Lactobacillus* | Lactobacillus_fermentum_CECT_5716_uid162003 | 14.02 |
| *Streptococcus* | Streptococcus_suis_BM407_uid59321 | 13.957 |
| *Escherichia* | Escherichia_blattae_DSM_4481_uid165043 | 13.919 |
| *Streptococcus* | Streptococcus_suis_JS14_uid162095 | 13.913 |
| *Streptococcus* | Streptococcus_suis_SS12_uid162123 | 13.862 |
| *Streptococcus* | Streptococcus_suis_SC070731_uid193769 | 13.855 |
| *Enterococcus* | Enterococcus_faecalis_Symbioflor_1_uid183342 | 13.843 |
| *Streptococcus* | Streptococcus_suis_D9_uid162125 | 13.741 |
| *Streptococcus* | Streptococcus_suis_YB51_uid222230 | 13.721 |
| *Streptococcus* | Streptococcus_suis_TL13_uid203123 | 13.703 |
| *Streptococcus* | Streptococcus_suis_ST3_uid66327 | 13.695 |
| *Streptococcus* | Streptococcus_suis_SC84_uid59323 | 13.646 |
| *Streptococcus* | Streptococcus_suis_98HAH33_uid58665 | 13.532 |
| *Streptococcus* | Streptococcus_suis_05ZYH33_uid58663 | 13.500 |
| *Enterobacter* | Enterobacter_aerogenes_KCTC_2190_uid68103 | 13.439 |
| *Enterococcus* | Enterococcus_faecalis_D32_uid171261 | 13.404 |
| *Streptococcus* | Streptococcus_suis_D12_uid162127 | 13.374 |
| *Streptococcus* | Streptococcus_suis_S735_uid174333 | 13.363 |
| *Enterobacter* | Enterobacter_aerogenes_EA1509E_uid187411 | 13.317 |
| *Streptococcus* | Streptococcus_suis_P1_7_uid32235 | 13.283 |
| *Streptococcus* | Streptococcus_dysgalactiae_equisimilis_RE378_uid176684 | 13.266 |
| *Enterococcus* | Enterococcus_faecalis_62_uid159663 | 13.217 |
| *Streptococcus* | Streptococcus_suis_A7_uid162111 | 13.145 |
| *Enterobacter* | Enterobacter_R4_368_uid208672 | 12.962 |
| *Streptococcus* | Streptococcus_pyogenes_MGAS9429_uid58569 | 12.817 |
| *Enterococcus* | Enterococcus_faecalis_V583_uid57669 | 12.811 |
| *Streptococcus* | Streptococcus_pyogenes_MGAS10750_uid58575 | 12.739 |
| *Streptococcus* | Streptococcus_pyogenes_MGAS15252_uid158037 | 12.727 |
| *Streptococcus* | Streptococcus_pyogenes_A20_uid178106 | 12.681 |
| *Streptococcus* | Streptococcus_pyogenes_MGAS5005_uid58337 | 12.668 |
| *Streptococcus* | Streptococcus_pyogenes_MGAS6180_uid58335 | 12.651 |
| *Streptococcus* | Streptococcus_pyogenes_MGAS10394_uid58105 | 12.645 |
| *Clostridium* | Clostridium_perfringens_SM101_uid58117 | 12.625 |
| *Enterococcus* | Enterococcus_7L76_uid197170 | 12.623 |
| *Streptococcus* | Streptococcus_pyogenes_HSC5_uid212978 | 12.599 |
| *Streptococcus* | Streptococcus_pyogenes_Alab49_uid162171 | 12.539 |
| *Prevotella* | Prevotella_melaninogenica_ATCC_25845_uid51377 | 12.53 |
| *Streptococcus* | Streptococcus_dysgalactiae_equisimilis_ATCC_12394_uid161979 | 12.524 |
| *Streptococcus* | Streptococcus_pyogenes_M1_GAS_uid57845 | 12.52 |
| *Streptococcus* | Streptococcus_pyogenes_MGAS1882_uid158061 | 12.51 |
| *Streptococcus* | Streptococcus_pyogenes_NZ131_uid59035 | 12.476 |
| *Streptococcus* | Streptococcus_pyogenes_M1_476_uid193766 | 12.408 |
| *Streptococcus* | Streptococcus_agalactiae_2603V_R_uid57943 | 12.386 |
| *Streptococcus* | Streptococcus_pyogenes_Manfredo_uid57847 | 12.347 |
| *Streptococcus* | Streptococcus_suis_T15_uid226112 | 12.336 |
| *Prevotella* | Prevotella_intermedia_17_uid163151 | 12.323 |
| *Streptococcus* | Streptococcus_agalactiae_SA20_06_uid178722 | 12.184 |
| *Streptococcus* | Streptococcus_pyogenes_MGAS10270_uid58571 | 12.149 |
| *Streptococcus* | Streptococcus_agalactiae_ILRI112_uid208675 | 12.115 |
| *Streptococcus* | Streptococcus_agalactiae_09mas018883_uid208674 | 12.098 |
| *Streptococcus* | Streptococcus_dysgalactiae_equisimilis_AC_2713_uid178644 | 12.056 |
| *Clostridium* | Clostridium_perfringens_13_uid57681 | 12.05 |
| *Streptococcus* | Streptococcus_pyogenes_MGAS315_uid57911 | 12.046 |
| *Streptococcus* | Streptococcus_pyogenes_MGAS8232_uid57871 | 12.03 |
| *Streptococcus* | Streptococcus_pyogenes_SSI_1_uid57895 | 11.788 |
| *Streptococcus* | Streptococcus_agalactiae_2_22_uid202215 | 11.696 |
| *Streptococcus* | Streptococcus_agalactiae_GD201008_001_uid175780 | 11.57 |
| *Ruminococcus* | _Ruminococcus__torques_uid197166 | 11.551 |
| *Streptococcus* | Streptococcus_dysgalactiae_equisimilis_GGS_124_uid59103 | 11.537 |
| *Streptococcus* | Streptococcus_dysgalactiae_equisimilis_167_uid222822 | 11.506 |
| *Streptococcus* | Streptococcus_agalactiae_A909_uid57935 | 11.389 |
| *Streptococcus* | Streptococcus_agalactiae_NEM316_uid61585 | 11.268 |
| *Clostridium* | Clostridium_SY8519_uid68705 | 11.262 |
| *Clostridium* | Clostridium_botulinum_E3_Alaska_E43_uid59157 | 11.23 |
| *Streptococcus* | Streptococcus_equi_zooepidemicus_uid59261 | 11.228 |
| *Streptococcus* | Streptococcus_agalactiae_ILRI005_uid208676 | 11.211 |
| *Clostridium* | Clostridium_perfringens_ATCC_13124_uid57901 | 11.156 |
| *Lactobacillus* | Lactobacillus_sanfranciscensis_TMW_1_1304_uid72937 | 11.092 |
| *Aggregatibacter* | Aggregatibacter_aphrophilus_NJ8700_uid59407 | 11.027 |
| *Clostridium* | Clostridium_botulinum_B_Eklund_17B_uid59159 | 10.843 |
| *Streptococcus* | Streptococcus_uberis_0140J_uid57959 | 10.81 |
| *Prevotella* | Prevotella_ruminicola_23_uid47507 | 10.768 |
| *Thermus* | Thermus_scotoductus_SA_01_uid62273 | 10.643 |
| *Olsenella* | Olsenella_uli_DSM_7084_uid51367 | 10.608 |
| *Clostridium* | Clostridium_saccharolyticum_WM1_uid51419 | 10.548 |
| *Streptococcus* | Streptococcus_iniae_SF1_uid206041 | 10.493 |
| *Haemophilus* | Haemophilus_somnus_129PT_uid57929 | 10.433 |
| *Streptococcus* | Streptococcus_equi_zooepidemicus_ATCC_35246_uid162155 | 10.169 |
| *Streptococcus* | Streptococcus_parauberis_KCTC_11537_uid67355 | 10.098 |
| *Bifidobacterium* | Bifidobacterium_asteroides_PRL2011_uid176921 | 9.529 |
| *Streptococcus* | Streptococcus_equi_4047_uid59259 | 9.421 |
| *Haemophilus* | Haemophilus_parasuis_SH0165_uid59273 | 9.346 |
| *Haemophilus* | Haemophilus_somnus_2336_uid57979 | 9.229 |
| *Haemophilus* | Haemophilus_parasuis_ZJ0906_uid209117 | 9.058 |
| *Haemophilus* | Haemophilus_ducreyi_35000HP_uid57625 | 8.813 |
| *Streptococcus* | Streptococcus_equi_zooepidemicus_MGCS10565_uid59263 | 8.671 |
| *Shigella* | Shigella_flexneri_2a_2457T_uid57991 | 8.505 |
| *Shigella* | Shigella_flexneri_5_8401_uid58583 | 8.481 |
| *Shigella* | Shigella_flexneri_2002017_uid159233 | 8.438 |
| *Shigella* | Shigella_sonnei_Ss046_uid58217 | 8.438 |
| *Shigella* | Shigella_boydii_Sb227_uid58215 | 8.419 |
| *Shigella* | Shigella_flexneri_2a_301_uid62907 | 8.339 |
| *Shigella* | Shigella_sonnei_53G_uid84383 | 8.283 |
| *Clostridium* | Clostridium_saccharobutylicum_DSM_13864_uid223284 | 8.218 |
| *Selenomonas* | Selenomonas_sputigena_ATCC_35185_uid55329 | 8.099 |
| *Shigella* | Shigella_dysenteriae_Sd197_uid58213 | 8.081 |
| *Shigella* | Shigella_dysenteriae_1617_uid229875 | 8.057 |
| *Shigella* | Shigella_boydii_CDC_3083_94_uid58415 | 8.011 |
| *Desulfovibrio* | Desulfovibrio_vulgaris__Miyazaki_F__uid59089 | 8.01 |
| *Enterococcus* | Enterococcus_faecium_Aus0004_uid87025 | 7.953 |
| *Rothia* | Rothia_dentocariosa_ATCC_17931_uid49331 | 7.91 |
| *Ruminococcus* | Ruminococcus_albus_7_uid51721 | 7.835 |
| *Clostridium* | Clostridium_novyi_NT_uid58643 | 7.72 |
| *Lactobacillus* | Lactobacillus_acidophilus_La_14_uid201479 | 7.438 |
| *Lactobacillus* | Lactobacillus_acidophilus_NCFM_uid57685 | 7.388 |
| *Selenomonas* | Selenomonas_ruminantium_lactilytica_TAM6421_uid157247 | 7.379 |
| *Gardnerella* | Gardnerella_vaginalis_409_05_uid43211 | 7.277 |
| *Clostridium* | Clostridium_botulinum_BKT015925_uid66203 | 7.112 |
| *Brachyspira* | Brachyspira_pilosicoli_P43_6_78_uid184077 | 6.969 |
| *Desulfovibrio* | Desulfovibrio_vulgaris_DP4_uid58679 | 6.916 |
| *Enterococcus* | Enterococcus_faecium_Aus0085_uid214432 | 6.907 |
| *Brachyspira* | Brachyspira_pilosicoli_95_1000_uid50609 | 6.8 |
| *Desulfovibrio* | Desulfovibrio_vulgaris_RCH1_uid161961 | 6.785 |
| *Pseudomonas* | Pseudomonas_aeruginosa_PAO581_uid219357 | 6.76 |
| *Brachyspira* | Brachyspira_hyodysenteriae_WA1_uid59291 | 6.734 |
| *Campylobacter* | Campylobacter_hominis_ATCC_BAA_381_uid58981 | 6.722 |
| *Desulfovibrio* | Desulfovibrio_vulgaris_Hildenborough_uid57645 | 6.716 |
| *Pseudomonas* | Pseudomonas_aeruginosa_PAO1_uid57945 | 6.682 |
| *Pseudomonas* | Pseudomonas_aeruginosa_PAO1_VE13_uid225027 | 6.681 |
| *Pseudomonas* | Pseudomonas_aeruginosa_PAO1_VE2_uid225026 | 6.681 |
| *Pseudomonas* | Pseudomonas_aeruginosa_c7447m_uid219358 | 6.678 |
| *Pseudomonas* | Pseudomonas_aeruginosa_RP73_uid209328 | 6.627 |
| *Lactobacillus* | Lactobacillus_gasseri_ATCC_33323_uid57687 | 6.624 |
| *Campylobacter* | Campylobacter_concisus_13826_uid58667 | 6.582 |
| *Brachyspira* | Brachyspira_pilosicoli_B2904_uid175255 | 6.573 |
| *Pseudomonas* | Pseudomonas_aeruginosa_M18_uid162089 | 6.56 |
| *Clostridium* | Clostridium_beijerinckii_NCIMB_8052_uid58137 | 6.555 |
| *Leuconostoc* | Leuconostoc_mesenteroides_J18_uid84337 | 6.507 |
| *Pseudomonas* | Pseudomonas_aeruginosa_PA1R_uid228932 | 6.488 |
| *Pseudomonas* | Pseudomonas_aeruginosa_B136_33_uid196598 | 6.477 |
| *Brachyspira* | Brachyspira_pilosicoli_WesB_uid175256 | 6.47 |
| *Pseudomonas* | Pseudomonas_aeruginosa_DK2_uid168996 | 6.462 |
| *Pseudomonas* | Pseudomonas_aeruginosa_PA1_uid228931 | 6.444 |
| *Tannerella* | Tannerella_forsythia_ATCC_43037_uid83157 | 6.43 |
| *Pseudomonas* | Pseudomonas_aeruginosa_UCBPP_PA14_uid57977 | 6.42 |
| *Ethanoligenens* | Ethanoligenens_harbinense_YUAN_3_uid46255 | 6.391 |
| *Clostridium* | Clostridium_saccharoperbutylacetonicum_ATCC_27021_uid189747 | 6.388 |
| *Pseudomonas* | Pseudomonas_aeruginosa_LES431_uid232245 | 6.349 |
| *Pseudomonas* | Pseudomonas_aeruginosa_MTB_uid231150 | 6.339 |
| *Pseudomonas* | Pseudomonas_aeruginosa_LESB58_uid59275 | 6.303 |
| *Leuconostoc* | Leuconostoc_mesenteroides_ATCC_8293_uid57919 | 6.265 |
| *Pseudomonas* | Pseudomonas_aeruginosa_SCV20265_uid232358 | 6.263 |
| *Brachyspira* | Brachyspira_intermedia_PWS_A_uid158369 | 6.202 |
| *Pseudomonas* | Pseudomonas_aeruginosa_NCGM2_S1_uid162173 | 6.151 |
| *Slackia* | Slackia_heliotrinireducens_DSM_20476_uid59051 | 6.127 |
| *Lactobacillus* | Lactobacillus_helveticus_DPC_4571_uid58761 | 6.086 |
| *Clostridium* | Clostridium_tetani_12124569_uid227214 | 6.059 |
| *Clostridium* | Clostridium_tetani_E88_uid57683 | 6.003 |
| *Brachyspira* | Brachyspira_murdochii_DSM_12563_uid48819 | 5.998 |
| *Butyrivibrio* | Butyrivibrio_fibrisolvens_uid197155 | 5.961 |
| *Porphyromonas* | Porphyromonas_gingivalis_ATCC_33277_uid58879 | 5.908 |
| *Porphyromonas* | Porphyromonas_gingivalis_TDC60_uid67407 | 5.821 |
| *Lactobacillus* | Lactobacillus_helveticus_CNRZ32_uid212302 | 5.795 |
| *Butyrivibrio* | Butyrivibrio_proteoclasticus_B316_uid51489 | 5.782 |
| *Lactobacillus* | Lactobacillus_helveticus_R0052_uid174439 | 5.743 |
| *Lactobacillus* | Lactobacillus_helveticus_H10_uid162017 | 5.664 |
| *Enterococcus* | Enterococcus_faecium_DO_uid55353 | 5.663 |
| *Enterococcus* | Enterococcus_faecium_NRRL_B_2354_uid188477 | 5.644 |
| *Raoultella* | Raoultella_ornithinolytica_B6_uid198431 | 5.641 |
| *Clostridium* | Clostridium_botulinum_A_Hall_uid58931 | 5.483 |
| *Pseudomonas* | Pseudomonas_aeruginosa_PA7_uid58627 | 5.428 |
| *Clostridium* | Clostridium_botulinum_A_ATCC_19397_uid58927 | 5.35 |
| *Clostridium* | Clostridium_botulinum_Ba4_657_uid59173 | 5.338 |
| *Clostridium* | Clostridium_botulinum_A_ATCC_3502_uid61579 | 5.32 |
| *Clostridium* | Clostridium_botulinum_H04402_065_uid162091 | 5.304 |
| *Aggregatibacter* | Aggregatibacter_actinomycetemcomitans_ANH9381_uid80743 | 5.267 |
| *Lactobacillus* | Lactobacillus_acidophilus_30SC_uid63605 | 5.255 |
| *Clostridium* | Clostridium_botulinum_F_Langeland_uid58929 | 5.25 |
| *Clostridium* | Clostridium_botulinum_A3_Loch_Maree_uid59149 | 5.231 |
| *Lactobacillus* | Lactobacillus_amylovorus_GRL1118_uid160233 | 5.223 |
| *Clostridium* | Clostridium_botulinum_B1_Okra_uid59147 | 5.215 |
| *Lactobacillus* | Lactobacillus_johnsonii_FI9785_uid41735 | 5.168 |
| *Clostridium* | Clostridium_botulinum_F_230613_uid159513 | 5.158 |
| *Clostridium* | Clostridium_botulinum_A2_Kyoto_uid59229 | 5.153 |
| *Lactobacillus* | Lactobacillus_johnsonii_DPC_6026_uid162057 | 5.131 |
| *Lactococcus* | Lactococcus_garvieae_Lg2_uid161935 | 5.038 |
| *Lactobacillus* | Lactobacillus_johnsonii_NCC_533_uid58029 | 4.997 |
| *Candidatus_Carsonella* | Candidatus_Carsonella_ruddii_CS_isolate_Thao2000_uid172733 | 4.989 |
| *Coriobacterium* | Coriobacterium_glomerans_PW2_uid65787 | 4.986 |
| *Thermus* | Thermus_CCB_US3_UF1_uid81197 | 4.951 |
| *Lactococcus* | Lactococcus_garvieae_ATCC_49156_uid73413 | 4.945 |
| *Lactobacillus* | Lactobacillus_crispatus_ST1_uid48359 | 4.924 |
| *Aggregatibacter* | Aggregatibacter_actinomycetemcomitans_D7S_1_uid46989 | 4.91 |
| *Aggregatibacter* | Aggregatibacter_actinomycetemcomitans_D11S_1_uid41333 | 4.883 |
| *Lactobacillus* | Lactobacillus_johnsonii_N6_2_uid229876 | 4.831 |
| *Lactobacillus* | Lactobacillus_amylovorus_GRL_1112_uid61179 | 4.823 |
| *Leuconostoc* | Leuconostoc_gasicomitatum_LMG_18811_uid50385 | 4.785 |
| *Thermus* | Thermus_thermophilus_JL_18_uid162129 | 4.783 |
| *Leuconostoc* | Leuconostoc_gelidum_JB7_uid175682 | 4.755 |
| *Thermus* | Thermus_thermophilus_SG0_5JP17_16_uid159537 | 4.722 |
| *Thermus* | Thermus_thermophilus_HB8_uid58223 | 4.705 |
| *Gardnerella* | Gardnerella_vaginalis_ATCC_14019_uid55487 | 4.68 |
| *Gardnerella* | Gardnerella_vaginalis_HMP9231_uid162045 | 4.673 |
| *Eubacterium* | Eubacterium_limosum_KIST612_uid59777 | 4.666 |
| *Propionibacterium* | Propionibacterium_freudenreichii_shermanii_CIRM_BIA1_uid49535 | 4.657 |
| *Thermus* | Thermus_thermophilus_HB27_uid58033 | 4.643 |
| *Porphyromonas* | Porphyromonas_gingivalis_W83_uid57641 | 4.62 |
| *Salmonella* | Salmonella_enterica_serovar_Pullorum_S06004_uid214431 | 4.589 |
| *Thermus* | Thermus_oshimai_JL_2_uid178948 | 4.585 |
| *Candidatus_Carsonella* | Candidatus_Carsonella_ruddii_HC_isolate_Thao2000_uid172734 | 4.57 |
| *Salmonella* | Salmonella_enterica_arizonae_serovar_62_z4_z23__uid58191 | 4.561 |
| *Lactobacillus* | Lactobacillus_kefiranofaciens_ZW3_uid67985 | 4.558 |
| *Desulfovibrio* | Desulfovibrio_desulfuricans_ND132_uid63159 | 4.551 |
| *Tropheryma* | Tropheryma_whipplei_TW08_27_uid57961 | 4.537 |
| *Tropheryma* | Tropheryma_whipplei_Twist_uid57705 | 4.534 |
| *Clostridium* | Clostridium_difficile_R20291_uid40921 | 4.518 |
| *Salmonella* | Salmonella_enterica_serovar_Gallinarum_Pullorum_CDC1983_67_uid217770 | 4.51 |
| *Salmonella* | Salmonella_enterica_serovar_Gallinarum_287_91_uid59249 | 4.493 |
| *Salmonella* | Salmonella_enterica_serovar_Gallinarum_pullorum_RKS5078_uid87035 | 4.491 |
| *Salmonella* | Salmonella_enterica_serovar_Javiana_CFSAN001992_uid190101 | 4.473 |
| *Salmonella* | Salmonella_enterica_serovar_Typhi_Ty21a_uid201427 | 4.466 |
| *Salmonella* | Salmonella_enterica_serovar_Enteritidis_P125109_uid59247 | 4.462 |
| *Salmonella* | Salmonella_enterica_serovar_Paratyphi_A_AKU_12601_uid59269 | 4.458 |
| *Salmonella* | Salmonella_enterica_serovar_Paratyphi_A_ATCC_9150_uid58201 | 4.447 |
| *Filifactor* | Filifactor_alocis_ATCC_35896_uid46625 | 4.444 |
| *Salmonella* | Salmonella_enterica_serovar_Typhi_Ty2_uid57973 | 4.419 |
| *Salmonella* | Salmonella_enterica_serovar_Typhi_CT18_uid57793 | 4.418 |
| *Salmonella* | Salmonella_enterica_serovar_Newport_USMARC_S3124_1_uid213895 | 4.413 |
| *Salmonella* | Salmonella_enterica_serovar_Agona_24249_uid230614 | 4.412 |
| *Salmonella* | Salmonella_enterica_serovar_Dublin_CT_02021853_uid58917 | 4.402 |
| *Salmonella* | Salmonella_enterica_serovar_Agona_SL483_uid59431 | 4.402 |
| *Salmonella* | Salmonella_enterica_serovar_Heidelberg_SL476_uid58973 | 4.384 |
| *Salmonella* | Salmonella_enterica_serovar_Typhi_P_stx_12_uid87001 | 4.372 |
| *Salmonella* | Salmonella_enterica_serovar_Typhimurium_SL1344_uid86645 | 4.37 |
| *Salmonella* | Salmonella_enterica_serovar_Typhimurium_ST4_74_uid84393 | 4.37 |
| *Salmonella* | Salmonella_enterica_serovar_Typhimurium_798_uid158047 | 4.355 |
| *Salmonella* | Salmonella_enterica_serovar_Bareilly_CFSAN000189_uid212971 | 4.355 |
| *Candidatus_Carsonella* | Candidatus_Carsonella_ruddii_CE_isolate_Thao2000_uid172732 | 4.351 |
| *Salmonella* | Salmonella_enterica_serovar_Heidelberg_41578_uid212970 | 4.343 |
| *Candidatus_Carsonella* | Candidatus_Carsonella_ruddii_HT_isolate_Thao2000_uid172735 | 4.333 |
| *Salmonella* | Salmonella_enterica_serovar_Heidelberg_B182_uid162195 | 4.316 |
| *Desulfovibrio* | Desulfovibrio_alaskensis_G20_uid57941 | 4.31 |
| *Salmonella* | Salmonella_enterica_Serovar_Heidelberg_CFSAN002069_uid212974 | 4.309 |
| *Salmonella* | Salmonella_enterica_serovar_Paratyphi_B_SPB7_uid59097 | 4.299 |
| *Salmonella* | Salmonella_enterica_serovar_Thompson_RM6836_uid222802 | 4.291 |
| *Salmonella* | Salmonella_enterica_serovar_Newport_SL254_uid58831 | 4.274 |
| *Salmonella* | Salmonella_enterica_serovar_4_5_12_i__08_1736_uid212969 | 4.273 |
| *Desulfovibrio* | Desulfovibrio_aespoeensis_Aspo_2_uid42613 | 4.256 |
| *Salmonella* | Salmonella_enterica_serovar_Typhimurium_D23580_uid86061 | 4.255 |
| *Salmonella* | Salmonella_enterica_Serovar_Typhimurium_var__5__CFSAN001921_uid212972 | 4.244 |
| *Salmonella* | Salmonella_enterica_serovar_Typhimurium_LT2_uid57799 | 4.232 |
| *Salmonella* | Salmonella_enterica_serovar_Schwarzengrund_CVM19633_uid58915 | 4.231 |
| *Salmonella* | Salmonella_enterica_serovar_Choleraesuis_SC_B67_uid58017 | 4.231 |
| *Salmonella* | Salmonella_enterica_serovar_Typhimurium_U288_uid198746 | 4.221 |
| *Salmonella* | Salmonella_enterica_serovar_Paratyphi_C_RKS4594_uid59063 | 4.212 |
| *Salmonella* | Salmonella_enterica_Serovar_Cubana_CFSAN002050_uid212973 | 4.208 |
| *Clostridium* | Clostridium_difficile_630_uid57679 | 4.208 |
| *Salmonella* | Salmonella_enterica_serovar_Typhimurium_UK_1_uid87049 | 4.208 |
| *Acinetobacter* | Acinetobacter_baumannii_SDF_uid61601 | 4.207 |
| *Salmonella* | Salmonella_enterica_serovar_Typhimurium_T000240_uid84397 | 4.203 |
| *Salmonella* | Salmonella_enterica_serovar_Typhimurium_DT2_uid222818 | 4.196 |
| *Salmonella* | Salmonella_enterica_serovar_Typhimurium_14028S_uid86059 | 4.183 |
| *Salmonella* | Salmonella_typhimurium_DT104_uid223287 | 4.175 |
| *Finegoldia* | Finegoldia_magna_ATCC_29328_uid58867 | 4.151 |
| *Clostridium* | Clostridium_difficile_M120_uid158361 | 4.142 |
| *Acinetobacter* | Acinetobacter_baumannii_D1279779_uid190222 | 4.12 |
| *Desulfovibrio* | Desulfovibrio_gigas_DSM_1382_uid221293 | 4.094 |
| *Salmonella* | Salmonella_enterica_serovar_Bovismorbificans_3114_uid218006 | 4.052 |
| *Clostridium* | Clostridium_difficile_M68_uid197172 | 4.01 |
| *Clostridium* | Clostridium_phytofermentans_ISDg_uid58519 | 4.009 |
| *Clostridium* | Clostridium_acetobutylicum_ATCC_824_uid57677 | 4.005 |
| *Clostridium* | Clostridium_acetobutylicum_DSM_1731_uid68293 | 3.998 |
| *Acinetobacter* | Acinetobacter_baumannii_AYE_uid61637 | 3.997 |
| *Clostridium* | Clostridium_acetobutylicum_EA_2018_uid159515 | 3.957 |
| *Acinetobacter* | Acinetobacter_baumannii_ATCC_17978_uid58731 | 3.95 |
| *Acinetobacter* | Acinetobacter_baumannii_MDR_ZJ06_uid158685 | 3.932 |
| *Acinetobacter* | Acinetobacter_baumannii_AB0057_uid59083 | 3.932 |
| *Clostridium* | Clostridium_difficile_ATCC_43255_uid197173 | 3.932 |
| *Clostridium* | Clostridium_difficile_2007855_uid158365 | 3.93 |
| *Salmonella* | Salmonella_bongori_NCTC_12419_uid70155 | 3.924 |
| *Acinetobacter* | Acinetobacter_baumannii_BJAB0868_uid210973 | 3.923 |
| *Acinetobacter* | Acinetobacter_baumannii_1656_2_uid158677 | 3.92 |
| *Acinetobacter* | Acinetobacter_baumannii_TYTH_1_uid176498 | 3.916 |
| *Acinetobacter* | Acinetobacter_baumannii_MDR_TJ_uid162739 | 3.899 |
| *Clostridium* | Clostridium_difficile_BI1_uid158363 | 3.897 |
| *Clostridium* | Clostridium_difficile_CD196_uid41017 | 3.891 |
| *Acinetobacter* | Acinetobacter_baumannii_ACICU_uid58765 | 3.89 |
| *Acinetobacter* | Acinetobacter_baumannii_ZW85_1_uid231518 | 3.887 |
| *Acinetobacter* | Acinetobacter_baumannii_AB307_0294_uid59271 | 3.875 |
| *Clostridium* | Clostridium_difficile_CF5_uid158359 | 3.875 |
| *Acinetobacter* | Acinetobacter_baumannii_BJAB0715_uid210972 | 3.855 |
| *Clostridium* | Clostridium_kluyveri_NBRC_12016_uid59369 | 3.839 |
| *Propionibacterium* | Propionibacterium_acnes_6609_uid162137 | 3.837 |
| *Acinetobacter* | Acinetobacter_ADP1_uid61597 | 3.834 |
| *Clostridium* | Clostridium_cellulovorans_743B_uid51503 | 3.829 |
| *Acinetobacter* | Acinetobacter_baumannii_BJAB07104_uid210971 | 3.827 |
| *Propionibacterium* | Propionibacterium_acnes_KPA171202_uid58101 | 3.827 |
| *Eubacterium* | Eubacterium_cylindroides_T2_87_uid197177 | 3.82 |
| *Propionibacterium* | Propionibacterium_acnes_266_uid162059 | 3.798 |
| *Propionibacterium* | Propionibacterium_acnes_TypeIA2_P_acn33_uid80745 | 3.793 |
| *Propionibacterium* | Propionibacterium_acnes_TypeIA2_P_acn31_uid80733 | 3.791 |
| *Acinetobacter* | Acinetobacter_calcoaceticus_PHEA_2_uid83123 | 3.777 |
| *Clostridium* | Clostridium_kluyveri_DSM_555_uid58885 | 3.776 |
| *Salmonella* | Salmonella_bongori_Sbon_167_uid213088 | 3.761 |
| *Propionibacterium* | Propionibacterium_acnes_C1_uid176501 | 3.761 |
| *Propionibacterium* | Propionibacterium_acnes_TypeIA2_P_acn17_uid80735 | 3.755 |
| *Propionibacterium* | Propionibacterium_acnes_SK137_uid48071 | 3.735 |
| *Propionibacterium* | Propionibacterium_acnes_HL096PA1_uid198524 | 3.735 |
| *Lactobacillus* | Lactobacillus_casei_ATCC_334_uid57985 | 3.665 |
| *Acinetobacter* | Acinetobacter_baumannii_TCDC_AB0715_uid158679 | 3.64 |
| *Candidatus_Zinderia* | Candidatus_Zinderia_insecticola_CARI_uid52459 | 3.595 |
| *Propionibacterium* | Propionibacterium_acnes_ATCC_11828_uid162177 | 3.584 |
| *Clostridium* | Clostridium_acidurici_9a_uid176126 | 3.521 |
| *Weissella* | Weissella_koreensis_KACC_15510_uid68837 | 3.483 |
| *Lactobacillus* | Lactobacillus_casei_LC2W_uid162121 | 3.479 |
| *Clostridium* | Clostridium_autoethanogenum_DSM_10061_uid225029 | 3.455 |
| *Lactobacillus* | Lactobacillus_casei_W56_uid178736 | 3.452 |
| *Lactobacillus* | Lactobacillus_casei_BD_II_uid162119 | 3.448 |
| *Lactobacillus* | Lactobacillus_casei_BL23_uid59237 | 3.445 |
| *Lactobacillus* | Lactobacillus_casei_Zhang_uid50673 | 3.396 |
| *Leuconostoc* | Leuconostoc_carnosum_JB16_uid176371 | 3.392 |
| *Acinetobacter* | Acinetobacter_oleivorans_DR1_uid50119 | 3.384 |
| *Lactobacillus* | Lactobacillus_casei_LOCK919_uid210959 | 3.361 |
| *Desulfovibrio* | Desulfovibrio_africanus_Walvis_Bay_uid66847 | 3.344 |
| *Citrobacter* | Citrobacter_koseri_ATCC_BAA_895_uid58143 | 3.309 |
| *Lactobacillus* | Lactobacillus_paracasei_8700_2_uid55295 | 3.309 |
| *Clostridium* | Clostridium_ljungdahlii_DSM_13528_uid50583 | 3.294 |
| *Clostridium* | Clostridium_pasteurianum_BC1_uid201478 | 3.251 |
| *Desulfovibrio* | Desulfovibrio_magneticus_RS_1_uid59309 | 3.235 |
| *Pediococcus* | Pediococcus_claussenii_ATCC_BAA_344_uid81103 | 3.219 |
| *Pseudomonas* | Pseudomonas_denitrificans_ATCC_13867_uid195459 | 3.207 |
| *Candidatus_Carsonella* | Candidatus_Carsonella_ruddii_PC_isolate_NHV_uid172736 | 3.169 |
| *Lactobacillus* | Lactobacillus_plantarum_16_uid209042 | 3.138 |
| *Mycoplasma* | Mycoplasma_hominis_ATCC_23114_uid41875 | 3.112 |
| *Leuconostoc* | Leuconostoc_citreum_KM20_uid58481 | 3.044 |
| *Citrobacter* | Citrobacter_rodentium_ICC168_uid43089 | 2.982 |
| *Candidatus_Tremblaya* | Candidatus_Tremblaya_princeps_PCVAL_uid159519 | 2.939 |
| *Lactobacillus* | Lactobacillus_plantarum_P8_uid203333 | 2.827 |
| *Streptobacillus* | Streptobacillus_moniliformis_DSM_12112_uid41863 | 2.803 |
| *Lactobacillus* | Lactobacillus_plantarum_ZJ316_uid188689 | 2.779 |
| *Mycoplasma* | Mycoplasma_leachii_99_014_6_uid162031 | 2.744 |
| *Mycoplasma* | Mycoplasma_leachii_PG50_uid60849 | 2.737 |
| *Mycoplasma* | Mycoplasma_capricolum_ATCC_27343_uid58525 | 2.727 |
| *Candidatus_Carsonella* | Candidatus_Carsonella_ruddii_uid58773 | 2.719 |
| *Lactobacillus* | Lactobacillus_plantarum_ST_III_uid53537 | 2.704 |
| *Candidatus_Sulcia* | Candidatus_Sulcia_muelleri_CARI_uid52535 | 2.697 |
| *Paludibacter* | Paludibacter_propionicigenes_WB4_uid60725 | 2.646 |
| *Lactobacillus* | Lactobacillus_plantarum_JDM1_uid59361 | 2.644 |
| *Staphylococcus* | Staphylococcus_aureus_71193_uid162141 | 2.644 |
| *Lactobacillus* | Lactobacillus_brevis_ATCC_367_uid57989 | 2.619 |
| *Leuconostoc* | Leuconostoc_kimchii_IMSNU_11154_uid48589 | 2.61 |
| *Staphylococcus* | Staphylococcus_aureus_SA40_uid221289 | 2.601 |
| *Staphylococcus* | Staphylococcus_aureus_6850_uid217772 | 2.596 |
| *Staphylococcus* | Staphylococcus_aureus_LGA251_uid159391 | 2.593 |
| *Mycoplasma* | Mycoplasma_putrefaciens_KS1_uid72481 | 2.588 |
| *Staphylococcus* | Staphylococcus_aureus_VC40_uid88071 | 2.587 |
| *Staphylococcus* | Staphylococcus_epidermidis_ATCC_12228_uid57861 | 2.586 |
| *Staphylococcus* | Staphylococcus_aureus_COL_uid57797 | 2.578 |
| *Staphylococcus* | Staphylococcus_aureus_HO_5096_0412_uid162163 | 2.578 |
| *Staphylococcus* | Staphylococcus_aureus_M013_uid88065 | 2.577 |
| *Staphylococcus* | Staphylococcus_aureus_SA957_uid221288 | 2.576 |
| *Lactobacillus* | Lactobacillus_plantarum_WCFS1_uid62911 | 2.575 |
| *Staphylococcus* | Staphylococcus_aureus_MW2_uid57903 | 2.574 |
| *Staphylococcus* | Staphylococcus_epidermidis_RP62A_uid57663 | 2.57 |
| *Staphylococcus* | Staphylococcus_aureus_08BA02176_uid175257 | 2.565 |
| *Staphylococcus* | Staphylococcus_aureus_MSSA476_uid57841 | 2.564 |
| *Candidatus_Phytoplasma* | Candidatus_Phytoplasma_mali_uid59087 | 2.562 |
| *Candidatus_Sulcia* | Candidatus_Sulcia_muelleri_SMDSEM_uid59393 | 2.555 |
| *Staphylococcus* | Staphylococcus_aureus_ECT_R_2_uid159389 | 2.551 |
| *Staphylococcus* | Staphylococcus_aureus_TCH60_uid159859 | 2.543 |
| *Neisseria* | Neisseria_meningitidis_alpha14_uid61649 | 2.536 |
| *Staphylococcus* | Staphylococcus_aureus_11819_97_uid159981 | 2.533 |
| *Staphylococcus* | Staphylococcus_aureus_ST228_18412_uid193760 | 2.52 |
| *Staphylococcus* | Staphylococcus_aureus_ST228_18583_uid193761 | 2.52 |
| *Staphylococcus* | Staphylococcus_aureus_uid193759 | 2.52 |
| *Staphylococcus* | Staphylococcus_aureus_ST228_10388_uid193754 | 2.52 |
| *Staphylococcus* | Staphylococcus_aureus_ST228_10497_uid193755 | 2.52 |
| *Staphylococcus* | Staphylococcus_aureus_ST228_16035_uid193757 | 2.52 |
| *Staphylococcus* | Staphylococcus_aureus_ST228_15532_uid193756 | 2.52 |
| *Staphylococcus* | Staphylococcus_aureus_uid193758 | 2.519 |
| *Staphylococcus* | Staphylococcus_aureus_ST398_uid159247 | 2.519 |
| *Candidatus_Arthromitus* | Candidatus_Arthromitus_SFB_rat_Yit_uid73425 | 2.518 |
| *Staphylococcus* | Staphylococcus_aureus_RF122_uid57661 | 2.512 |
| *Staphylococcus* | Staphylococcus_aureus_04_02981_uid161969 | 2.506 |
| *Staphylococcus* | Staphylococcus_aureus_CN1_uid217769 | 2.499 |
| *Lactobacillus* | Lactobacillus_brevis_KB290_uid195560 | 2.496 |
| *Staphylococcus* | Staphylococcus_aureus_N315_uid57837 | 2.485 |
| *Leuconostoc* | Leuconostoc_C2_uid68743 | 2.482 |
| *Staphylococcus* | Staphylococcus_warneri_SG1_uid187059 | 2.482 |
| *Mycoplasma* | Mycoplasma_mycoides_capri_LC_95010_uid66189 | 2.48 |
| *Staphylococcus* | Staphylococcus_aureus_JKD6008_uid159855 | 2.479 |
| *Mycoplasma* | Mycoplasma_putrefaciens_Mput9231_uid198525 | 2.477 |
| *Neisseria* | Neisseria_lactamica_020_06_uid60851 | 2.472 |
| *Candidatus_Tremblaya* | Candidatus_Tremblaya_princeps_PCIT_uid68741 | 2.471 |
| *Staphylococcus* | Staphylococcus_aureus_Bmb9393_uid210640 | 2.47 |
| *Staphylococcus* | Staphylococcus_aureus_NCTC_8325_uid57795 | 2.47 |
| *Staphylococcus* | Staphylococcus_aureus_CC45_uid209174 | 2.469 |
| *Pseudomonas* | Pseudomonas_mendocina_ymp_uid58723 | 2.468 |
| *Staphylococcus* | Staphylococcus_aureus_USA300_TCH1516_uid58925 | 2.467 |
| *Candidatus_Arthromitus* | Candidatus_Arthromitus_SFB_mouse_Japan_uid71379 | 2.467 |
| *Staphylococcus* | Staphylococcus_aureus_JH1_uid58457 | 2.464 |
| *Staphylococcus* | Staphylococcus_aureus_JH9_uid58455 | 2.461 |
| *Staphylococcus* | Staphylococcus_aureus_JKD6159_uid159691 | 2.458 |
| *Staphylococcus* | Staphylococcus_aureus_T0131_uid159861 | 2.457 |
| *Staphylococcus* | Staphylococcus_aureus_Z172_uid225604 | 2.457 |
| *Staphylococcus* | Staphylococcus_aureus_USA300_FPR3757_uid58555 | 2.453 |
| *Staphylococcus* | Staphylococcus_aureus_MRSA252_uid57839 | 2.449 |
| *Staphylococcus* | Staphylococcus_aureus_ED133_uid159689 | 2.442 |
| *Staphylococcus* | Staphylococcus_aureus_ED98_uid41455 | 2.442 |
| *Candidatus_Sulcia* | Candidatus_Sulcia_muelleri_Sulcia_ALF_uid214083 | 2.44 |
| *Burkholderia* | Burkholderia_KJ006_uid165871 | 2.435 |
| *Buchnera* | Buchnera_aphidicola_Cc__Cinara_cedri__uid58579 | 2.434 |
| *Heliobacterium* | Heliobacterium_modesticaldum_Ice1_uid58279 | 2.434 |
| *Staphylococcus* | Staphylococcus_aureus_Mu50_uid57835 | 2.428 |
| *Staphylococcus* | Staphylococcus_aureus_Mu3_uid58817 | 2.424 |
| *Staphylococcus* | Staphylococcus_aureus_Newman_uid58839 | 2.421 |
| *Neisseria* | Neisseria_gonorrhoeae_TCDC_NG08107_uid161097 | 2.414 |
| *Staphylococcus* | Staphylococcus_aureus_TW20_uid159241 | 2.398 |
| *Staphylococcus* | Staphylococcus_saprophyticus_ATCC_15305_uid58411 | 2.395 |
| *Clostridium* | Clostridium_cellulolyticum_H10_uid58709 | 2.388 |
| *Staphylococcus* | Staphylococcus_aureus_MSHR1132_uid89393 | 2.388 |
| *Candidatus_Arthromitus* | Candidatus_Arthromitus_SFB_mouse_Yit_uid159517 | 2.388 |
| *Staphylococcus* | Staphylococcus_aureus_55_2053_uid55909 | 2.384 |
| *Pseudomonas* | Pseudomonas_resinovorans_NBRC_106553_uid208671 | 2.377 |
| *Cryptobacterium* | Cryptobacterium_curtum_DSM_15641_uid59041 | 2.375 |
| *Staphylococcus* | Staphylococcus_pasteuri_SP1_uid226267 | 2.368 |
| *Neisseria* | Neisseria_gonorrhoeae_FA_1090_uid57611 | 2.361 |
| *Pseudomonas* | Pseudomonas_stutzeri_DSM_10701_uid170940 | 2.347 |
| *Neisseria* | Neisseria_meningitidis_053442_uid58587 | 2.331 |
| *Mycoplasma* | Mycoplasma_mycoides_SC_Gladysdale_uid197153 | 2.325 |
| *Candidatus_Sulcia* | Candidatus_Sulcia_muelleri_GWSS_uid58943 | 2.324 |
| *Neisseria* | Neisseria_meningitidis_Z2491_uid57819 | 2.313 |
| *Mycoplasma* | Mycoplasma_mycoides_SC_PG1_uid58031 | 2.308 |
| *Lactobacillus* | Lactobacillus_buchneri_NRRL_B_30929_uid66205 | 2.303 |
| *Neisseria* | Neisseria_gonorrhoeae_NCCP11945_uid59191 | 2.302 |
| *Lactobacillus* | Lactobacillus_rhamnosus_ATCC_8530_uid162169 | 2.272 |
| *Candidatus_Nasuia* | Candidatus_Nasuia_deltocephalinicola_NAS_ALF_uid214084 | 2.269 |
| *Dehalococcoides* | Dehalococcoides_ethenogenes_195_uid57763 | 2.266 |
| *Staphylococcus* | Staphylococcus_haemolyticus_JCSC1435_uid62919 | 2.263 |
| *Propionibacterium* | Propionibacterium_propionicum_F0230a_uid170533 | 2.257 |
| *Burkholderia* | Burkholderia_multivorans_ATCC_17616_uid58697 | 2.251 |
| *Burkholderia* | Burkholderia_multivorans_ATCC_17616_uid58909 | 2.248 |
| *Neisseria* | Neisseria_meningitidis_G2136_uid162085 | 2.242 |
| *Neisseria* | Neisseria_meningitidis_alpha710_uid161971 | 2.234 |
| *Neisseria* | Neisseria_meningitidis_WUE_2594_uid162093 | 2.226 |
| *Lactobacillus* | Lactobacillus_rhamnosus_LOCK908_uid210958 | 2.216 |
| *Burkholderia* | Burkholderia_ambifaria_MC40_6_uid58701 | 2.214 |
| *Neisseria* | Neisseria_meningitidis_8013_uid161967 | 2.213 |
| *Lactobacillus* | Lactobacillus_rhamnosus_GG_uid161983 | 2.211 |
| *Neisseria* | Neisseria_meningitidis_M01_240149_uid162079 | 2.21 |
| *Lactobacillus* | Lactobacillus_buchneri_uid73657 | 2.201 |
| *Lactobacillus* | Lactobacillus_rhamnosus_GG_uid59313 | 2.2 |
| *Lactobacillus* | Lactobacillus_rhamnosus_LOCK900_uid210957 | 2.192 |
| *Pseudomonas* | Pseudomonas_stutzeri_A1501_uid58641 | 2.191 |
| *Clostridium* | Clostridium_lentocellum_DSM_5427_uid49117 | 2.189 |
| *Symbiobacterium* | Symbiobacterium_thermophilum_IAM_14863_uid58165 | 2.185 |
| *Lactobacillus* | Lactobacillus_rhamnosus_Lc_705_uid59315 | 2.184 |
| *Candidatus_Sulcia* | Candidatus_Sulcia_muelleri_DMIN_uid47075 | 2.182 |
| *Neisseria* | Neisseria_meningitidis_NZ_05_33_uid162077 | 2.182 |
| *Pseudomonas* | Pseudomonas_stutzeri_ATCC_17588___LMG_11199_uid68749 | 2.181 |
| *Neisseria* | Neisseria_meningitidis_FAM18_uid57825 | 2.179 |
| *Burkholderia* | Burkholderia_cenocepacia_HI2424_uid58369 | 2.177 |
| *Spiroplasma* | Spiroplasma_diminutum_CUAS_1_uid212976 | 2.175 |
| *Neisseria* | Neisseria_meningitidis_M01_240355_uid162075 | 2.164 |
| *Neisseria* | Neisseria_meningitidis_H44_76_uid162083 | 2.163 |
| *Neisseria* | Neisseria_meningitidis_M04_240196_uid162081 | 2.155 |
| *Pseudomonas* | Pseudomonas_stutzeri_DSM_4166_uid162113 | 2.152 |
| *Neisseria* | Neisseria_meningitidis_MC58_uid57817 | 2.151 |
| *Burkholderia* | Burkholderia_YI23_uid81081 | 2.148 |
| *Burkholderia* | Burkholderia_RPE64_uid205541 | 2.146 |
| *Clostridium* | Clostridium_BNL1100_uid84307 | 2.139 |
| *Burkholderia* | Burkholderia_cenocepacia_MC0_3_uid58769 | 2.135 |
| *Burkholderia* | Burkholderia_cepacia_GG4_uid173858 | 2.134 |
| *Burkholderia* | Burkholderia_mallei_NCTC_10229_uid58383 | 2.134 |
| *Pseudomonas* | Pseudomonas_fulva_12_X_uid67351 | 2.132 |
| *Burkholderia* | Burkholderia_ambifaria_AMMD_uid58303 | 2.116 |
| *Burkholderia* | Burkholderia_mallei_SAVP1_uid58387 | 2.109 |
| *Burkholderia* | Burkholderia_mallei_NCTC_10247_uid58385 | 2.108 |
| *Proteus* | Proteus_mirabilis_BB2000_uid214430 | 2.106 |
| *Burkholderia* | Burkholderia_vietnamiensis_G4_uid58075 | 2.104 |
| *Staphylococcus* | Staphylococcus_aureus_M1_uid197263 | 2.101 |
| *Burkholderia* | Burkholderia_mallei_ATCC_23344_uid57725 | 2.093 |
| *Burkholderia* | Burkholderia_383_uid58073 | 2.087 |
| *Enterococcus* | Enterococcus_hirae_ATCC_9790_uid70619 | 2.087 |
| *Candidatus_Carsonella* | Candidatus_Carsonella_ruddii_DC_uid213383 | 2.052 |
| *Pseudomonas* | Pseudomonas_entomophila_L48_uid58639 | 2.032 |
| *Ureaplasma* | Ureaplasma_parvum_serovar_3_ATCC_27815_uid58887 | 2.027 |
| *Ureaplasma* | Ureaplasma_parvum_serovar_3_ATCC_700970_uid57711 | 2.027 |
| *Clostridium* | _Clostridium__sticklandii_uid59585 | 2.027 |
| *Clostridium* | Clostridium_thermocellum_DSM_1313_uid161989 | 2.022 |
| *Burkholderia* | Burkholderia_cenocepacia_J2315_uid57953 | 2.021 |
| *Burkholderia* | Burkholderia_pseudomallei_668_uid58389 | 2.02 |
| *Staphylococcus* | Staphylococcus_lugdunensis_HKU09_01_uid46233 | 2.009 |
| *Proteus* | Proteus_mirabilis_HI4320_uid61599 | 1.983 |
| *Pseudomonas* | Pseudomonas_mendocina_NK_01_uid66299 | 1.981 |
| *Burkholderia* | Burkholderia_pseudomallei_NCTC_13179_uid226109 | 1.979 |
| *Staphylococcus* | Staphylococcus_carnosus_TM300_uid59401 | 1.978 |
| *Cronobacter* | Cronobacter_sakazakii_45402_uid231516 | 1.976 |
| *Burkholderia* | Burkholderia_pseudomallei_BPC006_uid174460 | 1.964 |
| *Burkholderia* | Burkholderia_pseudomallei_1106a_uid58515 | 1.962 |
| *Pseudomonas* | Pseudomonas_stutzeri_RCH2_uid184342 | 1.959 |
| *Thermoanaerobacter* | Thermoanaerobacter_X513_uid53065 | 1.958 |
| *Burkholderia* | Burkholderia_thailandensis_E264_uid58081 | 1.957 |
| *Anaerococcus* | Anaerococcus_prevotii_DSM_20548_uid59219 | 1.957 |
| *Thermoanaerobacter* | Thermoanaerobacter_X514_uid58589 | 1.954 |
| *Burkholderia* | Burkholderia_thailandensis_MSMB121_uid201037 | 1.952 |
| *Pseudomonas* | Pseudomonas_stutzeri_CCUG_29243_uid168379 | 1.95 |
| *Clostridium* | Clostridium_thermocellum_ATCC_27405_uid57917 | 1.947 |
| *Burkholderia* | Burkholderia_pseudomallei_MSHR305_uid213227 | 1.942 |
| *Burkholderia* | Burkholderia_CCGE1002_uid42523 | 1.942 |
| *Salmonella* | Salmonella_enterica_serovar_Weltevreden_2007_60_3289_1_uid178014 | 1.941 |
| *Candidatus_Azobacteroides* | Candidatus_Azobacteroides_pseudotrichonymphae_genomovar__CFP2_uid59163 | 1.94 |
| *Alkaliphilus* | Alkaliphilus_oremlandii_OhILAs_uid58495 | 1.934 |
| *Burkholderia* | Burkholderia_cenocepacia_AU_1054_uid58371 | 1.933 |
| *Desulfotomaculum* | Desulfotomaculum_carboxydivorans_CO_1_SRB_uid67317 | 1.927 |
| *Enterococcus* | Enterococcus_casseliflavus_EC20_uid55693 | 1.927 |
| *Pseudomonas* | Pseudomonas_putida_HB3267_uid184078 | 1.924 |
| *Mesoplasma* | Mesoplasma_florum_L1_uid58055 | 1.923 |
| *Burkholderia* | Burkholderia_pseudomallei_K96243_uid57733 | 1.921 |
| *Burkholderia* | Burkholderia_pseudomallei_MSHR346_uid55259 | 1.919 |
| *Mycoplasma* | Mycoplasma_mobile_163K_uid58077 | 1.915 |
| *Mycoplasma* | Mycoplasma_cynos_C142_uid184824 | 1.91 |
| *Burkholderia* | Burkholderia_pseudomallei_1026b_uid162511 | 1.908 |
| *Staphylococcus* | Staphylococcus_lugdunensis_N920143_uid162143 | 1.906 |
| *Burkholderia* | Burkholderia_pseudomallei_1710b_uid58391 | 1.889 |
| *Burkholderia* | Burkholderia_glumae_BGR1_uid59397 | 1.871 |
| *Mesoplasma* | Mesoplasma_florum_W37_uid224253 | 1.869 |
| *Cronobacter* | Cronobacter_sakazakii_ATCC_BAA_894_uid58145 | 1.865 |
| *Burkholderia* | Burkholderia_phymatum_STM815_uid58699 | 1.858 |
| *Propionibacterium* | Propionibacterium_avidum_44067_uid197361 | 1.855 |
| *Mycoplasma* | Mycoplasma_fermentans_JER_uid53543 | 1.844 |
| *Pseudomonas* | Pseudomonas_monteilii_SB3101_uid232253 | 1.827 |
| *Pseudomonas* | Pseudomonas_putida_S16_uid68747 | 1.827 |
| *Cronobacter* | Cronobacter_sakazakii_Sp291_uid189241 | 1.825 |
| *Cronobacter* | Cronobacter_sakazakii_ES15_uid167045 | 1.818 |
| *Pseudomonas* | Pseudomonas_monteilii_SB3078_uid232252 | 1.813 |
| *Erysipelothrix* | Erysipelothrix_rhusiopathiae_SY1027_uid206518 | 1.795 |
| *Spiroplasma* | Spiroplasma_taiwanense_CT_1_uid212975 | 1.79 |
| *Cronobacter* | Cronobacter_turicensis_z3032_uid40821 | 1.779 |
| *Corynebacterium* | Corynebacterium_variabile_DSM_44702_uid62003 | 1.775 |
| *Pseudomonas* | Pseudomonas_fluorescens_CHA0_uid203393 | 1.771 |
| *Erysipelothrix* | Erysipelothrix_rhusiopathiae_Fujisawa_uid68021 | 1.77 |
| *Clostridium* | Clostridium_clariflavum_DSM_19732_uid82345 | 1.739 |
| *Pseudomonas* | Pseudomonas_fluorescens_Pf_5_uid57937 | 1.735 |
| *Leptotrichia* | Leptotrichia_buccalis_C_1013_b_uid59211 | 1.734 |
| *Pseudomonas* | Pseudomonas_putida_NBRC_14164_uid208670 | 1.733 |
| *Acholeplasma* | Acholeplasma_laidlawii_PG_8A_uid58901 | 1.728 |
| *Burkholderia* | Burkholderia_CCGE1001_uid42975 | 1.726 |
| *Mycoplasma* | Mycoplasma_crocodyli_MP145_uid47087 | 1.719 |
| *Pseudomonas* | Pseudomonas_VLB120_uid226717 | 1.716 |
| *Pseudomonas* | Pseudomonas_putida_GB_1_uid58735 | 1.709 |
| *Burkholderia* | Burkholderia_gladioli_BSR3_uid66301 | 1.707 |
| *Actinobacillus* | Actinobacillus_pleuropneumoniae_serovar_3_JL03_uid58891 | 1.704 |
| *Burkholderia* | Burkholderia_phenoliruptrix_BR3459a_uid176370 | 1.701 |
| *Serratia* | Serratia_marcescens_FGI94_uid185180 | 1.699 |
| *Serratia* | Serratia_marcescens_WW4_uid188478 | 1.696 |
| *Thermoanaerobacterium* | Thermoanaerobacterium_thermosaccharolyticum_M0795_uid184821 | 1.694 |
| *Pseudomonas* | Pseudomonas_poae_RE_1_1_14_uid188480 | 1.692 |
| *Staphylococcus* | Staphylococcus_pseudintermedius_HKU10_03_uid62125 | 1.69 |
| *Thermoanaerobacterium* | Thermoanaerobacterium_xylanolyticum_LX_11_uid63163 | 1.689 |
| *Pseudomonas* | Pseudomonas_putida_BIRD_1_uid162055 | 1.682 |
| *Staphylococcus* | Staphylococcus_pseudintermedius_ED99_uid162109 | 1.68 |
| *Enterococcus* | Enterococcus_mundtii_QU_25_uid229420 | 1.676 |
| *Thermaerobacter* | Thermaerobacter_marianensis_DSM_12885_uid61727 | 1.674 |
| *Desulfomicrobium* | Desulfomicrobium_baculatum_DSM_4028_uid59217 | 1.667 |
| *Thermoanaerobacterium* | Thermoanaerobacterium_thermosaccharolyticum_DSM_571_uid51639 | 1.665 |
| *Actinobacillus* | Actinobacillus_pleuropneumoniae_serovar_7_AP76_uid59231 | 1.662 |
| *Mycoplasma* | Mycoplasma_fermentans_PG18_uid197154 | 1.655 |
| *Mycoplasma* | Mycoplasma_fermentans_M64_uid62099 | 1.655 |
| *Actinobacillus* | Actinobacillus_pleuropneumoniae_serovar_5b_L20_uid58789 | 1.653 |
| *Mycoplasma* | Mycoplasma_hyorhinis_HUB_1_uid51695 | 1.638 |
| *Mycoplasma* | Mycoplasma_agalactiae_PG2_uid61619 | 1.632 |
| *Pseudomonas* | Pseudomonas_putida_W619_uid58651 | 1.632 |
| *Treponema* | Treponema_brennaborense_DSM_12168_uid66607 | 1.629 |
| *Lawsonia* | Lawsonia_intracellularis_N343_uid186598 | 1.626 |
| *Lawsonia* | Lawsonia_intracellularis_PHE_MN1_00_uid61575 | 1.624 |
| *Mycoplasma* | Mycoplasma_synoviae_53_uid58061 | 1.619 |
| *Mycoplasma* | Mycoplasma_hyorhinis_SK76_uid181997 | 1.616 |
| *Desulfotomaculum* | Desulfotomaculum_ruminis_DSM_2154_uid67507 | 1.613 |
| *Pseudomonas* | Pseudomonas_fluorescens_Pf0_1_uid57591 | 1.612 |
| *Desulfovibrio* | Desulfovibrio_salexigens_DSM_2638_uid59223 | 1.612 |
| *Mycoplasma* | Mycoplasma_hyorhinis_MCLD_uid162087 | 1.609 |
| *Actinobacillus* | Actinobacillus_suis_H91_0380_uid176363 | 1.606 |
| *Pseudomonas* | Pseudomonas_putida_KT2440_uid57843 | 1.6 |
| *Mycoplasma* | Mycoplasma_hyorhinis_DBS_1050_uid228933 | 1.598 |
| *Mycoplasma* | Mycoplasma_hyorhinis_GDL_1_uid87003 | 1.591 |
| *Burkholderia* | Burkholderia_CCGE1003_uid46253 | 1.59 |
| *Pseudomonas* | Pseudomonas_putida_F1_uid58355 | 1.58 |
| *Pseudomonas* | Pseudomonas_ND6_uid167583 | 1.561 |
| *Corynebacterium* | Corynebacterium_argentoratense_DSM_44202_uid217419 | 1.558 |
| *Pseudomonas* | Pseudomonas_fluorescens_A506_uid165185 | 1.558 |
| *Spiroplasma* | Spiroplasma_syrphidicola_EA_1_uid205054 | 1.551 |
| *Thermoanaerobacter* | Thermoanaerobacter_brockii_finnii_Ako_1_uid55639 | 1.546 |
| *Thermobacillus* | Thermobacillus_composti_KWC4_uid74021 | 1.545 |
| *Pasteurella* | Pasteurella_multocida_36950_uid86887 | 1.542 |
| *Pseudomonas* | Pseudomonas_putida_UW4_uid182733 | 1.533 |
| *Thermoanaerobacter* | Thermoanaerobacter_pseudethanolicus_ATCC_33223_uid58339 | 1.529 |
| *Pasteurella* | Pasteurella_multocida_Pm70_uid57627 | 1.523 |
| *Thermanaerovibrio* | Thermanaerovibrio_acidaminovorans_DSM_6589_uid41925 | 1.52 |
| *Spiroplasma* | Spiroplasma_chrysopicola_DF_1_uid205053 | 1.518 |
| *Buchnera* | Buchnera_aphidicola_Ua__Uroleucon_ambrosiae__uid158535 | 1.508 |
| *Ureaplasma* | Ureaplasma_urealyticum_serovar_10_ATCC_33699_uid59011 | 1.507 |
| *Pseudomonas* | Pseudomonas_putida_H8234_uid208673 | 1.504 |
| *Pseudomonas* | Pseudomonas_fluorescens_F113_uid87037 | 1.496 |
| *Pseudomonas* | Pseudomonas_brassicacearum_NFM421_uid66303 | 1.494 |
| *Mycoplasma* | Mycoplasma_agalactiae_uid46679 | 1.493 |
| *Treponema* | Treponema_succinifaciens_DSM_2489_uid65781 | 1.492 |
| *Serratia* | Serratia_plymuthica_S13_uid210642 | 1.482 |
| *Candidatus_Phytoplasma* | Candidatus_Phytoplasma_australiense_uid61641 | 1.481 |
| *Thermoanaerobacter* | Thermoanaerobacter_italicus_Ab9_uid46241 | 1.477 |
| *Desulfotomaculum* | Desulfotomaculum_reducens_MI_1_uid58277 | 1.474 |
| *Pasteurella* | Pasteurella_multocida_3480_uid161955 | 1.473 |
| *Pasteurella* | Pasteurella_multocida_HN06_uid156881 | 1.466 |
| *Mycoplasma* | Mycoplasma_bovis_HB0801_uid168665 | 1.464 |
| *Mycoplasma* | Mycoplasma_bovis_PG45_uid60859 | 1.461 |
| *Pseudomonas* | Pseudomonas_fluorescens_SBW25_uid158693 | 1.459 |
| *Serratia* | Serratia_odorifera_4Rx13_uid42253 | 1.452 |
| *Corynebacterium* | Corynebacterium_urealyticum_DSM_7111_uid188688 | 1.446 |
| *Burkholderia* | Burkholderia_phytofirmans_PsJN_uid58729 | 1.445 |
| *Thermoanaerobacter* | Thermoanaerobacter_mathranii_A3_uid49481 | 1.444 |
| *Alkaliphilus* | Alkaliphilus_metalliredigens_QYMF_uid58171 | 1.437 |
| *Pseudomonas* | Pseudomonas_TKP_uid232248 | 1.435 |
| *Blattabacterium* | Blattabacterium__Cryptocercus_punctulatus__Cpu_uid81083 | 1.432 |
| *Edwardsiella* | Edwardsiella_tarda_FL6_60_uid159657 | 1.426 |
| *Pseudomonas* | Pseudomonas_putida_DOT_T1E_uid171260 | 1.426 |
| *Serratia* | Serratia_liquefaciens_ATCC_27592_uid212306 | 1.413 |
| *Buchnera* | Buchnera_aphidicola__Cinara_tujafilina__uid68101 | 1.404 |
| *Serratia* | Serratia_AS13_uid162065 | 1.403 |
| *Blattabacterium* | Blattabacterium__Blaberus_giganteus__uid165873 | 1.403 |
| *Serratia* | Serratia_plymuthica_AS9_uid67313 | 1.4 |
| *Serratia* | Serratia_AS12_uid67315 | 1.4 |
| *Corynebacterium* | Corynebacterium_urealyticum_DSM_7109_uid61639 | 1.4 |
| *Serratia* | Serratia_proteamaculans_568_uid58725 | 1.399 |
| *Burkholderia* | Burkholderia_rhizoxinica_HKI_454_uid60487 | 1.394 |
| *Edwardsiella* | Edwardsiella_tarda_C07_087_uid193773 | 1.393 |
| *Burkholderia* | Burkholderia_xenovorans_LB400_uid57823 | 1.388 |
| *Syntrophobotulus* | Syntrophobotulus_glycolicus_DSM_8271_uid63343 | 1.387 |
| *Desulfotomaculum* | Desulfotomaculum_kuznetsovii_DSM_6115_uid67357 | 1.383 |
| *Corynebacterium* | Corynebacterium_jeikeium_K411_uid58399 | 1.382 |
| *Halanaerobium* | Halanaerobium_praevalens_DSM_2228_uid161959 | 1.381 |
| *Listeria* | Listeria_welshimeri_serovar_6b_SLCC5334_uid61605 | 1.372 |
| *Thermoanaerobacter* | Thermoanaerobacter_wiegelii_Rt8_B1_uid52581 | 1.365 |
| *Mycoplasma* | Mycoplasma_gallisepticum_S6_uid200523 | 1.364 |
| *Blattabacterium* | Blattabacterium__Blattella_germanica__Bge_uid41533 | 1.359 |
| *Bacteroides* | Bacteroides_CF50_uid222805 | 1.354 |
| *Geobacter* | Geobacter_bemidjiensis_Bem_uid58749 | 1.35 |
| *Geobacter* | Geobacter_M18_uid55771 | 1.346 |
| *Candidatus_Portiera* | Candidatus_Portiera_aleyrodidarum_TV_uid195460 | 1.345 |
| *Geobacter* | Geobacter_M21_uid59037 | 1.344 |
| *Edwardsiella* | Edwardsiella_tarda_EIB202_uid41819 | 1.344 |
| *Desulfovibrio* | Desulfovibrio_hydrothermalis_AM13___DSM_14728_uid184831 | 1.34 |
| *Blattabacterium* | Blattabacterium__Panesthia_angustipennis_spadica__BPAA_uid193717 | 1.324 |
| *Caldicellulosiruptor* | Caldicellulosiruptor_owensensis_OL_uid60165 | 1.322 |
| *Kinetoplastibacterium* | Candidatus_Kinetoplastibacterium_desouzaii_TCC079E_uid189750 | 1.322 |
| *Dehalobacter* | Dehalobacter_CF_uid177714 | 1.321 |
| *Bacillus* | Bacillus_coagulans_2_6_uid68053 | 1.313 |
| *Corynebacterium* | Corynebacterium_terpenotabidum_Y_11_uid210639 | 1.306 |
| *Blattabacterium* | Blattabacterium__Mastotermes_darwiniensis__MADAR_uid77127 | 1.304 |
| *Pseudomonas* | Pseudomonas_syringae_B728a_uid57931 | 1.298 |
| *Kinetoplastibacterium* | Candidatus_Kinetoplastibacterium_crithidii_TCC036E_uid189749 | 1.297 |
| *Kinetoplastibacterium* | Candidatus_Kinetoplastibacterium_crithidii__ex_Angomonas_deanei_ATCC_30255 | 1.29 |
| *Melissococcus* | Melissococcus_plutonius_DAT561_uid89371 | 1.28 |
| *Mycoplasma* | Mycoplasma_penetrans_HF_2_uid57729 | 1.272 |
| *Elusimicrobium* | Elusimicrobium_minutum_Pei191_uid58949 | 1.27 |
| *Caldicellulosiruptor* | Caldicellulosiruptor_obsidiansis_OB47_uid51501 | 1.266 |
| *Corynebacterium* | Corynebacterium_halotolerans_YIM_70093___DSM_44683_uid189953 | 1.266 |
| *Dehalobacter* | Dehalobacter_11DCA_uid177715 | 1.265 |
| *Corynebacterium* | Corynebacterium_maris_DSM_45190_uid214081 | 1.262 |
| *Candidatus_Phytoplasma* | Candidatus_Phytoplasma_solani_284_09_uid225030 | 1.261 |
| *Desulfotomaculum* | Desulfotomaculum_gibsoniae_DSM_7213_uid76945 | 1.261 |
| *Listeria* | Listeria_monocytogenes_Finland_1998_uid54443 | 1.257 |
| *Bacillus* | Bacillus_cytotoxicus_NVH_391_98_uid58317 | 1.257 |
| *Candidatus_Tremblaya* | Candidatus_Tremblaya_phenacola_PAVE_uid209173 | 1.254 |
| *Bacillus* | Bacillus_coagulans_36D1_uid54335 | 1.254 |
| *Deinococcus* | Deinococcus_proteolyticus_MRP_uid63399 | 1.253 |
| *Carnobacterium* | Carnobacterium_WN1359_uid225603 | 1.252 |
| *Mycoplasma* | Mycoplasma_pulmonis_UAB_CTIP_uid61569 | 1.245 |
| *Melissococcus* | Melissococcus_plutonius_ATCC_35311_uid66803 | 1.244 |
| *Mycoplasma* | Mycoplasma_gallisepticum_NC06_2006_080_5_2P_uid172629 | 1.242 |
| *Mycoplasma* | Mycoplasma_gallisepticum_NC08_2008_031_4_3P_uid172631 | 1.241 |
| *Alicyclobacillus* | Alicyclobacillus_acidocaldarius_DSM_446_uid59199 | 1.241 |
| *Mycoplasma* | Mycoplasma_gallisepticum_WI01_2001_043_13_2P_uid172628 | 1.239 |
| *Geobacter* | Geobacter_sulfurreducens_KN400_uid161977 | 1.238 |
| *Mycoplasma* | Mycoplasma_bovis_Hubei_1_uid68691 | 1.238 |
| *Listeria* | Listeria_monocytogenes_N53_1_uid193767 | 1.234 |
| *Buchnera* | Buchnera_aphidicola_Sg__Schizaphis_graminum__uid57913 | 1.233 |
| *Listeria* | Listeria_monocytogenes_L312_uid175768 | 1.233 |
| *Listeria* | Listeria_monocytogenes_La111_uid193768 | 1.229 |
| *Listeria* | Listeria_monocytogenes_Clip80459_uid59317 | 1.226 |
| *Geobacter* | Geobacter_metallireducens_GS_15_uid57731 | 1.223 |
| *Mycoplasma* | Mycoplasma_gallisepticum_F_uid162001 | 1.223 |
| *Listeria* | Listeria_monocytogenes_10403S_uid54461 | 1.222 |
| *Buchnera* | Buchnera_aphidicola_Ak__Acyrthosiphon_kondoi__uid158533 | 1.222 |
| *Listeria* | Listeria_monocytogenes_serotype_1_2b_SLCC2755_uid52455 | 1.221 |
| *Pelotomaculum* | Pelotomaculum_thermopropionicum_SI_uid58877 | 1.219 |
| *Mycoplasma* | Mycoplasma_gallisepticum_NY01_2001_047_5_1P_uid172627 | 1.217 |
| *Buchnera* | Buchnera_aphidicola_Tuc7__Acyrthosiphon_pisum__uid59283 | 1.217 |
| *Buchnera* | Buchnera_aphidicola_5A__Acyrthosiphon_pisum__uid59285 | 1.216 |
| *Mycoplasma* | Mycoplasma_gallisepticum_R_high__uid161999 | 1.213 |
| *Cupriavidus* | Cupriavidus_taiwanensis_LMG_19424_uid61615 | 1.212 |
| *Buchnera* | Buchnera_aphidicola_APS__Acyrthosiphon_pisum__uid57805 | 1.211 |
| *Moorella* | Moorella_thermoacetica_ATCC_39073_uid58051 | 1.209 |
| *Listeria* | Listeria_monocytogenes_07PF0776_uid162185 | 1.208 |
| *Propionibacterium* | Propionibacterium_acidipropionici_ATCC_4875_uid179069 | 1.208 |
| *Blattabacterium* | Blattabacterium__Nauphoeta_cinerea__uid222815 | 1.207 |
| *Mycoplasma* | Mycoplasma_genitalium_G37_uid57707 | 1.207 |
| *Listeria* | Listeria_monocytogenes_08_5923_uid43727 | 1.205 |
| *Mycoplasma* | Mycoplasma_gallisepticum_R_low__uid57993 | 1.205 |
| *Listeria* | Listeria_seeligeri_serovar_1_2b_SLCC3954_uid46215 | 1.204 |
| *Desulfotomaculum* | Desulfotomaculum_acetoxidans_DSM_771_uid59109 | 1.204 |
| *Listeria* | Listeria_monocytogenes_SLCC7179_uid175107 | 1.203 |
| *Geobacillus* | Geobacillus_thermoleovorans_CCB_US3_UF5_uid82949 | 1.202 |
| *Buchnera* | Buchnera_aphidicola_LL01__Acyrthosiphon_pisum__uid158843 | 1.202 |
| *Desulfitobacterium* | Desulfitobacterium_hafniense_Y51_uid58605 | 1.202 |
| *Listeria* | Listeria_monocytogenes_serotype_4b_F2365_uid57689 | 1.202 |
| *Listeria* | Listeria_monocytogenes_EGD_e_uid61583 | 1.201 |
| *Listeria* | Listeria_monocytogenes_SLCC2376_uid175111 | 1.2 |
| *Pseudomonas* | Pseudomonas_syringae_phaseolicola_1448A_uid58099 | 1.199 |
| *Mycoplasma* | Mycoplasma_gallisepticum_VA94_7994_1_7P_uid172624 | 1.199 |
| *Buchnera* | Buchnera_aphidicola_TLW03__Acyrthosiphon_pisum__uid158849 | 1.198 |
| *Listeria* | Listeria_monocytogenes_SLCC2378_uid175105 | 1.198 |
| *Pseudomonas* | Pseudomonas_syringae_tomato_DC3000_uid57967 | 1.197 |
| *Listeria* | Listeria_monocytogenes_SLCC5850_uid175110 | 1.197 |
| *Mycoplasma* | Mycoplasma_gallisepticum_NC95_13295_2_2P_uid172625 | 1.197 |
| *Caldicellulosiruptor* | Caldicellulosiruptor_kronotskyensis_2002_uid60491 | 1.196 |
| *Buchnera* | Buchnera_aphidicola_JF99__Acyrthosiphon_pisum__uid158847 | 1.196 |
| *Listeria* | Listeria_monocytogenes_uid43671 | 1.194 |
| *Listeria* | Listeria_monocytogenes_serotype_7_SLCC2482_uid174871 | 1.194 |
| *Geobacter* | Geobacter_sulfurreducens_PCA_uid57743 | 1.194 |
| *Mycoplasma* | Mycoplasma_genitalium_M2321_uid173373 | 1.193 |
| *Mycoplasma* | Mycoplasma_gallisepticum_CA06_2006_052_5_2P_uid172630 | 1.191 |
| *Listeria* | Listeria_monocytogenes_serotype_4b_LL195_uid182103 | 1.191 |
| *Buchnera* | Buchnera_aphidicola_JF98__Acyrthosiphon_pisum__uid158845 | 1.19 |
| *Listeria* | Listeria_monocytogenes_FSL_R2_561_uid54441 | 1.19 |
| *Blattabacterium* | Blattabacterium__Periplaneta_americana__BPLAN_uid41287 | 1.189 |
| *Listeria* | Listeria_monocytogenes_SLCC2540_uid175106 | 1.189 |
| *Listeria* | Listeria_monocytogenes_ATCC_19117_uid175109 | 1.188 |
| *Desulfarculus* | Desulfarculus_baarsii_DSM_2075_uid51371 | 1.187 |
| *Listeria* | Listeria_monocytogenes_serotype_1_2c_SLCC2372_uid174872 | 1.187 |
| *Listeria* | Listeria_monocytogenes_EGD_uid223288 | 1.185 |
| *Geobacillus* | Geobacillus_kaustophilus_HTA426_uid58227 | 1.179 |
| *Listeria* | Listeria_innocua_Clip11262_uid61567 | 1.178 |
| *Listeria* | Listeria_monocytogenes_SLCC2479_uid175108 | 1.178 |
| *Dickeya* | Dickeya_dadantii_3937_uid52537 | 1.177 |
| *Listeria* | Listeria_monocytogenes_J1816_uid179734 | 1.175 |
| *Bacillus* | Bacillus_thuringiensis_MC28_uid176369 | 1.174 |
| *Mycoplasma* | Mycoplasma_genitalium_M6320_uid173370 | 1.173 |
| *Mycoplasma* | Mycoplasma_genitalium_M6282_uid173371 | 1.173 |
| *Chlorobaculum* | Chlorobaculum_parvum_NCIB_8327_uid59185 | 1.173 |
| *Mycoplasma* | Mycoplasma_gallisepticum_NC96_1596_4_2P_uid172626 | 1.173 |
| *Kinetoplastibacterium* | Candidatus_Kinetoplastibacterium_oncopeltii_TCC290E_uid189840 | 1.171 |
| *Listeria* | Listeria_monocytogenes_J1_220_uid179735 | 1.171 |
| *Geobacillus* | Geobacillus_HH01_uid188479 | 1.169 |
| *Listeria* | Listeria_monocytogenes_HCC23_uid59203 | 1.167 |
| *Listeria* | Listeria_monocytogenes_serotype_4a_L99_uid161953 | 1.166 |
| *Listeria* | Listeria_monocytogenes_M7_uid162131 | 1.165 |
| *Geobacillus* | Geobacillus_JF8_uid215234 | 1.164 |
| *Meiothermus* | Meiothermus_silvanus_DSM_9946_uid49485 | 1.164 |
| *Bacillus* | Bacillus_cereus_FRI_35_uid173403 | 1.163 |
| *Tetragenococcus* | Tetragenococcus_halophilus_uid74441 | 1.162 |
| *Pantoea* | Pantoea_vagans_C9_1_uid49871 | 1.16 |
| *Mycoplasma* | Mycoplasma_genitalium_M2288_uid173372 | 1.158 |
| *Geobacillus* | Geobacillus_Y412MC61_uid41171 | 1.152 |
| *Listeria* | Listeria_monocytogenes_J0161_uid54459 | 1.151 |
| *Geobacillus* | Geobacillus_Y412MC52_uid55381 | 1.147 |
| *Caldicellulosiruptor* | Caldicellulosiruptor_lactoaceticus_6A_uid60575 | 1.146 |
| *Yersinia* | Yersinia_enterocolitica_8081_uid57741 | 1.144 |
| *Bacillus* | Bacillus_cereus_ATCC_10987_uid57673 | 1.144 |
| *Paenibacillus* | Paenibacillus_mucilaginosus_3016_uid89377 | 1.142 |
| *Edwardsiella* | Edwardsiella_ictaluri_93_146_uid59403 | 1.138 |
| *Caldicellulosiruptor* | Caldicellulosiruptor_hydrothermalis_108_uid60157 | 1.137 |
| *Bacillus* | Bacillus_anthracis_CDC_684_uid59303 | 1.137 |
| *Kinetoplastibacterium* | Candidatus_Kinetoplastibacterium_blastocrithidii_TCC012E_uid189752 | 1.136 |
| *Bacillus* | Bacillus_toyonensis_BCT_7112_uid227218 | 1.132 |
| *Paenibacillus* | Paenibacillus_mucilaginosus_KNP414_uid68311 | 1.131 |
| *Kinetoplastibacterium* | Candidatus_Kinetoplastibacterium_galatii_TCC219_uid189751 | 1.13 |
| *Bacillus* | Bacillus_cereus_NC7401_uid82815 | 1.129 |
| *Actinobacillus* | Actinobacillus_succinogenes_130Z_uid58247 | 1.129 |
| *Pelobacter* | Pelobacter_propionicus_DSM_2379_uid58255 | 1.128 |
| *Kinetoplastibacterium* | Candidatus_Kinetoplastibacterium_blastocrithidii__ex_Strigomonas_culicis | 1.127 |
| *Geobacillus* | Geobacillus_C56_T3_uid49467 | 1.126 |
| *Dickeya* | Dickeya_zeae_Ech1591_uid59297 | 1.123 |
| *Bacillus* | Bacillus_cereus_AH187_uid58753 | 1.117 |
| *Ralstonia* | Ralstonia_solanacearum_CFBP2957_uid50545 | 1.117 |
| *Campylobacter* | Campylobacter_curvus_525_92_uid58669 | 1.116 |
| *Paenibacillus* | Paenibacillus_mucilaginosus_K02_uid162117 | 1.116 |
| *Caldicellulosiruptor* | Caldicellulosiruptor_kristjanssonii_177R1B_uid60393 | 1.115 |
| *Mycoplasma* | Mycoplasma_conjunctivae_uid59325 | 1.115 |
| *Yersinia* | Yersinia_enterocolitica_palearctica_Y11_uid162069 | 1.114 |
| *Bacillus* | Bacillus_thuringiensis_serovar_finitimus_YBT_020_uid158875 | 1.114 |
| *Blattabacterium* | Blattabacterium__Blatta_orientalis__Tarazona_uid188115 | 1.111 |
| *Bacillus* | Bacillus_anthracis_Sterne_uid58091 | 1.109 |
| *Deinococcus* | Deinococcus_gobiensis_I_0_uid162509 | 1.109 |
| *Bacillus* | Bacillus_cereus_F837_76_uid83611 | 1.108 |
| *Bacillus* | Bacillus_subtilis_BSn5_uid62463 | 1.107 |
| *Desulfovibrio* | Desulfovibrio_piezophilus_C1TLV30_uid190704 | 1.106 |
| *Bacillus* | Bacillus_anthracis_H9401_uid162021 | 1.106 |
| *Acidovorax* | Acidovorax_ebreus_TPSY_uid59233 | 1.106 |
| *Bacillus* | Bacillus_anthracis_A0248_uid59385 | 1.105 |
| *Bacillus* | Bacillus_anthracis__Ames_Ancestor__uid58083 | 1.105 |
| *Bacillus* | Bacillus_amyloliquefaciens_FZB42_uid58271 | 1.105 |
| *Bacillus* | Bacillus_anthracis_Ames_uid57909 | 1.104 |
| *Bacillus* | Bacillus_thuringiensis_serovar_konkukian_97_27_uid58089 | 1.104 |
| *Alicyclobacillus* | Alicyclobacillus_acidocaldarius_Tc_4_1_uid158681 | 1.101 |
| *Candidatus_Desulforudis* | Candidatus_Desulforudis_audaxviator_MP104C_uid59067 | 1.1 |
| *Bacillus* | Bacillus_amyloliquefaciens_plantarum_AS43_3_uid183682 | 1.098 |
| *Listeria* | Listeria_ivanovii_PAM_55_uid73473 | 1.097 |
| *Bacillus* | Bacillus_thuringiensis_Al_Hakam_uid58795 | 1.097 |
| *Bacillus* | Bacillus_amyloliquefaciens_plantarum_UCMB5113_uid215236 | 1.096 |
| *Erwinia* | Erwinia_amylovora_CFBP1430_uid46839 | 1.095 |
| *Bacillus* | Bacillus_cereus_biovar_anthracis_CI_uid50615 | 1.095 |
| *Caldicellulosiruptor* | Caldicellulosiruptor_bescii_DSM_6725_uid59201 | 1.094 |
| *Erwinia* | Erwinia_amylovora_ATCC_49946_uid46943 | 1.094 |
| *Spiroplasma* | Spiroplasma_apis_B31_uid230613 | 1.093 |
| *Ralstonia* | Ralstonia_solanacearum_PSI07_uid50539 | 1.092 |
| *Bacillus* | Bacillus_amyloliquefaciens_CC178_uid226115 | 1.091 |
| *Bacillus* | Bacillus_cereus_AH820_uid58751 | 1.091 |
| *Bacillus* | Bacillus_cereus_E33L_uid58103 | 1.091 |
| *Ralstonia* | Ralstonia_pickettii_12D_uid58859 | 1.09 |
| *Ralstonia* | Ralstonia_solanacearum_Po82_uid162133 | 1.09 |
| *Bacillus* | Bacillus_amyloliquefaciens_IT_45_uid181617 | 1.09 |
| *Carnobacterium* | Carnobacterium_17_4_uid65789 | 1.089 |
| *Bacillus* | Bacillus_amyloliquefaciens_plantarum_UCMB5036_uid190705 | 1.085 |
| *Bacillus* | Bacillus_thuringiensis_BMB171_uid49135 | 1.084 |
| *Bacillus* | Bacillus_cereus_03BB102_uid59299 | 1.084 |
| *Bacillus* | Bacillus_cereus_Q1_uid58529 | 1.083 |
| *Deinococcus* | Deinococcus_radiodurans_R1_uid57665 | 1.083 |
| *Bacillus* | Bacillus_amyloliquefaciens_DSM_7_uid53535 | 1.078 |
| *Erwinia* | Erwinia_Ejp617_uid159955 | 1.076 |
| *Bacillus* | Bacillus_amyloliquefaciens_LFB112_uid232246 | 1.073 |
| *Bacillus* | Bacillus_weihenstephanensis_KBAB4_uid58315 | 1.07 |
| *Corynebacterium* | Corynebacterium_efficiens_YS_314_uid62905 | 1.07 |
| *Bacillus* | Bacillus_amyloliquefaciens_XH7_uid158881 | 1.069 |
| *Corynebacterium* | Corynebacterium_aurimucosum_ATCC_700975_uid59409 | 1.068 |
| *Dickeya* | Dickeya_dadantii_Ech703_uid59363 | 1.067 |
| *Paenibacillus* | Paenibacillus_larvae_04_309_uid232355 | 1.066 |
| *Bacillus* | Bacillus_amyloliquefaciens_plantarum_CAU_B946_uid84215 | 1.065 |
| *Bacillus* | Bacillus_subtilis_BSP1_uid184010 | 1.063 |
| *Paenibacillus* | Paenibacillus_polymyxa_E681_uid53477 | 1.063 |
| *Bacillus* | Bacillus_amyloliquefaciens_TA208_uid158701 | 1.062 |
| *Paenibacillus* | Paenibacillus_polymyxa_SC2_uid59583 | 1.062 |
| *Ralstonia* | Ralstonia_solanacearum_CMR15_uid227773 | 1.06 |
| *Bacillus* | Bacillus_cereus_ATCC_14579_uid57975 | 1.059 |
| *Bacillus* | Bacillus_cereus_B4264_uid58757 | 1.058 |
| *Erwinia* | Erwinia_pyrifoliae_DSM_12163_uid159693 | 1.058 |
| *Yersinia* | Yersinia_enterocolitica_palearctica_105_5R_r__uid63663 | 1.057 |
| *Bacillus* | Bacillus_amyloliquefaciens_LL3_uid158133 | 1.057 |
| *Geobacillus* | Geobacillus_thermodenitrificans_NG80_2_uid58829 | 1.055 |
| *Thermosediminibacter* | Thermosediminibacter_oceani_DSM_16646_uid51421 | 1.054 |
| *Ralstonia* | Ralstonia_solanacearum_GMI1000_uid57593 | 1.053 |
| *Erwinia* | Erwinia_pyrifoliae_Ep1_96_uid40659 | 1.052 |
| *Bacillus* | Bacillus_thuringiensis_Bt407_uid177931 | 1.052 |
| *Geobacillus* | Geobacillus_WCH70_uid59045 | 1.051 |
| *Bacillus* | Bacillus_subtilis_PY79_uid229877 | 1.049 |
| *Bacillus* | Bacillus_amyloliquefaciens_plantarum_NAU_B3_uid222816 | 1.047 |
| *Bacillus* | Bacillus_thuringiensis_serovar_kurstaki_HD73_uid189188 | 1.046 |
| *Campylobacter* | Campylobacter_jejuni_M1_uid159535 | 1.044 |
| *Bacillus* | Bacillus_cereus_G9842_uid58759 | 1.043 |
| *Erwinia* | Erwinia_tasmaniensis_Et1_99_uid59029 | 1.043 |
| *Acetobacterium* | Acetobacterium_woodii_DSM_1030_uid88073 | 1.041 |
| *Bacillus* | Bacillus_amyloliquefaciens_plantarum_UCMB5033_uid215237 | 1.04 |
| *Bacillus* | Bacillus_subtilis_spizizenii_W23_uid51879 | 1.037 |
| *Bacillus* | Bacillus_subtilis_RO_NN_1_uid158879 | 1.036 |
| *Paenibacillus* | Paenibacillus_polymyxa_M1_uid162159 | 1.034 |
| *Desulfitobacterium* | Desulfitobacterium_hafniense_DCB_2_uid57749 | 1.033 |
| *Pseudoalteromonas* | Pseudoalteromonas_haloplanktis_TAC125_uid58431 | 1.033 |
| *Cupriavidus* | Cupriavidus_necator_N_1_uid68689 | 1.033 |
| *Macrococcus* | Macrococcus_caseolyticus_JCSC5402_uid59003 | 1.032 |
| *Bacillus* | Bacillus_thuringiensis_serovar_IS5056_uid190186 | 1.031 |
| *Bacillus* | Bacillus_thuringiensis_HD_789_uid173860 | 1.031 |
| *Bacillus* | Bacillus_subtilis_XF_1_uid189187 | 1.03 |
| *Ralstonia* | Ralstonia_solanacearum_FQY_4_f_uid194089 | 1.029 |
| *Caldicellulosiruptor* | Caldicellulosiruptor_saccharolyticus_DSM_8903_uid58289 | 1.024 |
| *Bacillus* | Bacillus_amyloliquefaciens_plantarum_YAU_B9601_Y2_uid159001 | 1.024 |
| *Ilyobacter* | Ilyobacter_polytropus_DSM_2926_uid59769 | 1.024 |
| *Capnocytophaga* | Capnocytophaga_ochracea_DSM_7271_uid59197 | 1.023 |
| *Mobiluncus* | Mobiluncus_curtisii_ATCC_43063_uid49695 | 1.02 |
| *Desulfitobacterium* | Desulfitobacterium_dichloroeliminans_LMG_P_21439_uid82555 | 1.019 |
| *Bacillus* | Bacillus_subtilis_BAB_1_uid195461 | 1.018 |
| *Bacillus* | Bacillus_thuringiensis_serovar_chinensis_CT_43_uid158151 | 1.018 |
| *Paenibacillus* | Paenibacillus_polymyxa_CR1_uid231659 | 1.015 |
| *Laribacter* | Laribacter_hongkongensis_HLHK9_uid59265 | 1.015 |
| *Bacillus* | Bacillus_subtilis_6051_HGW_uid193706 | 1.012 |
| *Bacillus* | Bacillus_subtilis_168_uid57675 | 1.011 |
| *Geobacter* | Geobacter_lovleyi_SZ_uid58713 | 1.01 |
| *Rhodothermus* | Rhodothermus_marinus_SG0_5JP17_172_uid72767 | 1.008 |
| *Bacillus* | Bacillus_subtilis_natto_BEST195_uid183001 | 1.008 |
| *Campylobacter* | Campylobacter_jejuni_IA3902_uid159531 | 1.006 |
| *Bacillus* | Bacillus_pumilus_SAFR_032_uid59017 | 1.002 |
| *Bacillus* | Bacillus_subtilis_QB928_uid173926 | 1.001 |
| *Riemerella* | Riemerella_anatipestifer_RA_CH_2_uid186548 | 1.001 |
| *Rhodothermus* | Rhodothermus_marinus_DSM_4252_uid41729 | 1 |
| *Bacillus* | Bacillus_amyloliquefaciens_Y2_uid165195 | 0.996 |
| *Geobacillus* | Geobacillus_Y4_1MC1_uid55779 | 0.993 |
| *Ammonifex* | Ammonifex_degensii_KC4_uid41053 | 0.991 |
| *Ralstonia* | Ralstonia_pickettii_12J_uid58737 | 0.986 |
| *Paenibacillus* | Paenibacillus_JDR_2_uid59021 | 0.986 |
| *Bacillus* | Bacillus_subtilis_spizizenii_TU_B_10_uid73967 | 0.981 |
| *Dickeya* | Dickeya_dadantii_Ech586_uid42519 | 0.98 |
| *Fibrobacter* | Fibrobacter_succinogenes_S85_uid41169 | 0.978 |
| *Thermacetogenium* | Thermacetogenium_phaeum_DSM_12270_uid177811 | 0.975 |
| *Bacillus* | Bacillus_JS_uid162189 | 0.973 |
| *Bacillus* | Bacillus_thuringiensis_YBT_1518_uid229419 | 0.973 |
| *Fibrobacter* | Fibrobacter_succinogenes_S85_uid161919 | 0.972 |
| *Bacillus* | Bacillus_licheniformis_9945A_uid207072 | 0.971 |
| *Geobacillus* | Geobacillus_thermoglucosidasius_C56_YS93_uid48129 | 0.969 |
| *Desulfitobacterium* | Desulfitobacterium_dehalogenans_ATCC_51507_uid82553 | 0.968 |
| *Oceanithermus* | Oceanithermus_profundus_DSM_14977_uid60855 | 0.967 |
| *Acidovorax* | Acidovorax_JS42_uid58427 | 0.965 |
| *Anaeromyxobacter* | Anaeromyxobacter_dehalogenans_2CP_C_uid58135 | 0.965 |
| *Syntrophomonas* | Syntrophomonas_wolfei_Goettingen_uid58179 | 0.964 |
| *Candidatus_Moranella* | Candidatus_Moranella_endobia_PCVAL_uid197215 | 0.963 |
| *Candidatus_Portiera* | Candidatus_Portiera_aleyrodidarum_BT_B_uid176373 | 0.962 |
| *Candidatus_Portiera* | Candidatus_Portiera_aleyrodidarum_BT_QVLC_uid176374 | 0.961 |
| *Bacillus* | Bacillus_thuringiensis_HD_771_uid173374 | 0.961 |
| *Mannheimia* | Mannheimia_haemolytica_USDA_ARS_SAM_185_uid195457 | 0.958 |
| *Bacillus* | Bacillus_selenitireducens_MLS10_uid49513 | 0.957 |
| *Wigglesworthia* | Wigglesworthia_glossinidia_endosymbiont_of_Glossina_brevipalpis_uid57853 | 0.956 |
| *Mycoplasma* | Mycoplasma_hyopneumoniae_J_uid58059 | 0.953 |
| *Bordetella* | Bordetella_parapertussis_18323_uid175569 | 0.951 |
| *Bacillus* | Bacillus_licheniformis_DSM_13___ATCC_14580_uid58199 | 0.95 |
| *Bacillus* | Bacillus_licheniformis_ATCC_14580_uid58097 | 0.95 |
| *Azospirillum* | Azospirillum_lipoferum_4B_uid82343 | 0.948 |
| *Thioalkalivibrio* | Thioalkalivibrio_sulfidophilus_HL_EbGr7_uid59179 | 0.947 |
| *Candidatus_Portiera* | Candidatus_Portiera_aleyrodidarum_BT_B_uid173859 | 0.946 |
| *Mannheimia* | Mannheimia_haemolytica_USMARC_2286_uid213228 | 0.94 |
| *Bacillus* | Bacillus_pseudofirmus_OF4_uid45847 | 0.94 |
| *Deinococcus* | Deinococcus_geothermalis_DSM_11300_uid58275 | 0.939 |
| *Candidatus_Portiera* | Candidatus_Portiera_aleyrodidarum_BT_QVLC_uid175570 | 0.939 |
| *Dichelobacter* | Dichelobacter_nodosus_VCS1703A_uid57643 | 0.938 |
| *Bordetella* | Bordetella_pertussis_Tohama_I_uid57617 | 0.938 |
| *Erwinia* | Erwinia_billingiae_Eb661_uid50547 | 0.932 |
| *Mannheimia* | Mannheimia_haemolytica_D153_uid212303 | 0.931 |
| *Mannheimia* | Mannheimia_haemolytica_USDA_ARS_USMARC_183_uid195458 | 0.93 |
| *Bordetella* | Bordetella_pertussis_CS_uid158859 | 0.93 |
| *Amphibacillus* | Amphibacillus_xylanus_NBRC_15112_uid176453 | 0.929 |
| *Anaeromyxobacter* | Anaeromyxobacter_dehalogenans_2CP_1_uid58989 | 0.926 |
| *Paenibacillus* | Paenibacillus_Y412MC10_uid41127 | 0.925 |
| *Mahella* | Mahella_australiensis_50_1_BON_uid66917 | 0.923 |
| *Buchnera* | Buchnera_aphidicola_Bp__Baizongia_pistaciae__uid57827 | 0.923 |
| *Marinitoga* | Marinitoga_piezophila_KA3_uid81629 | 0.922 |
| *Taylorella* | Taylorella_equigenitalis_MCE9_uid62103 | 0.919 |
| *Halobacteroides* | Halobacteroides_halobius_DSM_5150_uid184862 | 0.918 |
| *Carnobacterium* | Carnobacterium_maltaromaticum_LMA28_uid179370 | 0.918 |
| *Campylobacter* | Campylobacter_lari_RM2100_uid58115 | 0.917 |
| *Clostridium* | Clostridium_stercorarium_DSM_8532_uid186819 | 0.917 |
| *Anaeromyxobacter* | Anaeromyxobacter_K_uid58953 | 0.916 |
| *Treponema* | Treponema_primitia_ZAS_2_uid67367 | 0.914 |
| *Pectobacterium* | Pectobacterium_carotovorum_PC1_uid59295 | 0.914 |
| *Clostridium* | Clostridium_stercorarium_DSM_8532_uid195569 | 0.913 |
| *Cupriavidus* | Cupriavidus_metallidurans_CH34_uid57815 | 0.913 |
| *Bacillus* | Bacillus_atrophaeus_1942_uid59887 | 0.911 |
| *Paenibacillus* | Paenibacillus_terrae_HPL_003_uid82371 | 0.909 |
| *Gallibacterium* | Gallibacterium_anatis_UMN179_uid66567 | 0.908 |
| *Mannheimia* | Mannheimia_haemolytica_M42548_uid198769 | 0.907 |
| *Pantoea* | Pantoea_At_9b_uid55845 | 0.907 |
| *Pectobacterium* | Pectobacterium_carotovorum_PCC21_uid174335 | 0.906 |
| *Riemerella* | Riemerella_anatipestifer_RA_GD_uid162013 | 0.903 |
| *Mycoplasma* | Mycoplasma_hyopneumoniae_7448_uid58039 | 0.902 |
| *Borrelia* | Borrelia_miyamotoi_LB_2001_uid215233 | 0.901 |
| *Rahnella* | Rahnella_aquatilis_CIP_78_65___ATCC_33071_uid86855 | 0.9 |
| *Mycoplasma* | Mycoplasma_hyopneumoniae_232_uid58205 | 0.9 |
| *Micrococcus* | Micrococcus_luteus_NCTC_2665_uid59033 | 0.899 |
| *Bacillus* | Bacillus_megaterium_QM_B1551_uid15862 | 0.897 |
| *Corynebacterium* | Corynebacterium_diphtheriae_HC02_uid84317 | 0.895 |
| *Bacillus* | Bacillus_megaterium_DSM319_uid48371 | 0.894 |
| *Halanaerobium* | Halanaerobium_hydrogeniformans_uid60191 | 0.893 |
| *Bacillus* | Bacillus_megaterium_WSH_002_uid159841 | 0.893 |
| *Spirochaeta* | Spirochaeta_thermophila_DSM_6192_uid53037 | 0.892 |
| *Campylobacter* | Campylobacter_jejuni_NCTC_11168___ATCC_700819_uid57587 | 0.892 |
| *Campylobacter* | Campylobacter_jejuni_NCTC_11168_BN148_uid174152 | 0.892 |
| *Yersinia* | Yersinia_pestis_CO92_uid57621 | 0.891 |
| *Thermincola* | Thermincola_potens_JR_uid48823 | 0.89 |
| *Mycoplasma* | Mycoplasma_arthritidis_158L3_1_uid58005 | 0.888 |
| *Campylobacter* | Campylobacter_jejuni_81116_uid58771 | 0.888 |
| *Mycoplasma* | Mycoplasma_hyopneumoniae_7422_uid212968 | 0.887 |
| *Thiobacillus* | Thiobacillus_denitrificans_ATCC_25259_uid58189 | 0.884 |
| *Riemerella* | Riemerella_anatipestifer_RA_CH_1_uid175469 | 0.883 |
| *Dehalogenimonas* | Dehalogenimonas_lykanthroporepellens_BL_DC_9_uid48131 | 0.88 |
| *Bacillus* | Bacillus_infantis_NRRL_B_14911_uid222804 | 0.88 |
| *Campylobacter* | Campylobacter_jejuni_81_176_uid58503 | 0.879 |
| *Campylobacter* | Campylobacter_jejuni_PT14_uid176499 | 0.879 |
| *Ornithobacterium* | Ornithobacterium_rhinotracheale_DSM_15997_uid168256 | 0.878 |
| *Desulfurispirillum* | Desulfurispirillum_indicum_S5_uid45897 | 0.873 |
| *Yersinia* | Yersinia_pestis_Angola_uid58485 | 0.87 |
| *Candidatus_Moranella* | Candidatus_Moranella_endobia_PCIT_uid68739 | 0.869 |
| *Mannheimia* | Mannheimia_haemolytica_D171_uid212304 | 0.869 |
| *Yersinia* | Yersinia_pestis_Pestoides_F_uid58619 | 0.869 |
| *Campylobacter* | Campylobacter_jejuni_ICDCCJ07001_uid61249 | 0.868 |
| *Borrelia* | Borrelia_burgdorferi_JD1_uid161197 | 0.868 |
| *Corynebacterium* | Corynebacterium_resistens_DSM_45100_uid50555 | 0.868 |
| *Campylobacter* | Campylobacter_jejuni_S3_uid159533 | 0.868 |
| *Deinococcus* | Deinococcus_deserti_VCD115_uid58615 | 0.865 |
| *Bacillus* | Bacillus_1NLA3E_uid81841 | 0.861 |
| *Candidatus_Riesia* | Candidatus_Riesia_pediculicola_USDA_uid46841 | 0.861 |
| *Borrelia* | Borrelia_burgdorferi_N40_uid161241 | 0.861 |
| *Pectobacterium* | Pectobacterium_SCC3193_uid193707 | 0.86 |
| *Campylobacter* | Campylobacter_coli_76339_uid217050 | 0.86 |
| *Borrelia* | Borrelia_burgdorferi_B31_uid57581 | 0.859 |
| *Tepidanaerobacter* | Tepidanaerobacter_Re1_uid66873 | 0.859 |
| *Yersinia* | Yersinia_pestis_biovar_Microtus_91001_uid58037 | 0.858 |
| *Pectobacterium* | Pectobacterium_atrosepticum_SCRI1043_uid57957 | 0.858 |
| *Bordetella* | Bordetella_parapertussis_Bpp5_uid177516 | 0.857 |
| *Bordetella* | Bordetella_parapertussis_12822_uid57615 | 0.857 |
| *Tepidanaerobacter* | Tepidanaerobacter_acetatoxydans_Re1_uid184827 | 0.857 |
| *Yersinia* | Yersinia_pestis_KIM_10_uid57875 | 0.857 |
| *Borrelia* | Borrelia_afzelii_PKo_uid159867 | 0.856 |
| *Robiginitalea* | Robiginitalea_biformata_HTCC2501_uid58285 | 0.855 |
| *Campylobacter* | Campylobacter_jejuni_4031_uid222817 | 0.855 |
| *Borrelia* | Borrelia_burgdorferi_CA382_uid214794 | 0.854 |
| *Azospirillum* | Azospirillum_B510_uid46085 | 0.851 |
| *Desulfurivibrio* | Desulfurivibrio_alkaliphilus_AHT2_uid49487 | 0.85 |
| *Yersinia* | Yersinia_pestis_A1122_uid158119 | 0.849 |
| *Borrelia* | Borrelia_garinii_BgVir_uid162165 | 0.848 |
| *Borrelia* | Borrelia_afzelii_PKo_uid58653 | 0.848 |
| *Arcobacter* | Arcobacter_butzleri_7h1h_uid200766 | 0.848 |
| *Pantoea* | Pantoea_ananatis_AJ13355_uid162073 | 0.847 |
| *Campylobacter* | Campylobacter_jejuni_00_2425_uid219359 | 0.846 |
| *Wigglesworthia* | Wigglesworthia_glossinidia_endosymbiont_of_Glossina_morsitans__Yale_colony | 0.846 |
| *Yersinia* | Yersinia_pestis_D182038_uid158073 | 0.845 |
| *Yersinia* | Yersinia_pseudotuberculosis_IP_32953_uid58157 | 0.845 |
| *Campylobacter* | Campylobacter_coli_15_537360_uid226113 | 0.843 |
| *Yersinia* | Yersinia_pseudotuberculosis_PB1__uid59153 | 0.842 |
| *Pantoea* | Pantoea_ananatis_uid86861 | 0.841 |
| *Yersinia* | Yersinia_pestis_D106004_uid158071 | 0.841 |
| *Bordetella* | Bordetella_avium_197N_uid61563 | 0.84 |
| *Bordetella* | Bordetella_bronchiseptica_253_uid178913 | 0.84 |
| *Corynebacterium* | Corynebacterium_diphtheriae_BH8_uid84311 | 0.835 |
| *Borrelia* | Borrelia_afzelii_HLJ01_uid177930 | 0.834 |
| *Acidovorax* | Acidovorax_avenae_ATCC_19860_uid42497 | 0.834 |
| *Aeromonas* | Aeromonas_hydrophila_ATCC_7966_uid58617 | 0.833 |
| *Treponema* | Treponema_denticola_ATCC_35405_uid57583 | 0.833 |
| *Borrelia* | Borrelia_crocidurae_Achema_uid162335 | 0.833 |
| *Yersinia* | Yersinia_pestis_Antiqua_uid58607 | 0.833 |
| *Pantoea* | Pantoea_ananatis_LMG_20103_uid46807 | 0.832 |
| *Rhodospirillum* | Rhodospirillum_centenum_SW_uid58805 | 0.832 |
| *Desulfobulbus* | Desulfobulbus_propionicus_DSM_2032_uid62265 | 0.831 |
| *Campylobacter* | Campylobacter_jejuni_RM1221_uid57899 | 0.83 |
| *Spirochaeta* | Spirochaeta_thermophila_DSM_6578_uid162041 | 0.83 |
| *Borrelia* | Borrelia_bissettii_DN127_uid71231 | 0.829 |
| *Mycoplasma* | Mycoplasma_hyopneumoniae_168_L_uid205052 | 0.824 |
| *Bordetella* | Bordetella_bronchiseptica_MO149_uid177517 | 0.824 |
| *Geobacter* | Geobacter_uraniireducens_Rf4_uid58475 | 0.823 |
| *Campylobacter* | Campylobacter_coli_CVM_N29710_uid219322 | 0.822 |
| *Rhodospirillum* | Rhodospirillum_rubrum_ATCC_11170_uid57655 | 0.821 |
| *Rhodospirillum* | Rhodospirillum_rubrum_F11_uid162149 | 0.821 |
| *Borrelia* | Borrelia_garinii_PBi_uid58125 | 0.821 |
| *Acidovorax* | Acidovorax_citrulli_AAC00_1_uid58429 | 0.82 |
| *Azospirillum* | Azospirillum_brasilense_Sp245_uid162161 | 0.82 |
| *Pectobacterium* | Pectobacterium_wasabiae_WPP163_uid41297 | 0.819 |
| *Pantoea* | Pantoea_ananatis_PA13_uid162181 | 0.819 |
| *Bordetella* | Bordetella_bronchiseptica_RB50_uid57613 | 0.819 |
| *Treponema* | Treponema_azotonutricium_ZAS_9_uid67365 | 0.818 |
| *Mannheimia* | Mannheimia_haemolytica_D174_uid212305 | 0.818 |
| *Rhodospirillum* | Rhodospirillum_photometricum_uid159003 | 0.816 |
| *Borrelia* | Borrelia_recurrentis_A1_uid58793 | 0.814 |
| *Pelobacter* | Pelobacter_carbinolicus_DSM_2380_uid58241 | 0.814 |
| *Borrelia* | Borrelia_duttonii_Ly_uid58791 | 0.813 |
| *Corynebacterium* | Corynebacterium_diphtheriae_INCA_402_uid83605 | 0.813 |
| *Yersinia* | Yersinia_pestis_biovar_Medievalis_Harbin_35_uid158537 | 0.811 |
| *Rahnella* | Rahnella_aquatilis_HX2_uid158049 | 0.811 |
| *Yersinia* | Yersinia_pestis_Nepal516_uid58609 | 0.807 |
| *Deinococcus* | Deinococcus_maricopensis_DSM_21211_uid62225 | 0.805 |
| *Corynebacterium* | Corynebacterium_diphtheriae_HC01_uid84297 | 0.803 |
| *Rickettsia* | Rickettsia_typhi_Wilmington_uid58063 | 0.802 |
| *Corynebacterium* | Corynebacterium_diphtheriae_241_uid83607 | 0.802 |
| *Rickettsia* | Rickettsia_typhi_TH1527_uid158161 | 0.801 |
| *Rickettsia* | Rickettsia_typhi_B9991CWPP_uid158357 | 0.801 |
| *Aeromonas* | Aeromonas_hydrophila_ML09_119_uid205540 | 0.801 |
| *Arcobacter* | Arcobacter_butzleri_ED_1_uid158699 | 0.799 |
| *Bacillus* | Bacillus_cellulosilyticus_DSM_2522_uid43329 | 0.799 |
| *Corynebacterium* | Corynebacterium_diphtheriae_C7__beta__uid84313 | 0.797 |
| *Corynebacterium* | Corynebacterium_diphtheriae_NCTC_13129_uid57691 | 0.796 |
| *Corynebacterium* | Corynebacterium_diphtheriae_CDCE_8392_uid84295 | 0.795 |
| *Thiomonas* | Thiomonas_intermedia_K12_uid48825 | 0.795 |
| *Yersinia* | Yersinia_pestis_Z176003_uid47317 | 0.794 |
| *Corynebacterium* | Corynebacterium_diphtheriae_VA01_uid84305 | 0.793 |
| *Geobacter* | Geobacter_FRC_32_uid58543 | 0.791 |
| *Corynebacterium* | Corynebacterium_diphtheriae_HC04_uid84301 | 0.79 |
| *Rhodobacter* | Rhodobacter_sphaeroides_ATCC_17029_uid58449 | 0.79 |
| *Corynebacterium* | Corynebacterium_diphtheriae_HC03_uid84299 | 0.79 |
| *Corynebacterium* | Corynebacterium_diphtheriae_PW8_uid84303 | 0.789 |
| *Mycoplasma* | Mycoplasma_hyopneumoniae_168_uid162053 | 0.787 |
| *Anaeromyxobacter* | Anaeromyxobacter_Fw109_5_uid58755 | 0.786 |
| *Borrelia* | Borrelia_burgdorferi_ZS7_uid59429 | 0.784 |
| *Corynebacterium* | Corynebacterium_diphtheriae_31A_uid84309 | 0.783 |
| *Bordetella* | Bordetella_petrii_uid61631 | 0.78 |
| *Dehalococcoides* | Dehalococcoides_mccartyi_BTF08_uid190183 | 0.777 |
| *Oenococcus* | Oenococcus_oeni_PSU_1_uid59417 | 0.775 |
| *Desulfococcus* | Desulfococcus_oleovorans_Hxd3_uid58777 | 0.775 |
| *Borrelia* | Borrelia_turicatae_91E135_uid58311 | 0.775 |
| *Rahnella* | Rahnella_Y9602_uid62715 | 0.772 |
| *Candidatus_Blochmannia* | Candidatus_Blochmannia_floridanus_uid57999 | 0.77 |
| *Nitratifractor* | Nitratifractor_salsuginis_DSM_16511_uid62183 | 0.769 |
| *Desulfohalobium* | Desulfohalobium_retbaense_DSM_5692_uid59183 | 0.768 |
| *Campylobacter* | Campylobacter_jejuni_00_2538_uid219325 | 0.767 |
| *Borrelia* | Borrelia_garinii_NMJW1_uid177081 | 0.766 |
| *Aeromonas* | Aeromonas_veronii_B565_uid66323 | 0.766 |
| *Oceanimonas* | Oceanimonas_GK1_uid81627 | 0.762 |
| *Arcobacter* | Arcobacter_butzleri_RM4018_uid58557 | 0.761 |
| *Taylorella* | Taylorella_asinigenitalis_MCE3_uid73771 | 0.758 |
| *Yersinia* | Yersinia_pseudotuberculosis_YPIII_uid59151 | 0.757 |
| *Yersinia* | Yersinia_pseudotuberculosis_IP_31758_uid58487 | 0.757 |
| *Campylobacter* | Campylobacter_jejuni_doylei_269_97_uid58671 | 0.753 |
| *Bibersteinia* | Bibersteinia_trehalosi_192_uid193709 | 0.752 |
| *Streptomyces* | Streptomyces_albus_J1074_uid196849 | 0.751 |
| *Corynebacterium* | Corynebacterium_kroppenstedtii_DSM_44385_uid59411 | 0.751 |
| *Capnocytophaga* | Capnocytophaga_canimorsus_Cc5_uid70727 | 0.751 |
| *Rickettsia* | Rickettsia_prowazekii_Katsinyian_uid158055 | 0.75 |
| *Rickettsia* | Rickettsia_prowazekii_BuV67_CWPP_uid158063 | 0.749 |
| *Rickettsia* | Rickettsia_prowazekii_Rp22_uid161945 | 0.749 |
| *Rickettsia* | Rickettsia_prowazekii_NMRC_Madrid_E_uid196850 | 0.749 |
| *Agrobacterium* | Agrobacterium_fabrum_C58_uid57865 | 0.748 |
| *Rickettsia* | Rickettsia_prowazekii_Dachau_uid158057 | 0.747 |
| *Rickettsia* | Rickettsia_prowazekii_Chernikova_uid158053 | 0.747 |
| *Rickettsia* | Rickettsia_prowazekii_Breinl_uid196851 | 0.744 |
| *Meiothermus* | Meiothermus_ruber_DSM_1279_uid198526 | 0.742 |
| *Thiomonas* | Thiomonas_3As_uid178369 | 0.742 |
| *Meiothermus* | Meiothermus_ruber_DSM_1279_uid46661 | 0.741 |
| *Rickettsia* | Rickettsia_conorii_Malish_7_uid57633 | 0.74 |
| *Rickettsia* | Rickettsia_prowazekii_Madrid_E_uid61565 | 0.74 |
| *Agrobacterium* | Agrobacterium_H13_3_uid63403 | 0.74 |
| *Taylorella* | Taylorella_equigenitalis_ATCC_35865_uid170255 | 0.737 |
| *Synechococcus* | Synechococcus_JA_3_3Ab_uid58535 | 0.737 |
| *Rickettsia* | Rickettsia_prowazekii_GvV257_uid158051 | 0.737 |
| *Rickettsia* | Rickettsia_prowazekii_RpGvF24_uid158065 | 0.734 |
| *Solibacillus* | Solibacillus_silvestris_StLB046_uid168516 | 0.73 |
| *Aerococcus* | Aerococcus_urinae_ACS_120_V_Col10a_uid64757 | 0.73 |
| *Acidovorax* | Acidovorax_KKS102_uid176500 | 0.726 |
| *Streptomyces* | Streptomyces_cattleya_NRRL_8057___DSM_46488_uid77117 | 0.726 |
| *Treponema* | Treponema_pedis_T_A4_uid215715 | 0.726 |
| *Streptomyces* | Streptomyces_cattleya_NRRL_8057___DSM_46488_uid162187 | 0.724 |
| *Rickettsia* | Rickettsia_canadensis_McKiel_uid58159 | 0.721 |
| *Rickettsia* | Rickettsia_canadensis_CA410_uid88063 | 0.719 |
| *Rickettsia* | Rickettsia_slovaca_13_B_uid82369 | 0.718 |
| *Rickettsia* | Rickettsia_slovaca_D_CWPP_uid158159 | 0.718 |
| *Rhodobacter* | Rhodobacter_sphaeroides_2_4_1_uid57653 | 0.717 |
| *Halorhodospira* | Halorhodospira_halophila_SL1_uid58473 | 0.715 |
| *Rickettsia* | Rickettsia_felis_URRWXCal2_uid58331 | 0.713 |
| *Oceanobacillus* | Oceanobacillus_iheyensis_HTE831_uid57867 | 0.712 |
| *Rickettsia* | Rickettsia_africae_ESF_5_uid58799 | 0.711 |
| *Francisella* | Francisella_tularensis_TIGB03_uid89379 | 0.711 |
| *Carboxydothermus* | Carboxydothermus_hydrogenoformans_Z_2901_uid57821 | 0.71 |
| *Rhodobacter* | Rhodobacter_sphaeroides_KD131_uid59277 | 0.71 |
| *Micavibrio* | Micavibrio_aeruginosavorus_ARL_13_uid73585 | 0.708 |
| *Brucella* | Brucella_canis_HSK_A52141_uid83613 | 0.707 |
| *Francisella* | Francisella_tularensis_WY96_3418_uid58811 | 0.707 |
| *Brucella* | Brucella_ovis_ATCC_25840_uid58113 | 0.706 |
| *Allochromatium* | Allochromatium_vinosum_DSM_180_uid46083 | 0.705 |
| *Micavibrio* | Micavibrio_EPB_uid194120 | 0.704 |
| *Brucella* | Brucella_canis_ATCC_23365_uid59009 | 0.704 |
| *Polaromonas* | Polaromonas_naphthalenivorans_CJ2_uid58273 | 0.703 |
| *Rickettsia* | Rickettsia_japonica_YH_uid73963 | 0.701 |
| *Francisella* | Francisella_tularensis_holarctica_LVS_uid58595 | 0.7 |
| *Azoarcus* | Azoarcus_BH72_uid61603 | 0.7 |
| *Thioalkalivibrio* | Thioalkalivibrio_K90mix_uid46181 | 0.7 |
| *Francisella* | Francisella_tularensis_FSC198_uid58693 | 0.699 |
| *Francisella* | Francisella_tularensis_NE061598_uid161973 | 0.699 |
| *Francisella* | Francisella_tularensis_TI0902_uid89373 | 0.699 |
| *Francisella* | Francisella_tularensis_SCHU_S4_uid57589 | 0.699 |
| *Rickettsia* | Rickettsia_parkeri_Portsmouth_uid158045 | 0.699 |
| *Francisella* | Francisella_tularensis_mediasiatica_FSC147_uid58939 | 0.699 |
| *Francisella* | Francisella_tularensis_holarctica_F92_uid181998 | 0.699 |
| *Campylobacter* | Campylobacter_03_427_uid226993 | 0.697 |
| *Rickettsia* | Rickettsia_rickettsii_Brazil_uid88069 | 0.697 |
| *Rickettsia* | Rickettsia_akari_Hartford_uid58161 | 0.697 |
| *Francisella* | Francisella_tularensis_holarctica_FTNF002_00_uid58999 | 0.697 |
| *Brucella* | Brucella_abortus_A13334_uid83615 | 0.697 |
| *Borrelia* | Borrelia_hermsii_DAH_uid59225 | 0.697 |
| *Brucella* | Brucella_suis_1330_uid159871 | 0.696 |
| *Brucella* | Brucella_suis_1330_uid57927 | 0.696 |
| *Francisella* | Francisella_tularensis_holarctica_FSC200_uid54341 | 0.696 |
| *Brucella* | Brucella_suis_VBI22_uid83617 | 0.696 |
| *Brucella* | Brucella_microti_CCM_4915_uid59319 | 0.695 |
| *Acetohalobium* | Acetohalobium_arabaticum_DSM_5501_uid51423 | 0.695 |
| *Corynebacterium* | Corynebacterium_glutamicum_MB001_uid214793 | 0.695 |
| *Rickettsia* | Rickettsia_peacockii_Rustic_uid59301 | 0.695 |
| *Brucella* | Brucella_abortus_S19_uid58873 | 0.694 |
| *Brucella* | Brucella_abortus_bv__1_9_941_uid58019 | 0.694 |
| *Francisella* | Francisella_tularensis_holarctica_OSU18_uid58687 | 0.694 |
| *Rickettsia* | Rickettsia_massiliae_AZT80_uid86751 | 0.693 |
| *Bacillus* | Bacillus_halodurans_C_125_uid57791 | 0.692 |
| *Brucella* | Brucella_melitensis_biovar_Abortus_2308_uid62937 | 0.692 |
| *Providencia* | Providencia_stuartii_MRSN_2154_uid162193 | 0.692 |
| *Brucella* | Brucella_ceti_TE28753_12_uid229879 | 0.69 |
| *Kocuria* | Kocuria_rhizophila_DC2201_uid59099 | 0.689 |
| *Rickettsia* | Rickettsia_rickettsii__Sheila_Smith__uid58027 | 0.688 |
| *Sodalis* | Sodalis_glossinidius__morsitans__uid58553 | 0.688 |
| *Brucella* | Brucella_ceti_TE10759_12_uid229880 | 0.688 |
| *Brucella* | Brucella_melitensis_bv__1_16M_uid57735 | 0.688 |
| *Campylobacter* | Campylobacter_fetus_82_40_uid58545 | 0.686 |
| *Rickettsia* | Rickettsia_rhipicephali_3_7_female6_CWPP_uid156977 | 0.685 |
| *Azoarcus* | Azoarcus_KH32C_uid193704 | 0.685 |
| *Francisella* | Francisella_cf__novicida_3523_uid162107 | 0.685 |
| *Desulfosporosinus* | Desulfosporosinus_meridiei_DSM_13257_uid75097 | 0.685 |
| *Variovorax* | Variovorax_paradoxus_S110_uid59437 | 0.684 |
| *Rubrivivax* | Rubrivivax_gelatinosus_IL144_uid158163 | 0.684 |
| *Flavobacterium* | Flavobacterium_indicum_GPTSA100_9_uid157999 | 0.683 |
| *Brucella* | Brucella_melitensis_ATCC_23457_uid59241 | 0.683 |
| *Rickettsia* | Rickettsia_rickettsii_Arizona_uid86655 | 0.683 |
| *Brucella* | Brucella_melitensis_M28_uid158857 | 0.683 |
| *Magnetospirillum* | Magnetospirillum_magneticum_AMB_1_uid58527 | 0.683 |
| *Rickettsia* | Rickettsia_rickettsii_Iowa_uid58961 | 0.682 |
| *Brucella* | Brucella_melitensis_M5_90_uid158855 | 0.682 |
| *Francisella* | Francisella_cf__novicida_Fx1_uid162105 | 0.682 |
| *Rickettsia* | Rickettsia_rickettsii_Colombia_uid86653 | 0.682 |
| *Rickettsia* | Rickettsia_montanensis_OSU_85_930_uid158043 | 0.682 |
| *Bacillus* | Bacillus_clausii_KSM_K16_uid58237 | 0.681 |
| *Rickettsia* | Rickettsia_rickettsii_Hauke_uid86659 | 0.681 |
| *Rickettsia* | Rickettsia_rickettsii_Hino_uid86657 | 0.681 |
| *Rhodobacter* | Rhodobacter_sphaeroides_ATCC_17025_uid58451 | 0.681 |
| *Rickettsia* | Rickettsia_philipii_364D_uid89383 | 0.681 |
| *Rhizobium* | Rhizobium_etli_CIAT_652_uid59115 | 0.681 |
| *Herbaspirillum* | Herbaspirillum_seropedicae_SmR1_uid50427 | 0.681 |
| *Candidatus_Blochmannia* | Candidatus_Blochmannia_vafer_BVAF_uid62083 | 0.681 |
| *Francisella* | Francisella_philomiragia_ATCC_25017_uid59105 | 0.681 |
| *Rickettsia* | Rickettsia_rickettsii_Hlp_2_uid88067 | 0.681 |
| *Rhizobium* | Rhizobium_tropici_CIAT_899_uid185179 | 0.679 |
| *Brucella* | Brucella_pinnipedialis_B2_94_uid71131 | 0.679 |
| *Nautilia* | Nautilia_profundicola_AmH_uid59345 | 0.678 |
| *Corynebacterium* | Corynebacterium_pseudotuberculosis_1002_uid159677 | 0.678 |
| *Brucella* | Brucella_melitensis_NI_uid158853 | 0.678 |
| *Corynebacterium* | Corynebacterium_pseudotuberculosis_3_99_5_uid83609 | 0.678 |
| *Corynebacterium* | Corynebacterium_pseudotuberculosis_FRC41_uid50585 | 0.677 |
| *Arcanobacterium* | Arcanobacterium_haemolyticum_DSM_20595_uid49489 | 0.677 |
| *Chromobacterium* | Chromobacterium_violaceum_ATCC_12472_uid58001 | 0.676 |
| *Campylobacter* | Campylobacter_jejuni_00_2426_uid219324 | 0.676 |
| *Paracoccus* | Paracoccus_denitrificans_PD1222_uid58187 | 0.676 |
| *Corynebacterium* | Corynebacterium_pseudotuberculosis_Cp162_uid168258 | 0.675 |
| *Corynebacterium* | Corynebacterium_pseudotuberculosis_C231_uid159675 | 0.675 |
| *Rickettsia* | Rickettsia_heilongjiangensis_054_uid70839 | 0.674 |
| *Francisella* | Francisella_novicida_U112_uid58499 | 0.674 |
| *Rhizobium* | Rhizobium_etli_bv__mimosae_Mim1_uid213896 | 0.674 |
| *Corynebacterium* | Corynebacterium_pseudotuberculosis_PAT10_uid159671 | 0.673 |
| *Corynebacterium* | Corynebacterium_pseudotuberculosis_31_uid162167 | 0.673 |
| *Francisella* | Francisella_noatunensis_orientalis_LADL_07_285A_uid231515 | 0.672 |
| *Corynebacterium* | Corynebacterium_pseudotuberculosis_42_02_A_uid159669 | 0.672 |
| *Corynebacterium* | Corynebacterium_pseudotuberculosis_I19_uid159673 | 0.672 |
| *Corynebacterium* | Corynebacterium_pseudotuberculosis_P54B96_uid157909 | 0.672 |
| *Sideroxydans* | Sideroxydans_lithotrophicus_ES_1_uid46801 | 0.672 |
| *Riemerella* | Riemerella_anatipestifer_ATCC_11845___DSM_15868_uid60727 | 0.668 |
| *Corynebacterium* | Corynebacterium_pseudotuberculosis_267_uid162175 | 0.668 |
| *Riemerella* | Riemerella_anatipestifer_ATCC_11845___DSM_15868_uid159857 | 0.667 |
| *Corynebacterium* | Corynebacterium_pseudotuberculosis_1_06_A_uid159665 | 0.667 |
| *Exiguobacterium* | Exiguobacterium_sibiricum_255_15_uid58053 | 0.667 |
| *Thauera* | Thauera_MZ1T_uid58987 | 0.666 |
| *Rickettsia* | Rickettsia_australis_Cutlack_uid158039 | 0.666 |
| *Corynebacterium* | Corynebacterium_pseudotuberculosis_258_uid167260 | 0.663 |
| *Sphaerobacter* | Sphaerobacter_thermophilus_DSM_20745_uid41997 | 0.663 |
| *Vibrio* | Vibrio_cholerae_LMA3984_4_uid159541 | 0.663 |
| *Candidatus_Pelagibacter* | Candidatus_Pelagibacter_ubique_HTCC1062_uid58401 | 0.662 |
| *Corynebacterium* | Corynebacterium_pseudotuberculosis_CIP_52_97_uid159667 | 0.662 |
| *Herminiimonas* | Herminiimonas_arsenicoxydans_uid58291 | 0.661 |
| *Exiguobacterium* | Exiguobacterium_antarcticum_B7_uid176125 | 0.66 |
| *Rhizobium* | Rhizobium_etli_CFN_42_uid58377 | 0.66 |
| *Corynebacterium* | Corynebacterium_callunae_DSM_20147_uid193714 | 0.659 |
| *Corynebacterium* | Corynebacterium_ulcerans_809_uid159659 | 0.658 |
| *Rhizobium* | Rhizobium_IRBG74_uid222820 | 0.658 |
| *Corynebacterium* | Corynebacterium_pseudotuberculosis_316_uid89381 | 0.657 |
| *Brucella* | Brucella_suis_ATCC_23445_uid59015 | 0.656 |
| *Rickettsia* | Rickettsia_massiliae_MTU5_uid58801 | 0.656 |
| *Campylobacter* | Campylobacter_jejuni_00_2544_uid219326 | 0.656 |
| *Aeromonas* | Aeromonas_salmonicida_A449_uid58631 | 0.654 |
| *Corynebacterium* | Corynebacterium_glutamicum_ATCC_13032_uid61611 | 0.654 |
| *Thermosipho* | Thermosipho_africanus_TCF52B_uid59095 | 0.654 |
| *Rhizobium* | Rhizobium_leguminosarum_bv__trifolii_WSM2304_uid58997 | 0.654 |
| *Rhizobium* | Rhizobium_leguminosarum_bv__trifolii_WSM1325_uid58991 | 0.652 |
| *Corynebacterium* | Corynebacterium_ulcerans_0102_uid169879 | 0.652 |
| *Vibrio* | Vibrio_Ex25_uid41601 | 0.65 |
| *Arthrobacter* | Arthrobacter_phenanthrenivorans_Sphe3_uid63629 | 0.65 |
| *Corynebacterium* | Corynebacterium_glutamicum_ATCC_13032_uid57905 | 0.649 |
| *Corynebacterium* | Corynebacterium_glutamicum_ATCC_13032_uid193708 | 0.647 |
| *Alkalilimnicola* | Alkalilimnicola_ehrlichii_MLHE_1_uid58467 | 0.647 |
| *Synechococcus* | Synechococcus_CC9605_uid58319 | 0.646 |
| *Spiribacter* | Spiribacter_UAH_SP71_uid226111 | 0.646 |
| *Cyanobium* | Cyanobium_gracile_PCC_6307_uid182931 | 0.646 |
| *Corynebacterium* | Corynebacterium_glutamicum_R_uid58897 | 0.644 |
| *Comamonas* | Comamonas_testosteroni_CNB_2_uid62961 | 0.644 |
| *Rhodobacter* | Rhodobacter_capsulatus_SB_1003_uid47509 | 0.644 |
| *Synechococcus* | Synechococcus_WH_8102_uid61581 | 0.644 |
| *Mycoplasma* | Mycoplasma_parvum_Indiana_uid223379 | 0.643 |
| *Synechococcus* | Synechococcus_WH_7803_uid61607 | 0.642 |
| *Chlorobium* | Chlorobium_phaeovibrioides_DSM_265_uid58129 | 0.642 |
| *Morganella* | Morganella_morganii_KT_uid180867 | 0.641 |
| *Pseudoxanthomonas* | Pseudoxanthomonas_suwonensis_11_1_uid62105 | 0.64 |
| *Pedobacter* | Pedobacter_saltans_DSM_12145_uid61349 | 0.64 |
| *Vibrio* | Vibrio_cholerae_O1_biovar_El_Tor_N16961_uid57623 | 0.64 |
| *Corynebacterium* | Corynebacterium_ulcerans_BR_AD22_uid68291 | 0.639 |
| *Corynebacterium* | Corynebacterium_glutamicum_SCgG2_uid207286 | 0.639 |
| *Sinorhizobium* | Sinorhizobium_meliloti_GR4_uid184823 | 0.639 |
| *Brachybacterium* | Brachybacterium_faecium_DSM_4810_uid58649 | 0.637 |
| *Vibrio* | Vibrio_parahaemolyticus_BB22OP_uid184822 | 0.637 |
| *Corynebacterium* | Corynebacterium_glutamicum_SCgG1_uid207285 | 0.636 |
| *Vibrio* | Vibrio_cholerae_M66_2_uid59355 | 0.635 |
| *Acholeplasma* | Acholeplasma_palmae_J233_uid222824 | 0.635 |
| *Vibrio* | Vibrio_alginolyticus_NBRC_15630___ATCC_17749_uid199933 | 0.635 |
| *Variovorax* | Variovorax_paradoxus_B4_uid218005 | 0.634 |
| *Sphaerochaeta* | Sphaerochaeta_pleomorpha_Grapes_uid82365 | 0.634 |
| *Rhizobium* | Rhizobium_leguminosarum_bv__viciae_3841_uid57955 | 0.634 |
| *Candidatus_Blochmannia* | Candidatus_Blochmannia_pennsylvanicus_BPEN_uid58329 | 0.634 |
| *Sinorhizobium* | Sinorhizobium_meliloti_BL225C_uid52605 | 0.633 |
| *Vibrio* | Vibrio_parahaemolyticus_RIMD_2210633_uid57969 | 0.633 |
| *Halothermothrix* | Halothermothrix_orenii_H_168_uid58585 | 0.633 |
| *Sinorhizobium* | Sinorhizobium_meliloti_1021_uid57603 | 0.633 |
| *Deinococcus* | Deinococcus_peraridilitoris_DSM_19664_uid183485 | 0.633 |
| *Arthrobacter* | Arthrobacter_chlorophenolicus_A6_uid58969 | 0.632 |
| *Vibrio* | Vibrio_cholerae_IEC224_uid89389 | 0.632 |
| *Streptomyces* | Streptomyces_SirexAA_E_uid72627 | 0.632 |
| *Vibrio* | Vibrio_cholerae_O395_uid58425 | 0.632 |
| *Sinorhizobium* | Sinorhizobium_meliloti_2011_uid193772 | 0.63 |
| *Vibrio* | Vibrio_cholerae_O395_uid159869 | 0.629 |
| *Streptomyces* | Streptomyces_fulvissimus_DSM_40593_uid201038 | 0.629 |
| *Vibrio* | Vibrio_cholerae_O1_2010EL_1786_uid78933 | 0.629 |
| *Streptomyces* | Streptomyces_coelicolor_A3_2__uid57801 | 0.628 |
| *Sinorhizobium* | Sinorhizobium_meliloti_Rm41_uid176372 | 0.628 |
| *Sulfurihydrogenibium* | Sulfurihydrogenibium_YO3AOP1_uid58855 | 0.627 |
| *Methylobacterium* | Methylobacterium_extorquens_AM1_uid57605 | 0.626 |
| *Gluconobacter* | Gluconobacter_oxydans_621H_uid58239 | 0.625 |
| *Streptomyces* | Streptomyces_collinus_Tu_365_uid214429 | 0.625 |
| *Methylovorus* | Methylovorus_MP688_uid60723 | 0.625 |
| *Spirochaeta* | Spirochaeta_africana_DSM_8902_uid81779 | 0.625 |
| *Kyrpidia* | Kyrpidia_tusciae_DSM_2912_uid48361 | 0.625 |
| *Synechococcus* | Synechococcus_RCC307_uid61609 | 0.622 |
| *Arcobacter* | Arcobacter_L_uid158135 | 0.621 |
| *Acidithiobacillus* | Acidithiobacillus_ferrooxidans_ATCC_53993_uid58613 | 0.62 |
| *Flavobacterium* | Flavobacterium_psychrophilum_JIP02_86_uid61627 | 0.619 |
| *Desulfosporosinus* | Desulfosporosinus_orientis_DSM_765_uid82939 | 0.618 |
| *Rickettsia* | Candidatus_Rickettsia_amblyommii_GAT_30V_uid156845 | 0.617 |
| *Vibrio* | Vibrio_parahaemolyticus_O1_K33_CDC_K4557_uid212977 | 0.617 |
| *Thermosipho* | Thermosipho_melanesiensis_BI429_uid58683 | 0.617 |
| *Treponema* | Treponema_pallidum_Nichols_uid57585 | 0.614 |
| *Treponema* | Treponema_pallidum_Chicago_uid159543 | 0.613 |
| *Treponema* | Treponema_pallidum_SS14_uid58977 | 0.613 |
| *Treponema* | Treponema_pallidum_Nichols_uid208669 | 0.613 |
| *Vibrio* | Vibrio_vulnificus_MO6_24_O_uid62243 | 0.613 |
| *Treponema* | Treponema_pallidum_DAL_1_uid87065 | 0.613 |
| *Treponema* | Treponema_pallidum_Mexico_A_uid176920 | 0.613 |
| *Francisella* | Francisella_TX077308_uid68321 | 0.612 |
| *Vibrio* | Vibrio_furnissii_NCTC_11218_uid82347 | 0.612 |
| *Magnetospirillum* | Magnetospirillum_gryphiswaldense_MSR_1_uid232249 | 0.611 |
| *Synechococcus* | Synechococcus_JA_2_3B_a_2_13__uid58537 | 0.611 |
| *Treponema* | Treponema_pallidum_pertenue_SamoaD_uid87069 | 0.611 |
| *Treponema* | Treponema_pallidum_pertenue_Gauthier_uid87067 | 0.611 |
| *Treponema* | Treponema_pallidum_pertenue_CDC2_uid87051 | 0.611 |
| *Vibrio* | Vibrio_cholerae_MJ_1236_uid59387 | 0.61 |
| *Streptomyces* | Streptomyces_flavogriseus_ATCC_33331_uid40839 | 0.609 |
| *Methylobacterium* | Methylobacterium_populi_BJ001_uid58937 | 0.609 |
| *Treponema* | Treponema_pallidum_Fribourg_Blanc_uid201428 | 0.608 |
| *Exiguobacterium* | Exiguobacterium_AT1b_uid59093 | 0.608 |
| *Acidithiobacillus* | Acidithiobacillus_caldus_SM_1_uid70791 | 0.607 |
| *Sinorhizobium* | Sinorhizobium_meliloti_AK83_uid52607 | 0.606 |
| *Acidithiobacillus* | Acidithiobacillus_ferrooxidans_ATCC_23270_uid57649 | 0.606 |
| *Rubrobacter* | Rubrobacter_xylanophilus_DSM_9941_uid58057 | 0.605 |
| *Weeksella* | Weeksella_virosa_DSM_16922_uid63627 | 0.605 |
| *Sebaldella* | Sebaldella_termitidis_ATCC_33386_uid41865 | 0.605 |
| *Francisella* | Francisella_noatunensis_orientalis_Toba_04_uid164779 | 0.605 |
| *Methylovorus* | Methylovorus_glucosetrophus_SIP3_4_uid59367 | 0.602 |
| *Kytococcus* | Kytococcus_sedentarius_DSM_20547_uid59071 | 0.601 |
| *Streptomyces* | Streptomyces_venezuelae_ATCC_10712_uid177080 | 0.601 |
| *Brevundimonas* | Brevundimonas_subvibrioides_ATCC_15264_uid42117 | 0.6 |
| *Vibrio* | Vibrio_vulnificus_CMCP6_uid62909 | 0.599 |
| *Streptomyces* | Streptomyces_PAMC26508_uid197217 | 0.597 |
| *Ketogulonicigenium* | Ketogulonicigenium_vulgare_Y25_uid59581 | 0.596 |
| *Streptomyces* | Streptomyces_griseus_NBRC_13350_uid58983 | 0.596 |
| *Methylobacterium* | Methylobacterium_extorquens_PA1_uid58821 | 0.595 |
| *Flavobacterium* | Flavobacterium_columnare_ATCC_49512_uid80731 | 0.595 |
| *Polaromonas* | Polaromonas_JS666_uid58207 | 0.594 |
| *Clavibacter* | Clavibacter_michiganensis_nebraskensis_NCPPB_2581_uid195908 | 0.594 |
| *Acidiphilium* | Acidiphilium_cryptum_JF_5_uid58447 | 0.594 |
| *Candidatus_Blochmannia* | Candidatus_Blochmannia_chromaiodes_640_uid185308 | 0.594 |
| *Desulfurobacterium* | Desulfurobacterium_thermolithotrophum_DSM_11699_uid63405 | 0.593 |
| *Sinorhizobium* | Sinorhizobium_meliloti_SM11_uid159685 | 0.593 |
| *Clavibacter* | Clavibacter_michiganensis_NCPPB_382_uid61625 | 0.593 |
| *Vibrio* | Vibrio_EJY3_uid83161 | 0.592 |
| *Treponema* | Treponema_paraluiscuniculi_Cuniculi_A_uid68447 | 0.592 |
| *Achromobacter* | Achromobacter_xylosoxidans_NBRC_15126_uid232243 | 0.592 |
| *Dechloromonas* | Dechloromonas_aromatica_RCB_uid58025 | 0.591 |
| *Sphingobium* | Sphingobium_chlorophenolicum_L_1_uid52597 | 0.591 |
| *Methylococcus* | Methylococcus_capsulatus_Bath_uid57607 | 0.591 |
| *Achromobacter* | Achromobacter_xylosoxidans_A8_uid59899 | 0.59 |
| *Ketogulonicigenium* | Ketogulonicigenium_vulgare_WSH_001_uid161161 | 0.59 |
| *Syntrophobacter* | Syntrophobacter_fumaroxidans_MPOB_uid58177 | 0.59 |
| *Desulfosporosinus* | Desulfosporosinus_acidiphilus_SJ4_uid156759 | 0.589 |
| *Sphingobium* | Sphingobium_japonicum_UT26S_uid47077 | 0.589 |
| *Candidatus_Liberibacter* | Candidatus_Liberibacter_americanus_Sao_Paulo_uid227424 | 0.588 |
| *Helicobacter* | Helicobacter_pylori_Aklavik86_uid182202 | 0.587 |
| *Methylobacterium* | Methylobacterium_radiotolerans_JCM_2831_uid58845 | 0.586 |
| *Clavibacter* | Clavibacter_michiganensis_sepedonicus_uid61577 | 0.586 |
| *Mycobacterium* | Mycobacterium_avium_paratuberculosis_MAP4_uid202426 | 0.585 |
| *Mycobacterium* | Mycobacterium_avium_paratuberculosis_K_10_uid57699 | 0.585 |
| *Sulfuricella* | Sulfuricella_denitrificans_skB26_uid170240 | 0.584 |
| *Streptomyces* | Streptomyces_hygroscopicus_jinggangensis_TL01_uid189753 | 0.584 |
| *Ramlibacter* | Ramlibacter_tataouinensis_TTB310_uid68279 | 0.582 |
| *Mycoplasma* | Mycoplasma_pneumoniae_FH_uid162027 | 0.582 |
| *Cellulomonas* | Cellulomonas_flavigena_DSM_20109_uid48821 | 0.582 |
| *Mycoplasma* | Mycoplasma_pneumoniae_309_uid85495 | 0.581 |
| *Sinorhizobium* | Sinorhizobium_fredii_HH103_uid86865 | 0.58 |
| *Syntrophothermus* | Syntrophothermus_lipocalidus_DSM_12680_uid49527 | 0.579 |
| *Mycoplasma* | Mycoplasma_pneumoniae_M129_B7_uid185759 | 0.578 |
| *Fibrella* | Fibrella_aestuarina_uid178352 | 0.578 |
| *Caulobacter* | Caulobacter_crescentus_CB15_uid57891 | 0.577 |
| *Pseudogulbenkiania* | Pseudogulbenkiania_NH8B_uid73423 | 0.577 |
| *Alicycliphilus* | Alicycliphilus_denitrificans_BC_uid49953 | 0.576 |
| *Arthrobacter* | Arthrobacter_FB24_uid58141 | 0.576 |
| *Caulobacter* | Caulobacter_crescentus_NA1000_uid59307 | 0.576 |
| *Dictyoglomus* | Dictyoglomus_turgidum_DSM_6724_uid59177 | 0.575 |
| *Collimonas* | Collimonas_fungivorans_Ter331_uid70793 | 0.575 |
| *Sinorhizobium* | Sinorhizobium_medicae_WSM419_uid58549 | 0.574 |
| *Mycobacterium* | Mycobacterium_JDM601_uid67369 | 0.573 |
| *Methylobacterium* | Methylobacterium_chloromethanicum_CM4_uid58933 | 0.573 |
| *Vibrio* | Vibrio_harveyi_ATCC_BAA_1116_uid58957 | 0.572 |
| *Mycoplasma* | Mycoplasma_pneumoniae_M129_uid57709 | 0.572 |
| *Methylobacterium* | Methylobacterium_extorquens_DM4_uid61617 | 0.571 |
| *Leptothrix* | Leptothrix_cholodnii_SP_6_uid58971 | 0.569 |
| *Thioflavicoccus* | Thioflavicoccus_mobilis_8321_uid184343 | 0.568 |
| *Solitalea* | Solitalea_canadensis_DSM_3403_uid81783 | 0.567 |
| *Gallionella* | Gallionella_capsiferriformans_ES_2_uid51505 | 0.566 |
| *Streptomyces* | Streptomyces_hygroscopicus_jinggangensis_5008_uid89409 | 0.566 |
| *Dictyoglomus* | Dictyoglomus_thermophilum_H_6_12_uid59439 | 0.564 |
| *Pseudoxanthomonas* | Pseudoxanthomonas_spadix_BD_a59_uid75113 | 0.564 |
| *Vibrio* | Vibrio_vulnificus_YJ016_uid58007 | 0.563 |
| *Chlorobium* | Chlorobium_limicola_DSM_245_uid58127 | 0.563 |
| *Vibrio* | Vibrio_harveyi_ATCC_BAA_1116_uid218471 | 0.561 |
| *Achromobacter* | Achromobacter_xylosoxidans_uid205255 | 0.561 |
| *Marinithermus* | Marinithermus_hydrothermalis_DSM_14884_uid65783 | 0.56 |
| *Helicobacter* | Helicobacter_pylori_HPAG1_uid58517 | 0.56 |
| *Helicobacter* | Helicobacter_pylori_uid159983 | 0.559 |
| *Hyphomicrobium* | Hyphomicrobium_nitrativorans_NL23_uid230615 | 0.558 |
| *Thioalkalivibrio* | Thioalkalivibrio_nitratireducens_DSM_14787_uid184011 | 0.558 |
| *Pandoraea* | Pandoraea_RB_44_uid231151 | 0.558 |
| *Vibrio* | Vibrio_anguillarum_775_uid68057 | 0.558 |
| *Dehalococcoides* | Dehalococcoides_VS_uid42393 | 0.557 |
| *Helicobacter* | Helicobacter_pylori_OK310_uid193716 | 0.557 |
| *Rhodopseudomonas* | Rhodopseudomonas_palustris_BisB5_uid58441 | 0.555 |
| *Helicobacter* | Helicobacter_pylori_BM012A_uid229744 | 0.555 |
| *Exiguobacterium* | Exiguobacterium_MH3_uid227425 | 0.555 |
| *Helicobacter* | Helicobacter_pylori_Aklavik117_uid182201 | 0.554 |
| *Helicobacter* | Helicobacter_pylori_BM012S_uid229881 | 0.553 |
| *Helicobacter* | Helicobacter_pylori_Sat464_uid159467 | 0.553 |
| *Thermodesulfobacterium* | Thermodesulfobacterium_OPB45_uid68283 | 0.552 |
| *Tolumonas* | Tolumonas_auensis_DSM_9187_uid59395 | 0.55 |
| *Rhodopseudomonas* | Rhodopseudomonas_palustris_DX_1_uid43327 | 0.55 |
| *Helicobacter* | Helicobacter_pylori_v225d_uid159639 | 0.55 |
| *Helicobacter* | Helicobacter_pylori_Shi169_uid162209 | 0.55 |
| *Helicobacter* | Helicobacter_pylori_F30_uid159991 | 0.549 |
| *Prochlorococcus* | Prochlorococcus_marinus_MIT_9301_uid58437 | 0.549 |
| *Rhodopseudomonas* | Rhodopseudomonas_palustris_BisB18_uid58443 | 0.548 |
| *Pandoraea* | Pandoraea_pnomenusa_3kgm_uid229878 | 0.548 |
| *Variovorax* | Variovorax_paradoxus_EPS_uid62107 | 0.547 |
| *Methylibium* | Methylibium_petroleiphilum_PM1_uid58085 | 0.546 |
| *Flavobacterium* | Flavobacterium_branchiophilum_FL_15_uid73421 | 0.546 |
| *Lysinibacillus* | Lysinibacillus_sphaericus_C3_41_uid58945 | 0.546 |
| *Helicobacter* | Helicobacter_pylori_SNT49_uid159615 | 0.545 |
| *Streptomyces* | Streptomyces_avermitilis_MA_4680_uid57739 | 0.545 |
| *Helicobacter* | Helicobacter_pylori_51_uid161925 | 0.544 |
| *Helicobacter* | Helicobacter_pylori_Puno135_uid161157 | 0.544 |
| *Natranaerobius* | Natranaerobius_thermophilus_JW_NM_WN_LF_uid59001 | 0.543 |
| *Taylorella* | Taylorella_equigenitalis_14_56_uid197193 | 0.543 |
| *Prochlorococcus* | Prochlorococcus_marinus_MIT_9215_uid58819 | 0.542 |
| *Acidiphilium* | Acidiphilium_multivorum_AIU301_uid63345 | 0.542 |
| *Helicobacter* | Helicobacter_pylori_F16_uid161145 | 0.541 |
| *Delftia* | Delftia_Cs1_4_uid67319 | 0.541 |
| *Sulfurihydrogenibium* | Sulfurihydrogenibium_azorense_Az_Fu1_uid58121 | 0.541 |
| *Alicycliphilus* | Alicycliphilus_denitrificans_K601_uid66307 | 0.54 |
| *Helicobacter* | Helicobacter_pylori_Puno120_uid159611 | 0.54 |
| *Anoxybacillus* | Anoxybacillus_flavithermus_WK1_uid59135 | 0.539 |
| *Cellulomonas* | Cellulomonas_fimi_ATCC_484_uid66779 | 0.539 |
| *Rhodopseudomonas* | Rhodopseudomonas_palustris_CGA009_uid62901 | 0.538 |
| *Helicobacter* | Helicobacter_pylori_UM032_uid203025 | 0.537 |
| *Streptomyces* | Streptomyces_davawensis_JCM_4913_uid193657 | 0.537 |
| *Helicobacter* | Helicobacter_pylori_26695_uid178201 | 0.536 |
| *Mycobacterium* | Mycobacterium_chubuense_NBB4_uid168322 | 0.535 |
| *Helicobacter* | Helicobacter_pylori_Rif2_uid178203 | 0.535 |
| *Helicobacter* | Helicobacter_pylori_26695_uid57787 | 0.535 |
| *Helicobacter* | Helicobacter_pylori_Rif1_uid178202 | 0.535 |
| *Helicobacter* | Helicobacter_pylori_OK113_uid193715 | 0.535 |
| *Helicobacter* | Helicobacter_pylori_UM299_uid203026 | 0.534 |
| *Dyadobacter* | Dyadobacter_fermentans_DSM_18053_uid59049 | 0.533 |
| *Helicobacter* | Helicobacter_pylori_UM298_uid213226 | 0.533 |
| *Xanthomonas* | Xanthomonas_campestris_raphani_756C_uid159539 | 0.532 |
| *Delftia* | Delftia_acidovorans_SPH_1_uid58703 | 0.532 |
| *Caulobacter* | Caulobacter_segnis_ATCC_21756_uid41709 | 0.53 |
| *Pedobacter* | Pedobacter_heparinus_DSM_2366_uid59111 | 0.53 |
| *Syntrophus* | Syntrophus_aciditrophicus_SB_uid58539 | 0.529 |
| *Rhodopseudomonas* | Rhodopseudomonas_palustris_HaA2_uid58439 | 0.529 |
| *Xanthomonas* | Xanthomonas_campestris_ATCC_33913_uid57887 | 0.528 |
| *Salinibacter* | Salinibacter_ruber_DSM_13855_uid58513 | 0.528 |
| *Anaerolinea* | Anaerolinea_thermophila_UNI_1_uid62245 | 0.528 |
| *Helicobacter* | Helicobacter_pylori_F57_uid161143 | 0.527 |
| *Pusillimonas* | Pusillimonas_T7_7_uid66391 | 0.527 |
| *Ferrimonas* | Ferrimonas_balearica_DSM_9799_uid53371 | 0.526 |
| *Candidatus_Liberibacter* | Candidatus_Liberibacter_solanacearum_CLso_ZC1_uid61245 | 0.526 |
| *Helicobacter* | Helicobacter_pylori_F32_uid161139 | 0.526 |
| *Helicobacter* | Helicobacter_pylori_Shi470_uid59165 | 0.525 |
| *Rickettsia* | Rickettsia_bellii_OSU_85_389_uid58681 | 0.525 |
| *Mycobacterium* | Mycobacterium_MCS_uid58465 | 0.524 |
| *Mycobacterium* | Mycobacterium_avium_104_uid57693 | 0.524 |
| *Ralstonia* | Ralstonia_eutropha_H16_uid62925 | 0.524 |
| *Paracoccus* | Paracoccus_aminophilus_JCM_7686_uid214795 | 0.524 |
| *Dehalococcoides* | Dehalococcoides_GT_uid42115 | 0.523 |
| *Rickettsia* | Rickettsia_bellii_RML369_C_uid58405 | 0.523 |
| *Zymomonas* | Zymomonas_mobilis_CP4___NRRL_B_14023_uid229874 | 0.523 |
| *Aromatoleum* | Aromatoleum_aromaticum_EbN1_uid58231 | 0.523 |
| *Rhodopseudomonas* | Rhodopseudomonas_palustris_TIE_1_uid58995 | 0.523 |
| *Helicobacter* | Helicobacter_pylori_G27_uid59305 | 0.522 |
| *Helicobacter* | Helicobacter_pylori_83_uid161153 | 0.522 |
| *Phycisphaera* | Phycisphaera_mikurensis_NBRC_102666_uid157331 | 0.522 |
| *Zymomonas* | Zymomonas_mobilis_ATCC_29191_uid170612 | 0.522 |
| *Xanthomonas* | Xanthomonas_campestris_uid61643 | 0.521 |
| *Polaribacter* | Polaribacter_MED152_uid54207 | 0.521 |
| *Mycobacterium* | Mycobacterium_KMS_uid58491 | 0.521 |
| *Helicobacter* | Helicobacter_pylori_Cuz20_uid159987 | 0.521 |
| *Vibrio* | Vibrio_splendidus_LGP32_uid59353 | 0.52 |
| *Xanthomonas* | Xanthomonas_campestris_8004_uid57595 | 0.52 |
| *Rhodopseudomonas* | Rhodopseudomonas_palustris_BisA53_uid58445 | 0.519 |
| *Zymomonas* | Zymomonas_mobilis_NCIMB_11163_uid41019 | 0.519 |
| *Salinibacter* | Salinibacter_ruber_M8_uid47323 | 0.519 |
| *Helicobacter* | Helicobacter_pylori_XZ274_uid165869 | 0.518 |
| *Helicobacter* | Helicobacter_pylori_B38_uid59415 | 0.518 |
| *Shewanella* | Shewanella_amazonensis_SB2B_uid58257 | 0.518 |
| *Desulfatibacillum* | Desulfatibacillum_alkenivorans_AK_01_uid58913 | 0.518 |
| *Helicobacter* | Helicobacter_pylori_SJM180_uid53541 | 0.517 |
| *Candidatus_Pelagibacter* | Candidatus_Pelagibacter_IMCC9063_uid66305 | 0.517 |
| *Zymomonas* | Zymomonas_mobilis_ZM4_uid58095 | 0.516 |
| *Helicobacter* | Helicobacter_pylori_PeCan4_uid53539 | 0.516 |
| *Mycobacterium* | Mycobacterium_gilvum_PYR_GCK_uid59421 | 0.515 |
| *Leadbetterella* | Leadbetterella_byssophila_DSM_17132_uid60161 | 0.515 |
| *Helicobacter* | Helicobacter_pylori_35A_uid49903 | 0.515 |
| *Agrobacterium* | Agrobacterium_radiobacter_K84_uid58269 | 0.514 |
| *Dehalococcoides* | Dehalococcoides_BAV1_uid58477 | 0.514 |
| *Mycoplasma* | Mycoplasma_ovis_Michigan_uid232247 | 0.514 |
| *Zymomonas* | Zymomonas_mobilis_ATCC_10988_uid55403 | 0.512 |
| *Ehrlichia* | Ehrlichia_canis_Jake_uid58071 | 0.511 |
| *Dehalococcoides* | Dehalococcoides_CBDB1_uid58413 | 0.511 |
| *Novosphingobium* | Novosphingobium_PP1Y_uid67383 | 0.511 |
| *Mycobacterium* | Mycobacterium_intracellulare_ATCC_13950_uid167994 | 0.511 |
| *Helicobacter* | Helicobacter_acinonychis_Sheeba_uid58685 | 0.511 |
| *Mycobacterium* | Mycobacterium_JLS_uid58489 | 0.51 |
| *Acidobacterium* | Acidobacterium_capsulatum_ATCC_51196_uid59127 | 0.509 |
| *Tistrella* | Tistrella_mobilis_KA081020_065_uid167486 | 0.508 |
| *Mycobacterium* | Mycobacterium_gilvum_Spyr1_uid61403 | 0.507 |
| *Helicobacter* | Helicobacter_pylori_PeCan18_uid162211 | 0.507 |
| *Helicobacter* | Helicobacter_pylori_UM066_uid203028 | 0.506 |
| *Stenotrophomonas* | Stenotrophomonas_maltophilia_JV3_uid72473 | 0.506 |
| *Xanthomonas* | Xanthomonas_axonopodis_citrumelo_F1_uid73179 | 0.505 |
| *Helicobacter* | Helicobacter_pylori_Shi112_uid162207 | 0.505 |
| *Leifsonia* | Leifsonia_xyli_cynodontis_DSM_46306_uid221294 | 0.505 |
| *Cytophaga* | Cytophaga_hutchinsonii_ATCC_33406_uid57651 | 0.504 |
| *Novosphingobium* | Novosphingobium_aromaticivorans_DSM_12444_uid57747 | 0.504 |
| *Psychrobacter* | Psychrobacter_G_uid210641 | 0.503 |
| *Helicobacter* | Helicobacter_pylori_Shi417_uid162205 | 0.503 |
| *Helicobacter* | Helicobacter_pylori_HUP_B14_uid162213 | 0.503 |
| *Helicobacter* | Helicobacter_pylori_India7_uid161149 | 0.503 |
| *Vibrio* | Vibrio_nigripulchritudo_SnF1_uid222819 | 0.503 |
| *Taylorella* | Taylorella_asinigenitalis_14_45_uid197194 | 0.502 |
| *Vibrio* | Vibrio_fischeri_ES114_uid58163 | 0.502 |
| *Psychrobacter* | Psychrobacter_arcticus_273_4_uid58021 | 0.501 |
| *Arthrobacter* | Arthrobacter_arilaitensis_Re117_uid53509 | 0.501 |
| *Mycobacterium* | Mycobacterium_intracellulare_MOTT_02_uid89387 | 0.501 |
| *Deferribacter* | Deferribacter_desulfuricans_SSM1_uid46653 | 0.5 |
| *Dehalococcoides* | Dehalococcoides_mccartyi_DCMB5_uid190184 | 0.499 |
| *Helicobacter* | Helicobacter_pylori_UM037_uid203027 | 0.499 |
| *Helicobacter* | Helicobacter_pylori_Lithuania75_uid159491 | 0.498 |
| *Leifsonia* | Leifsonia_xyli_CTCB07_uid57759 | 0.498 |
| *Sphingomonas* | Sphingomonas_MM_1_uid193771 | 0.498 |
| *Ralstonia* | Ralstonia_eutropha_JMP134_uid58047 | 0.498 |
| *Streptomyces* | Streptomyces_scabiei_87_22_uid46531 | 0.498 |
| *Frateuria* | Frateuria_aurantia_DSM_6220_uid81775 | 0.497 |
| *Ehrlichia* | Ehrlichia_chaffeensis_Arkansas_uid57933 | 0.496 |
| *Xanthomonas* | Xanthomonas_oryzae_oryzicola_BLS256_uid54411 | 0.496 |
| *Acetobacter* | Acetobacter_pasteurianus_IFO_3283_01_42C_uid158377 | 0.496 |
| *Helicobacter* | Helicobacter_pylori_B8_uid49873 | 0.495 |
| *Xanthomonas* | Xanthomonas_albilineans_GPE_PC73_uid43163 | 0.495 |
| *Dehalococcoides* | Dehalococcoides_mccartyi_GY50_uid230266 | 0.494 |
| *Thermodesulfovibrio* | Thermodesulfovibrio_yellowstonii_DSM_11347_uid59257 | 0.492 |
| *Thermodesulfobium* | Thermodesulfobium_narugense_DSM_14796_uid66601 | 0.491 |
| *Helicobacter* | Helicobacter_pylori_P12_uid59327 | 0.49 |
| *Hyphomicrobium* | Hyphomicrobium_denitrificans_ATCC_51888_uid50325 | 0.49 |
| *Mycobacterium* | Mycobacterium_VKM_Ac_1815D_uid199859 | 0.489 |
| *Acidithiobacillus* | Acidithiobacillus_ferrivorans_SS3_uid67387 | 0.489 |
| *Mycobacterium* | Mycobacterium_vanbaalenii_PYR_1_uid58463 | 0.489 |
| *Truepera* | Truepera_radiovictrix_DSM_17093_uid49533 | 0.487 |
| *Mycobacterium* | Mycobacterium_intracellulare_MOTT_64_uid89385 | 0.487 |
| *Shewanella* | Shewanella_loihica_PV_4_uid58349 | 0.486 |
| *Bradyrhizobium* | Bradyrhizobium_S23321_uid158167 | 0.486 |
| *Flavobacterium* | Flavobacterium_johnsoniae_UW101_uid58493 | 0.486 |
| *Prochlorococcus* | Prochlorococcus_marinus_MIT_9312_uid58357 | 0.485 |
| *Mycobacterium* | Mycobacterium_indicus_pranii_MTCC_9506_uid175523 | 0.485 |
| *Acetobacter* | Acetobacter_pasteurianus_IFO_3283_12_uid158379 | 0.484 |
| *Acetobacter* | Acetobacter_pasteurianus_IFO_3283_32_uid158375 | 0.484 |
| *Acetobacter* | Acetobacter_pasteurianus_IFO_3283_07_uid158381 | 0.484 |
| *Verminephrobacter* | Verminephrobacter_eiseniae_EF01_2_uid58675 | 0.484 |
| *Acetobacter* | Acetobacter_pasteurianus_IFO_3283_22_uid158383 | 0.484 |
| *Acetobacter* | Acetobacter_pasteurianus_IFO_3283_03_uid158373 | 0.484 |
| *Acetobacter* | Acetobacter_pasteurianus_IFO_3283_26_uid158531 | 0.484 |
| *Acetobacter* | Acetobacter_pasteurianus_IFO_3283_01_uid59279 | 0.484 |
| *Mycobacterium* | Mycobacterium_yongonense_05_1390_uid189649 | 0.483 |
| *Methylobacterium* | Methylobacterium_4_46_uid58843 | 0.483 |
| *Candidatus_Liberibacter* | Candidatus_Liberibacter_asiaticus_gxpsy_uid193764 | 0.483 |
| *Acetobacter* | Acetobacter_pasteurianus_386B_uid214433 | 0.482 |
| *Xanthomonas* | Xanthomonas_axonopodis_Xac29_1_uid193774 | 0.481 |
| *Helicobacter* | Helicobacter_pylori_Gambia94_24_uid159493 | 0.481 |
| *Bartonella* | Bartonella_bacilliformis_KC583_uid58533 | 0.48 |
| *Spirochaeta* | Spirochaeta_smaragdinae_DSM_11293_uid51369 | 0.48 |
| *Xanthomonas* | Xanthomonas_axonopodis_citri_306_uid57889 | 0.48 |
| *Streptomyces* | Streptomyces_violaceusniger_Tu_4113_uid52609 | 0.479 |
| *Mycobacterium* | Mycobacterium_MOTT36Y_uid164001 | 0.479 |
| *Xanthomonas* | Xanthomonas_campestris_vesicatoria_85_10_uid58321 | 0.478 |
| *Arthrobacter* | Arthrobacter_aurescens_TC1_uid58109 | 0.478 |
| *Prochlorococcus* | Prochlorococcus_marinus_AS9601_uid58307 | 0.478 |
| *Rhodomicrobium* | Rhodomicrobium_vannielii_ATCC_17100_uid43247 | 0.478 |
| *Ralstonia* | Ralstonia_pickettii_DTP0602_uid222229 | 0.478 |
| *Helicobacter* | Helicobacter_pylori_SouthAfrica7_uid159989 | 0.478 |
| *Stenotrophomonas* | Stenotrophomonas_maltophilia_R551_3_uid58657 | 0.477 |
| *Xylanimonas* | Xylanimonas_cellulosilytica_DSM_15894_uid41935 | 0.477 |
| *Arcobacter* | Arcobacter_nitrofigilis_DSM_7299_uid49001 | 0.477 |
| *Sulfurimonas* | Sulfurimonas_autotrophica_DSM_16294_uid53043 | 0.476 |
| *Vibrio* | Vibrio_fischeri_MJ11_uid58907 | 0.476 |
| *Sphingobium* | Sphingobium_SYK_6_uid73353 | 0.476 |
| *Helicobacter* | Helicobacter_pylori_SouthAfrica20_uid216150 | 0.476 |
| *Synechococcus* | Synechococcus_elongatus_PCC_7942_uid58045 | 0.475 |
| *Opitutus* | Opitutus_terrae_PB90_1_uid58965 | 0.474 |
| *Stenotrophomonas* | Stenotrophomonas_maltophilia_D457_uid162199 | 0.474 |
| *Helicobacter* | Helicobacter_pylori_J99_uid57789 | 0.474 |
| *Marinobacter* | Marinobacter_hydrocarbonoclasticus_ATCC_49840_uid162203 | 0.474 |
| *Sphingobacterium* | Sphingobacterium_21_uid64755 | 0.473 |
| *Myxococcus* | Myxococcus_fulvus_HW_1_uid68443 | 0.473 |
| *Mycoplasma* | Mycoplasma_wenyonii_Massachusetts_uid170731 | 0.473 |
| *Halobacillus* | Halobacillus_halophilus_DSM_2266_uid162033 | 0.472 |
| *Spirochaeta* | Spirochaeta_L21_RPul_D2_uid231658 | 0.472 |
| *Xanthomonas* | Xanthomonas_oryzae_KACC_10331_uid58155 | 0.472 |
| *Synechococcus* | Synechococcus_elongatus_PCC_6301_uid58235 | 0.472 |
| *Xanthomonas* | Xanthomonas_fuscans_4834_R_uid222814 | 0.472 |
| *Xanthomonas* | Xanthomonas_citri_Aw12879_uid194444 | 0.472 |
| *Streptomyces* | Streptomyces_bingchenggensis_BCW_1_uid82931 | 0.471 |
| *Xanthomonas* | Xanthomonas_oryzae_MAFF_311018_uid58547 | 0.47 |
| *Mycoplasma* | Mycoplasma_suis_Illinois_uid61897 | 0.469 |
| *Sinorhizobium* | Sinorhizobium_fredii_USDA_257_uid168059 | 0.468 |
| *Candidatus_Liberibacter* | Candidatus_Liberibacter_asiaticus_psy62_uid59227 | 0.468 |
| *Mycoplasma* | Mycoplasma_suis_KI3806_uid63665 | 0.468 |
| *Azorhizobium* | Azorhizobium_caulinodans_ORS_571_uid58905 | 0.467 |
| *Rhodoferax* | Rhodoferax_ferrireducens_T118_uid58353 | 0.466 |
| *Isoptericola* | Isoptericola_variabilis_225_uid67501 | 0.466 |
| *Helicobacter* | Helicobacter_pylori_ELS37_uid158157 | 0.465 |
| *Granulibacter* | Granulibacter_bethesdensis_CGDNIH1_uid58661 | 0.464 |
| *Arthrobacter* | Arthrobacter_Rue61a_uid174511 | 0.464 |
| *Stenotrophomonas* | Stenotrophomonas_maltophilia_K279a_uid61647 | 0.464 |
| *Shewanella* | Shewanella_MR_4_uid58345 | 0.462 |
| *Psychrobacter* | Psychrobacter_cryohalolentis_K5_uid58373 | 0.462 |
| *Mycobacterium* | Mycobacterium_bovis_BCG_Pasteur_1173P2_uid58781 | 0.461 |
| *Hyphomicrobium* | Hyphomicrobium_denitrificans_1NES1_uid179904 | 0.46 |
| *Halothiobacillus* | Halothiobacillus_neapolitanus_c2_uid41317 | 0.459 |
| *Halomonas* | Halomonas_elongata_DSM_2581_uid52781 | 0.459 |
| *Mycobacterium* | Mycobacterium_smegmatis_MC2_155_uid171958 | 0.459 |
| *Mycobacterium* | Mycobacterium_smegmatis_MC2_155_uid57701 | 0.459 |
| *Mycoplasma* | Candidatus_Mycoplasma_haemominutum__Birmingham_1__uid197195 | 0.458 |
| *Agrobacterium* | Agrobacterium_vitis_S4_uid58249 | 0.456 |
| *Methylobacillus* | Methylobacillus_flagellatus_KT_uid58049 | 0.456 |
| *Prochlorococcus* | Prochlorococcus_marinus_pastoris_CCMP1986_uid57761 | 0.456 |
| *Bradyrhizobium* | Bradyrhizobium_japonicum_USDA_6_uid158851 | 0.455 |
| *Bartonella* | Bartonella_clarridgeiae_73_uid62131 | 0.454 |
| *Mycobacterium* | Mycobacterium_bovis_AF2122_97_uid57695 | 0.454 |
| *Mycobacterium* | Mycobacterium_bovis_BCG_Mexico_uid86889 | 0.453 |
| *Owenweeksia* | Owenweeksia_hongkongensis_DSM_17368_uid82951 | 0.453 |
| *Gloeobacter* | Gloeobacter_violaceus_PCC_7421_uid58011 | 0.453 |
| *Mycobacterium* | Mycobacterium_canettii_CIPT_140070008_uid184832 | 0.452 |
| *Prochlorococcus* | Prochlorococcus_marinus_MIT_9515_uid58313 | 0.452 |
| *Mycobacterium* | Mycobacterium_bovis_BCG_Tokyo_172_uid59281 | 0.452 |
| *Methylobacterium* | Methylobacterium_nodulans_ORS_2060_uid59023 | 0.452 |
| *Mycobacterium* | Mycobacterium_bovis_BCG_Korea_1168P_uid189029 | 0.451 |
| *Mycobacterium* | Mycobacterium_africanum_GM041182_uid68839 | 0.45 |
| *Marinobacter* | Marinobacter_aquaeolei_VT8_uid59419 | 0.45 |
| *Prosthecochloris* | Prosthecochloris_aestuarii_DSM_271_uid58151 | 0.45 |
| *Mycobacterium* | Mycobacterium_tuberculosis_KZN_4207_uid83619 | 0.45 |
| *Candidatus_Accumulibacter* | Candidatus_Accumulibacter_phosphatis_clade_IIA_UW_1_uid59207 | 0.45 |
| *Mycobacterium* | Mycobacterium_tuberculosis_H37Rv_uid170532 | 0.449 |
| *Ehrlichia* | Ehrlichia_muris_AS145_uid232250 | 0.449 |
| *Mycobacterium* | Mycobacterium_tuberculosis_CCDC5079_uid161943 | 0.448 |
| *Mycobacterium* | Mycobacterium_tuberculosis_CTRI_2_uid161997 | 0.448 |
| *Mycobacterium* | Mycobacterium_tuberculosis_H37Ra_uid58853 | 0.448 |
| *Mycobacterium* | Mycobacterium_tuberculosis_H37Rv_uid57777 | 0.448 |
| *Janthinobacterium* | Janthinobacterium_Marseille_uid58603 | 0.448 |
| *Phenylobacterium* | Phenylobacterium_zucineum_HLK1_uid58959 | 0.448 |
| *Mycobacterium* | Mycobacterium_tuberculosis_Haarlem_uid54453 | 0.448 |
| *Mycobacterium* | Mycobacterium_tuberculosis_KZN_1435_uid59069 | 0.447 |
| *Mycobacterium* | Mycobacterium_tuberculosis_CCDC5180_uid161941 | 0.447 |
| *Mycobacterium* | Mycobacterium_tuberculosis_RGTB327_uid157907 | 0.447 |
| *Mycobacterium* | Mycobacterium_tuberculosis_Erdman___ATCC_35801_uid193763 | 0.447 |
| *Mycobacterium* | Mycobacterium_tuberculosis_EAI5_uid212307 | 0.447 |
| *Mycobacterium* | Mycobacterium_tuberculosis_CDC1551_uid57775 | 0.447 |
| *Caulobacter* | Caulobacter_K31_uid58551 | 0.447 |
| *Shewanella* | Shewanella_MR_7_uid58343 | 0.447 |
| *Mycobacterium* | Mycobacterium_canettii_CIPT_140070010_uid184828 | 0.446 |
| *Mycobacterium* | Mycobacterium_tuberculosis_uid185758 | 0.446 |
| *Mycobacterium* | Mycobacterium_tuberculosis_EAI5_NITR206_uid202218 | 0.446 |
| *Mycobacterium* | Mycobacterium_tuberculosis_KZN_605_uid54947 | 0.446 |
| *Mycobacterium* | Mycobacterium_tuberculosis_F11_uid58417 | 0.446 |
| *Mycobacterium* | Mycobacterium_tuberculosis_CCDC5079_uid203790 | 0.446 |
| *Mycobacterium* | Mycobacterium_tuberculosis_Beijing_NITR203_uid197218 | 0.445 |
| *Xanthomonas* | Xanthomonas_oryzae_PXO99A_uid59131 | 0.445 |
| *Gluconacetobacter* | Gluconacetobacter_diazotrophicus_PAl_5_uid59075 | 0.445 |
| *Gluconacetobacter* | Gluconacetobacter_diazotrophicus_PAl_5_uid61587 | 0.443 |
| *Mycobacterium* | Mycobacterium_tuberculosis_UT205_uid162183 | 0.443 |
| *Nitrosomonas* | Nitrosomonas_europaea_ATCC_19718_uid57647 | 0.442 |
| *Bradyrhizobium* | Bradyrhizobium_ORS_278_uid58941 | 0.442 |
| *Rhodanobacter* | Rhodanobacter_2APBS1_uid74431 | 0.442 |
| *Myxococcus* | Myxococcus_xanthus_DK_1622_uid58003 | 0.442 |
| *Mycobacterium* | Mycobacterium_canettii_CIPT_140070017_uid184830 | 0.441 |
| *Erythrobacter* | Erythrobacter_litoralis_HTCC2594_uid58299 | 0.441 |
| *Mycobacterium* | Mycobacterium_canettii_CIPT_140060008_uid184829 | 0.441 |
| *Xanthobacter* | Xanthobacter_autotrophicus_Py2_uid58453 | 0.44 |
| *Candidatus_Chloracidobacterium* | Candidatus_Chloracidobacterium_thermophilum_B_uid73587 | 0.44 |
| *Ruegeria* | Ruegeria_pomeroyi_DSS_3_uid57863 | 0.44 |
| *Serratia* | Serratia_symbiotica__Cinara_cedri__uid82363 | 0.439 |
| *Mycobacterium* | Mycobacterium_tuberculosis_RGTB423_uid162179 | 0.438 |
| *Thiocystis* | Thiocystis_violascens_DSM_198_uid74025 | 0.438 |
| *Helicobacter* | Helicobacter_mustelae_12198_uid46647 | 0.438 |
| *Mycobacterium* | Mycobacterium_tuberculosis_CAS_NITR204_uid202217 | 0.437 |
| *Mycobacterium* | Mycobacterium_canettii_CIPT_140010059_uid70731 | 0.437 |
| *Bradyrhizobium* | Bradyrhizobium_japonicum_USDA_110_uid57599 | 0.437 |
| *Shewanella* | Shewanella_ANA_3_uid58347 | 0.435 |
| *Chromohalobacter* | Chromohalobacter_salexigens_DSM_3043_uid62921 | 0.433 |
| *Mesorhizobium* | Mesorhizobium_ciceri_biovar_biserrulae_WSM1271_uid62101 | 0.433 |
| *Starkeya* | Starkeya_novella_DSM_506_uid48815 | 0.432 |
| *Serratia* | Serratia_ATCC_39006_uid218470 | 0.431 |
| *Chlamydia* | Chlamydia_trachomatis_RC_J_s_122_uid213392 | 0.43 |
| *Mycobacterium* | Mycobacterium_massiliense_GO_06_uid170732 | 0.43 |
| *Alcanivorax* | Alcanivorax_dieselolei_B5_uid176364 | 0.429 |
| *Ochrobactrum* | Ochrobactrum_anthropi_ATCC_49188_uid58921 | 0.427 |
| *Mesorhizobium* | Mesorhizobium_australicum_WSM2073_uid75101 | 0.425 |
| *Caldilinea* | Caldilinea_aerophila_DSM_14535___NBRC_104270_uid158165 | 0.424 |
| *Caldisericum* | Caldisericum_exile_AZM16c01_uid158173 | 0.423 |
| *Bartonella* | Bartonella_vinsonii_berkhoffii_Winnie_uid189951 | 0.422 |
| *Chlamydia* | Chlamydia_trachomatis_RC_F_s_342_uid213391 | 0.42 |
| *Chlamydia* | Chlamydia_trachomatis_RC_F_s_852_uid213387 | 0.42 |
| *Thermoanaerobacter* | Thermoanaerobacterium_xylanolyticum_LX_11_uid63163 | 0.42 |
| *Acidimicrobium* | Acidimicrobium_ferrooxidans_DSM_10331_uid59215 | 0.42 |
| *Aminobacterium* | Aminobacterium_colombiense_DSM_12261_uid47083 | 0.42 |
| *Chlamydia* | Chlamydia_trachomatis_J_6276tet1_uid213394 | 0.419 |
| *Conexibacter* | Conexibacter_woesei_DSM_14684_uid43467 | 0.418 |
| *Pseudoalteromonas* | Pseudoalteromonas_SM9913_uid61247 | 0.417 |
| *Sphingopyxis* | Sphingopyxis_alaskensis_RB2256_uid58351 | 0.417 |
| *Mesorhizobium* | Mesorhizobium_opportunistum_WSM2075_uid40861 | 0.417 |
| *Mycobacterium* | Mycobacterium_abscessus_bolletii_50594_uid205422 | 0.417 |
| *Chlorobium* | Chlorobium_phaeobacteroides_DSM_266_uid58133 | 0.417 |
| *Gramella* | Gramella_forsetii_KT0803_uid58881 | 0.416 |
| *Advenella* | Advenella_kashmirensis_WT001_uid80859 | 0.416 |
| *Acholeplasma* | Acholeplasma_brassicae_uid222823 | 0.416 |
| *Cellulophaga* | Cellulophaga_lytica_DSM_7489_uid63401 | 0.416 |
| *Gloeobacter* | Gloeobacter_JS_uid225602 | 0.415 |
| *Mesorhizobium* | Mesorhizobium_loti_MAFF303099_uid57601 | 0.414 |
| *Brevibacillus* | Brevibacillus_brevis_NBRC_100599_uid59175 | 0.414 |
| *Ehrlichia* | Ehrlichia_ruminantium_Welgevonden_uid58013 | 0.413 |
| *Ehrlichia* | Ehrlichia_ruminantium_Welgevonden_uid58243 | 0.412 |
| *Marinobacter* | Marinobacter_adhaerens_HP15_uid162009 | 0.411 |
| *Fluviicola* | Fluviicola_taffensis_DSM_16823_uid65271 | 0.411 |
| *Gluconobacter* | Gluconobacter_oxydans_H24_uid179202 | 0.409 |
| *Helicobacter* | Helicobacter_pylori_908_uid159985 | 0.409 |
| *Helicobacter* | Helicobacter_pylori_2017_uid161151 | 0.408 |
| *Mycobacterium* | Mycobacterium_abscessus_uid61613 | 0.408 |
| *Bartonella* | Bartonella_quintana_Toulouse_uid57635 | 0.407 |
| *Synechococcus* | Synechococcus_CC9902_uid58323 | 0.406 |
| *Denitrovibrio* | Denitrovibrio_acetiphilus_DSM_12809_uid46657 | 0.405 |
| *Ehrlichia* | Ehrlichia_ruminantium_Gardel_uid58245 | 0.405 |
| *Calditerrivibrio* | Calditerrivibrio_nitroreducens_DSM_19672_uid60821 | 0.405 |
| *Mycobacterium* | Mycobacterium_tuberculosis_Haarlem3_NITR202_uid202216 | 0.405 |
| *Bartonella* | Bartonella_quintana_RM_11_uid174512 | 0.404 |
| *Shewanella* | Shewanella_oneidensis_MR_1_uid57949 | 0.404 |
| *Pelagibacterium* | Pelagibacterium_halotolerans_B2_uid74393 | 0.404 |
| *Parvibaculum* | Parvibaculum_lavamentivorans_DS_1_uid58739 | 0.402 |
| *Croceibacter* | Croceibacter_atlanticus_HTCC2559_uid49661 | 0.402 |
| *Helicobacter* | Helicobacter_pylori_2018_uid161159 | 0.402 |
| *Wolbachia* | Wolbachia_endosymbiont_of_Onchocerca_ochengi_uid171829 | 0.401 |
| *Fervidobacterium* | Fervidobacterium_nodosum_Rt17_B1_uid58625 | 0.401 |
| *Dinoroseobacter* | Dinoroseobacter_shibae_DFL_12_uid58707 | 0.4 |
| *Asticcacaulis* | Asticcacaulis_excentricus_CB_48_uid55641 | 0.399 |
| *Shewanella* | Shewanella_W3_18_1_uid58341 | 0.399 |
| *Bradyrhizobium* | Bradyrhizobium_BTAi1_uid58505 | 0.399 |
| *Chitinophaga* | Chitinophaga_pinensis_DSM_2588_uid59113 | 0.399 |
| *Rhizobium* | Rhizobium_NGR234_uid59081 | 0.399 |
| *Microbacterium* | Microbacterium_testaceum_StLB037_uid62789 | 0.397 |
| *Runella* | Runella_slithyformis_DSM_19594_uid68317 | 0.397 |
| *Nitrobacter* | Nitrobacter_winogradskyi_Nb_255_uid58295 | 0.397 |
| *Bartonella* | Bartonella_australis_Aust_NH1_uid189950 | 0.397 |
| *Sulfurovum* | Sulfurovum_NBC37_1_uid58863 | 0.396 |
| *Streptomyces* | Streptomyces_rapamycinicus_NRRL_5491_uid227224 | 0.395 |
| *Hippea* | Hippea_maritima_DSM_10411_uid65267 | 0.394 |
| *Maricaulis* | Maricaulis_maris_MCS10_uid58689 | 0.394 |
| *Candidatus_Midichloria* | Candidatus_Midichloria_mitochondrii_IricVA_uid68687 | 0.394 |
| *Wolbachia* | Wolbachia_endosymbiont_TRS_of_Brugia_malayi_uid58107 | 0.394 |
| *Shewanella* | Shewanella_putrefaciens_CN_32_uid58267 | 0.393 |
| *Methylophaga* | Methylophaga_JAM1_uid162947 | 0.393 |
| *Muricauda* | Muricauda_ruestringensis_DSM_13258_uid72479 | 0.393 |
| *Polynucleobacter* | Polynucleobacter_necessarius_STIR1_uid58967 | 0.393 |
| *Mycobacterium* | Mycobacterium_rhodesiae_NBB3_uid75107 | 0.393 |
| *Methylocystis* | Methylocystis_SC2_uid174072 | 0.392 |
| *Shewanella* | Shewanella_baltica_BA175_uid52601 | 0.391 |
| *Desulfobacca* | Desulfobacca_acetoxidans_DSM_11109_uid65785 | 0.391 |
| *Chlorobium* | Chlorobium_phaeobacteroides_BS1_uid58131 | 0.391 |
| *Methylotenera* | Methylotenera_301_uid49469 | 0.391 |
| *Wolbachia* | Wolbachia_endosymbiont_of_Drosophila_simulans_wNo_uid198767 | 0.39 |
| *Thermomonospora* | Thermomonospora_curvata_DSM_43183_uid41885 | 0.389 |
| *Xenorhabdus* | Xenorhabdus_bovienii_SS_2004_uid46345 | 0.389 |
| *Persephonella* | Persephonella_marina_EX_H1_uid58119 | 0.389 |
| *Methylophaga* | Methylophaga_JAM7_uid162949 | 0.387 |
| *Chelativorans* | Chelativorans_BNC1_uid58069 | 0.385 |
| *Wolbachia* | Wolbachia_endosymbiont_of_Drosophila_simulans_wHa_uid198768 | 0.385 |
| *Moraxella* | Moraxella_catarrhalis_BBH18_uid48809 | 0.384 |
| *Melioribacter* | Melioribacter_roseus_P3M_uid170941 | 0.383 |
| *Candidatus_Methylomirabilis* | Candidatus_Methylomirabilis_oxyfera_uid161981 | 0.381 |
| *Mycobacterium* | Mycobacterium_smegmatis_JS623_uid184820 | 0.381 |
| *Thermovibrio* | Thermovibrio_ammonificans_HB_1_uid62095 | 0.381 |
| *Lacinutrix* | Lacinutrix_5H_3_7_4_uid68067 | 0.381 |
| *Simiduia* | Simiduia_agarivorans_SA1_uid177713 | 0.381 |
| *Oligotropha* | Oligotropha_carboxidovorans_OM4_uid162135 | 0.38 |
| *Emticicia* | Emticicia_oligotrophica_DSM_17448_uid177079 | 0.38 |
| *Helicobacter* | Helicobacter_cetorum_MIT_99_5656_uid162215 | 0.38 |
| *Thermoanaerobacter* | Thermoanaerobacterium_thermosaccharolyticum_DSM_571_uid51639 | 0.38 |
| *Flexibacter* | Flexibacter_litoralis_DSM_6794_uid168257 | 0.38 |
| *Pelodictyon* | Pelodictyon_phaeoclathratiforme_BU_1_uid58173 | 0.379 |
| *Sanguibacter* | Sanguibacter_keddieii_DSM_10542_uid40845 | 0.379 |
| *Nocardiopsis* | Nocardiopsis_alba_ATCC_BAA_2165_uid174334 | 0.378 |
| *Phaeobacter* | Phaeobacter_gallaeciensis_DSM_17395_uid54717 | 0.377 |
| *Phaeobacter* | Phaeobacter_gallaeciensis_uid54715 | 0.377 |
| *Oligotropha* | Oligotropha_carboxidovorans_OM5_uid72795 | 0.377 |
| *Echinicola* | Echinicola_vietnamensis_DSM_17526_uid184076 | 0.376 |
| *Phaeobacter* | Phaeobacter_gallaeciensis_DSM_26640_uid232357 | 0.376 |
| *Shewanella* | Shewanella_putrefaciens_200_uid161927 | 0.375 |
| *Thermoanaerobacter* | Thermoanaerobacterium_thermosaccharolyticum_M0795_uid184821 | 0.374 |
| *Photorhabdus* | Photorhabdus_asymbiotica_ATCC_43949_uid59243 | 0.374 |
| *Thermovirga* | Thermovirga_lienii_DSM_17291_uid77129 | 0.373 |
| *Petrotoga* | Petrotoga_mobilis_SJ95_uid58747 | 0.373 |
| *Shewanella* | Shewanella_baltica_OS223_uid58775 | 0.372 |
| *Hyphomicrobium* | Hyphomicrobium_MC1_uid68453 | 0.372 |
| *Thermobifida* | Thermobifida_fusca_YX_uid57703 | 0.372 |
| *Helicobacter* | Helicobacter_hepaticus_ATCC_51449_uid57737 | 0.372 |
| *Rhodococcus* | Rhodococcus_equi_103S_uid60171 | 0.372 |
| *Kineococcus* | Kineococcus_radiotolerans_SRS30216_uid58067 | 0.371 |
| *Wolbachia* | Wolbachia_endosymbiont_of_Drosophila_melanogaster_uid57851 | 0.371 |
| *Sulfobacillus* | Sulfobacillus_acidophilus_DSM_10332_uid88061 | 0.371 |
| *Sulfurimonas* | Sulfurimonas_denitrificans_DSM_1251_uid58185 | 0.37 |
| *Shewanella* | Shewanella_baltica_OS678_uid50553 | 0.369 |
| *Coraliomargarita* | Coraliomargarita_akajimensis_DSM_45221_uid47079 | 0.369 |
| *Xenorhabdus* | Xenorhabdus_nematophila_ATCC_19061_uid49133 | 0.369 |
| *Hyphomonas* | Hyphomonas_neptunium_ATCC_15444_uid58433 | 0.368 |
| *Oligotropha* | Oligotropha_carboxidovorans_OM5_uid59155 | 0.367 |
| *Nitrosomonas* | Nitrosomonas_eutropha_C91_uid58363 | 0.367 |
| *Helicobacter* | Helicobacter_cetorum_MIT_00_7128_uid162217 | 0.366 |
| *Shewanella* | Shewanella_baltica_OS195_uid58261 | 0.365 |
| *Myxococcus* | Myxococcus_stipitatus_DSM_14675_uid186549 | 0.365 |
| *Candidatus_Hamiltonella* | Candidatus_Hamiltonella_defensa_5AT__Acyrthosiphon_pisum__uid59289 | 0.365 |
| *Thermobispora* | Thermobispora_bispora_DSM_43833_uid48999 | 0.365 |
| *Azotobacter* | Azotobacter_vinelandii_CA6_uid198830 | 0.364 |
| *Nitrosococcus* | Nitrosococcus_watsonii_C_113_uid50331 | 0.363 |
| *Shewanella* | Shewanella_baltica_OS185_uid58743 | 0.363 |
| *Azotobacter* | Azotobacter_vinelandii_DJ_uid57597 | 0.362 |
| *Azotobacter* | Azotobacter_vinelandii_CA_uid198829 | 0.362 |
| *Leisingera* | Leisingera_methylohalidivorans_DSM_14336_uid232356 | 0.361 |
| *Mycobacterium* | Mycobacterium_kansasii_ATCC_12478_uid55385 | 0.361 |
| *Sulfobacillus* | Sulfobacillus_acidophilus_TPY_uid68841 | 0.361 |
| *Nocardiopsis* | Nocardiopsis_dassonvillei_DSM_43111_uid49483 | 0.36 |
| *Bartonella* | Bartonella_henselae_Houston_1_uid57745 | 0.36 |
| *Ignavibacterium* | Ignavibacterium_album_JCM_16511_uid162097 | 0.36 |
| *Haliangium* | Haliangium_ochraceum_DSM_14365_uid41425 | 0.36 |
| *Prochlorococcus* | Prochlorococcus_marinus_CCMP1375_uid57995 | 0.36 |
| *Shewanella* | Shewanella_baltica_OS117_uid162025 | 0.359 |
| *Chloroherpeton* | Chloroherpeton_thalassium_ATCC_35110_uid59187 | 0.359 |
| *Thermomicrobium* | Thermomicrobium_roseum_DSM_5159_uid59341 | 0.357 |
| *Intrasporangium* | Intrasporangium_calvum_DSM_43043_uid61729 | 0.357 |
| *Anaerobaculum* | Anaerobaculum_mobile_DSM_13181_uid168323 | 0.356 |
| *Nitrosococcus* | Nitrosococcus_oceani_ATCC_19707_uid58403 | 0.356 |
| *Thermotoga* | Thermotoga_RQ2_uid58935 | 0.355 |
| *Frankia* | Frankia_symbiont_of_Datisca_glomerata_uid46257 | 0.352 |
| *Nitrosospira* | Nitrosospira_multiformis_ATCC_25196_uid58361 | 0.351 |
| *Mycoplasma* | Mycoplasma_haemocanis_Illinois_uid82367 | 0.35 |
| *Alcanivorax* | Alcanivorax_borkumensis_SK2_uid58169 | 0.347 |
| *Aequorivita* | Aequorivita_sublithincola_DSM_14238_uid168181 | 0.347 |
| *Rhodococcus* | Rhodococcus_pyridinivorans_SB3094_uid232359 | 0.346 |
| *Methylotenera* | Methylotenera_mobilis_JLW8_uid59373 | 0.346 |
| *Mycobacterium* | Mycobacterium_ulcerans_Agy99_uid62939 | 0.345 |
| *Sphingomonas* | Sphingomonas_wittichii_RW1_uid58691 | 0.345 |
| *Ruegeria* | Ruegeria_TM1040_uid58193 | 0.345 |
| *Thermoanaerobacter* | Thermoanaerobacter_tengcongensis_MB4_uid57813 | 0.345 |
| *Acidothermus* | Acidothermus_cellulolyticus_11B_uid58501 | 0.344 |
| *Flexistipes* | Flexistipes_sinusarabici_DSM_4947_uid68147 | 0.344 |
| *Photorhabdus* | Photorhabdus_luminescens_laumondii_TTO1_uid61593 | 0.344 |
| *Zunongwangia* | Zunongwangia_profunda_SM_A87_uid48073 | 0.344 |
| *Zymomonas* | Zymomonas_mobilis_pomaceae_ATCC_29192_uid68445 | 0.343 |
| *Prochlorococcus* | Prochlorococcus_marinus_NATL1A_uid58423 | 0.343 |
| *Terriglobus* | Terriglobus_roseus_DSM_18391_uid168183 | 0.342 |
| *Prochlorococcus* | Prochlorococcus_marinus_NATL2A_uid58359 | 0.342 |
| *Belliella* | Belliella_baltica_DSM_15883_uid168182 | 0.342 |
| *Synechococcus* | Synechococcus_PCC_7002_uid59137 | 0.341 |
| *Gemmatimonas* | Gemmatimonas_aurantiaca_T_27_uid58813 | 0.341 |
| *Nitrobacter* | Nitrobacter_hamburgensis_X14_uid58293 | 0.338 |
| *Frankia* | Frankia_CcI3_uid58397 | 0.338 |
| *Shewanella* | Shewanella_baltica_OS155_uid58259 | 0.338 |
| *Geitlerinema* | Geitlerinema_PCC_7407_uid183007 | 0.337 |
| *Wolbachia* | Wolbachia_endosymbiont_of_Culex_quinquefasciatus_Pel_uid61645 | 0.337 |
| *Mycobacterium* | Mycobacterium_liflandii_128FXT_uid59005 | 0.335 |
| *Mycoplasma* | Candidatus_Mycoplasma_haemolamae_Purdue_uid171259 | 0.335 |
| *Wolbachia* | Wolbachia_wRi_uid59371 | 0.335 |
| *Desulfobacterium* | Desulfobacterium_autotrophicum_HRM2_uid59061 | 0.335 |
| *Marinobacter* | Marinobacter_BSs20148_uid171995 | 0.333 |
| *Thermotoga* | Thermotoga_petrophila_RKU_1_uid58655 | 0.333 |
| *Hydrogenobaculum* | Hydrogenobaculum_SN_uid46251 | 0.332 |
| *Leptospirillum* | Leptospirillum_ferriphilum_ML_04_uid175904 | 0.331 |
| *Helicobacter* | Helicobacter_cinaedi_PAGU611_uid162219 | 0.331 |
| *Bartonella* | Bartonella_tribocorum_CIP_105476_uid59129 | 0.331 |
| *Cellulophaga* | Cellulophaga_algicola_DSM_14237_uid62159 | 0.33 |
| *Beutenbergia* | Beutenbergia_cavernae_DSM_12333_uid59047 | 0.33 |
| *Bartonella* | Bartonella_grahamii_as4aup_uid59405 | 0.329 |
| *Synechococcus* | Synechococcus_CC9311_uid58123 | 0.329 |
| *Krokinobacter* | Krokinobacter_4H_3_7_5_uid66593 | 0.328 |
| *Hydrogenobaculum* | Hydrogenobaculum_HO_uid190882 | 0.327 |
| *Polynucleobacter* | Polynucleobacter_necessarius_asymbioticus_QLW_P1DMWA_1_uid58611 | 0.327 |
| *Coprothermobacter* | Coprothermobacter_proteolyticus_DSM_5265_uid59253 | 0.327 |
| *Spirosoma* | Spirosoma_linguale_DSM_74_uid43413 | 0.326 |
| *Mycobacterium* | Mycobacterium_marinum_M_uid59423 | 0.325 |
| *Methylocella* | Methylocella_silvestris_BL2_uid59433 | 0.325 |
| *Niastella* | Niastella_koreensis_GR20_10_uid83125 | 0.324 |
| *Magnetococcus* | Magnetococcus_MC_1_uid57833 | 0.323 |
| *Thermotoga* | Thermotoga_maritima_MSB8_uid202924 | 0.323 |
| *Thermotoga* | Thermotoga_maritima_MSB8_uid57723 | 0.322 |
| *Thermotoga* | Thermotoga_maritima_MSB8_uid179902 | 0.322 |
| *Bdellovibrio* | Bdellovibrio_bacteriovorus_HD100_uid61595 | 0.321 |
| *Methylomonas* | Methylomonas_methanica_MC09_uid67363 | 0.321 |
| *Sulfuricurvum* | Sulfuricurvum_kujiense_DSM_16994_uid60789 | 0.32 |
| *Thermotoga* | Thermotoga_neapolitana_DSM_4359_uid59065 | 0.317 |
| *Thermotoga* | Thermotoga_naphthophila_RKU_10_uid42777 | 0.316 |
| *Shewanella* | Shewanella_sediminis_HAW_EB3_uid58835 | 0.316 |
| *Nocardioides* | Nocardioides_JS614_uid58149 | 0.315 |
| *Hydrogenobaculum* | Hydrogenobaculum_Y04AAS1_uid58857 | 0.313 |
| *Shewanella* | Shewanella_denitrificans_OS217_uid58263 | 0.311 |
| *Shewanella* | Shewanella_pealeana_ATCC_700345_uid58705 | 0.31 |
| *Psychrobacter* | Psychrobacter_PRwf_1_uid58459 | 0.31 |
| *Frankia* | Frankia_alni_ACN14a_uid58695 | 0.309 |
| *Chlorobium* | Chlorobium_chlorochromatii_CaD3_uid58375 | 0.308 |
| *Chlamydia* | Chlamydia_trachomatis_RC_L2_s_46_uid213386 | 0.307 |
| *Chlamydia* | Chlamydia_trachomatis_RC_L2_55_uid213396 | 0.307 |
| *Mycobacterium* | Mycobacterium_leprae_TN_uid57697 | 0.307 |
| *Mycobacterium* | Mycobacterium_leprae_Br4923_uid59293 | 0.306 |
| *Methylomicrobium* | Methylomicrobium_alcaliphilum_uid77119 | 0.305 |
| *Chlamydia* | Chlamydia_trachomatis_RC_L2_s_3_uid213390 | 0.305 |
| *Marivirga* | Marivirga_tractuosa_DSM_4126_uid60837 | 0.305 |
| *Saprospira* | Saprospira_grandis_Lewin_uid89375 | 0.305 |
| *Chlamydia* | Chlamydia_trachomatis_L2c_uid68843 | 0.304 |
| *Chlamydia* | Chlamydia_trachomatis_434_Bu_uid61633 | 0.304 |
| *Chlamydia* | Chlamydia_trachomatis_L2_434_Bu_f__uid198644 | 0.304 |
| *Chlamydia* | Chlamydia_trachomatis_L2_434_Bu_i__uid198643 | 0.304 |
| *Desulfotalea* | Desulfotalea_psychrophila_LSv54_uid58153 | 0.304 |
| *Chlamydia* | Chlamydia_trachomatis_L2b_UCH_1_proctitis_uid61635 | 0.304 |
| *Chlamydia* | Chlamydia_trachomatis_RC_J_966_uid213393 | 0.303 |
| *Shewanella* | Shewanella_frigidimarina_NCIMB_400_uid58265 | 0.303 |
| *Fervidobacterium* | Fervidobacterium_pennivorans_DSM_9078_uid78143 | 0.302 |
| *Chlamydia* | Chlamydia_trachomatis_RC_J_943_uid213388 | 0.302 |
| *Chlamydia* | Chlamydia_muridarum_Nigg_uid57785 | 0.302 |
| *Helicobacter* | Helicobacter_cinaedi_ATCC_BAA_847_uid193765 | 0.301 |
| *Segniliparus* | Segniliparus_rotundus_DSM_44985_uid49049 | 0.299 |
| *Prochlorococcus* | Prochlorococcus_marinus_MIT_9211_uid58309 | 0.299 |
| *Shewanella* | Shewanella_violacea_DSS12_uid47085 | 0.299 |
| *Shewanella* | Shewanella_halifaxensis_HAW_EB4_uid59007 | 0.298 |
| *Zobellia* | Zobellia_galactanivorans_uid70621 | 0.298 |
| *Haliscomenobacter* | Haliscomenobacter_hydrossis_DSM_1100_uid66777 | 0.298 |
| *Actinoplanes* | Actinoplanes_missouriensis_431_uid158169 | 0.298 |
| *Mycoplasma* | Mycoplasma_haemofelis_Langford_1_uid62461 | 0.297 |
| *Rhodococcus* | Rhodococcus_erythropolis_CCM2595_uid216088 | 0.297 |
| *Sulfurospirillum* | Sulfurospirillum_deleyianum_DSM_6946_uid41861 | 0.296 |
| *Bdellovibrio* | Bdellovibrio_bacteriovorus_Tiberius_uid182482 | 0.296 |
| *Cyanobacterium* | Cyanobacterium_aponinum_PCC_10605_uid183340 | 0.296 |
| *Helicobacter* | Helicobacter_felis_ATCC_49179_uid61409 | 0.295 |
| *Chlamydia* | Chlamydia_trachomatis_D_UW_3_CX_uid57637 | 0.295 |
| *Chlamydia* | Chlamydia_trachomatis_D_LC_uid159879 | 0.295 |
| *Chlamydia* | Chlamydia_trachomatis_D_EC_uid159881 | 0.295 |
| *Chlamydia* | Chlamydia_trachomatis_D_SotonD5_uid196773 | 0.295 |
| *Chlamydia* | Chlamydia_trachomatis_G_SotonG1_uid196779 | 0.295 |
| *Chlamydia* | Chlamydia_trachomatis_K_SotonK1_uid196782 | 0.295 |
| *Chlamydia* | Chlamydia_trachomatis_D_SotonD6_uid196774 | 0.295 |
| *Chthonomonas* | Chthonomonas_calidirosea_T49_uid208678 | 0.294 |
| *Coxiella* | Coxiella_burnetii_RSA_493_uid57631 | 0.294 |
| *Jonesia* | Jonesia_denitrificans_DSM_20603_uid59053 | 0.293 |
| *Chlamydia* | Chlamydia_trachomatis_RC_J_971_uid213395 | 0.293 |
| *Candidatus_Puniceispirillum* | Candidatus_Puniceispirillum_marinum_IMCC1322_uid47081 | 0.293 |
| *Chlamydia* | Chlamydia_trachomatis_L2b_Canada1_uid196798 | 0.293 |
| *Chlamydia* | Chlamydia_trachomatis_L2b_Ams3_uid196794 | 0.293 |
| *Chlamydia* | Chlamydia_trachomatis_L2b_795_uid196791 | 0.293 |
| *Chlamydia* | Chlamydia_trachomatis_L1_224_uid196785 | 0.292 |
| *Chlamydia* | Chlamydia_trachomatis_L2b_Ams5_uid196796 | 0.292 |
| *Chlamydia* | Chlamydia_trachomatis_L2_25667R_uid196786 | 0.292 |
| *Chlamydia* | Chlamydia_trachomatis_L2b_8200_07_uid196787 | 0.292 |
| *Chlamydia* | Chlamydia_trachomatis_L2b_Ams2_uid196793 | 0.292 |
| *Chlamydia* | Chlamydia_trachomatis_L2b_Ams4_uid196795 | 0.292 |
| *Chlamydia* | Chlamydia_trachomatis_L2b_UCH_2_uid196788 | 0.292 |
| *Chlamydia* | Chlamydia_trachomatis_L2b_LST_uid196789 | 0.292 |
| *Chlamydia* | Chlamydia_trachomatis_L2b_Ams1_uid196792 | 0.292 |
| *Chlamydia* | Chlamydia_trachomatis_L1_115_uid196784 | 0.292 |
| *Chlamydia* | Chlamydia_trachomatis_L2b_CV204_uid196790 | 0.292 |
| *Chlamydia* | Chlamydia_trachomatis_L1_440_LN_uid196783 | 0.292 |
| *Chlamydia* | Chlamydia_trachomatis_L3_404_LN_uid196797 | 0.292 |
| *Chlamydia* | Chlamydia_trachomatis_uid196800 | 0.292 |
| *Chlamydia* | Chlamydia_trachomatis_RC_F_69_uid213385 | 0.292 |
| *Chlamydia* | Chlamydia_trachomatis_RC_J_953_uid213389 | 0.292 |
| *Coxiella* | Coxiella_burnetii_CbuG_Q212_uid58893 | 0.291 |
| *Roseiflexus* | Roseiflexus_castenholzii_DSM_13941_uid58287 | 0.291 |
| *Thiomicrospira* | Thiomicrospira_crunogena_XCL_2_uid58183 | 0.29 |
| *Chlamydia* | Chlamydia_trachomatis_E_150_uid161403 | 0.29 |
| *Chlamydia* | Chlamydia_trachomatis_E_11023_uid161369 | 0.29 |
| *Chlamydia* | Chlamydia_trachomatis_E_Bour_uid196775 | 0.29 |
| *Chlamydia* | Chlamydia_trachomatis_E_SotonE4_uid196776 | 0.29 |
| *Chlamydia* | Chlamydia_trachomatis_E_C599_uid222812 | 0.29 |
| *Chlamydia* | Chlamydia_trachomatis_Sweden2_uid161995 | 0.29 |
| *Chlamydia* | Chlamydia_trachomatis_E_SW3_uid167483 | 0.29 |
| *Chlamydia* | Chlamydia_trachomatis_D_SotonD1_uid196772 | 0.29 |
| *Chlamydia* | Chlamydia_trachomatis_F_SWFPminus_uid222813 | 0.29 |
| *Chlamydia* | Chlamydia_trachomatis_F_SW5_uid167485 | 0.29 |
| *Chlamydia* | Chlamydia_trachomatis_F_SW4_uid167484 | 0.29 |
| *Sulfuricurvum* | uncultured_Sulfuricurvum_RIFRC_1_uid193658 | 0.29 |
| *Chlamydia* | Chlamydia_trachomatis_uid196778 | 0.29 |
| *Corallococcus* | Corallococcus_coralloides_DSM_2259_uid157997 | 0.29 |
| *Nitratiruptor* | Nitratiruptor_SB155_2_uid58861 | 0.289 |
| *Chlamydia* | Chlamydia_trachomatis_A_5291_uid196770 | 0.288 |
| *Chlamydia* | Chlamydia_trachomatis_A_363_uid196769 | 0.288 |
| *Chlamydia* | Chlamydia_trachomatis_A2497_uid159863 | 0.288 |
| *Chlamydia* | Chlamydia_trachomatis_A2497_uid159993 | 0.288 |
| *Chlamydia* | Chlamydia_trachomatis_A_7249_uid196771 | 0.288 |
| *Chlamydia* | Chlamydia_trachomatis_E_SotonE8_uid196777 | 0.288 |
| *Psychromonas* | Psychromonas_CNPT3_uid54249 | 0.288 |
| *Chlamydia* | Chlamydia_trachomatis_G_11222_uid161361 | 0.288 |
| *Chlamydia* | Chlamydia_trachomatis_G_9768_uid161353 | 0.288 |
| *Chlamydia* | Chlamydia_trachomatis_G_9301_uid161377 | 0.288 |
| *Chlamydia* | Chlamydia_trachomatis_G_11074_uid161409 | 0.288 |
| *Chlamydia* | Chlamydia_trachomatis_uid216090 | 0.287 |
| *Chlamydia* | Chlamydia_trachomatis_Ia_SotonIa1_uid196780 | 0.287 |
| *Chlamydia* | Chlamydia_trachomatis_uid196781 | 0.287 |
| *Roseiflexus* | Roseiflexus_RS_1_uid58523 | 0.287 |
| *Chlamydia* | Chlamydia_trachomatis_C_TW_3_uid232251 | 0.287 |
| *Chlamydia* | Chlamydia_trachomatis_B_Jali20_OT_uid59351 | 0.287 |
| *Coxiella* | Coxiella_burnetii_CbuK_Q154_uid58895 | 0.286 |
| *Wolinella* | Wolinella_succinogenes_DSM_1740_uid61591 | 0.285 |
| *Chlamydia* | Chlamydia_trachomatis_IU824_uid193712 | 0.285 |
| *Chlamydia* | Chlamydia_trachomatis_IU888_uid193713 | 0.285 |
| *Thermotoga* | Thermotoga_elfii_NBRC_107921_uid227422 | 0.285 |
| *Thermotoga* | Thermotoga_lettingae_TMO_uid58419 | 0.285 |
| *Helicobacter* | Helicobacter_bizzozeronii_CIII_1_uid68141 | 0.285 |
| *Parvularcula* | Parvularcula_bermudensis_HTCC2503_uid51641 | 0.284 |
| *Chlamydia* | Chlamydia_trachomatis_B_TZ1A828_OT_uid59349 | 0.284 |
| *Chlamydia* | Chlamydia_trachomatis_A_HAR_13_uid58333 | 0.283 |
| *Chlamydia* | Chlamydia_trachomatis_L2b_Canada2_uid196799 | 0.283 |
| *Neorickettsia* | Neorickettsia_risticii_Illinois_uid58889 | 0.282 |
| *Granulicella* | Granulicella_mallensis_MP5ACTX8_uid49957 | 0.282 |
| *Nocardia* | Nocardia_farcinica_IFM_10152_uid58203 | 0.282 |
| *Thioalkalimicrobium* | Thioalkalimicrobium_cyclicum_ALM1_uid67391 | 0.281 |
| *Thermodesulfatator* | Thermodesulfatator_indicus_DSM_15286_uid68285 | 0.281 |
| *Nitrosomonas* | Nitrosomonas_Is79A3_uid68745 | 0.281 |
| *Candidatus_Koribacter* | Candidatus_Koribacter_versatilis_Ellin345_uid58479 | 0.281 |
| *Thermobaculum* | Thermobaculum_terrenum_ATCC_BAA_798_uid42011 | 0.28 |
| *Frankia* | Frankia_EuI1c_uid42615 | 0.278 |
| *Legionella* | Legionella_pneumophila_Hextuple_2q_uid197191 | 0.278 |
| *Rhodococcus* | Rhodococcus_erythropolis_PR4_uid59019 | 0.278 |
| *Legionella* | Legionella_pneumophila_Hextuple_3a_uid197192 | 0.276 |
| *Leptospirillum* | Leptospirillum_ferrooxidans_C2_3_uid158171 | 0.275 |
| *Nitrospira* | Candidatus_Nitrospira_defluvii_uid51175 | 0.275 |
| *Tsukamurella* | Tsukamurella_paurometabola_DSM_20162_uid48829 | 0.274 |
| *Coxiella* | Coxiella_burnetii_RSA_331_uid58637 | 0.274 |
| *Roseobacter* | Roseobacter_denitrificans_OCh_114_uid58597 | 0.273 |
| *Terriglobus* | Terriglobus_saanensis_SP1PR4_uid53251 | 0.272 |
| *Stigmatella* | Stigmatella_aurantiaca_DW4_3_1_uid158509 | 0.271 |
| *Coxiella* | Coxiella_burnetii_Dugway_5J108_111_uid58629 | 0.271 |
| *Turneriella* | Turneriella_parva_DSM_21527_uid168321 | 0.271 |
| *Desulfocapsa* | Desulfocapsa_sulfexigens_DSM_10523_uid189952 | 0.27 |
| *Actinoplanes* | Actinoplanes_N902_109_uid202219 | 0.269 |
| *Kangiella* | Kangiella_koreensis_DSM_16069_uid59209 | 0.269 |
| *Rhodococcus* | Rhodococcus_opacus_B4_uid13791 | 0.269 |
| *Synechococcus* | Synechococcus_PCC_7502_uid183008 | 0.268 |
| *Neorickettsia* | Neorickettsia_sennetsu_Miyayama_uid57965 | 0.268 |
| *Aliivibrio* | Aliivibrio_salmonicida_LFI1238_uid59251 | 0.268 |
| *Jannaschia* | Jannaschia_CCS1_uid58147 | 0.267 |
| *Cellvibrio* | Cellvibrio_japonicus_Ueda107_uid59139 | 0.267 |
| *Shewanella* | Shewanella_piezotolerans_WP3_uid58745 | 0.267 |
| *Helicobacter* | Helicobacter_heilmannii_ASB1_4_uid182935 | 0.266 |
| *Kosmotoga* | Kosmotoga_olearia_TBF_19_5_1_uid59205 | 0.266 |
| *Mycoplasma* | Mycoplasma_haemofelis_Ohio2_uid162029 | 0.265 |
| *Nitrosomonas* | Nitrosomonas_AL212_uid55727 | 0.265 |
| *Xylella* | Xylella_fastidiosa_9a5c_uid57849 | 0.264 |
| *Cycloclasticus* | Cycloclasticus_P1_uid176368 | 0.264 |
| *Kitasatospora* | Kitasatospora_setae_KM_6054_uid77027 | 0.261 |
| *Sorangium* | Sorangium_cellulosum_So0157_2_uid210741 | 0.26 |
| *Pseudovibrio* | Pseudovibrio_FO_BEG1_uid82373 | 0.26 |
| *Sulfurospirillum* | Sulfurospirillum_barnesii_SES_3_uid168117 | 0.26 |
| *Frankia* | Frankia_EAN1pec_uid58367 | 0.26 |
| *Nakamurella* | Nakamurella_multipartita_DSM_44233_uid59221 | 0.259 |
| *Desulfobacula* | Desulfobacula_toluolica_Tol2_uid175777 | 0.258 |
| *Shewanella* | Shewanella_woodyi_ATCC_51908_uid58721 | 0.258 |
| *Cyanobacterium* | Cyanobacterium_stanieri_PCC_7202_uid183337 | 0.258 |
| *Leptospira* | Leptospira_borgpetersenii_serovar_Hardjo_bovis_L550_uid58507 | 0.258 |
| *Sorangium* | Sorangium_cellulosum__So_ce_56__uid61629 | 0.258 |
| *Candidatus_Solibacter* | Candidatus_Solibacter_usitatus_Ellin6076_uid58139 | 0.256 |
| *Spirochaeta* | Spirochaeta_coccoides_DSM_17374_uid66331 | 0.255 |
| *Rhodococcus* | Rhodococcus_jostii_RHA1_uid58325 | 0.255 |
| *Candidatus_Amoebophilus* | Candidatus_Amoebophilus_asiaticus_5a2_uid58963 | 0.253 |
| *Xylella* | Xylella_fastidiosa_M12_uid58763 | 0.253 |
| *Actinoplanes* | Actinoplanes_friuliensis_DSM_7358_uid226110 | 0.252 |
| *Thermosynechococcus* | Thermosynechococcus_elongatus_BP_1_uid57907 | 0.252 |
| *Idiomarina* | Idiomarina_loihiensis_L2TR_uid58087 | 0.252 |
| *Idiomarina* | Idiomarina_loihiensis_GSL_199_uid205256 | 0.252 |
| *Mannheimia* | Mannheimia_succiniciproducens_MBEL55E_uid58197 | 0.251 |
| *Bacteriovorax* | Bacteriovorax_marinus_SJ_uid82341 | 0.251 |
| *Beijerinckia* | Beijerinckia_indica_ATCC_9039_uid59057 | 0.25 |
| *Nocardia* | Nocardia_cyriacigeorgica_GUH_2_uid89395 | 0.249 |
| *Actinoplanes* | Actinoplanes_SE50_110_uid162333 | 0.248 |
| *Gordonia* | Gordonia_KTR9_uid174812 | 0.245 |
| *Psychroflexus* | Psychroflexus_torquis_ATCC_700755_uid54205 | 0.245 |
| *Photobacterium* | Photobacterium_profundum_SS9_uid62923 | 0.244 |
| *Xylella* | Xylella_fastidiosa_Temecula1_uid57869 | 0.244 |
| *Mesotoga* | Mesotoga_prima_MesG1_Ag_4_2_uid52599 | 0.244 |
| *Prochlorococcus* | Prochlorococcus_marinus_MIT_9313_uid57773 | 0.244 |
| *Cycloclasticus* | Cycloclasticus_zancles_7_ME_uid214092 | 0.243 |
| *Xylella* | Xylella_fastidiosa_M23_uid58809 | 0.242 |
| *Stackebrandtia* | Stackebrandtia_nassauensis_DSM_44728_uid46663 | 0.242 |
| *Thermosynechococcus* | Thermosynechococcus_NK55_uid231517 | 0.241 |
| *Xylella* | Xylella_fastidiosa_GB514_uid162023 | 0.241 |
| *Chlorobium* | Chlorobium_tepidum_TLS_uid57897 | 0.241 |
| *Leptospira* | Leptospira_borgpetersenii_serovar_Hardjo_bovis_JB197_uid58509 | 0.241 |
| *Salinispora* | Salinispora_tropica_CNB_440_uid58565 | 0.239 |
| *Blastococcus* | Blastococcus_saxobsidens_DD2_uid89391 | 0.237 |
| *Chlamydia* | Chlamydia_psittaci_WC_uid175577 | 0.235 |
| *Legionella* | Legionella_pneumophila_Lens_uid58209 | 0.235 |
| *Thermotoga* | Thermotoga_hypogea_NBRC_106472_uid227423 | 0.234 |
| *Gordonia* | Gordonia_bronchialis_DSM_43247_uid41403 | 0.232 |
| *Gordonia* | Gordonia_polyisoprenivorans_VH2_uid86651 | 0.232 |
| *Orientia* | Orientia_tsutsugamushi_Ikeda_uid58869 | 0.231 |
| *Saccharomonospora* | Saccharomonospora_viridis_DSM_43017_uid59055 | 0.23 |
| *Chlorobium* | Chlorobium_luteolum_DSM_273_uid58175 | 0.23 |
| *Microlunatus* | Microlunatus_phosphovorus_NM_1_uid68055 | 0.229 |
| *Legionella* | Legionella_pneumophila_LPE509_uid193710 | 0.228 |
| *Chlamydia* | Chlamydia_psittaci_VS225_uid175574 | 0.228 |
| *Chlamydia* | Chlamydia_psittaci_M56_uid175576 | 0.228 |
| *Bdellovibrio* | Bdellovibrio_exovorus_JSS_uid194119 | 0.228 |
| *Chlamydia* | Chlamydia_pecorum_P787_uid221292 | 0.227 |
| *Streptosporangium* | Streptosporangium_roseum_DSM_43021_uid42521 | 0.227 |
| *Legionella* | Legionella_pneumophila_Philadelphia_1_uid57609 | 0.227 |
| *Chlamydia* | Chlamydia_psittaci_GR9_uid175572 | 0.226 |
| *Modestobacter* | Modestobacter_marinus_uid167487 | 0.226 |
| *Chlamydia* | Chlamydia_psittaci_84_55_uid175571 | 0.225 |
| *Legionella* | Legionella_pneumophila_ATCC_43290_uid86885 | 0.225 |
| *Chlamydia* | Chlamydia_psittaci_NJ1_uid175579 | 0.224 |
| *Chlamydia* | Chlamydia_pecorum_W73_uid221291 | 0.224 |
| *Anaplasma* | Anaplasma_marginale_Dawn_uid226994 | 0.223 |
| *Anaplasma* | Anaplasma_marginale_Maries_uid57629 | 0.223 |
| *Prochlorococcus* | Prochlorococcus_marinus_MIT_9303_uid58305 | 0.223 |
| *Thermotoga* | Thermotoga_thermarum_DSM_5069_uid68449 | 0.222 |
| *Chlamydia* | Chlamydia_psittaci_WS_RT_E30_uid175575 | 0.222 |
| *Chloroflexus* | Chloroflexus_Y_400_fl_uid59085 | 0.221 |
| *Anaplasma* | Anaplasma_marginale_Gypsy_Plains_uid227217 | 0.22 |
| *Chlamydia* | Chlamydia_psittaci_CP3_uid175578 | 0.22 |
| *Cyclobacterium* | Cyclobacterium_marinum_DSM_745_uid71485 | 0.22 |
| *Legionella* | Legionella_pneumophila_Paris_uid58211 | 0.22 |
| *Chlamydia* | Chlamydia_psittaci_01DC12_uid179070 | 0.219 |
| *Legionella* | Legionella_pneumophila_2300_99_Alcoy_uid48801 | 0.219 |
| *Chloroflexus* | Chloroflexus_aurantiacus_J_10_fl_uid57657 | 0.218 |
| *Anaplasma* | Anaplasma_centrale_Israel_uid42155 | 0.218 |
| *Anaplasma* | Anaplasma_marginale_Florida_uid58577 | 0.218 |
| *Aquifex* | Aquifex_aeolicus_VF5_uid57765 | 0.217 |
| *Legionella* | Legionella_pneumophila_uid170534 | 0.217 |
| *Chlamydia* | Chlamydia_psittaci_MN_uid175573 | 0.217 |
| *Salinispora* | Salinispora_arenicola_CNS_205_uid58659 | 0.216 |
| *Roseobacter* | Roseobacter_litoralis_Och_149_uid54719 | 0.215 |
| *Planctomyces* | Planctomyces_brasiliensis_DSM_5305_uid60583 | 0.214 |
| *Orientia* | Orientia_tsutsugamushi_Boryong_uid61621 | 0.214 |
| *Kribbella* | Kribbella_flavida_DSM_17836_uid43465 | 0.213 |
| *Isosphaera* | Isosphaera_pallida_ATCC_43644_uid62207 | 0.213 |
| *Waddlia* | Waddlia_chondrophila_WSU_86_1044_uid49531 | 0.213 |
| *Psychromonas* | Psychromonas_ingrahamii_37_uid58521 | 0.213 |
| *Alteromonas* | Alteromonas_macleodii__Aegean_Sea_MED64__uid231689 | 0.213 |
| *Thalassolituus* | Thalassolituus_oleivorans_MIL_1_uid195604 | 0.212 |
| *Legionella* | Legionella_pneumophila_Lorraine_uid170535 | 0.21 |
| *Alteromonas* | Alteromonas_macleodii__Ionian_Sea_UM4b__uid210784 | 0.209 |
| *Chlamydia* | Chlamydia_pecorum_PV3056_3_uid221290 | 0.209 |
| *Alteromonas* | Alteromonas_macleodii_AltDE1_uid179068 | 0.208 |
| *Alteromonas* | Alteromonas_macleodii__Ionian_Sea_UM7__uid210783 | 0.208 |
| *Nitrosococcus* | Nitrosococcus_halophilus_Nc4_uid46803 | 0.207 |
| *Legionella* | Legionella_pneumophila_Thunder_Bay_uid206517 | 0.206 |
| *Legionella* | Legionella_pneumophila_Corby_uid58733 | 0.206 |
| *Alteromonas* | Alteromonas_macleodii__Ionian_Sea_U4__uid210780 | 0.205 |
| *Alteromonas* | Alteromonas_macleodii__English_Channel_615__uid210781 | 0.204 |
| *Chloroflexus* | Chloroflexus_aggregans_DSM_9485_uid58621 | 0.204 |
| *Legionella* | Legionella_longbeachae_NSW150_uid46099 | 0.204 |
| *Verrucosispora* | Verrucosispora_maris_AB_18_032_uid66297 | 0.202 |
| *Geodermatophilus* | Geodermatophilus_obscurus_DSM_43160_uid43725 | 0.202 |
| *Synechocystis* | Synechocystis_PCC_6803_uid189748 | 0.202 |
| *Synechocystis* | Synechocystis_PCC_6803_substr__GT_I_uid157913 | 0.202 |
| *Synechocystis* | Synechocystis_PCC_6803_substr__GT_I_uid158059 | 0.202 |
| *Synechocystis* | Synechocystis_PCC_6803_substr__PCC_N_uid159835 | 0.202 |
| *Synechocystis* | Synechocystis_PCC_6803_uid159873 | 0.202 |
| *Catenulispora* | Catenulispora_acidiphila_DSM_44928_uid59077 | 0.202 |
| *Synechocystis* | Synechocystis_PCC_6803_uid57659 | 0.202 |
| *Alteromonas* | Alteromonas_macleodii__Balearic_Sea_AD45__uid176366 | 0.201 |
| *Alteromonas* | Alteromonas_macleodii__Black_Sea_11__uid176365 | 0.199 |
| *Alteromonas* | Alteromonas_macleodii__English_Channel_673__uid176367 | 0.198 |
| *Alteromonas* | Alteromonas_macleodii__Ionian_Sea_U7__uid210785 | 0.198 |
| *Alteromonas* | Alteromonas_macleodii__Ionian_Sea_U8__uid210782 | 0.197 |
| *Alteromonas* | Alteromonas_macleodii__Deep_ecotype__uid58251 | 0.197 |
| *Hahella* | Hahella_chejuensis_KCTC_2396_uid58483 | 0.196 |
| *Alteromonas* | Alteromonas_macleodii_ATCC_27126_uid55253 | 0.195 |
| *Leptospira* | Leptospira_biflexa_serovar_Patoc__Patoc_1__Paris__uid58993 | 0.194 |
| *Renibacterium* | Renibacterium_salmoninarum_ATCC_33209_uid58899 | 0.193 |
| *Actinosynnema* | Actinosynnema_mirum_DSM_43827_uid58951 | 0.192 |
| *Leptospira* | Leptospira_biflexa_serovar_Patoc__Patoc_1__Ames__uid58511 | 0.192 |
| *Marinomonas* | Marinomonas_posidonica_IVIA_Po_181_uid67323 | 0.191 |
| *Spirochaeta* | Spirochaeta_Buddy_uid63633 | 0.191 |
| *Pseudonocardia* | Pseudonocardia_dioxanivorans_CB1190_uid65087 | 0.19 |
| *Micromonospora* | Micromonospora_L5_uid45895 | 0.188 |
| *Teredinibacter* | Teredinibacter_turnerae_T7901_uid59267 | 0.188 |
| *Candidatus_Protochlamydia* | Candidatus_Protochlamydia_amoebophila_UWE25_uid58079 | 0.188 |
| *Synechococcus* | Synechococcus_PCC_6312_uid182934 | 0.187 |
| *Desulfomonile* | Desulfomonile_tiedjei_DSM_6799_uid168320 | 0.184 |
| *Leptospira* | Leptospira_interrogans_serovar_Lai_IPAV_uid161957 | 0.184 |
| *Singulisphaera* | Singulisphaera_acidiphila_DSM_18658_uid81777 | 0.184 |
| *Anaplasma* | Anaplasma_phagocytophilum_HZ_uid57951 | 0.183 |
| *Anaplasma* | Anaplasma_phagocytophilum_Dog2_uid213225 | 0.183 |
| *Anaplasma* | Anaplasma_phagocytophilum_HZ2_uid213224 | 0.182 |
| *Octadecabacter* | Octadecabacter_antarcticus_307_uid54701 | 0.182 |
| *Leptospira* | Leptospira_interrogans_serovar_Copenhageni_Fiocruz_L1_130_uid58065 | 0.182 |
| *Anaplasma* | Anaplasma_phagocytophilum_JM_uid213223 | 0.181 |
| *Micromonospora* | Micromonospora_aurantiaca_ATCC_27029_uid42501 | 0.181 |
| *Marinomonas* | Marinomonas_MWYL1_uid58715 | 0.18 |
| *Anabaena* | Anabaena_90_uid179383 | 0.18 |
| *Leptospira* | Leptospira_interrogans_serovar_Lai_56601_uid57881 | 0.18 |
| *Saccharopolyspora* | Saccharopolyspora_erythraea_NRRL_2338_uid62947 | 0.175 |
| *Amycolatopsis* | Amycolatopsis_mediterranei_S699_uid158689 | 0.174 |
| *Amycolatopsis* | Amycolatopsis_mediterranei_U32_uid50565 | 0.174 |
| *Amycolatopsis* | Amycolatopsis_mediterranei_RB_uid216089 | 0.174 |
| *Amycolatopsis* | Amycolatopsis_mediterranei_S699_uid171830 | 0.174 |
| *Spirochaeta* | Spirochaeta_caldaria_DSM_7334_uid68753 | 0.174 |
| *Oscillatoria* | Oscillatoria_nigro_viridis_PCC_7112_uid183110 | 0.173 |
| *Hirschia* | Hirschia_baltica_ATCC_49814_uid59365 | 0.171 |
| *Amycolatopsis* | Amycolatopsis_orientalis_HCCB10007_uid203791 | 0.171 |
| *Octadecabacter* | Octadecabacter_arcticus_238_uid54699 | 0.17 |
| *Stanieria* | Stanieria_cyanosphaera_PCC_7437_uid183115 | 0.168 |
| *Hydrogenobacter* | Hydrogenobacter_thermophilus_TK_6_uid159875 | 0.167 |
| *Hydrogenobacter* | Hydrogenobacter_thermophilus_TK_6_uid45927 | 0.167 |
| *Cyanothece* | Cyanothece_PCC_8801_uid59027 | 0.167 |
| *Marinomonas* | Marinomonas_mediterranea_MMB_1_uid64753 | 0.165 |
| *Planctomyces* | Planctomyces_limnophilus_DSM_3776_uid48643 | 0.161 |
| *Alteromonas* | Alteromonas_SN2_uid67349 | 0.16 |
| *Amycolicicoccus* | Amycolicicoccus_subflavus_DQS3_9A1_uid67253 | 0.159 |
| *Cyanothece* | Cyanothece_PCC_7425_uid59435 | 0.157 |
| *Cyanothece* | Cyanothece_PCC_8802_uid59143 | 0.154 |
| *Methylacidiphilum* | Methylacidiphilum_infernorum_V4_uid59161 | 0.152 |
| *Nocardia* | Nocardia_brasiliensis_ATCC_700358_uid86913 | 0.152 |
| *Pseudanabaena* | Pseudanabaena_PCC_7367_uid183004 | 0.151 |
| *Saccharophagus* | Saccharophagus_degradans_2_40_uid57921 | 0.15 |
| *Cyanothece* | Cyanothece_PCC_7424_uid59025 | 0.15 |
| *Parachlamydia* | Parachlamydia_acanthamoebae_UV7_uid68335 | 0.147 |
| *Cyanothece* | Cyanothece_ATCC_51142_uid59013 | 0.143 |
| *Saccharothrix* | Saccharothrix_espanaensis_DSM_44229_uid184826 | 0.138 |
| *Pirellula* | Pirellula_staleyi_DSM_6068_uid43209 | 0.136 |
| *Chroococcidiopsis* | Chroococcidiopsis_thermalis_PCC_7203_uid183002 | 0.135 |
| *Colwellia* | Colwellia_psychrerythraea_34H_uid57855 | 0.133 |
| *Chlamydophila* | Chlamydophila_caviae_GPIC_uid57783 | 0.133 |
| *Cyanothece* | Cyanothece_PCC_7822_uid52547 | 0.132 |
| *Chlamydophila* | Chlamydophila_felis_Fe_C_56_uid57971 | 0.129 |
| *Herpetosiphon* | Herpetosiphon_aurantiacus_DSM_785_uid58599 | 0.129 |
| *Glaciecola* | Glaciecola_nitratireducens_FR1064_uid73759 | 0.128 |
| *Anabaena* | Anabaena_cylindrica_PCC_7122_uid183339 | 0.124 |
| *Crinalium* | Crinalium_epipsammum_PCC_9333_uid183113 | 0.124 |
| *Chamaesiphon* | Chamaesiphon_minutus_PCC_6605_uid183005 | 0.122 |
| *Pleurocapsa* | Pleurocapsa_PCC_7327_uid183006 | 0.122 |
| *Thermocrinis* | Thermocrinis_albus_DSM_14484_uid46231 | 0.12 |
| *Nostoc* | Nostoc_PCC_7120_uid57803 | 0.119 |
| *Glaciecola* | Glaciecola_psychrophila_170_uid193711 | 0.118 |
| *Simkania* | Simkania_negevensis_Z_uid68451 | 0.118 |
| *Pseudoalteromonas* | Pseudoalteromonas_atlantica_T6c_uid58283 | 0.117 |
| *Dactylococcopsis* | Dactylococcopsis_salina_PCC_8305_uid183341 | 0.117 |
| *Nostoc* | Nostoc_PCC_7524_uid182933 | 0.115 |
| *Oscillatoria* | Oscillatoria_acuminata_PCC_6304_uid183003 | 0.114 |
| *Rivularia* | Rivularia_PCC_7116_uid182929 | 0.113 |
| *Halothece* | Halothece_PCC_7418_uid183338 | 0.112 |
| *Arthrospira* | Arthrospira_platensis_NIES_39_uid197171 | 0.111 |
| *Leptolyngbya* | Leptolyngbya_PCC_7376_uid182928 | 0.11 |
| *Microcoleus* | Microcoleus_PCC_7113_uid183114 | 0.11 |
| *Microcystis* | Microcystis_aeruginosa_NIES_843_uid59101 | 0.108 |
| *Glaciecola* | Glaciecola_4H_3_7_YE_5_uid66595 | 0.104 |
| *Chlamydophila* | Chlamydophila_abortus_S26_3_uid57963 | 0.102 |
| *Rhodopirellula* | Rhodopirellula_baltica_SH_1_uid61589 | 0.097 |
| *Gloeocapsa* | Gloeocapsa_PCC_7428_uid183112 | 0.095 |
| *Trichodesmium* | Trichodesmium_erythraeum_IMS101_uid57925 | 0.095 |
| *Nostoc* | Nostoc_PCC_7107_uid182932 | 0.094 |
| *Nostoc* | Nostoc_punctiforme_PCC_73102_uid57767 | 0.093 |
| *Acaryochloris* | Acaryochloris_marina_MBIC11017_uid58167 | 0.093 |
| *Calothrix* | Calothrix_PCC_7507_uid182930 | 0.093 |
| *Calothrix* | Calothrix_PCC_6303_uid183109 | 0.092 |
| *Anabaena* | Anabaena_variabilis_ATCC_29413_uid58043 | 0.081 |
| *Acidobacterium* | Acidobacterium_MP5ACTX9_uid50551 | 0.08 |
| *Chlamydophila* | Chlamydophila_pecorum_E58_uid66295 | 0.079 |
| *Gluconacetobacter* | Gluconacetobacter_xylinus_NBRC_3288_uid46523 | 0.078 |
| *Nostoc* | _Nostoc_azollae__0708_uid49725 | 0.07 |
| *Cylindrospermum* | Cylindrospermum_stagnale_PCC_7417_uid183111 | 0.061 |
| *Chlamydophila* | Chlamydophila_psittaci_Mat116_uid189026 | 0.053 |
| *Chlamydophila* | Chlamydophila_psittaci_C19_98_uid159523 | 0.051 |
| *Chlamydophila* | Chlamydophila_psittaci_6BC_uid159845 | 0.051 |
| *Chlamydophila* | Chlamydophila_psittaci_6BC_uid63621 | 0.051 |
| *Chlamydophila* | Chlamydophila_psittaci_08DC60_uid159525 | 0.051 |
| *Chlamydophila* | Chlamydophila_psittaci_02DC15_uid159521 | 0.051 |
| *Chlamydophila* | Chlamydophila_psittaci_01DC11_uid159527 | 0.051 |
| *Chlamydophila* | Chlamydophila_psittaci_RD1_uid162063 | 0.04 |
| *Acetobacter* | Acetobacterium_woodii_DSM_1030_uid88073 | 0.031 |
| *Chlamydophila* | Chlamydophila_pneumoniae_TW_183_uid57997 | 0.021 |
| *Chlamydophila* | Chlamydophila_pneumoniae_J138_uid57829 | 0.021 |
| *Chlamydophila* | Chlamydophila_pneumoniae_CWL029_uid57811 | 0.021 |
| *Chlamydophila* | Chlamydophila_pneumoniae_AR39_uid57809 | 0.02 |
| *Chlamydophila* | Chlamydophila_pneumoniae_LPCoLN_uid159529 | 0.02 |
| *Cellvibrio* | Cellvibrio__gilvus_ATCC_13127_uid68143 | 0.013 |
