# Supplementary material for: Reconstruction of Bacterial and Viral Genomes from Multiple Metagenomes
Source: Front Microbiol. 2016 Apr 12;7:469. doi: 10.3389/fmicb.2016.00469 (PMC4828583; doi:10.3389/fmicb.2016.00469)
Supplement: Supplementary file 6 [file Table6.DOCX]

**Table S6. Genome validation of the draft assemblies on the basis of the completeness of the ORFs detected.** * represents sequences without N's (unknown nucleotides). ** represents sequences which can contain only a single N at multiple places.

| **Genome** | **Total ORFs** | **Total ORFs in draft* (without N's)** | **Complete ORFs* (without N's)** | **% Complete ORFs** | **Total ORFs in draft** (with single N's)** | **% ORFs Found** | **Complete ORFs** (with single N's)** | **% Complete ORFs** |
| --- | --- | --- | --- | --- | --- | --- | --- | --- |
| Akkermansia_muciniphila_ATCC_BAA_835 | 2138 | 994 | 993 | 99.90 | 2056 | 96.12 | 2055 | 99.95 |
| Bacteroides_thetaiotaomicron_VPI_5482 | 4778 | 2827 | 2821 | 99.79 | 4588 | 95.84 | 4579 | 99.80 |
| Bifidobacterium_longum_JCM_1217 | 1924 | 386 | 384 | 99.48 | 1284 | 66.63 | 1282 | 99.84 |
| Escherichia_coli_K_12_substr__MDS42 | 3543 | 1412 | 1410 | 99.86 | 2535 | 71.44 | 2531 | 99.84 |
| Eubacterium_siraeum_V10Sc8a | 2202 | 1434 | 1426 | 99.44 | 2053 | 92.82 | 2044 | 99.56 |
| Odoribacter_splanchnicus_DSM_20712 | 3497 | 2484 | 2483 | 99.96 | 3356 | 95.91 | 3354 | 99.94 |
| Parabacteroides_distasonis_ATCC_8503 | 3849 | 3018 | 3011 | 99.77 | 3675 | 95.30 | 3668 | 99.81 |
| Roseburia_hominis_A2_183 | 3362 | 1508 | 1506 | 99.87 | 3031 | 90.04 | 3027 | 99.87 |
