# Supplementary material for: Reconstruction of Bacterial and Viral Genomes from Multiple Metagenomes
Source: Front Microbiol. 2016 Apr 12;7:469. doi: 10.3389/fmicb.2016.00469 (PMC4828583; doi:10.3389/fmicb.2016.00469)
Supplement: Supplementary file 7 [file Table7.DOCX]

**Table S7. Percentage of assembly achieved, number of reads, cumulative reads and number of reads mapped for the incremental addition of 72 metagenomes based on the respective genus abundance for eight selected bacterial genomes. '** # Reads' represents the number of reads present of the genus in that particular metagenome. '# Reads Used' represents the number of reads which were able to map on the reference genome using BWA.

| **Genus** | **Metagenome** | **Assembly (%)** | **# Reads** | **# Cumulative Reads** | **# Reads Used** |
| --- | --- | --- | --- | --- | --- |
| **Akkermansia** | MH0060_081222 | 91.02 | 164835 | 164835 | 164296 |
|  | MH0054_081222 | 95.79 | 162501 | 327336 | 326976 |
|  | MH0002_081224 | 96.91 | 129991 | 457327 | 457110 |
|  | MH0035_081223 | 97.01 | 92750 | 550077 | 549859 |
|  | MH0066_081223 | 97.05 | 70912 | 620989 | 619485 |
|  | MH0037_081226 | 97.22 | 61727 | 682716 | 681790 |
|  | MH0012_081224 | 97.43 | 56938 | 739654 | 738594 |
|  | MH0025_090120 | 98.03 | 50278 | 789932 | 788665 |
|  | MH0071_090104 | 98.05 | 41517 | 831449 | 829721 |
|  | MH0011_081224 | 98.20 | 38689 | 870138 | 868177 |
|  | MH0048_081226 | 98.40 | 34214 | 904352 | 902450 |
|  | MH0012_081203 | 98.41 | 30822 | 935174 | 933167 |
|  | MH0050_081223 | 98.42 | 29613 | 964787 | 962490 |
|  | MH0043_081214 | 98.42 | 28715 | 993502 | 991057 |
|  | MH0059_081230 | 98.43 | 27655 | 1021157 | 1018754 |
|  | MH0011_081203 | 98.45 | 25900 | 1047057 | 1044439 |
|  | MH0058_081230 | 98.45 | 21496 | 1068553 | 1065792 |
|  | MH0064_081223 | 98.45 | 21091 | 1089644 | 1086567 |
|  | MH0009_081224 | 98.45 | 19961 | 1109605 | 1106417 |
|  | MH0021_081224 | 98.46 | 18694 | 1128299 | 1124992 |
|  | MH0028_081223 | 98.50 | 16212 | 1144511 | 1140942 |
|  | MH0075_090104 | 98.51 | 15952 | 1160463 | 1156483 |
|  | MH0042_081226 | 98.51 | 11118 | 1171581 | 1167551 |
|  | MH0068_081223 | 98.52 | 10625 | 1182206 | 1178002 |
|  | MH0040_081223 | 98.52 | 9631 | 1191837 | 1187177 |
|  | MH0031_081223 | 98.52 | 8117 | 1199954 | 1194874 |
|  | MH0083_090105 | 98.53 | 6424 | 1206378 | 1200759 |
|  | MH0080_090109 | 98.54 | 6175 | 1212553 | 1206678 |
|  | MH0086_081223 | 98.54 | 4287 | 1216840 | 1210652 |
|  | MH0045_081223 | 98.54 | 4264 | 1221104 | 1214643 |
|  | MH0070_090104 | 98.54 | 4201 | 1225305 | 1218479 |
|  | MH0067_090103 | 98.54 | 3858 | 1229163 | 1222086 |
|  | MH0039_081126 | 98.58 | 3699 | 1232862 | 1225622 |
|  | MH0082_090105 | 98.60 | 3631 | 1236493 | 1228883 |
|  | MH0014_081224 | 98.60 | 3605 | 1240098 | 1232219 |
|  | MH0081_090105 | 98.60 | 2906 | 1243004 | 1234620 |
|  | MH0041_081222 | 98.60 | 2720 | 1245724 | 1237055 |
|  | MH0014_081203 | 98.60 | 2324 | 1248048 | 1239191 |
|  | MH0074_081223 | 98.60 | 2013 | 1250061 | 1240803 |
|  | MH0051_081226 | 98.60 | 1866 | 1251927 | 1242309 |
|  | MH0057_081222 | 98.60 | 1774 | 1253701 | 1243650 |
|  | MH0076_081223 | 98.62 | 1755 | 1255456 | 1245081 |
|  | MH0065_081223 | 98.62 | 1650 | 1257106 | 1246339 |
|  | MH0032_081224 | 98.70 | 1565 | 1258671 | 1247622 |
|  | MH0024_081224 | 98.70 | 1343 | 1260014 | 1248484 |
|  | MH0016_081224 | 98.70 | 1252 | 1261266 | 1249524 |
|  | MH0084_090109 | 98.70 | 1217 | 1262483 | 1250592 |
|  | MH0047_081223 | 98.70 | 1127 | 1263610 | 1251062 |
|  | MH0073_081223 | 98.70 | 1071 | 1264681 | 1251928 |
|  | MH0046_081230 | 98.70 | 1009 | 1265690 | 1252794 |
|  | MH0079_090104 | 98.70 | 921 | 1266611 | 1253345 |
|  | MH0016_081203 | 98.70 | 892 | 1267503 | 1254082 |
|  | MH0061_090103 | 98.70 | 738 | 1268241 | 1254522 |
|  | MH0036_081226 | 98.70 | 673 | 1268914 | 1254849 |
|  | MH0063_090103 | 98.70 | 660 | 1269574 | 1255192 |
|  | MH0062_081223 | 98.71 | 638 | 1270212 | 1255482 |
|  | MH0077_081223 | 98.71 | 606 | 1270818 | 1255744 |
|  | MH0055_081223 | 98.71 | 597 | 1271415 | 1256002 |
|  | MH0085_081223 | 98.71 | 589 | 1272004 | 1256265 |
|  | MH0052_090103 | 98.71 | 580 | 1272584 | 1256497 |
|  | MH0053_081222 | 98.71 | 543 | 1273127 | 1256767 |
|  | MH0044_081222 | 98.71 | 519 | 1273646 | 1257063 |
|  | MH0069_090103 | 98.71 | 509 | 1274155 | 1257241 |
|  | MH0049_081223 | 98.71 | 504 | 1274659 | 1257505 |
|  | MH0003_081224 | 98.71 | 469 | 1275128 | 1257669 |
|  | MH0033_081224 | 98.71 | 462 | 1275590 | 1257983 |
|  | MH0078_081222 | 98.71 | 441 | 1276031 | 1258267 |
|  | MH0030_081224 | 98.71 | 343 | 1276374 | 1258371 |
|  | MH0020_081224 | 98.71 | 339 | 1276713 | 1258590 |
|  | MH0038_081226 | 98.71 | 315 | 1277028 | 1258731 |
|  | MH0072_081223 | 98.71 | 305 | 1277333 | 1258872 |
|  | MH0003_081203 | 98.71 | 192 | 1277525 | 1258928 |
|  |  |  |  |  |  |
| **Bacteroides** | MH0068_081223 | 76.64 | 3001408 | 3001408 | 709587 |
|  | MH0061_090103 | 78.64 | 2471548 | 5472956 | 1060740 |
|  | MH0072_081223 | 91.13 | 1930568 | 7403524 | 1466991 |
|  | MH0020_081224 | 91.57 | 1767185 | 9170709 | 1730366 |
|  | MH0016_081224 | 92.87 | 1735128 | 10905837 | 2002920 |
|  | MH0045_081223 | 93.00 | 1609690 | 12515527 | 2346104 |
|  | MH0067_090103 | 93.00 | 1388074 | 13903601 | 2551586 |
|  | MH0085_081223 | 93.03 | 1336091 | 15239692 | 2879241 |
|  | MH0080_090109 | 93.03 | 1257003 | 16496695 | 3136696 |
|  | MH0043_081214 | 93.05 | 1198574 | 17695269 | 3320068 |
|  | MH0041_081222 | 93.05 | 1184234 | 18879503 | 3685533 |
|  | MH0036_081226 | 93.06 | 1098418 | 19977921 | 3893915 |
|  | MH0044_081222 | 93.06 | 1093000 | 21070921 | 4038516 |
|  | MH0076_081223 | 93.06 | 1085662 | 22156583 | 4212060 |
|  | MH0073_081223 | 94.47 | 1079615 | 23236198 | 4455959 |
|  | MH0058_081230 | 94.67 | 1066613 | 24302811 | 4682921 |
|  | MH0049_081223 | 95.25 | 1058018 | 25360829 | 4859222 |
|  | MH0039_081126 | 96.20 | 1043973 | 26404802 | 5059848 |
|  | MH0025_090120 | 96.70 | 998260 | 27403062 | 5229055 |
|  | MH0003_081224 | 97.03 | 948202 | 28351264 | 5435283 |
|  | MH0016_081203 | 97.14 | 936284 | 29287548 | 5583834 |
|  | MH0083_090105 | 97.18 | 934674 | 30222222 | 5856511 |
|  | MH0081_090105 | 97.31 | 913498 | 31135720 | 5995985 |
|  | MH0084_090109 | 97.34 | 895544 | 32031264 | 6229227 |
|  | MH0082_090105 | 97.67 | 809212 | 32840476 | 6321905 |
|  | MH0035_081223 | 97.67 | 800144 | 33640620 | 6459041 |
|  | MH0021_081224 | 97.73 | 695711 | 34336331 | 6572557 |
|  | MH0028_081223 | 97.75 | 685750 | 35022081 | 6736080 |
|  | MH0070_090104 | 97.96 | 679046 | 35701127 | 6872631 |
|  | MH0040_081223 | 97.96 | 672219 | 36373346 | 7015926 |
|  | MH0047_081223 | 98.03 | 667295 | 37040641 | 7080988 |
|  | MH0002_081224 | 98.07 | 640624 | 37681265 | 7200314 |
|  | MH0014_081224 | 98.09 | 609561 | 38290826 | 7373233 |
|  | MH0033_081224 | 98.16 | 601446 | 38892272 | 7465141 |
|  | MH0059_081230 | 98.18 | 592410 | 39484682 | 7559997 |
|  | MH0071_090104 | 98.19 | 577292 | 40061974 | 7670826 |
|  | MH0077_081223 | 98.19 | 575035 | 40637009 | 7756379 |
|  | MH0057_081222 | 98.22 | 572387 | 41209396 | 7853302 |
|  | MH0064_081223 | 98.23 | 568742 | 41778138 | 7978331 |
|  | MH0051_081226 | 98.23 | 562350 | 42340488 | 8135475 |
|  | MH0075_090104 | 98.23 | 552039 | 42892527 | 8225801 |
|  | MH0037_081226 | 98.26 | 551388 | 43443915 | 8331901 |
|  | MH0086_081223 | 98.27 | 522661 | 43966576 | 8438956 |
|  | MH0054_081222 | 98.27 | 521111 | 44487687 | 8555619 |
|  | MH0055_081223 | 98.28 | 510858 | 44998545 | 8664554 |
|  | MH0074_081223 | 98.29 | 479280 | 45477825 | 8813490 |
|  | MH0062_081223 | 98.30 | 469070 | 45946895 | 8920518 |
|  | MH0011_081224 | 98.39 | 464347 | 46411242 | 9023176 |
|  | MH0069_090103 | 98.40 | 453111 | 46864353 | 9109234 |
|  | MH0048_081226 | 98.40 | 440553 | 47304906 | 9189870 |
|  | MH0038_081226 | 98.41 | 419284 | 47724190 | 9296069 |
|  | MH0066_081223 | 98.41 | 398749 | 48122939 | 9453475 |
|  | MH0024_081224 | 98.41 | 383011 | 48505950 | 9566083 |
|  | MH0050_081223 | 98.42 | 380304 | 48886254 | 9664852 |
|  | MH0042_081226 | 98.42 | 352957 | 49239211 | 9717619 |
|  | MH0014_081203 | 98.42 | 329994 | 49569205 | 9810573 |
|  | MH0032_081224 | 98.61 | 324778 | 49893983 | 9866485 |
|  | MH0079_090104 | 98.61 | 323283 | 50217266 | 9952614 |
|  | MH0052_090103 | 98.62 | 303624 | 50520890 | 10011638 |
|  | MH0065_081223 | 98.76 | 301423 | 50822313 | 10064130 |
|  | MH0012_081224 | 98.77 | 289827 | 51112140 | 10132642 |
|  | MH0003_081203 | 98.77 | 271612 | 51383752 | 10191681 |
|  | MH0063_090103 | 98.77 | 250230 | 51633982 | 10264167 |
|  | MH0011_081203 | 98.77 | 247641 | 51881623 | 10319675 |
|  | MH0060_081222 | 98.78 | 226383 | 52108006 | 10386517 |
|  | MH0046_081230 | 98.78 | 216071 | 52324077 | 10442924 |
|  | MH0009_081224 | 98.78 | 162758 | 52486835 | 10475363 |
|  | MH0078_081222 | 98.83 | 146310 | 52633145 | 10540727 |
|  | MH0012_081203 | 98.87 | 145063 | 52778208 | 10575126 |
|  | MH0031_081223 | 98.87 | 134081 | 52912289 | 10615603 |
|  | MH0053_081222 | 98.87 | 106218 | 53018507 | 10670656 |
|  | MH0030_081224 | 98.89 | 71481 | 53089988 | 10692762 |
|  |  | | | | |
| **Bifidobacterium** | MH0041_081222 | 43.22 | 90369 | 90369 | 23630 |
|  | MH0086_081223 | 83.99 | 83436 | 173805 | 70454 |
|  | MH0066_081223 | 86.85 | 65474 | 239279 | 90700 |
|  | MH0035_081223 | 87.62 | 49702 | 288981 | 103207 |
|  | MH0069_090103 | 87.94 | 46704 | 335685 | 113648 |
|  | MH0042_081226 | 88.85 | 43112 | 378797 | 126146 |
|  | MH0071_090104 | 90.72 | 41805 | 420602 | 146786 |
|  | MH0012_081224 | 91.41 | 34953 | 455555 | 161898 |
|  | MH0025_090120 | 91.48 | 33824 | 489379 | 168836 |
|  | MH0021_081224 | 91.71 | 30800 | 520179 | 189710 |
|  | MH0020_081224 | 91.82 | 29437 | 549616 | 197355 |
|  | MH0083_090105 | 92.04 | 28902 | 578518 | 208565 |
|  | MH0062_081223 | 94.72 | 28198 | 606716 | 224094 |
|  | MH0077_081223 | 94.77 | 27689 | 634405 | 233381 |
|  | MH0048_081226 | 94.83 | 27358 | 661763 | 239648 |
|  | MH0028_081223 | 94.83 | 27189 | 688952 | 244806 |
|  | MH0059_081230 | 95.73 | 23731 | 712683 | 257526 |
|  | MH0054_081222 | 95.76 | 23117 | 735800 | 261667 |
|  | MH0068_081223 | 95.90 | 22164 | 757964 | 277667 |
|  | MH0075_090104 | 95.95 | 20961 | 778925 | 286356 |
|  | MH0012_081203 | 96.01 | 20121 | 799046 | 295167 |
|  | MH0067_090103 | 96.10 | 19996 | 819042 | 309954 |
|  | MH0053_081222 | 96.12 | 19282 | 838324 | 313758 |
|  | MH0002_081224 | 96.14 | 18242 | 856566 | 317905 |
|  | MH0047_081223 | 96.15 | 18109 | 874675 | 322225 |
|  | MH0082_090105 | 96.17 | 17973 | 892648 | 327319 |
|  | MH0043_081214 | 96.19 | 17931 | 910579 | 332620 |
|  | MH0064_081223 | 96.20 | 16919 | 927498 | 337039 |
|  | MH0050_081223 | 96.52 | 16902 | 944400 | 342789 |
|  | MH0072_081223 | 96.55 | 16185 | 960585 | 350464 |
|  | MH0076_081223 | 96.58 | 15087 | 975672 | 355209 |
|  | MH0074_081223 | 96.66 | 14916 | 990588 | 364962 |
|  | MH0073_081223 | 96.69 | 12875 | 1003463 | 371374 |
|  | MH0031_081223 | 96.72 | 12865 | 1016328 | 377205 |
|  | MH0085_081223 | 96.87 | 12756 | 1029084 | 383246 |
|  | MH0003_081224 | 96.90 | 12424 | 1041508 | 390121 |
|  | MH0070_090104 | 96.91 | 12223 | 1053731 | 394276 |
|  | MH0081_090105 | 96.92 | 12076 | 1065807 | 397801 |
|  | MH0044_081222 | 96.94 | 12055 | 1077862 | 401912 |
|  | MH0060_081222 | 96.96 | 11639 | 1089501 | 407375 |
|  | MH0055_081223 | 96.96 | 11636 | 1101137 | 409542 |
|  | MH0052_090103 | 96.97 | 11565 | 1112702 | 413181 |
|  | MH0040_081223 | 97.01 | 10853 | 1123555 | 419208 |
|  | MH0049_081223 | 97.01 | 10786 | 1134341 | 421030 |
|  | MH0011_081224 | 97.02 | 9964 | 1144305 | 423177 |
|  | MH0065_081223 | 97.03 | 9887 | 1154192 | 426508 |
|  | MH0033_081224 | 97.04 | 9230 | 1163422 | 429663 |
|  | MH0030_081224 | 97.04 | 9124 | 1172546 | 431210 |
|  | MH0063_090103 | 97.04 | 8663 | 1181209 | 432305 |
|  | MH0039_081126 | 97.05 | 7919 | 1189128 | 435368 |
|  | MH0014_081224 | 97.05 | 7606 | 1196734 | 436865 |
|  | MH0011_081203 | 97.06 | 7568 | 1204302 | 438678 |
|  | MH0058_081230 | 97.07 | 6960 | 1211262 | 440999 |
|  | MH0079_090104 | 97.07 | 6838 | 1218100 | 441620 |
|  | MH0009_081224 | 97.08 | 6348 | 1224448 | 443294 |
|  | MH0051_081226 | 97.09 | 6251 | 1230699 | 445854 |
|  | MH0038_081226 | 97.09 | 5914 | 1236613 | 448194 |
|  | MH0032_081224 | 97.10 | 5680 | 1242293 | 449720 |
|  | MH0014_081203 | 97.10 | 5439 | 1247732 | 450845 |
|  | MH0078_081222 | 97.10 | 5198 | 1252930 | 451879 |
|  | MH0045_081223 | 97.10 | 4982 | 1257912 | 452584 |
|  | MH0003_081203 | 97.12 | 4957 | 1262869 | 455398 |
|  | MH0084_090109 | 97.12 | 4509 | 1267378 | 455802 |
|  | MH0057_081222 | 97.13 | 4337 | 1271715 | 457722 |
|  | MH0024_081224 | 97.13 | 4261 | 1275976 | 458034 |
|  | MH0080_090109 | 97.13 | 4235 | 1280211 | 458522 |
|  | MH0061_090103 | 97.13 | 3464 | 1283675 | 458790 |
|  | MH0037_081226 | 97.13 | 3352 | 1287027 | 459070 |
|  | MH0036_081226 | 97.13 | 3093 | 1290120 | 459256 |
|  | MH0016_081224 | 97.13 | 2689 | 1292809 | 459532 |
|  | MH0016_081203 | 97.14 | 1642 | 1294451 | 459742 |
|  | MH0046_081230 | 97.14 | 1265 | 1295716 | 459827 |
|  |  | | | | |
| **Escherichia** | MH0014_081224 | 82.27 | 640389 | 640389 | 450228 |
|  | MH0014_081203 | 83.06 | 381788 | 1022177 | 722373 |
|  | MH0025_090120 | 91.49 | 228133 | 1250310 | 862001 |
|  | MH0079_090104 | 93.20 | 71318 | 1321628 | 906580 |
|  | MH0071_090104 | 93.52 | 28740 | 1350368 | 925977 |
|  | MH0054_081222 | 93.76 | 26335 | 1376703 | 944778 |
|  | MH0086_081223 | 93.99 | 24134 | 1400837 | 961093 |
|  | MH0061_090103 | 94.23 | 13505 | 1414342 | 969294 |
|  | MH0002_081224 | 94.42 | 13238 | 1427580 | 975520 |
|  | MH0068_081223 | 94.56 | 12475 | 1440055 | 983378 |
|  | MH0012_081224 | 94.70 | 12125 | 1452180 | 990693 |
|  | MH0075_090104 | 94.75 | 10782 | 1462962 | 997766 |
|  | MH0059_081230 | 94.80 | 9945 | 1472907 | 1004196 |
|  | MH0012_081203 | 94.86 | 6249 | 1479156 | 1007998 |
|  | MH0058_081230 | 94.93 | 6186 | 1485342 | 1012063 |
|  | MH0082_090105 | 95.02 | 6028 | 1491370 | 1015657 |
|  | MH0003_081224 | 95.05 | 5349 | 1496719 | 1019138 |
|  | MH0037_081226 | 95.08 | 4824 | 1501543 | 1022106 |
|  | MH0052_090103 | 95.14 | 4443 | 1505986 | 1024158 |
|  | MH0062_081223 | 95.17 | 4416 | 1510402 | 1026687 |
|  | MH0009_081224 | 95.29 | 4016 | 1514418 | 1028978 |
|  | MH0074_081223 | 95.41 | 3750 | 1518168 | 1030863 |
|  | MH0047_081223 | 95.42 | 2887 | 1521055 | 1032438 |
|  | MH0070_090104 | 95.46 | 2853 | 1523908 | 1033858 |
|  | MH0041_081222 | 95.48 | 2625 | 1526533 | 1035159 |
|  | MH0073_081223 | 95.51 | 2597 | 1529130 | 1036483 |
|  | MH0083_090105 | 95.53 | 2179 | 1531309 | 1036925 |
|  | MH0085_081223 | 95.54 | 2034 | 1533343 | 1037807 |
|  | MH0081_090105 | 95.54 | 1693 | 1535036 | 1038408 |
|  | MH0003_081203 | 95.55 | 1681 | 1536717 | 1039536 |
|  | MH0072_081223 | 95.57 | 1673 | 1538390 | 1040301 |
|  | MH0045_081223 | 95.58 | 1601 | 1539991 | 1041002 |
|  | MH0065_081223 | 95.59 | 1502 | 1541493 | 1041447 |
|  | MH0036_081226 | 95.62 | 1232 | 1542725 | 1041791 |
|  | MH0060_081222 | 95.63 | 1198 | 1543923 | 1042213 |
|  | MH0050_081223 | 95.64 | 1161 | 1545084 | 1042553 |
|  | MH0053_081222 | 95.65 | 1075 | 1546159 | 1042809 |
|  | MH0011_081224 | 95.65 | 1062 | 1547221 | 1043051 |
|  | MH0076_081223 | 95.65 | 1048 | 1548269 | 1043088 |
|  | MH0066_081223 | 95.66 | 1030 | 1549299 | 1043394 |
|  | MH0084_090109 | 95.66 | 977 | 1550276 | 1043432 |
|  | MH0042_081226 | 95.66 | 929 | 1551205 | 1043556 |
|  | MH0043_081214 | 95.67 | 903 | 1552108 | 1043704 |
|  | MH0035_081223 | 95.67 | 879 | 1552987 | 1043765 |
|  | MH0064_081223 | 95.67 | 879 | 1553866 | 1043788 |
|  | MH0044_081222 | 95.68 | 864 | 1554730 | 1043999 |
|  | MH0032_081224 | 95.68 | 855 | 1555585 | 1044081 |
|  | MH0078_081222 | 95.70 | 847 | 1556432 | 1044450 |
|  | MH0080_090109 | 95.70 | 844 | 1557276 | 1044603 |
|  | MH0063_090103 | 95.70 | 830 | 1558106 | 1044647 |
|  | MH0033_081224 | 95.70 | 789 | 1558895 | 1044832 |
|  | MH0077_081223 | 95.70 | 774 | 1559669 | 1044886 |
|  | MH0067_090103 | 95.70 | 748 | 1560417 | 1044915 |
|  | MH0020_081224 | 95.70 | 744 | 1561161 | 1045023 |
|  | MH0021_081224 | 95.70 | 737 | 1561898 | 1045048 |
|  | MH0028_081223 | 95.71 | 733 | 1562631 | 1045082 |
|  | MH0031_081223 | 95.71 | 732 | 1563363 | 1045129 |
|  | MH0048_081226 | 95.71 | 680 | 1564043 | 1045152 |
|  | MH0024_081224 | 95.71 | 662 | 1564705 | 1045177 |
|  | MH0049_081223 | 95.71 | 662 | 1565367 | 1045203 |
|  | MH0051_081226 | 95.71 | 649 | 1566016 | 1045361 |
|  | MH0030_081224 | 95.71 | 645 | 1566661 | 1045392 |
|  | MH0055_081223 | 95.71 | 631 | 1567292 | 1045418 |
|  | MH0038_081226 | 95.71 | 622 | 1567914 | 1045481 |
|  | MH0016_081224 | 95.71 | 581 | 1568495 | 1045511 |
|  | MH0011_081203 | 95.71 | 577 | 1569072 | 1045675 |
|  | MH0040_081223 | 95.72 | 576 | 1569648 | 1045713 |
|  | MH0069_090103 | 95.72 | 569 | 1570217 | 1045751 |
|  | MH0057_081222 | 95.72 | 424 | 1570641 | 1045797 |
|  | MH0039_081126 | 95.72 | 402 | 1571043 | 1045806 |
|  | MH0016_081203 | 95.72 | 333 | 1571376 | 1045827 |
|  | MH0046_081230 | 95.72 | 239 | 1571615 | 1045845 |
|  |  | | | | |
| **Eubacterium** | MH0002_081224 | 83.20 | 1351700 | 1351700 | 240673 |
|  | MH0082_090105 | 89.42 | 771716 | 2123416 | 593639 |
|  | MH0059_081230 | 90.28 | 689605 | 2813021 | 722884 |
|  | MH0085_081223 | 90.35 | 652867 | 3465888 | 731072 |
|  | MH0051_081226 | 91.12 | 606004 | 4071892 | 1149723 |
|  | MH0080_090109 | 91.95 | 593693 | 4665585 | 1289632 |
|  | MH0033_081224 | 95.54 | 527945 | 5193530 | 1605645 |
|  | MH0069_090103 | 95.56 | 464193 | 5657723 | 1633099 |
|  | MH0072_081223 | 95.56 | 463900 | 6121623 | 1643436 |
|  | MH0035_081223 | 95.60 | 445080 | 6566703 | 1658885 |
|  | MH0045_081223 | 92.27 | 414779 | 6981482 | 1672334 |
|  | MH0038_081226 | 92.77 | 409777 | 7391259 | 1948629 |
|  | MH0036_081226 | 92.88 | 393425 | 7784684 | 1980081 |
|  | MH0021_081224 | 93.23 | 365742 | 8150426 | 1994177 |
|  | MH0071_090104 | 93.42 | 352358 | 8502784 | 2078028 |
|  | MH0041_081222 | 93.42 | 344978 | 8847762 | 2087901 |
|  | MH0009_081224 | 93.48 | 334724 | 9182486 | 2366426 |
|  | MH0066_081223 | 93.51 | 326822 | 9509308 | 2409371 |
|  | MH0076_081223 | 93.76 | 319521 | 9828829 | 2520487 |
|  | MH0070_090104 | 93.83 | 306440 | 10135269 | 2539885 |
|  | MH0077_081223 | 93.83 | 299451 | 10434720 | 2559966 |
|  | MH0054_081222 | 93.85 | 295014 | 10729734 | 2626198 |
|  | MH0031_081223 | 93.85 | 282757 | 11012491 | 2764703 |
|  | MH0044_081222 | 93.85 | 282632 | 11295123 | 2778215 |
|  | MH0068_081223 | 93.85 | 277150 | 11572273 | 2789144 |
|  | MH0028_081223 | 93.88 | 272448 | 11844721 | 2867903 |
|  | MH0052_090103 | 93.93 | 269781 | 12114502 | 2913412 |
|  | MH0053_081222 | 94.13 | 266248 | 12380750 | 3070189 |
|  | MH0042_081226 | 94.22 | 258080 | 12638830 | 3209546 |
|  | MH0025_090120 | 94.27 | 257130 | 12895960 | 3255787 |
|  | MH0043_081214 | 94.28 | 255428 | 13151388 | 3278845 |
|  | MH0040_081223 | 94.29 | 255103 | 13406491 | 3381359 |
|  | MH0073_081223 | 94.29 | 254924 | 13661415 | 3388924 |
|  | MH0081_090105 | 94.29 | 234788 | 13896203 | 3452159 |
|  | MH0024_081224 | 94.32 | 233890 | 14130093 | 3553024 |
|  | MH0058_081230 | 94.32 | 223857 | 14353950 | 3573411 |
|  | MH0016_081224 | 94.36 | 218586 | 14572536 | 3655265 |
|  | MH0003_081224 | 94.37 | 217296 | 14789832 | 3716830 |
|  | MH0075_090104 | 94.38 | 208695 | 14998527 | 3753612 |
|  | MH0055_081223 | 94.39 | 202438 | 15200965 | 3808472 |
|  | MH0050_081223 | 94.40 | 193768 | 15394733 | 3851192 |
|  | MH0086_081223 | 94.41 | 187457 | 15582190 | 3876626 |
|  | MH0063_090103 | 94.45 | 171407 | 15753597 | 3890860 |
|  | MH0057_081222 | 94.45 | 166900 | 15920497 | 3911475 |
|  | MH0065_081223 | 94.45 | 160753 | 16081250 | 3923089 |
|  | MH0061_090103 | 94.45 | 156983 | 16238233 | 3931174 |
|  | MH0067_090103 | 94.45 | 147119 | 16385352 | 3941541 |
|  | MH0014_081224 | 94.45 | 141322 | 16526674 | 3952754 |
|  | MH0046_081230 | 94.45 | 140018 | 16666692 | 3957060 |
|  | MH0032_081224 | 94.45 | 139997 | 16806689 | 3967403 |
|  | MH0079_090104 | 94.47 | 138354 | 16945043 | 4000496 |
|  | MH0062_081223 | 94.48 | 133896 | 17078939 | 4022551 |
|  | MH0039_081126 | 94.48 | 132894 | 17211833 | 4035145 |
|  | MH0030_081224 | 94.49 | 130373 | 17342206 | 4042336 |
|  | MH0060_081222 | 94.49 | 122936 | 17465142 | 4058459 |
|  | MH0083_090105 | 94.50 | 120382 | 17585524 | 4071590 |
|  | MH0016_081203 | 94.50 | 118075 | 17703599 | 4118200 |
|  | MH0048_081226 | 94.50 | 108840 | 17812439 | 4163157 |
|  | MH0037_081226 | 94.50 | 104178 | 17916617 | 4173507 |
|  | MH0064_081223 | 94.51 | 102633 | 18019250 | 4182564 |
|  | MH0012_081224 | 94.51 | 93472 | 18112722 | 4194249 |
|  | MH0084_090109 | 94.51 | 90808 | 18203530 | 4200748 |
|  | MH0011_081224 | 94.51 | 80948 | 18284478 | 4212205 |
|  | MH0078_081222 | 95.06 | 80120 | 18364598 | 4228601 |
|  | MH0047_081223 | 95.06 | 74174 | 18438772 | 4263649 |
|  | MH0014_081203 | 95.06 | 73006 | 18511778 | 4270367 |
|  | MH0049_081223 | 95.07 | 68374 | 18580152 | 4274872 |
|  | MH0003_081203 | 95.07 | 62173 | 18642325 | 4294063 |
|  | MH0074_081223 | 95.07 | 54678 | 18697003 | 4298487 |
|  | MH0012_081203 | 95.07 | 47796 | 18744799 | 4304967 |
|  | MH0020_081224 | 95.07 | 47684 | 18792483 | 4309352 |
|  | MH0011_081203 | 95.08 | 42030 | 18834513 | 4316322 |
|  |  | | | | |
| **Odoribacter** | MH0080_090109 | 76.94 | 104861 | 104861 | 104058 |
|  | MH0074_081223 | 82.65 | 94861 | 199722 | 197774 |
|  | MH0067_090103 | 84.08 | 93721 | 293443 | 290157 |
|  | MH0050_081223 | 93.22 | 92573 | 386016 | 381435 |
|  | MH0043_081214 | 93.81 | 76308 | 462324 | 456772 |
|  | MH0081_090105 | 95.59 | 75049 | 537373 | 531102 |
|  | MH0068_081223 | 95.80 | 71775 | 609148 | 602583 |
|  | MH0036_081226 | 96.06 | 66758 | 675906 | 668528 |
|  | MH0016_081224 | 96.54 | 63745 | 739651 | 729604 |
|  | MH0003_081224 | 96.84 | 59589 | 799240 | 787707 |
|  | MH0045_081223 | 96.86 | 57442 | 856682 | 843568 |
|  | MH0021_081224 | 96.92 | 57273 | 913955 | 900373 |
|  | MH0075_090104 | 96.95 | 55591 | 969546 | 954650 |
|  | MH0066_081223 | 97.23 | 55124 | 1024670 | 1008797 |
|  | MH0012_081224 | 97.24 | 52443 | 1077113 | 1060033 |
|  | MH0002_081224 | 97.39 | 52162 | 1129275 | 1111442 |
|  | MH0079_090104 | 97.41 | 52024 | 1181299 | 1162211 |
|  | MH0041_081222 | 97.65 | 50098 | 1231397 | 1211219 |
|  | MH0028_081223 | 97.71 | 49993 | 1281390 | 1259511 |
|  | MH0011_081224 | 97.74 | 49785 | 1331175 | 1307804 |
|  | MH0051_081226 | 97.86 | 49342 | 1380517 | 1356261 |
|  | MH0085_081223 | 97.87 | 49316 | 1429833 | 1403880 |
|  | MH0077_081223 | 97.95 | 48303 | 1478136 | 1451148 |
|  | MH0020_081224 | 97.98 | 46947 | 1525083 | 1497233 |
|  | MH0058_081230 | 97.99 | 46097 | 1571180 | 1542355 |
|  | MH0072_081223 | 98.00 | 44403 | 1615583 | 1586094 |
|  | MH0040_081223 | 98.06 | 40280 | 1655863 | 1625877 |
|  | MH0083_090105 | 98.06 | 39316 | 1695179 | 1663725 |
|  | MH0039_081126 | 98.15 | 38953 | 1734132 | 1702045 |
|  | MH0035_081223 | 98.25 | 38373 | 1772505 | 1739736 |
|  | MH0073_081223 | 98.27 | 37826 | 1810331 | 1775975 |
|  | MH0086_081223 | 98.28 | 37168 | 1847499 | 1812474 |
|  | MH0076_081223 | 98.29 | 36522 | 1884021 | 1847741 |
|  | MH0016_081203 | 98.30 | 35602 | 1919623 | 1881746 |
|  | MH0084_090109 | 98.32 | 35471 | 1955094 | 1914244 |
|  | MH0060_081222 | 98.32 | 35267 | 1990361 | 1949086 |
|  | MH0069_090103 | 98.33 | 34514 | 2024875 | 1982571 |
|  | MH0049_081223 | 98.33 | 34385 | 2059260 | 2015184 |
|  | MH0064_081223 | 98.35 | 33799 | 2093059 | 2047648 |
|  | MH0063_090103 | 98.36 | 33280 | 2126339 | 2079210 |
|  | MH0037_081226 | 98.36 | 32845 | 2159184 | 2111624 |
|  | MH0065_081223 | 98.37 | 32240 | 2191424 | 2142833 |
|  | MH0047_081223 | 98.39 | 32129 | 2223553 | 2172847 |
|  | MH0062_081223 | 98.39 | 31743 | 2255296 | 2203812 |
|  | MH0014_081224 | 98.40 | 29152 | 2284448 | 2232164 |
|  | MH0055_081223 | 98.40 | 28529 | 2312977 | 2258522 |
|  | MH0044_081222 | 98.41 | 28455 | 2341432 | 2286217 |
|  | MH0059_081230 | 98.42 | 28199 | 2369631 | 2313778 |
|  | MH0012_081203 | 98.42 | 27331 | 2396962 | 2340489 |
|  | MH0070_090104 | 98.42 | 26673 | 2423635 | 2366037 |
|  | MH0052_090103 | 98.42 | 26272 | 2449907 | 2390778 |
|  | MH0011_081203 | 98.43 | 26102 | 2476009 | 2415975 |
|  | MH0024_081224 | 98.43 | 25951 | 2501960 | 2440258 |
|  | MH0082_090105 | 98.43 | 25628 | 2527588 | 2464899 |
|  | MH0042_081226 | 98.44 | 24793 | 2552381 | 2489132 |
|  | MH0071_090104 | 98.44 | 24670 | 2577051 | 2512409 |
|  | MH0009_081224 | 98.45 | 23996 | 2601047 | 2535588 |
|  | MH0033_081224 | 98.46 | 23305 | 2624352 | 2558331 |
|  | MH0048_081226 | 98.47 | 23138 | 2647490 | 2580789 |
|  | MH0038_081226 | 98.48 | 19890 | 2667380 | 2600199 |
|  | MH0031_081223 | 98.54 | 19869 | 2687249 | 2618868 |
|  | MH0078_081222 | 98.55 | 19075 | 2706324 | 2636602 |
|  | MH0057_081222 | 98.55 | 18890 | 2725214 | 2653894 |
|  | MH0054_081222 | 98.55 | 18222 | 2743436 | 2671410 |
|  | MH0025_090120 | 98.55 | 17680 | 2761116 | 2687684 |
|  | MH0003_081203 | 98.55 | 17308 | 2778424 | 2704522 |
|  | MH0030_081224 | 98.56 | 16206 | 2794630 | 2719638 |
|  | MH0014_081203 | 98.56 | 16161 | 2810791 | 2735292 |
|  | MH0032_081224 | 98.56 | 15764 | 2826555 | 2749944 |
|  | MH0046_081230 | 98.57 | 13991 | 2840546 | 2763096 |
|  | MH0061_090103 | 98.57 | 13539 | 2854085 | 2775044 |
|  | MH0053_081222 | 98.57 | 11982 | 2866067 | 2785796 |
|  |  | | | | |
| **Parabacteroides** | MH0074_081223 | 79.53 | 682853 | 682853 | 681875 |
|  | MH0068_081223 | 88.11 | 427379 | 1110232 | 1109505 |
|  | MH0020_081224 | 91.41 | 400726 | 1510958 | 1508606 |
|  | MH0080_090109 | 92.48 | 387490 | 1898448 | 1895131 |
|  | MH0024_081224 | 93.03 | 321273 | 2219721 | 2214184 |
|  | MH0067_090103 | 93.67 | 237149 | 2456870 | 2447248 |
|  | MH0083_090105 | 94.01 | 194353 | 2651223 | 2639867 |
|  | MH0021_081224 | 94.80 | 175362 | 2826585 | 2814986 |
|  | MH0025_090120 | 94.84 | 166177 | 2992762 | 2979043 |
|  | MH0028_081223 | 95.29 | 165566 | 3158328 | 3142272 |
|  | MH0085_081223 | 95.43 | 137994 | 3296322 | 3276932 |
|  | MH0038_081226 | 95.74 | 125243 | 3421565 | 3402472 |
|  | MH0076_081223 | 95.75 | 121960 | 3543525 | 3522764 |
|  | MH0058_081230 | 95.80 | 112633 | 3656158 | 3633517 |
|  | MH0039_081126 | 95.91 | 102415 | 3758573 | 3734904 |
|  | MH0016_081224 | 96.22 | 100294 | 3858867 | 3832201 |
|  | MH0059_081230 | 96.25 | 91985 | 3950852 | 3923199 |
|  | MH0057_081222 | 96.69 | 86830 | 4037682 | 4006212 |
|  | MH0035_081223 | 96.78 | 85014 | 4122696 | 4089969 |
|  | MH0062_081223 | 96.96 | 76702 | 4199398 | 4165647 |
|  | MH0048_081226 | 96.98 | 73650 | 4273048 | 4238608 |
|  | MH0047_081223 | 97.00 | 73214 | 4346262 | 4308328 |
|  | MH0014_081224 | 97.06 | 72923 | 4419185 | 4380159 |
|  | MH0079_090104 | 97.06 | 71975 | 4491160 | 4450639 |
|  | MH0072_081223 | 97.07 | 70691 | 4561851 | 4519940 |
|  | MH0084_090109 | 97.07 | 70409 | 4632260 | 4585375 |
|  | MH0055_081223 | 97.07 | 70180 | 4702440 | 4652667 |
|  | MH0003_081224 | 97.08 | 69921 | 4772361 | 4720347 |
|  | MH0075_090104 | 97.08 | 64385 | 4836746 | 4781896 |
|  | MH0082_090105 | 97.10 | 62754 | 4899500 | 4843341 |
|  | MH0051_081226 | 97.15 | 62331 | 4961831 | 4904419 |
|  | MH0045_081223 | 97.23 | 60610 | 5022441 | 4963125 |
|  | MH0011_081224 | 97.24 | 60297 | 5082738 | 5020822 |
|  | MH0077_081223 | 97.25 | 58722 | 5141460 | 5077645 |
|  | MH0012_081224 | 97.41 | 56341 | 5197801 | 5132378 |
|  | MH0016_081203 | 97.41 | 55030 | 5252831 | 5185718 |
|  | MH0041_081222 | 97.42 | 52530 | 5305361 | 5235741 |
|  | MH0071_090104 | 97.42 | 52257 | 5357618 | 5286221 |
|  | MH0049_081223 | 97.42 | 50590 | 5408208 | 5334129 |
|  | MH0064_081223 | 97.42 | 48642 | 5456850 | 5379072 |
|  | MH0081_090105 | 97.43 | 47810 | 5504660 | 5425504 |
|  | MH0065_081223 | 97.54 | 44541 | 5549201 | 5468186 |
|  | MH0070_090104 | 97.87 | 43166 | 5592367 | 5509675 |
|  | MH0014_081203 | 97.88 | 40971 | 5633338 | 5549941 |
|  | MH0032_081224 | 97.88 | 39711 | 5673049 | 5586945 |
|  | MH0073_081223 | 97.88 | 39070 | 5712119 | 5623102 |
|  | MH0002_081224 | 97.89 | 39018 | 5751137 | 5660703 |
|  | MH0063_090103 | 97.89 | 37354 | 5788491 | 5695383 |
|  | MH0009_081224 | 97.89 | 35728 | 5824219 | 5729775 |
|  | MH0033_081224 | 97.90 | 34973 | 5859192 | 5764084 |
|  | MH0044_081222 | 97.90 | 34445 | 5893637 | 5797006 |
|  | MH0011_081203 | 97.90 | 32989 | 5926626 | 5828544 |
|  | MH0069_090103 | 97.97 | 30754 | 5957380 | 5857464 |
|  | MH0060_081222 | 97.97 | 30536 | 5987916 | 5887184 |
|  | MH0012_081203 | 97.98 | 29323 | 6017239 | 5915670 |
|  | MH0043_081214 | 97.98 | 28995 | 6046234 | 5943228 |
|  | MH0066_081223 | 98.07 | 25706 | 6071940 | 5967384 |
|  | MH0052_090103 | 98.08 | 25651 | 6097591 | 5991141 |
|  | MH0050_081223 | 98.08 | 22640 | 6120231 | 6010622 |
|  | MH0031_081223 | 98.08 | 21725 | 6141956 | 6030540 |
|  | MH0003_081203 | 98.08 | 20540 | 6162496 | 6050408 |
|  | MH0053_081222 | 98.09 | 14478 | 6176974 | 6063339 |
|  | MH0046_081230 | 98.09 | 14135 | 6191109 | 6076110 |
|  | MH0061_090103 | 98.10 | 12333 | 6203442 | 6085626 |
|  | MH0042_081226 | 98.10 | 12040 | 6215482 | 6097147 |
|  | MH0078_081222 | 98.10 | 10096 | 6225578 | 6104754 |
|  | MH0086_081223 | 98.10 | 8165 | 6233743 | 6111910 |
|  | MH0040_081223 | 98.10 | 6660 | 6240403 | 6117280 |
|  | MH0036_081226 | 98.10 | 6240 | 6246643 | 6122223 |
|  | MH0030_081224 | 98.10 | 5307 | 6251950 | 6125894 |
|  | MH0037_081226 | 98.10 | 4374 | 6256324 | 6129409 |
|  | MH0054_081222 | 98.10 | 4126 | 6260450 | 6132555 |
|  |  | | | | |
| **Roseburia** | MH0072_081223 | 51.57 | 658740 | 658740 | 92426 |
|  | MH0086_081223 | 83.37 | 409336 | 1068076 | 197906 |
|  | MH0058_081230 | 86.08 | 347943 | 1416019 | 262659 |
|  | MH0073_081223 | 86.94 | 330091 | 1746110 | 320094 |
|  | MH0080_090109 | 87.93 | 285506 | 2031616 | 379622 |
|  | MH0045_081223 | 88.52 | 255792 | 2287408 | 441984 |
|  | MH0076_081223 | 90.96 | 245073 | 2532481 | 516740 |
|  | MH0067_090103 | 91.32 | 216459 | 2748940 | 572808 |
|  | MH0055_081223 | 91.85 | 184804 | 2933744 | 615052 |
|  | MH0035_081223 | 94.82 | 179924 | 3113668 | 723292 |
|  | MH0081_090105 | 94.91 | 166887 | 3280555 | 773661 |
|  | MH0039_081126 | 95.00 | 161283 | 3441838 | 807630 |
|  | MH0077_081223 | 95.23 | 154323 | 3596161 | 867338 |
|  | MH0036_081226 | 95.50 | 142329 | 3738490 | 921779 |
|  | MH0011_081224 | 95.55 | 141380 | 3879870 | 953336 |
|  | MH0070_090104 | 95.84 | 132540 | 4012410 | 1027038 |
|  | MH0003_081224 | 96.14 | 126276 | 4138686 | 1082714 |
|  | MH0063_090103 | 96.22 | 123514 | 4262200 | 1136339 |
|  | MH0075_090104 | 96.48 | 121443 | 4383643 | 1214210 |
|  | MH0083_090105 | 96.52 | 113987 | 4497630 | 1267651 |
|  | MH0082_090105 | 96.58 | 113875 | 4611505 | 1319889 |
|  | MH0030_081224 | 96.63 | 111410 | 4722915 | 1374369 |
|  | MH0064_081223 | 96.71 | 110927 | 4833842 | 1417708 |
|  | MH0049_081223 | 96.73 | 108292 | 4942134 | 1451950 |
|  | MH0031_081223 | 96.88 | 108244 | 5050378 | 1517959 |
|  | MH0068_081223 | 96.92 | 103271 | 5153649 | 1552369 |
|  | MH0016_081224 | 96.96 | 99994 | 5253643 | 1588589 |
|  | MH0002_081224 | 96.99 | 96667 | 5350310 | 1638543 |
|  | MH0044_081222 | 97.01 | 94438 | 5444748 | 1685204 |
|  | MH0051_081226 | 97.04 | 93951 | 5538699 | 1716985 |
|  | MH0085_081223 | 97.05 | 90983 | 5629682 | 1739494 |
|  | MH0084_090109 | 97.05 | 90659 | 5720341 | 1758896 |
|  | MH0014_081224 | 97.11 | 90619 | 5810960 | 1815009 |
|  | MH0050_081223 | 97.13 | 85860 | 5896820 | 1852972 |
|  | MH0069_090103 | 97.15 | 85562 | 5982382 | 1885564 |
|  | MH0054_081222 | 97.21 | 84248 | 6066630 | 1937220 |
|  | MH0028_081223 | 97.21 | 83502 | 6150132 | 1972315 |
|  | MH0059_081230 | 97.26 | 82740 | 6232872 | 2009716 |
|  | MH0065_081223 | 97.33 | 82190 | 6315062 | 2054768 |
|  | MH0052_090103 | 97.35 | 82166 | 6397228 | 2096689 |
|  | MH0024_081224 | 97.36 | 76047 | 6473275 | 2127261 |
|  | MH0011_081203 | 97.36 | 72187 | 6545462 | 2144724 |
|  | MH0041_081222 | 97.37 | 67298 | 6612760 | 2180818 |
|  | MH0042_081226 | 97.43 | 66328 | 6679088 | 2220546 |
|  | MH0032_081224 | 97.44 | 66098 | 6745186 | 2248648 |
|  | MH0061_090103 | 97.45 | 61527 | 6806713 | 2281321 |
|  | MH0033_081224 | 97.47 | 61105 | 6867818 | 2321840 |
|  | MH0021_081224 | 97.47 | 60010 | 6927828 | 2353259 |
|  | MH0062_081223 | 97.49 | 58680 | 6986508 | 2385663 |
|  | MH0025_090120 | 97.50 | 58193 | 7044701 | 2413785 |
|  | MH0043_081214 | 97.51 | 57559 | 7102260 | 2445324 |
|  | MH0016_081203 | 97.51 | 55130 | 7157390 | 2465831 |
|  | MH0078_081222 | 97.52 | 54257 | 7211647 | 2484106 |
|  | MH0074_081223 | 97.53 | 53990 | 7265637 | 2508967 |
|  | MH0040_081223 | 97.54 | 53974 | 7319611 | 2534166 |
|  | MH0012_081224 | 97.63 | 51229 | 7370840 | 2564315 |
|  | MH0037_081226 | 97.64 | 50136 | 7420976 | 2591766 |
|  | MH0014_081203 | 97.65 | 50130 | 7471106 | 2623282 |
|  | MH0071_090104 | 97.66 | 50079 | 7521185 | 2650242 |
|  | MH0048_081226 | 97.67 | 48952 | 7570137 | 2680049 |
|  | MH0047_081223 | 97.67 | 48849 | 7618986 | 2697517 |
|  | MH0079_090104 | 97.68 | 45447 | 7664433 | 2727498 |
|  | MH0009_081224 | 97.70 | 44699 | 7709132 | 2750623 |
|  | MH0066_081223 | 97.74 | 44668 | 7753800 | 2779161 |
|  | MH0060_081222 | 97.74 | 39713 | 7793513 | 2800205 |
|  | MH0046_081230 | 97.75 | 39489 | 7833002 | 2814498 |
|  | MH0038_081226 | 97.75 | 39344 | 7872346 | 2832569 |
|  | MH0003_081203 | 97.76 | 38007 | 7910353 | 2849932 |
|  | MH0053_081222 | 97.76 | 37987 | 7948340 | 2869530 |
|  | MH0020_081224 | 97.77 | 28810 | 7977150 | 2884810 |
|  | MH0057_081222 | 97.77 | 27596 | 8004746 | 2897618 |
|  | MH0012_081203 | 97.78 | 26589 | 8031335 | 2913425 |
