# Supplementary material for: Reconstruction of Bacterial and Viral Genomes from Multiple Metagenomes
Source: Front Microbiol. 2016 Apr 12;7:469. doi: 10.3389/fmicb.2016.00469 (PMC4828583; doi:10.3389/fmicb.2016.00469)
Supplement: Supplementary file 8 [file Table8.DOCX]

**Table S8. Percentage of assembly achieved at different genomic coverage for eight selected bacterial genomes.** All the values are shown in percentage

| **Genus Pool** | **5x** | **10x** | **15x** | **20x** | **25x** | **30x** | **35x** | **40x** | **45x** | **50x** |
| --- | --- | --- | --- | --- | --- | --- | --- | --- | --- | --- |
| **Akkermansia** | 95.35 | 96.79 | 97.51 | 98.02 | 98.26 | 98.38 | 98.47 | 98.52 | 98.56 | 98.61 |
| **Bacteroides** | 39.48 | 57.42 | 68.67 | 76.10 | 81.18 | 84.72 | 87.25 | 89.06 | 90.43 | 91.53 |
| **Bifidobacterium** | 67.60 | 83.59 | 89.22 | 91.73 | 93.12 | 93.98 | 94.57 | 95.09 | 95.54 | 95.94 |
| **Escherichia** | 82.82 | 88.93 | 91.27 | 92.72 | 93.56 | 94.23 | 94.69 | 95.08 | 95.39 | 95.64 |
| **Eubacterium** | 57.74 | 76.88 | 83.95 | 87.08 | 88.70 | 89.47 | 89.95 | 90.29 | 90.61 | 90.86 |
| **Odoribacter** | 90.69 | 94.92 | 96.33 | 97.28 | 97.72 | 97.94 | 98.09 | 98.18 | 98.27 | 98.34 |
| **Parabacteroides** | 91.57 | 94.39 | 95.68 | 96.42 | 96.74 | 96.97 | 97.13 | 97.26 | 97.36 | 97.44 |
| **Roseburia** | 47.44 | 66.13 | 76.46 | 82.87 | 86.91 | 89.62 | 91.38 | 92.61 | 93.45 | 94.08 |
