# Supplementary material for: Reconstruction of Bacterial and Viral Genomes from Multiple Metagenomes
Source: Front Microbiol. 2016 Apr 12;7:469. doi: 10.3389/fmicb.2016.00469 (PMC4828583; doi:10.3389/fmicb.2016.00469)
Supplement: Supplementary file 9 [file Table9.docx]

**Table S9. Percentage of assembly achieved after alignment of virus reads with all the viral genomes available at NCBI.** The virus shown in bold are used for the further analysis.

| **Viral Genome** | **% Assembly** |
| --- | --- |
| **Lactococcus_phage_P008_uid17737** | 97.86 |
| Lactococcus_phage_bIBB29_uid30597 | 97.82 |
| Lactococcus_phage_jj50_uid17759 | 96.96 |
| Lactococcus_phage_sk1_uid14096 | 96.27 |
| Lactococcus_phage_bIL170_uid14087 | 95.71 |
| Lactococcus_phage_jm3_uid213075 | 95.51 |
| Lactococcus_phage_P680_uid213080 | 95.21 |
| Lactococcus_phage_712_uid17757 | 91.81 |
| Lactococcus_phage_jm2_uid213074 | 89.52 |
| Lactococcus_phage_phi7_uid213073 | 88.72 |
| Lactococcus_phage_340_uid213081 | 88.39 |
| **Enterobacteria_phage_EK99P_1_uid260479** | 77.74 |
| **Sodalis_phage_SO_1_uid42597** | 75.38 |
| Lactococcus_phage_bIL67_uid32321 | 74.59 |
| Lactococcus_phage_c2_uid14029 | 73.50 |
| Enterobacteria_phage_SSL_2009a_uid34919 | 73.44 |
| Enterobacteria_phage_JL1_uid179426 | 73.02 |
| **Shigella_phage_EP23_uid80919** | 69.63 |
| Enterobacteria_phage_HK578_uid183138 | 68.41 |
| Enterobacteria_phage_phiX174_sensu_lato_uid14015 | 58.20 |
| **Bacteroides_phage_B40_8_uid31249** | 49.56 |
| Bacteroides_phage_B124_14_uid82753 | 44.46 |
| Streptococcus_phage_Abc2_uid42791 | 43.24 |
| Encephalomyocarditis_virus_uid15307 | 42.35 |
| Wheat_dwarf_virus_uid15478 | 38.67 |
| Streptococcus_phage_DT1_uid15124 | 38.57 |
| Streptococcus_phage_5093_uid38299 | 36.03 |
| Tobacco_leaf_curl_Zimbabwe_virus_uid14119 | 33.96 |
| Tomato_leaf_curl_Barka_virus_uid248098 | 33.96 |
| Tomato_leaf_curl_Sudan_virus_uid14372 | 33.76 |
| Tomato_leaf_curl_Arusha_virus_uid18861 | 33.65 |
| Tomato_leaf_curl_Madagascar_virus_Menabe__Madagascar_Morondova_2001__uid15211 | 33.55 |
| Pepper_yellow_vein_Mali_virus_uid14348 | 33.33 |
| Tomato_leaf_curl_Seychelles_virus_uid18869 | 33.12 |
| Tomato_yellow_leaf_curl_Saudi_virus_uid217879 | 33.01 |
| Lactococcus_phage_r1t_uid14225 | 32.56 |
| Tomato_leaf_curl_Oman_virus_uid52947 | 31.78 |
| Lactococcus_phage_phiLC3_uid14362 | 31.17 |
| Streptococcus_phage_7201_uid14051 | 29.29 |
| Human_papillomavirus_type_6b_uid15454 | 27.61 |
| Streptococcus_phage_Sfi19_uid14045 | 26.88 |
| Streptococcus_phage_858_uid28829 | 23.80 |
| Streptococcus_phage_ALQ13_2_uid42593 | 23.59 |
| Streptococcus_phage_2972_uid15254 | 23.31 |
| Streptococcus_phage_Sfi21_uid14133 | 22.15 |
| Lactococcus_phage_ul36_uid14331 | 21.37 |
| Lactococcus_phage_Tuc2009_uid14131 | 20.74 |
| Streptococcus_phage_TP_778L_uid227111 | 19.93 |
| Streptococcus_phage_20617_uid239271 | 19.56 |
| Lactococcus_phage_P335_sensu_lato_uid14281 | 19.40 |
| Lactococcus_phage_TP901_1_uid14116 | 18.62 |
| Chayote_yellow_mosaic_virus_uid15193 | 18.06 |
| Yersinia_phage_L_413C_uid14280 | 16.61 |
| Enterobacteria_phage_P2_uid14035 | 16.59 |
| Tomato_leaf_curl_Nigeria_virus_Nigeria_2006__uid34815 | 16.20 |
| Streptococcus_phage_Sfi11_uid14054 | 15.18 |
| Streptococcus_phage_TP_J34_uid188154 | 14.85 |
| Streptococcus_phage_O1205_uid14226 | 14.81 |
| Tobacco_vein_clearing_virus_uid14150 | 14.50 |
| Enterobacteria_phage_fiAA91_ss_uid226726 | 14.34 |
| Tomato_leaf_curl_Cameroon_virus_uid42743 | 14.19 |
| Chicken_anemia_virus_uid15484 | 12.92 |
| Human_papillomavirus___18_uid15506 | 12.82 |
| Enterobacteria_phage_phiEcoM_GJ1_uid27979 | 11.68 |
| Yersinia_pestis_phage_phiA1122_uid14332 | 11.51 |
| Saccharomyces_cerevisiae_killer_virus_M1_uid14678 | 10.74 |
| Lactococcus_phage_bIL286_uid14397 | 10.16 |
| Enterobacteria_phage_HK022_uid14048 | 9.85 |
| Lactococcus_phage_BK5_T_uid15244 | 9.80 |
| Enterobacteria_phage_P4_uid14414 | 9.41 |
| Bacillus_virus_1_uid20397 | 9.07 |
| Enterobacteria_phage_HK97_uid14592 | 8.84 |
| Enterobacteria_phage_HK633_uid183143 | 8.72 |
| Enterobacteria_phage_HK544_uid183160 | 8.51 |
| Enterobacteria_phage_mEpX2_uid183150 | 8.39 |
| Escherichia_phage_HK75_uid76733 | 8.27 |
| Enterobacteria_phage_HK446_uid183141 | 8.16 |
| Propionibacterium_phage_P105_uid177533 | 8.06 |
| Propionibacterium_phage_ATCC29399B_C_uid177539 | 8.04 |
| Propionibacterium_phage_PHL111M01_uid219111 | 8.04 |
| Enterobacteria_phage_HK542_uid183159 | 8.02 |
| Enterobacteria_phage_HK620_uid14115 | 7.97 |
| Enterobacteria_phage_Sf6_uid14498 | 7.93 |
| Enterobacteria_phage_HK106_uid183158 | 7.73 |
| Enterobacteria_phage_HK140_uid183139 | 7.61 |
| Enterobacterial_phage_mEp234_uid183153 | 7.54 |
| Stx2_converting_phage_1717_uid32213 | 7.54 |
| Enterobacteria_phage_mEp235_uid183146 | 7.50 |
| Propionibacterium_phage_P100_A_uid177535 | 7.47 |
| Enterobacteria_phage_YYZ_2008_uid32231 | 7.44 |
| Streptococcus_phage_EJ_1_uid14604 | 7.43 |
| Human_adenovirus_C_uid14518 | 7.24 |
| Enterobacteria_phage_WV8_uid38281 | 7.16 |
| Enterobacterial_phage_mEp390_uid183154 | 7.09 |
| Enterobacteria_phage_mEpX1_uid183149 | 7.01 |
| Enterobacteria_phage_Felix_01_uid14323 | 6.89 |
| Enterobacteria_phage_BP_4795_uid14287 | 6.78 |
| Enterobacteria_phage_IME10_uid181235 | 6.62 |
| Thermus_phage_IN93_uid14235 | 6.28 |
| Enterobacteria_phage_If1_uid14039 | 6.18 |
| Propionibacterium_phage_PHL113M01_uid219107 | 6.15 |
| Propionibacterium_phage_ATCC29399B_T_uid177538 | 6.13 |
| Lactococcus_phage_BM13_uid213076 | 6.09 |
| HCBI8_215_virus_uid257701 | 5.89 |
| Enterobacteria_phage_SfV_uid14162 | 5.85 |
| Mongoose_feces_associated_gemycircularvirus_c_uid281404 | 5.68 |
| Avian_endogenous_retrovirus_EAV_HP_uid15213 | 5.49 |
| Enterobacteria_phage_ST104_uid14499 | 5.44 |
| Enterobacteria_phage_mEp237_uid183147 | 5.18 |
| Enterobacteria_phage_ID18_sensu_lato_uid16628 | 5.17 |
| Streptococcus_phage_PH10_uid38365 | 5.07 |
| Tomato_yellow_leaf_curl_virus_uid15182 | 5.07 |
| Pseudomonas_phage_phi_2_uid42717 | 4.83 |
| Haemophilus_phage_HP1_uid14078 | 4.82 |
| Mycobacterium_phage_Liefie_uid240722 | 4.70 |
| Mycobacterium_phage_BPs_uid29917 | 4.70 |
| Porcine_endogenous_retrovirus_E_uid14126 | 4.68 |
| Mycobacterium_phage_DNAIII_uid213079 | 4.65 |
| Mycobacterium_phage_Halo_uid17147 | 4.64 |
| Mycobacterium_phage_Leo_uid209361 | 4.61 |
| Streptococcus_phage_SM1_uid14295 | 4.61 |
| Salmonella_phage_ST160_uid61857 | 4.55 |
| Shigella_phage_SfII_uid213070 | 4.51 |
| Human_papillomavirus_type_34_uid15509 | 4.43 |
| Enterobacterial_phage_mEp213_uid183152 | 4.43 |
| Enterobacteria_phage_HK630_uid183142 | 4.36 |
| Tomato_leaf_curl_Java_virus_Ageratum__satellite_DNA_uid14452 | 4.34 |
| Lactococcus_phage_KSY1_uid20783 | 4.26 |
| Enterobacteria_phage_P22_uid14478 | 4.17 |
| Streptococcus_phage_PH15_uid30161 | 4.11 |
| Tomato_yellow_dwarf_disease_associated_satellite_DNA_beta_Kochi__uid20983 | 4.06 |
| Salmonella_phage_FSL_SP_004_uid212714 | 4.02 |
| Salmonella_phage_epsilon34_uid33779 | 3.96 |
| Lactococcus_phage_bIL309_uid14338 | 3.91 |
| Salmonella_phage_vB_SemP_Emek_uid171957 | 3.88 |
| Enterobacteria_phage_mEp043_c_1_uid183145 | 3.83 |
| Salmonella_phage_SE1_uid33483 | 3.70 |
| Enterobacteria_phage_phiP27_uid14599 | 3.69 |
| Salmonella_phage_c341_uid39795 | 3.66 |
| Enterobacteria_phage_lambda_uid14204 | 3.57 |
| Stx2_converting_phage_86_uid17979 | 3.56 |
| Simian_adenovirus_49_uid64487 | 3.55 |
| Enterobacteria_phage_VT2_Sakai_uid14480 | 3.53 |
| Thermus_phage_P23_77_uid40235 | 3.53 |
| Ludwigia_leaf_distortion_betasatellite__India_Amadalavalasa_Hibiscus_2007__uid29233 | 3.53 |
| Salmonella_phage_SPN9CC_uid167665 | 3.52 |
| Chickpea_redleaf_virus_uid60625 | 3.52 |
| Enterobacteria_phage_ST64T_uid14230 | 3.50 |
| Enterobacteria_phage_933W_uid14043 | 3.48 |
| Haemophilus_phage_HP2_uid14231 | 3.48 |
| Enterobacteria_phage_Min27_uid29143 | 3.37 |
| Alternanthera_yellow_vein_virus_satellite_DNA_beta_uid19833 | 3.35 |
| Stx2_converting_phage_I_uid14167 | 3.30 |
| Stx2_converting_phage_II_uid14310 | 3.23 |
| Enterobacteria_phage_HK629_uid183144 | 3.20 |
| Simian_adenovirus_18_uid218146 | 3.16 |
| Ball_python_nidovirus_uid259246 | 3.10 |
| Actinomyces_phage_Av_1_uid20057 | 3.08 |
| Enterobacteria_phage_T1_uid14496 | 3.08 |
| Burkholderia_phage_phi644_2_uid62941 | 3.01 |
| Shigella_phage_SfIV_uid227000 | 2.96 |
| Enterobacteria_phage_N15_uid14086 | 2.91 |
| Gemycircularvirus_SL1_uid281396 | 2.88 |
| Enterobacteria_phage_G4_sensu_lato_uid14318 | 2.81 |
| Enterobacteria_phage_ES18_uid15174 | 2.78 |
| Mycobacterium_phage_Jobu08_uid209074 | 2.67 |
| Enterobacteria_phage_P1_uid14493 | 2.58 |
| Lactobacillus_phage_KC5a_uid16663 | 2.55 |
| Enterobacteria_phage_ID2_Moscow_ID_2001_uid16591 | 2.55 |
| Rosellinia_necatrix_partitivirus_2_uid188731 | 2.52 |
| Stx1_converting_phage_uid14293 | 2.48 |
| Mycobacterium_phage_Bxz2_uid14275 | 2.48 |
| Enterobacteria_phage_HK225_uid183140 | 2.44 |
| Enterobacteria_phage_alpha3_uid14570 | 2.39 |
| Enterobacteria_phage_WA13_sensu_lato_uid16595 | 2.37 |
| Enterobacteria_phage_PsP3_uid14345 | 2.36 |
| Torque_teno_midi_virus_1_uid19131 | 2.33 |
| Lactococcus_phage_bIL285_uid14111 | 2.30 |
| Pseudomonas_phage_vB_PaeS_PMG1_uid82649 | 2.29 |
| Enterobacteria_phage_St_1_uid38669 | 2.28 |
| Human_papillomavirus_54_uid15466 | 2.25 |
| Enterobacteria_phage_cdtI_uid19737 | 2.24 |
| Oat_dwarf_virus_uid30037 | 2.22 |
| Phytophthora_infestans_RNA_virus_1_uid40329 | 2.17 |
| Lactobacillus_phage_J_1_uid227005 | 2.14 |
| Lactobacillus_phage_PL_1_uid227007 | 2.14 |
| Escherichia_phage_TL_2011c_uid181075 | 2.10 |
| Staphylococcus_phage_PH15_uid18525 | 2.09 |
| Dill_cryptic_virus_1_uid225921 | 2.04 |
| Streptococcus_phage_Cp_1_uid14584 | 2.04 |
| Enterobacteria_phage_TLS_uid19775 | 2.01 |
| Lactobacillus_phage_A2_uid14602 | 2.00 |
| Propionibacterium_phage_P104A_uid177532 | 1.99 |
| Human_adenovirus_D_uid14535 | 1.99 |
| Aleutian_mink_disease_virus_uid14077 | 1.99 |
| Propionibacterium_phage_PA6_uid19767 | 1.97 |
| Propionibacterium_phage_P100D_uid177534 | 1.97 |
| Propionibacterium_phage_PHL037M02_uid219116 | 1.96 |
| Propionibacterium_phage_P9_1_uid177529 | 1.96 |
| Propionibacterium_phage_P101A_uid177531 | 1.95 |
| Salmonella_phage_vB_SosS_Oslo_uid171977 | 1.95 |
| Felis_catus_papillomavirus_4_uid221115 | 1.95 |
| Cronobacter_phage_ENT39118_uid184168 | 1.94 |
| Oyster_mushroom_spherical_virus_uid14951 | 1.92 |
| Sweet_potato_caulimo_like_virus_uid65307 | 1.91 |
| Human_adenovirus_54_uid39353 | 1.91 |
| Propionibacterium_phage_PHL010M04_uid219117 | 1.90 |
| Simian_adenovirus_1_uid14626 | 1.80 |
| Enterobacteria_phage_phiV10_uid16381 | 1.79 |
| Propionibacterium_phage_PAD20_uid66341 | 1.79 |
| Lactobacillus_phage_LL_H_uid19803 | 1.78 |
| Lactococcus_phage_949_uid64559 | 1.78 |
| Propionibacterium_phage_PHL060L00_uid219122 | 1.78 |
| Mycobacterium_phage_Trixie_uid240732 | 1.77 |
| Human_papillomavirus_type_10_uid15504 | 1.77 |
| Propionibacterium_phage_PHL067M10_uid219115 | 1.77 |
| Shigella_phage_Shfl1_uid66345 | 1.76 |
| Propionibacterium_phage_PHL071N05_uid219109 | 1.75 |
| Propionibacterium_phage_PHL112N00_uid219110 | 1.75 |
| Rhizoctonia_solani_dsRNA_virus_2_uid240575 | 1.73 |
| Propionibacterium_phage_P1_1_uid177537 | 1.73 |
| Escherichia_phage_P13374_uid177543 | 1.69 |
| Klebsiella_phage_phiKO2_uid14495 | 1.66 |
| Torque_teno_midi_virus_2_uid48185 | 1.65 |
| Ranid_herpesvirus_1_uid17181 | 1.65 |
| Titi_monkey_adenovirus_ECC_2011_uid192854 | 1.64 |
| Jingmen_Tick_Virus_uid247973 | 1.64 |
| Enterococcus_phage_phiFL3A_uid42787 | 1.63 |
| Pseudocowpox_virus_uid45973 | 1.61 |
| Pseudomonas_phage_119X_uid16385 | 1.61 |
| Pseudomonas_phage_PaP2_uid14377 | 1.59 |
| Hibiscus_latent_Singapore_virus_uid17573 | 1.57 |
| Lactobacillus_phage_Lrm1_uid30879 | 1.56 |
| Escherichia_phage_D108_uid42515 | 1.56 |
| Escherichia_phage_ADB_2_uid183155 | 1.51 |
| Propionibacterium_phage_PHL114L00_uid219112 | 1.50 |
| Mycobacterium_phage_Wile_uid240730 | 1.48 |
| Lactobacillus_johnsonii_prophage_Lj771_uid28145 | 1.47 |
| Human_herpesvirus_7_uid14625 | 1.39 |
| Mycobacterium_phage_D29_uid14203 | 1.35 |
| Mycobacterium_phage_Chy4_uid206477 | 1.35 |
| Human_adenovirus_E_uid15152 | 1.33 |
| Propionibacterium_phage_PAS50_uid66339 | 1.33 |
| Chimpanzee_adenovirus_Y25_uid162489 | 1.32 |
| Fathead_minnow_picornavirus_uid237541 | 1.31 |
| Propionibacterium_phage_P14_4_uid177530 | 1.29 |
| Escherichia_phage_TL_2011b_uid181074 | 1.29 |
| Propionibacterium_phage_P100_1_uid177536 | 1.29 |
| Enterobacteria_phage_mEp460_uid183148 | 1.29 |
| Mycobacterium_phage_Chy5_uid206476 | 1.25 |
| Lactococcus_phage_bIL312_uid14113 | 1.21 |
| Human_adenovirus_F_uid14487 | 1.21 |
| Tomato_yellow_leaf_curl_Malaga_virus_uid14239 | 1.19 |
| Clostridium_phage_phi_CD119_uid16662 | 1.18 |
| Cycad_leaf_necrosis_virus_uid30835 | 1.18 |
| Listeria_phage_LMSP_25_uid253225 | 1.16 |
| Streptococcus_phage_K13_uid253223 | 1.16 |
| Ursus_maritimus_papillomavirus_1_uid29915 | 1.12 |
| Hepatitis_C_virus_genotype_2_uid20937 | 1.12 |
| Hepatitis_C_virus_uid15432 | 1.11 |
| Rabbit_vesivirus_uid18289 | 1.11 |
| Mycobacterium_phage_Artemis2UCLA_uid230596 | 1.08 |
| Enterobacteria_phage_P88_uid271780 | 1.08 |
| Dulcamara_mottle_virus_uid16188 | 1.08 |
| Staphylococcus_phage_ROSA_uid15274 | 1.07 |
| Mycobacterium_phage_CloudWang3_uid230588 | 1.06 |
| Mycobacterium_phage_Zaka_uid230589 | 1.06 |
| Lactococcus_phage_P092_uid251389 | 1.06 |
| Sputnik_virophage_2_uid243067 | 1.04 |
| Sputnik_virophage_3_uid243065 | 1.04 |
| Sputnik_virophage_uid30929 | 1.04 |
| Sclerotinia_sclerotiorum_debilitation_associated_RNA_virus_uid15717 | 1.04 |
| Lactococcus_phage_bIL311_uid14139 | 1.02 |
| Streptococcus_phage_SMP_uid18529 | 1.02 |
| Enterobacteria_phage_Mu_uid14105 | 1.01 |
| Clostridium_phage_c_st_uid16151 | 1.00 |
| Enterobacteria_phage_epsilon15_uid14285 | 0.97 |
| Human_papillomavirus_type_9_uid15456 | 0.97 |
| Cotesia_congregata_bracovirus_uid14556 | 0.96 |
| Carp_picornavirus_1_uid232646 | 0.94 |
| Canine_papillomavirus_2_uid14551 | 0.93 |
| Nyamanini_virus_uid38109 | 0.92 |
| Cyprinid_herpesvirus_1_uid181227 | 0.92 |
| Mycobacterium_phage_Adzzy_uid215109 | 0.91 |
| Mycobacterium_phage_Che12_uid17143 | 0.91 |
| Simian_enterovirus_A_uid15371 | 0.91 |
| Saffold_virus_uid19577 | 0.90 |
| Torque_teno_virus_28_uid48145 | 0.89 |
| Human_adenovirus_A_uid14517 | 0.89 |
| Cronobacter_phage_ESP2949_1_uid181234 | 0.89 |
| Human_adenovirus_B_uid15150 | 0.88 |
| Human_adenovirus_B_uid31177 | 0.87 |
| Lactococcus_phage_P087_uid37887 | 0.87 |
| Mycobacterium_phage_Pukovnik_uid30521 | 0.87 |
| Enterococcus_phage_vB_Efae230P_4_uid266788 | 0.87 |
| Duck_picornavirus_TW90A_uid15039 | 0.84 |
| Hibiscus_latent_Fort_Pierce_virus_uid264910 | 0.84 |
| Sclerotinia_sclerotiorum_debilitation_associated_RNA_virus_2_uid252621 | 0.83 |
| Haemophilus_phage_Aaphi23_uid15228 | 0.83 |
| Erwinia_phage_ENT90_uid184166 | 0.83 |
| Edwardsiella_phage_MSW_3_uid185428 | 0.82 |
| Hepatitis_C_virus_genotype_6_uid20939 | 0.81 |
| Streptococcus_phage_MM1_uid14601 | 0.78 |
| Mamestra_brassicae_multiple_nucleopolyhedrovirus_uid240576 | 0.77 |
| Human_papillomavirus_type_16_uid15505 | 0.76 |
| Gallid_herpesvirus_3_uid14103 | 0.76 |
| Bacillus_phage_BCJA1c_uid14548 | 0.75 |
| Pepino_mosaic_virus_uid15125 | 0.74 |
| Eel_picornavirus_1_uid219023 | 0.74 |
| Mamastrovirus_3_uid264920 | 0.73 |
| Lactobacillus_phage_AQ113_uid188466 | 0.72 |
| Hyphantria_cunea_nucleopolyhedrovirus_uid16343 | 0.71 |
| Mycobacterium_phage_Blue7_uid240757 | 0.68 |
| unidentified_phage_uid249088 | 0.68 |
| Burkholderia_phage_KS9_uid39771 | 0.67 |
| Enterobacteria_phage_K1F_uid15880 | 0.64 |
| Cutthroat_trout_virus_uid66895 | 0.64 |
| Human_herpesvirus_6B_uid14422 | 0.64 |
| Planaria_asexual_strain_specific_virus_like_element_type_1_uid14140 | 0.63 |
| Porcine_astrovirus_3_uid181247 | 0.62 |
| Lactococcus_phage_phiL47_uid240042 | 0.61 |
| Salmonella_phage_E1_uid29079 | 0.61 |
| Lactobacillus_phage_phiJB_uid227128 | 0.61 |
| Simian_adenovirus_C_uid200956 | 0.60 |
| Lactobacillus_phage_phiAT3_uid14475 | 0.60 |
| Shigella_phage_pSf_1_uid206484 | 0.60 |
| Staphylococcus_phage_GH15_uid181069 | 0.59 |
| Pseudomonas_phage_F10_uid16383 | 0.59 |
| Lactococcus_phage_1706_uid29283 | 0.59 |
| Tick_borne_encephalitis_virus_uid15335 | 0.59 |
| Porcine_astrovirus_4_uid240319 | 0.59 |
| Lactococcus_phage_Q54_uid17739 | 0.59 |
| Porcine_astrovirus_2_uid240318 | 0.59 |
| Yersinia_phage_Berlin_uid18481 | 0.56 |
| Gallid_herpesvirus_2_uid14402 | 0.56 |
| Seneca_valley_virus_uid32193 | 0.56 |
| Streptococcus_phage_M102_uid38845 | 0.55 |
| Streptococcus_pyogenes_phage_315_2_uid14528 | 0.55 |
| Burkholderia_phage_Bcep43_uid14411 | 0.54 |
| Burkholderia_phage_Bcep781_uid14405 | 0.54 |
| Enterobacteria_phage_T5_uid15143 | 0.54 |
| Acidianus_filamentous_virus_9_uid29195 | 0.54 |
| Dasheen_mosaic_virus_uid15388 | 0.53 |
| Rice_tungro_spherical_virus_uid15332 | 0.52 |
| Streptomyce_phage_TG1_uid177524 | 0.51 |
| Choristoneura_occidentalis_granulovirus_uid17097 | 0.51 |
| Crohivirus_uid267248 | 0.51 |
| Turkey_astrovirus_2_uid14954 | 0.50 |
| Bat_hepevirus_uid172460 | 0.50 |
| Yersinia_phage_Yepe2_uid62965 | 0.49 |
| Streptococcus_phage_DCC1738_uid253219 | 0.49 |
| Bidens_mottle_virus_uid50559 | 0.48 |
| Salivirus_FHB_uid262543 | 0.47 |
| Formica_exsecta_virus_1_uid231521 | 0.47 |
| Moroccan_watermelon_mosaic_virus_uid27897 | 0.46 |
| Bovine_rhinovirus_2_uid28835 | 0.45 |
| Zucchini_tigre_mosaic_virus_uid232774 | 0.45 |
| Canine_picodicistrovirus_uid201444 | 0.44 |
| Pigeon_adenovirus_1_uid255102 | 0.44 |
| Human_herpesvirus_6A_uid14462 | 0.44 |
| Gryllus_bimaculatus_nudivirus_uid19181 | 0.44 |
| Grapevine_Bulgarian_latent_virus_uid66553 | 0.43 |
| Salmonella_phage_Vi06_uid64609 | 0.43 |
| Sclerotinia_sclerotiorum_hypovirus_1_uid72389 | 0.43 |
| Iodobacteriophage_phiPLPE_uid30965 | 0.43 |
| Staphylococcus_phage_phiSA012_uid239723 | 0.42 |
| Edwardsiella_phage_PEi21_uid230645 | 0.42 |
| Colobus_guereza_papillomavirus_type_2_uid68289 | 0.42 |
| Enterobacteria_phage_9g_uid248627 | 0.42 |
| Morganella_phage_MmP1_uid30793 | 0.41 |
| Lactobacillus_phage_phiJL_1_uid15156 | 0.41 |
| Mycobacterium_phage_Charlie_uid240739 | 0.41 |
| Bacillus_phage_WBeta_uid16329 | 0.41 |
| Cyanophage_S_TIM5_uid181237 | 0.40 |
| Sicinivirus_1_uid242975 | 0.40 |
| Salmonella_phage_ST64B_uid14228 | 0.40 |
| Mouse_kobuvirus_M_5_USA_2010_uid72383 | 0.39 |
| Abalone_shriveling_syndrome_associated_virus_uid33141 | 0.39 |
| Alcelaphine_herpesvirus_2_uid253391 | 0.38 |
| Nam_Dinh_virus_uid71143 | 0.38 |
| Falconid_herpesvirus_1_uid253588 | 0.38 |
| Equid_herpesvirus_2_uid14457 | 0.37 |
| Garlic_common_latent_virus_uid78925 | 0.37 |
| Clostridium_phage_39_O_uid32103 | 0.37 |
| Adoxophyes_orana_granulovirus_uid14298 | 0.36 |
| Yersinia_phage_Yep_phi_uid240772 | 0.36 |
| Whataroa_virus_uid88119 | 0.36 |
| Klebsiella_phage_KP32_uid42779 | 0.36 |
| Southern_elephant_seal_virus_uid88117 | 0.36 |
| Chilli_ringspot_virus_uid73825 | 0.35 |
| Enterobacteria_phage_285P_uid64539 | 0.35 |
| Clostridium_phage_phiC2_uid19153 | 0.35 |
| Bacillus_phage_Fah_uid16382 | 0.35 |
| Mycobacterium_phage_Pacc40_uid32017 | 0.35 |
| Bacillus_phage_Cherry_uid15784 | 0.35 |
| Clostridium_phage_phiMMP02_uid179416 | 0.35 |
| Cyprinid_herpesvirus_3_uid19059 | 0.35 |
| Enterobacteria_phage_13a_uid30603 | 0.34 |
| Escherichia_phage_bV_EcoS_AKFV33_uid167572 | 0.34 |
| Enterobacteria_phage_K30_uid68413 | 0.34 |
| Enterococcus_phage_phiEf11_uid42943 | 0.34 |
| Saimiriine_herpesvirus_1_uid54017 | 0.34 |
| Bacillus_phage_Gamma_uid15783 | 0.34 |
| Klebsiella_phage_K11_uid62963 | 0.34 |
| Ictalurid_herpesvirus_1_uid14018 | 0.34 |
| Escherichia_phage_rv5_uid30613 | 0.33 |
| Posavirus_2_uid240238 | 0.33 |
| Pseudomonas_phage_D3_uid14500 | 0.33 |
| Enterobacteria_phage_SPC35_uid64605 | 0.33 |
| Laodelphax_striatella_honeydew_virus_1_uid239909 | 0.33 |
| Escherichia_phage_vB_EcoS_FFH1_uid248533 | 0.33 |
| Mycobacterium_phage_Job42_uid209072 | 0.33 |
| Erwinia_phage_phiEa100_uid184154 | 0.33 |
| Ndumu_virus_uid88115 | 0.33 |
| Nile_crocodilepox_virus_uid16798 | 0.32 |
| Enterobacteria_phage_vB_EcoM_FV3_uid181219 | 0.32 |
| Mycobacteriophage_Velveteen_uid215123 | 0.32 |
| Lactobacillus_phage_Ld25A_uid266648 | 0.32 |
| Clostridium_phage_phiCD27_uid32323 | 0.31 |
| Staphylococcus_phage_S25_3_uid230094 | 0.31 |
| Brochothrix_phage_A9_uid64547 | 0.31 |
| Vibrio_phage_pYD38_A_uid209063 | 0.31 |
| Trichoplusia_ni_ascovirus_2c_uid18003 | 0.30 |
| Bebaru_virus_uid88121 | 0.29 |
| Staphylococcus_phage_S25_4_uid230071 | 0.29 |
| Phthorimaea_operculella_granulovirus_uid14202 | 0.29 |
| Escherichia_phage_2_JES_2013_uid219124 | 0.29 |
| Pseudoalteromonas_phage_pYD6_A_uid195478 | 0.29 |
| Clostridium_phage_phi3626_uid14166 | 0.29 |
| Staphylococcus_phage_Sb_1_uid230853 | 0.29 |
| Cyprinid_herpesvirus_2_uid181228 | 0.28 |
| Eliat_virus_uid175588 | 0.28 |
| Streptococcus_pyogenes_phage_315_3_uid14529 | 0.28 |
| Mycobacterium_phage_SiSi_uid206026 | 0.28 |
| Enterobacteria_phage_RB51_uid37819 | 0.28 |
| Lactobacillus_phage_Ld17_uid266649 | 0.28 |
| Mycobacterium_phage_GUmbie_uid240735 | 0.28 |
| Sclerotinia_sclerotiorum_hypovirus_2_uid229826 | 0.27 |
| Cercopithecine_herpesvirus_2_uid14558 | 0.27 |
| Goatpox_virus_Pellor_uid14197 | 0.27 |
| Oryctes_rhinoceros_virus_uid32781 | 0.27 |
| Cellulophaga_phage_phi19_1_uid212942 | 0.26 |
| Lactococcus_phage_P078_uid251390 | 0.26 |
| Bovine_respiratory_coronavirus_bovine_US_OH_440_TC_1996_uid39333 | 0.26 |
| Lactobacillus_phage_Lc_Nu_uid16114 | 0.26 |
| Enterobacteria_phage_RB14_uid37825 | 0.26 |
| Cryphonectria_hypovirus_1_uid14664 | 0.25 |
| Aeromonas_phage_31_uid15416 | 0.25 |
| White_bream_virus_uid18013 | 0.25 |
| Shigella_phage_phiSboM_AG3_uid42937 | 0.25 |
| Cydia_pomonella_granulovirus_uid14118 | 0.24 |
| Shrimp_white_spot_syndrome_virus_uid14616 | 0.24 |
| Buzura_suppressaria_nucleopolyhedrovirus_uid237559 | 0.24 |
| Staphylococcus_phage_vB_SauM_Romulus_uid195528 | 0.24 |
| Agrotis_ipsilon_multiple_nucleopolyhedrovirus_uid32171 | 0.24 |
| Listeria_phage_LMTA_148_uid260452 | 0.24 |
| Escherichia_phage_HK639_uid76729 | 0.24 |
| Escherichia_phage_vB_EcoM_FFH2_uid248531 | 0.23 |
| Equid_herpesvirus_3_uid259985 | 0.23 |
| Staphylococcus_phage_K_uid14479 | 0.23 |
| Staphylococcus_phage_vB_SauM_Remus_uid215669 | 0.23 |
| Molluscum_contagiosum_virus_subtype_1_uid14328 | 0.23 |
| Bordetella_phage_BPP_1_uid14353 | 0.22 |
| Bordetella_phage_BIP_1_uid14359 | 0.22 |
| Bordetella_phage_BMP_1_uid14358 | 0.22 |
| Streptococcus_phage_phi3396_uid18859 | 0.22 |
| Agrotis_segetum_nucleopolyhedrovirus_uid16661 | 0.22 |
| Geobacillus_phage_GBSV1_uid17775 | 0.22 |
| Synechococcus_phage_Syn19_uid64709 | 0.22 |
| Staphylococcus_phage_G1_uid15261 | 0.22 |
| Lymphocystis_disease_virus___isolate_China_uid14472 | 0.22 |
| Enterobacteria_phage_RB32_uid17997 | 0.22 |
| Escherichia_phage_vB_EcoP_PhAPEC7_uid260485 | 0.22 |
| Meleagrid_herpesvirus_1_uid14106 | 0.22 |
| Paramecium_bursaria_Chlorella_virus_NY2A_uid20989 | 0.22 |
| Staphylococcus_phage_Twort_uid15246 | 0.22 |
| Erwinia_phage_phiEa104_uid64759 | 0.22 |
| Erwinia_phage_phiEa21_4_uid33537 | 0.22 |
| Aeromonas_phage_44RR2_8t_uid14321 | 0.22 |
| Pseudomonas_phage_201phi2_1_uid30097 | 0.22 |
| Macacine_herpesvirus_1_uid14489 | 0.21 |
| Alcelaphine_herpesvirus_1_uid14099 | 0.21 |
| Escherichia_phage_wV7_uid181232 | 0.21 |
| Spodoptera_exigua_MNPV_uid14134 | 0.21 |
| Burkholderia_phage_BcepC6B_uid14379 | 0.21 |
| Enterobacteria_phage_ime09_uid181233 | 0.21 |
| Helicoverpa_armigera_NPV_uid14615 | 0.21 |
| Fowl_adenovirus_D_uid14523 | 0.20 |
| Ectocarpus_siliculosus_virus_1_uid14114 | 0.20 |
| Helicoverpa_armigera_NPV_NNg1_uid32205 | 0.20 |
| Thermus_phage_phiYS40_uid18277 | 0.19 |
| Kluyvera_phage_Kvp1_uid32673 | 0.19 |
| Alphacoronavirus_2_uid241029 | 0.19 |
| Hemileuca_nucleopolyhedrovirus_uid214353 | 0.19 |
| Synechococcus_phage_S_RIM2_R1_1999_uid195488 | 0.19 |
| Enterobacteria_phage_BA14_uid30599 | 0.19 |
| Ovine_herpesvirus_2_uid16234 | 0.19 |
| Flavobacterium_phage_11b_uid14565 | 0.19 |
| Listeria_phage_List_36_uid253221 | 0.19 |
| Burkholderia_phage_Bcep22_uid14335 | 0.18 |
| Aeromonas_phage_25_uid17105 | 0.18 |
| Anticarsia_gemmatalis_nucleopolyhedrovirus_uid17995 | 0.18 |
| Salmonella_phage_SPN3UB_uid181984 | 0.18 |
| Orgyia_pseudotsugata_MNPV_uid14084 | 0.18 |
| Lactobacillus_phage_Lb338_1_uid36611 | 0.18 |
| Lactobacillus_phage_phiadh_uid14588 | 0.18 |
| Bacillus_phage_IEBH_uid31057 | 0.18 |
| Colwellia_phage_9A_uid169428 | 0.18 |
| Spodoptera_litura_NPV_uid14138 | 0.18 |
| Listeria_phage_LP_125_uid212716 | 0.18 |
| Synechococcus_phage_S_RIM8_A_HR1_uid192853 | 0.18 |
| Human_coronavirus_229E_uid14913 | 0.18 |
| Salmonella_phage_SETP13_uid226727 | 0.17 |
| Staphylococcus_phage_vB_SepS_SEP9_uid240035 | 0.17 |
| Tupaiid_herpesvirus_1_uid14597 | 0.17 |
| Papiine_herpesvirus_2_uid16246 | 0.17 |
| Mycobacterium_phage_Myrna_uid31279 | 0.17 |
| Streptococcus_phage_IC1_uid253220 | 0.17 |
| Mycobacterium_phage_PBI1_uid17165 | 0.17 |
| Mycobacterium_phage_Adjutor_uid29919 | 0.17 |
| Mycobacterium_phage_Butterscotch_uid32007 | 0.17 |
| Acidianus_bottle_shaped_virus_uid19605 | 0.17 |
| Synechococcus_phage_syn9_uid17541 | 0.17 |
| Simian_adenovirus_DM_2014_uid267780 | 0.17 |
| Caviid_herpesvirus_2_uid188730 | 0.17 |
| Bacteriophage_APSE_2_uid32705 | 0.17 |
| Rhodobacter_phage_RcapNL_uid192926 | 0.16 |
| Taterapox_virus_uid17483 | 0.16 |
| Suid_herpesvirus_1_uid14424 | 0.16 |
| Listeria_phage_LP_048_uid253330 | 0.16 |
| Clostridium_phage_CDMH1_uid248532 | 0.16 |
| Penaeus_monodon_nudivirus_uid259190 | 0.16 |
| Fowl_adenovirus_E_uid62241 | 0.16 |
| Bacillus_phage_SPO1_uid32379 | 0.16 |
| Human_herpesvirus_8_uid14158 | 0.16 |
| Aureococcus_anophagefferens_virus_MM_2014_uid258005 | 0.15 |
| Enterobacteria_phage_EPS7_uid29287 | 0.15 |
| Listeria_phage_A511_uid20793 | 0.15 |
| Edwardsiella_phage_eiAU_uid240025 | 0.15 |
| Staphylococcus_phage_6ec_uid253318 | 0.15 |
| Enterobacteria_phage_T4_uid14044 | 0.15 |
| Duck_adenovirus_2_uid255793 | 0.15 |
| Escherichia_phage_vB_EcoP_PhAPEC5_uid260483 | 0.15 |
| Cryptophlebia_leucotreta_granulovirus_uid14302 | 0.14 |
| Listeria_phage_vB_LmoM_AG20_uid195527 | 0.14 |
| Emiliania_huxleyi_virus_86_uid15618 | 0.14 |
| Mycobacterium_phage_LinStu_uid240727 | 0.14 |
| Staphylococcus_phage_JD007_uid183162 | 0.14 |
| Phaeocystis_globosa_virus_uid206023 | 0.13 |
| Vibrio_phage_VP882_uid18851 | 0.13 |
| Cyanophage_Syn30_uid198437 | 0.13 |
| Listeria_phage_LP_083_2_uid253387 | 0.13 |
| Paramecium_bursaria_Chlorella_virus_1_uid14564 | 0.13 |
| Monkeypox_virus_Zaire_96_I_16_uid15142 | 0.13 |
| Bovine_herpesvirus_5_uid14313 | 0.13 |
| Amsacta_moorei_entomopoxvirus__L__uid14097 | 0.13 |
| Staphylococcus_phage_P108_uid266646 | 0.13 |
| Thermus_phage_TMA_uid72385 | 0.13 |
| Clostridium_phage_phiCP34O_uid181211 | 0.13 |
| Clostridium_phage_phiCP26F_uid181251 | 0.13 |
| Enterobacteria_phage_vB_EcoM_ACG_C40_uid179415 | 0.13 |
| Clostridium_phage_phiCP13O_uid181210 | 0.13 |
| Cyanophage_KBS_M_1A_uid195500 | 0.13 |
| Elephantid_herpesvirus_1_uid192609 | 0.13 |
| Epinotia_aporema_granulovirus_uid177904 | 0.12 |
| Bovine_herpesvirus_6_uid252717 | 0.12 |
| Burkholderia_phage_BcepMigl_uid184149 | 0.12 |
| Staphylococcus_phage_77_uid14352 | 0.11 |
| Mycobacterium_phage_Bruin_uid230594 | 0.11 |
| Clavibacter_phage_CN1A_uid240037 | 0.11 |
| Acanthocystis_turfacea_Chlorella_virus_1_uid18527 | 0.11 |
| Glossina_pallidipes_salivary_gland_hypertrophy_virus_uid28839 | 0.11 |
| Anguillid_herpesvirus_1_uid42931 | 0.11 |
| Mycobacterium_phage_Contagion_uid215114 | 0.11 |
| Mycobacterium_phage_Dumbo_uid206034 | 0.11 |
| Erinnyis_ello_granulovirus_uid263949 | 0.11 |
| Mycobacterium_phage_Bernal13_uid248633 | 0.11 |
| Staphylococcus_phage_SA11_uid181242 | 0.11 |
| Spodoptera_frugiperda_ascovirus_1a_uid17721 | 0.11 |
| Mycobacterium_phage_Nala_uid230597 | 0.11 |
| Bacillus_phage_phiAGATE_uid185318 | 0.11 |
| Mycobacterium_phage_PhatBacter_uid230595 | 0.11 |
| Mycobacterium_phage_Goku_uid215672 | 0.11 |
| Mycobacterium_phage_Lilac_uid240733 | 0.11 |
| Helicoverpa_SNPV_AC53_uid259191 | 0.11 |
| Mycobacterium_phage_HufflyPuff_uid230582 | 0.11 |
| Coronavirus_SW1_uid29509 | 0.11 |
| Deep_sea_thermophilic_phage_D6E_uid181996 | 0.11 |
| Mycobacterium_phage_Rizal_uid31281 | 0.11 |
| Mycobacterium_phage_Catera_uid17141 | 0.11 |
| Abalone_herpesvirus_Victoria_AUS_2009_uid177933 | 0.11 |
| Helicoverpa_zea_SNPV_uid14148 | 0.11 |
| Mycobacterium_phage_Astraea_uid206480 | 0.11 |
| Mycobacterium_phage_ScottMcG_uid31283 | 0.11 |
| Listeria_phage_LP_030_3_uid253385 | 0.11 |
| Mycobacterium_phage_Spud_uid31285 | 0.11 |
| Mycobacterium_phage_MoMoMixon_uid240773 | 0.11 |
| Mycobacterium_phage_Pleione_uid240719 | 0.11 |
| Mycobacterium_phage_Bxz1_uid14309 | 0.11 |
| Rabbit_fibroma_virus_uid14590 | 0.11 |
| Geobacillus_virus_E2_uid19797 | 0.11 |
| Diadromus_pulchellus_ascovirus_4a_uid32133 | 0.11 |
| Shigella_phage_SP18_uid56019 | 0.11 |
| Aeromonas_phage_65_uid64543 | 0.11 |
| Enterobacteria_phage_JSE_uid38263 | 0.11 |
| Xanthomonas_phage_CP1_uid184158 | 0.11 |
| Cowpox_virus_uid14174 | 0.10 |
| Mycobacterium_phage_BigNuz_uid240721 | 0.10 |
| Streptococcus_phage_P9_uid20785 | 0.10 |
| Mycobacterium_phage_Dandelion_uid240728 | 0.10 |
| Acanthamoeba_polyphaga_mimivirus_uid60053 | 0.10 |
| Mycobacterium_phage_MichelleMyBell_uid240036 | 0.10 |
| Bovine_herpesvirus_1_uid14585 | 0.10 |
| Mycobacterium_phage_ArcherS7_uid206478 | 0.10 |
| Frog_virus_3_uid14560 | 0.10 |
| Mycobacterium_phage_Butters_uid197297 | 0.10 |
| Pandoravirus_salinus_uid215788 | 0.10 |
| Cercopithecine_herpesvirus_5_uid38429 | 0.10 |
| Staphylococcus_phage_85_uid15260 | 0.10 |
| Mycobacterium_phage_Redi_uid240717 | 0.10 |
| Maruca_vitrata_MNPV_uid18533 | 0.10 |
| Murid_herpesvirus_2_uid14419 | 0.10 |
| Choristoneura_fumiferana_MNPV_uid15133 | 0.10 |
| Mycobacterium_phage_Troll4_uid32011 | 0.10 |
| Escherichia_phage_ECML_134_uid266786 | 0.09 |
| Mycobacterium_phage_Brujita_uid32005 | 0.09 |
| Mycobacterium_phage_Fishburne_uid206033 | 0.09 |
| Mycobacterium_phage_Donovan_uid240020 | 0.09 |
| Aeromonas_phage_Aes012_uid195532 | 0.09 |
| Spodoptera_litura_granulovirus_uid19695 | 0.09 |
| Orgyia_leucostigma_NPV_uid28501 | 0.09 |
| T4likevirus_uid266638 | 0.09 |
| Human_herpesvirus_4_uid14413 | 0.09 |
| Mycobacterium_phage_Phrux_uid206029 | 0.09 |
| Pseudomonas_phage_Lu11_uid167656 | 0.09 |
| Mycobacterium_phage_Porky_uid30699 | 0.09 |
| Mycobacterium_phage_244_uid17115 | 0.09 |
| Mycobacterium_phage_Phaux_uid206025 | 0.09 |
| Mycobacterium_phage_Murphy_uid206024 | 0.09 |
| Pandoravirus_dulcis_uid213019 | 0.09 |
| Pectobacterium_phage_phiTE_uid188533 | 0.09 |
| Mycobacterium_phage_Quink_uid219113 | 0.09 |
| Paramecium_bursaria_Chlorella_virus_FR483_uid18305 | 0.09 |
| Mycobacterium_phage_Cjw1_uid14270 | 0.08 |
| Apocheima_cinerarium_nucleopolyhedrovirus_uid173863 | 0.08 |
| Heliothis_zea_virus_1_uid14215 | 0.08 |
| Mycobacterium_phage_Babsiella_uid240766 | 0.08 |
| Mycobacterium_phage_Hawkeye_uid251394 | 0.08 |
| Melanoplus_sanguinipes_entomopoxvirus_uid14042 | 0.08 |
| Mamestra_configurata_NPV_B_uid15128 | 0.08 |
| Alteromonas_phage_vB_AmaP_AD45_P1_uid209073 | 0.08 |
| Culex_nigripalpus_NPV_uid14128 | 0.08 |
| Mycobacterium_phage_Che9c_uid14271 | 0.07 |
| Lumpy_skin_disease_virus_NI_2490_uid14122 | 0.07 |
| Enterococcus_phage_phiEF24C_uid21009 | 0.07 |
| Porcine_cytomegalovirus_uid217990 | 0.07 |
| Mycobacterium_phage_Gizmo_uid206479 | 0.07 |
| Peridroma_alphabaculovirus_uid257515 | 0.07 |
| Xestia_c_nigrum_granulovirus_uid14092 | 0.07 |
| Synechococcus_phage_S_RSM4_uid39923 | 0.07 |
| Human_herpesvirus_3_uid15198 | 0.07 |
| Helicoverpa_armigera_nucleopolyhedrovirus_G4_uid14108 | 0.07 |
| Saimiriine_herpesvirus_2_uid14417 | 0.06 |
| Vibrio_phage_SHOU24_uid240043 | 0.06 |
| Leucania_separata_nuclear_polyhedrosis_virus_uid17669 | 0.06 |
| Mycobacterium_phage_Barnyard_uid14274 | 0.06 |
| Enterobacteria_phage_JS10_uid38265 | 0.06 |
| Enterobacteria_phage_IME08_uid50177 | 0.06 |
| Vibrio_phage_11895_B1_uid195495 | 0.06 |
| Enterobacteria_phage_JS98_uid27983 | 0.06 |
| Ectromelia_virus_uid14211 | 0.06 |
| Bombyx_mandarina_nucleopolyhedrovirus_uid37971 | 0.06 |
| Choristoneura_fumiferana_DEF_MNPV_uid15137 | 0.06 |
| Mycobacterium_phage_BarrelRoll_uid240718 | 0.06 |
| Mycobacterium_phage_CrimD_uid51669 | 0.06 |
| Cellulophaga_phage_phi4_1_uid212952 | 0.06 |
| Mamestra_configurata_NPV_A_uid14168 | 0.06 |
| Cellulophaga_phage_phi17_2_uid212965 | 0.06 |
| Camelpox_virus_uid14156 | 0.06 |
| Escherichia_phage_vB_EcoM_PhAPEC2_uid260484 | 0.06 |
| Synechococcus_phage_S_PM2_uid15223 | 0.05 |
| Megavirus_chiliensis_uid74349 | 0.05 |
| Ecotropis_obliqua_NPV_uid18273 | 0.05 |
| Sheeppox_virus_uid14196 | 0.05 |
| Corynebacterium_phage_P1201_uid20781 | 0.05 |
| Rhodothermus_phage_RM378_uid14420 | 0.05 |
| Aotine_herpesvirus_1_uid78945 | 0.05 |
| Mycobacterium_phage_Cali_uid31291 | 0.05 |
| Pigeonpox_virus_uid253476 | 0.05 |
| Mycobacterium_phage_Nappy_uid240775 | 0.05 |
| Chimpanzee_alpha_1_herpesvirus_uid240320 | 0.05 |
| Invertebrate_iridescent_virus_6_uid14124 | 0.05 |
| Ostreid_herpesvirus_1_uid14552 | 0.05 |
| Felid_herpesvirus_1_uid42429 | 0.05 |
| Cyanophage_PSS2_uid39613 | 0.05 |
| Cercopithecine_herpesvirus_9_uid14596 | 0.05 |
| Bacillus_phage_Hakuna_uid249092 | 0.05 |
| Bathycoccus_RCC1105_virus_BpV1_uid61009 | 0.05 |
| Cafeteria_roenbergensis_virus_BV_PW1_uid59783 | 0.05 |
| Viruses_uid266711 | 0.05 |
| Chrysodeixis_chalcites_nucleopolyhedrovirus_uid15469 | 0.05 |
| Bacillus_phage_Grass_uid227126 | 0.05 |
| Escherichia_phage_e11_2_uid248462 | 0.05 |
| Lactobacillus_phage_LP65_uid14547 | 0.05 |
| Shigella_phage_Shfl2_uid66347 | 0.05 |
| Erwinia_phage_phiEaH2_uid184155 | 0.05 |
| Megavirus_lba_uid188728 | 0.05 |
| Swinepox_virus_uid14155 | 0.05 |
| Enterobacteria_phage_CC31_uid60119 | 0.05 |
| Mycobacterium_phage_ET08_uid42783 | 0.05 |
| Ostreococcus_tauri_virus_1_uid40907 | 0.04 |
| Micromonas_RCC1109_virus_MpV1_uid61013 | 0.04 |
| Variola_virus_uid15197 | 0.04 |
| Synechococcus_phage_S_IOM18_uid209067 | 0.04 |
| Deerpox_virus_W_848_83_uid15462 | 0.04 |
| Clanis_bilineata_nucleopolyhedrosis_virus_uid17485 | 0.04 |
| Canarypox_virus_uid14340 | 0.04 |
| Synechococcus_phage_S_CAM1_uid195484 | 0.04 |
| Pseudomonas_phage_phiKZ_uid14251 | 0.04 |
| Paramecium_bursaria_Chlorella_virus_AR158_uid20991 | 0.04 |
| Enterobacter_phage_PG7_uid240014 | 0.04 |
| Pseudomonas_phage_PaBG_uid215670 | 0.04 |
| Lymantria_dispar_MNPV_uid14390 | 0.04 |
| Spodoptera_frugiperda_MNPV_uid18827 | 0.04 |
| Aeromonas_phage_phiAS4_uid59727 | 0.04 |
| Yaba_monkey_tumor_virus_uid14466 | 0.04 |
| Yoka_poxvirus_uid72715 | 0.04 |
| Murid_herpesvirus_1_uid15181 | 0.04 |
| Vibrio_phage_VH7D_uid239728 | 0.04 |
| Bacillus_phage_SP10_uid181082 | 0.04 |
| Aeromonas_phage_phiAS5_uid59729 | 0.04 |
| Musca_domestica_salivary_gland_hypertrophy_virus_uid29631 | 0.03 |
| Salmonella_phage_Vi01_uid64767 | 0.03 |
| Salmonella_phage_PhiSH19_uid181236 | 0.03 |
| Antheraea_pernyi_nucleopolyhedrovirus_uid16793 | 0.03 |
| Bombyx_mori_NPV_uid14089 | 0.03 |
| Saimiriine_herpesvirus_4_uid78947 | 0.03 |
| Choristoneura_occidentalis_alphabaculovirus_uid214177 | 0.03 |
| Pseudaletia_unipuncta_granulovirus_uid43731 | 0.03 |
| Ralstonia_phage_RSL1_uid30059 | 0.03 |
| Enterobacteria_phage_phi92_uid240593 | 0.03 |
| Anomala_cuprea_entomopoxvirus_uid237470 | 0.03 |
| Murid_herpesvirus_8_uid182227 | 0.03 |
| Dickeya_phage_vB_DsoM_LIMEstone1_uid185317 | 0.03 |
| Aeromonas_phage_Aeh1_uid14312 | 0.03 |
| Lambdina_fiscellaria_nucleopolyhedrovirus_uid282403 | 0.03 |
| Penguinpox_virus_uid253477 | 0.03 |
| Erwinia_phage_Ea35_70_uid240017 | 0.03 |
| Ostreococcus_virus_OsV5_uid28159 | 0.03 |
| Moumouvirus_uid186430 | 0.03 |
| Cotia_virus_SPAn232_uid85563 | 0.03 |
| Invertebrate_iridovirus_22_uid213479 | 0.02 |
| Bacillus_phage_BigBertha_uid227118 | 0.02 |
| Cronobacter_phage_CR8_uid253325 | 0.02 |
| Cronobacter_phage_CR3_uid167658 | 0.02 |
| Macacine_herpesvirus_4_uid14467 | 0.02 |
| Fowlpox_virus_uid14052 | 0.02 |
| Psittacid_herpesvirus_1_uid14314 | 0.02 |
| Ranid_herpesvirus_2_uid17183 | 0.02 |
| Vibrio_phage_KVP40_uid14416 | 0.02 |
| Synechococcus_phage_S_SSM7_uid64711 | 0.01 |
| Yersinia_phage_phiR1_37_uid76739 | 0.01 |
| Vibrio_phage_nt_1_uid209064 | 0.01 |
| Bacillus_phage_G_uid240716 | 0.01 |
