# Supplementary material for: Reconstruction of Bacterial and Viral Genomes from Multiple Metagenomes
Source: Front Microbiol. 2016 Apr 12;7:469. doi: 10.3389/fmicb.2016.00469 (PMC4828583; doi:10.3389/fmicb.2016.00469)
Supplement: Supplementary file 10 [file Table10.DOCX]

**Table S10: Statistics of the assembly performed for the selected six genera.**

|  | **Genus** | | | | |
| --- | --- | --- | --- | --- | --- |
| **Assembly Statistics** | **Akkermansia** | **Bifidobacterium** | **Escheria** | **Odoribacter** | **Parabacteroides** |
| Number of contigs | 1516 | 2196 | 2125 | 1838 | 4269 |
| Total size of contigs | 3118714 | 5163356 | 5663542 | 4850253 | 6439778 |
| Longest contig | 12484 | 31512 | 23302 | 24537 | 16287 |
| Shortest contig | 501 | 501 | 501 | 501 | 501 |
| Number of contigs > 1K nt | 1029 | 1404 | 1585 | 1123 | 2349 |
| Percentage of contigs > 1K nt | 67.9 | 63.9 | 74.6 | 61.1 | 55 |
| Number of contigs > 10K nt | 9 | 43 | 52 | 71 | 11 |
| Percentage of contigs > 10K nt | 0.6 | 2 | 2.4 | 3.9 | 0.3 |
| Mean contig size | 2057 | 2351 | 2665 | 2639 | 1508 |
| Median contig size | 1457 | 1400 | 1711 | 1334 | 1098 |
| N50 contig length | 2886 | 3824 | 4108 | 5063 | 1901 |
| contig %A | 21.86 | 20.1 | 24.97 | 28.15 | 27.36 |
| contig %C | 28.08 | 29.87 | 25.04 | 21.57 | 22.76 |
| contig %G | 28.06 | 29.85 | 25.02 | 21.91 | 22.65 |
| contig %T | 22 | 20.17 | 24.97 | 28.37 | 27.23 |
| contig %N | 0 | 0 | 0 | 0 | 0 |
| contig %non-ACGTN | 0 | 0 | 0 | 0 | 0 |
