# Supplementary material for: Reconstruction of Bacterial and Viral Genomes from Multiple Metagenomes
Source: Front Microbiol. 2016 Apr 12;7:469. doi: 10.3389/fmicb.2016.00469 (PMC4828583; doi:10.3389/fmicb.2016.00469)
Supplement: Supplementary file 11 [file Table11.docx]

**Table S11. Comparison of percentage of assembly achieved by aligning reads and contigs of five selected bacterial genomes with their respective reference genomes.**

| **Genus** | **Genomes** | **% Assembly using Contigs** | **% Assembly using Reads** |
| --- | --- | --- | --- |
| *Akkermansia* | *Akkermansia muciniphila* ATCC BAA 835 | 96.91 | 98.71 |
| *Bifidobacterium* | *Bifidobacterium longum* JCM 1217 | 89.44 | 97.14 |
| *Escherichia* | *Escherichia coli* K 12 substr MDS42 | 88.54 | 95.72 |
| *Odoribacter* | *Odoribacter splanchnicus* DSM_20712 | 96.12 | 99.17 |
| *Parabacteroides* | *Parabacteroides distasonis ATCC 8503* | 93.48 | *98.69* |
