# Supplementary material for: Reconstruction of Bacterial and Viral Genomes from Multiple Metagenomes
Source: Front Microbiol. 2016 Apr 12;7:469. doi: 10.3389/fmicb.2016.00469 (PMC4828583; doi:10.3389/fmicb.2016.00469)
Supplement: Supplementary file 12 [file Table12.DOCX]

**Table S12. Comparison of percentage of assembly achieved by aligning reads and contigs for viral genomes with their respective reference genomes.**

| **Viral Genome** | **% Assembly using Reads** | **% Assembly using Contigs** |
| --- | --- | --- |
| *Lactococcus phage* P008 | 97.86 | 94.52 |
| *Enterobacteria phage* EK99P 1 | 77.74 | 42.03 |
| *Sodalis phage* SO 1 | 75.38 | 40.24 |
| *Shigella phage* EP23 | 69.63 | 38.71 |
| *Bacteroides phage* B40 8 | 49.56 | 10.52 |
