# Supplementary material for: Reconstruction of Bacterial and Viral Genomes from Multiple Metagenomes
Source: Front Microbiol. 2016 Apr 12;7:469. doi: 10.3389/fmicb.2016.00469 (PMC4828583; doi:10.3389/fmicb.2016.00469)
Supplement: Supplementary file 13 [file Table13.DOCX]

**Table S13. Alignment results for the 570 sites (N's) in the simulated draft genome, where gaps (N's) were inserted, with the known bases from the complete reference genome sequence.**

| **Query Name** | **Subject Name** | **Percent Identities** | **Aligned Length** | **# Mismatched pos.** | **# Gap Pos.** | **Query Seq. Start** | **Query Seq. End** | **Subject Seq. Start** | **Subject Seq. End** | **E-Value** | **Bit Score** |
| --- | --- | --- | --- | --- | --- | --- | --- | --- | --- | --- | --- |
| Assembled_7000-7700 | Reference_7000-7700 | 96 | 700 | 28 | 0 | 1 | 700 | 1 | 700 | 0 | 1204 |
| Assembled_14700-15400 | Reference_14700-15400 | 99.57 | 700 | 3 | 0 | 1 | 700 | 1 | 700 | 0 | 1364 |
| Assembled_22400-23100 | Reference_22400-23100 | 99.71 | 700 | 2 | 0 | 1 | 700 | 1 | 700 | 0 | 1382 |
| Assembled_30100-30800 | Reference_30100-30800 | 100 | 700 | 0 | 0 | 1 | 700 | 1 | 700 | 0 | 1388 |
| Assembled_37800-38500 | Reference_37800-38500 | 99.57 | 700 | 3 | 0 | 1 | 700 | 1 | 700 | 0 | 1380 |
| Assembled_45500-46200 | Reference_45500-46200 | 99.57 | 700 | 3 | 0 | 1 | 700 | 1 | 700 | 0 | 1364 |
| Assembled_53200-53900 | Reference_53200-53900 | 100 | 700 | 0 | 0 | 1 | 700 | 1 | 700 | 0 | 1298 |
| Assembled_60900-61600 | Reference_60900-61600 | 98.29 | 700 | 12 | 0 | 1 | 700 | 1 | 700 | 0 | 1308 |
| Assembled_68600-69300 | Reference_68600-69300 | 100 | 700 | 0 | 0 | 1 | 700 | 1 | 700 | 0 | 1388 |
| Assembled_76300-77000 | Reference_76300-77000 | 99.29 | 700 | 5 | 0 | 1 | 700 | 1 | 700 | 0 | 1348 |
| Assembled_84000-84700 | Reference_84000-84700 | 99.14 | 700 | 6 | 0 | 1 | 700 | 1 | 700 | 0 | 1229 |
| Assembled_91700-92400 | Reference_91700-92400 | 97.57 | 700 | 17 | 0 | 1 | 700 | 1 | 700 | 0 | 1144 |
| Assembled_99400-100100 | Reference_99400-100100 | 99.43 | 700 | 4 | 0 | 1 | 700 | 1 | 700 | 0 | 1372 |
| Assembled_107100-107800 | Reference_107100-107800 | 100 | 673 | 0 | 0 | 28 | 700 | 28 | 700 | 0 | 1292 |
| Assembled_114800-115500 | Reference_114800-115500 | 100 | 700 | 0 | 0 | 1 | 700 | 1 | 700 | 0 | 1388 |
| Assembled_122500-123200 | Reference_122500-123200 | 100 | 254 | 0 | 0 | 447 | 700 | 447 | 700 | 5e-144 | 504 |
| Assembled_130200-130900 | Reference_130200-130900 | 99.43 | 700 | 4 | 0 | 1 | 700 | 1 | 700 | 0 | 1356 |
| Assembled_137900-138600 | Reference_137900-138600 | 95.57 | 700 | 31 | 0 | 1 | 700 | 1 | 700 | 0 | 1266 |
| Assembled_145600-146300 | Reference_145600-146300 | 99.86 | 696 | 1 | 0 | 1 | 696 | 1 | 696 | 0 | 1241 |
| Assembled_153300-154000 | Reference_153300-154000 | 98.14 | 700 | 13 | 0 | 1 | 700 | 1 | 700 | 0 | 1337 |
| Assembled_161000-161700 | Reference_161000-161700 | 99.71 | 700 | 2 | 0 | 1 | 700 | 1 | 700 | 0 | 1372 |
| Assembled_168700-169400 | Reference_168700-169400 | 99.14 | 700 | 6 | 0 | 1 | 700 | 1 | 700 | 0 | 1309 |
| Assembled_176400-177100 | Reference_176400-177100 | 100 | 700 | 0 | 0 | 1 | 700 | 1 | 700 | 0 | 1340 |
| Assembled_184100-184800 | Reference_184100-184800 | 99.86 | 700 | 1 | 0 | 1 | 700 | 1 | 700 | 0 | 1380 |
| Assembled_191800-192500 | Reference_191800-192500 | 99 | 700 | 7 | 0 | 1 | 700 | 1 | 700 | 0 | 1369 |
| Assembled_199500-200200 | Reference_199500-200200 | 99.86 | 700 | 1 | 0 | 1 | 700 | 1 | 700 | 0 | 1385 |
| Assembled_207200-207900 | Reference_207200-207900 | 99.14 | 700 | 6 | 0 | 1 | 700 | 1 | 700 | 0 | 1366 |
| Assembled_214900-215600 | Reference_214900-215600 | 99.86 | 700 | 1 | 0 | 1 | 700 | 1 | 700 | 0 | 1338 |
| Assembled_222600-223300 | Reference_222600-223300 | 100 | 700 | 0 | 0 | 1 | 700 | 1 | 700 | 0 | 1388 |
| Assembled_230300-231000 | Reference_230300-231000 | 99.29 | 700 | 5 | 0 | 1 | 700 | 1 | 700 | 0 | 1374 |
| Assembled_238000-238700 | Reference_238000-238700 | 96.55 | 695 | 24 | 0 | 1 | 695 | 1 | 695 | 0 | 1209 |
| Assembled_245700-246400 | Reference_245700-246400 | 99.43 | 700 | 4 | 0 | 1 | 700 | 1 | 700 | 0 | 1366 |
| Assembled_253400-254100 | Reference_253400-254100 | 99 | 700 | 7 | 0 | 1 | 700 | 1 | 700 | 0 | 1332 |
| Assembled_261100-261800 | Reference_261100-261800 | 99.86 | 700 | 1 | 0 | 1 | 700 | 1 | 700 | 0 | 1380 |
| Assembled_268800-269500 | Reference_268800-269500 | 97.43 | 700 | 18 | 0 | 1 | 700 | 1 | 700 | 0 | 1328 |
| Assembled_276500-277200 | Reference_276500-277200 | 99.86 | 700 | 1 | 0 | 1 | 700 | 1 | 700 | 0 | 1338 |
| Assembled_284200-284900 | Reference_284200-284900 | 96.57 | 700 | 24 | 0 | 1 | 700 | 1 | 700 | 0 | 1249 |
| Assembled_291900-292600 | Reference_291900-292600 | 99.57 | 700 | 3 | 0 | 1 | 700 | 1 | 700 | 0 | 1322 |
| Assembled_299600-300300 | Reference_299600-300300 | 99.46 | 371 | 2 | 0 | 1 | 371 | 1 | 371 | 0 | 684 |
| Assembled_307300-308000 | Reference_307300-308000 | 97.71 | 700 | 16 | 0 | 1 | 700 | 1 | 700 | 0 | 1345 |
| Assembled_315000-315700 | Reference_315000-315700 | 100 | 700 | 0 | 0 | 1 | 700 | 1 | 700 | 0 | 1304 |
| Assembled_322700-323400 | Reference_322700-323400 | 99.71 | 700 | 2 | 0 | 1 | 700 | 1 | 700 | 0 | 1372 |
| Assembled_330400-331100 | Reference_330400-331100 | 99.71 | 700 | 2 | 0 | 1 | 700 | 1 | 700 | 0 | 1372 |
| Assembled_338100-338800 | Reference_338100-338800 | 99.29 | 700 | 5 | 0 | 1 | 700 | 1 | 700 | 0 | 1348 |
| Assembled_345800-346500 | Reference_345800-346500 | 99.57 | 700 | 3 | 0 | 1 | 700 | 1 | 700 | 0 | 1374 |
| Assembled_353500-354200 | Reference_353500-354200 | 99 | 700 | 7 | 0 | 1 | 700 | 1 | 700 | 0 | 1290 |
| Assembled_361200-361900 | Reference_361200-361900 | 96.86 | 700 | 22 | 0 | 1 | 700 | 1 | 700 | 0 | 1300 |
| Assembled_368900-369600 | Reference_368900-369600 | 99.86 | 700 | 1 | 0 | 1 | 700 | 1 | 700 | 0 | 1332 |
| Assembled_376600-377300 | Reference_376600-377300 | 99.86 | 700 | 1 | 0 | 1 | 700 | 1 | 700 | 0 | 1380 |
| Assembled_384300-385000 | Reference_384300-385000 | 99.71 | 700 | 2 | 0 | 1 | 700 | 1 | 700 | 0 | 1330 |
| Assembled_392000-392700 | Reference_392000-392700 | 100 | 307 | 0 | 0 | 394 | 700 | 394 | 700 | 2e-176 | 611 |
| Assembled_399700-400400 | Reference_399700-400400 | 98.57 | 700 | 10 | 0 | 1 | 700 | 1 | 700 | 0 | 1314 |
| Assembled_407400-408100 | Reference_407400-408100 | 100 | 170 | 0 | 0 | 1 | 170 | 1 | 170 | 7e-94 | 337 |
| Assembled_415100-415800 | Reference_415100-415800 | 99.43 | 700 | 4 | 0 | 1 | 700 | 1 | 700 | 0 | 1366 |
| Assembled_422800-423500 | Reference_422800-423500 | 98.45 | 386 | 6 | 0 | 1 | 386 | 1 | 386 | 0 | 718 |
| Assembled_430500-431200 | Reference_430500-431200 | 100 | 700 | 0 | 0 | 1 | 700 | 1 | 700 | 0 | 1388 |
| Assembled_438200-438900 | Reference_438200-438900 | 97.29 | 700 | 19 | 0 | 1 | 700 | 1 | 700 | 0 | 1237 |
| Assembled_445900-446600 | Reference_445900-446600 | 100 | 700 | 0 | 0 | 1 | 700 | 1 | 700 | 0 | 1388 |
| Assembled_453600-454300 | Reference_453600-454300 | 100 | 700 | 0 | 0 | 1 | 700 | 1 | 700 | 0 | 1388 |
| Assembled_461300-462000 | Reference_461300-462000 | 99.86 | 700 | 1 | 0 | 1 | 700 | 1 | 700 | 0 | 1338 |
| Assembled_469000-469700 | Reference_469000-469700 | 99 | 700 | 7 | 0 | 1 | 700 | 1 | 700 | 0 | 1285 |
| Assembled_476700-477400 | Reference_476700-477400 | 99.76 | 410 | 1 | 0 | 1 | 410 | 1 | 410 | 0 | 722 |
| Assembled_484400-485100 | Reference_484400-485100 | 100 | 700 | 0 | 0 | 1 | 700 | 1 | 700 | 0 | 1388 |
| Assembled_492100-492800 | Reference_492100-492800 | 98.71 | 700 | 9 | 0 | 1 | 700 | 1 | 700 | 0 | 1316 |
| Assembled_499800-500500 | Reference_499800-500500 | 99.71 | 700 | 2 | 0 | 1 | 700 | 1 | 700 | 0 | 1377 |
| Assembled_507500-508200 | Reference_507500-508200 | 96.14 | 648 | 25 | 0 | 1 | 648 | 1 | 648 | 0 | 1167 |
| Assembled_515200-515900 | Reference_515200-515900 | 99.14 | 700 | 6 | 0 | 1 | 700 | 1 | 700 | 0 | 1361 |
| Assembled_522900-523600 | Reference_522900-523600 | 88.38 | 697 | 81 | 0 | 1 | 697 | 1 | 697 | 0 | 1099 |
| Assembled_530600-531300 | Reference_530600-531300 | 99.86 | 700 | 1 | 0 | 1 | 700 | 1 | 700 | 0 | 1296 |
| Assembled_538300-539000 | Reference_538300-539000 | 100 | 567 | 0 | 0 | 1 | 567 | 1 | 567 | 0 | 1124 |
| Assembled_546000-546700 | Reference_546000-546700 | 99.86 | 700 | 1 | 0 | 1 | 700 | 1 | 700 | 0 | 1255 |
| Assembled_553700-554400 | Reference_553700-554400 | 98.71 | 700 | 9 | 0 | 1 | 700 | 1 | 700 | 0 | 1364 |
| Assembled_561400-562100 | Reference_561400-562100 | 99.43 | 700 | 4 | 0 | 1 | 700 | 1 | 700 | 0 | 1356 |
| Assembled_569100-569800 | Reference_569100-569800 | 99.43 | 700 | 4 | 0 | 1 | 700 | 1 | 700 | 0 | 1356 |
| Assembled_576800-577500 | Reference_576800-577500 | 99.14 | 700 | 6 | 0 | 1 | 700 | 1 | 700 | 0 | 1345 |
| Assembled_584500-585200 | Reference_584500-585200 | 98.43 | 700 | 11 | 0 | 1 | 700 | 1 | 700 | 0 | 1320 |
| Assembled_592200-592900 | Reference_592200-592900 | 95.54 | 471 | 21 | 0 | 167 | 637 | 167 | 637 | 0 | 745 |
| Assembled_599900-600600 | Reference_599900-600600 | 95 | 700 | 35 | 0 | 1 | 700 | 1 | 700 | 0 | 1238 |
| Assembled_607600-608300 | Reference_607600-608300 | 99.46 | 370 | 2 | 0 | 331 | 700 | 331 | 700 | 0 | 718 |
| Assembled_615300-616000 | Reference_615300-616000 | 98.43 | 700 | 11 | 0 | 1 | 700 | 1 | 700 | 0 | 1334 |
| Assembled_623000-623700 | Reference_623000-623700 | 95.57 | 700 | 31 | 0 | 1 | 700 | 1 | 700 | 0 | 1298 |
| Assembled_630700-631400 | Reference_630700-631400 | 97.56 | 698 | 17 | 0 | 3 | 700 | 3 | 700 | 0 | 1332 |
| Assembled_638400-639100 | Reference_638400-639100 | 99.86 | 700 | 1 | 0 | 1 | 700 | 1 | 700 | 0 | 1380 |
| Assembled_646100-646800 | Reference_646100-646800 | 99.86 | 700 | 1 | 0 | 1 | 700 | 1 | 700 | 0 | 1385 |
| Assembled_653800-654500 | Reference_653800-654500 | 98.42 | 698 | 11 | 0 | 3 | 700 | 3 | 700 | 0 | 1166 |
| Assembled_661500-662200 | Reference_661500-662200 | 100 | 700 | 0 | 0 | 1 | 700 | 1 | 700 | 0 | 1346 |
| Assembled_669200-669900 | Reference_669200-669900 | 99.57 | 700 | 3 | 0 | 1 | 700 | 1 | 700 | 0 | 1364 |
| Assembled_676900-677600 | Reference_676900-677600 | 99.86 | 700 | 1 | 0 | 1 | 700 | 1 | 700 | 0 | 1380 |
| Assembled_684600-685300 | Reference_684600-685300 | 98.21 | 560 | 10 | 0 | 141 | 700 | 141 | 700 | 0 | 1082 |
| Assembled_692300-693000 | Reference_692300-693000 | 100 | 700 | 0 | 0 | 1 | 700 | 1 | 700 | 0 | 1388 |
| Assembled_700000-700700 | Reference_700000-700700 | 100 | 492 | 0 | 0 | 1 | 492 | 1 | 492 | 0 | 934 |
| Assembled_707700-708400 | Reference_707700-708400 | 100 | 700 | 0 | 0 | 1 | 700 | 1 | 700 | 0 | 1388 |
| Assembled_715400-716100 | Reference_715400-716100 | 100 | 607 | 0 | 0 | 16 | 622 | 16 | 622 | 0 | 1203 |
| Assembled_723100-723800 | Reference_723100-723800 | 94 | 700 | 42 | 0 | 1 | 700 | 1 | 700 | 0 | 1055 |
| Assembled_730800-731500 | Reference_730800-731500 | 100 | 700 | 0 | 0 | 1 | 700 | 1 | 700 | 0 | 1388 |
| Assembled_738500-739200 | Reference_738500-739200 | 100 | 700 | 0 | 0 | 1 | 700 | 1 | 700 | 0 | 1388 |
| Assembled_746200-746900 | Reference_746200-746900 | 99.86 | 700 | 1 | 0 | 1 | 700 | 1 | 700 | 0 | 1380 |
| Assembled_753900-754600 | Reference_753900-754600 | 99.71 | 700 | 2 | 0 | 1 | 700 | 1 | 700 | 0 | 1382 |
| Assembled_761600-762300 | Reference_761600-762300 | 100 | 194 | 0 | 0 | 507 | 700 | 507 | 700 | 3e-108 | 385 |
| Assembled_769300-770000 | Reference_769300-770000 | 100 | 531 | 0 | 0 | 170 | 700 | 170 | 700 | 0 | 1053 |
| Assembled_777000-777700 | Reference_777000-777700 | 98 | 700 | 14 | 0 | 1 | 700 | 1 | 700 | 0 | 1258 |
| Assembled_784700-785400 | Reference_784700-785400 | 99.86 | 700 | 1 | 0 | 1 | 700 | 1 | 700 | 0 | 1380 |
| Assembled_792400-793100 | Reference_792400-793100 | 99.14 | 700 | 6 | 0 | 1 | 700 | 1 | 700 | 0 | 1340 |
| Assembled_800100-800800 | Reference_800100-800800 | 99.86 | 700 | 1 | 0 | 1 | 700 | 1 | 700 | 0 | 1385 |
| Assembled_807800-808500 | Reference_807800-808500 | 98.57 | 700 | 10 | 0 | 1 | 700 | 1 | 700 | 0 | 1308 |
| Assembled_815500-816200 | Reference_815500-816200 | 100 | 700 | 0 | 0 | 1 | 700 | 1 | 700 | 0 | 1388 |
| Assembled_823200-823900 | Reference_823200-823900 | 99.71 | 700 | 2 | 0 | 1 | 700 | 1 | 700 | 0 | 1372 |
| Assembled_830900-831600 | Reference_830900-831600 | 99.57 | 700 | 3 | 0 | 1 | 700 | 1 | 700 | 0 | 1364 |
| Assembled_838600-839300 | Reference_838600-839300 | 100 | 700 | 0 | 0 | 1 | 700 | 1 | 700 | 0 | 1388 |
| Assembled_846300-847000 | Reference_846300-847000 | 99.86 | 692 | 1 | 0 | 9 | 700 | 9 | 700 | 0 | 1287 |
| Assembled_854000-854700 | Reference_854000-854700 | 99.79 | 486 | 1 | 0 | 215 | 700 | 215 | 700 | 0 | 955 |
| Assembled_861700-862400 | Reference_861700-862400 | 99.29 | 700 | 5 | 0 | 1 | 700 | 1 | 700 | 0 | 1316 |
| Assembled_869400-870100 | Reference_869400-870100 | 99.86 | 700 | 1 | 0 | 1 | 700 | 1 | 700 | 0 | 1338 |
| Assembled_877100-877800 | Reference_877100-877800 | 98.43 | 700 | 11 | 0 | 1 | 700 | 1 | 700 | 0 | 1300 |
| Assembled_884800-885500 | Reference_884800-885500 | 99.29 | 700 | 5 | 0 | 1 | 700 | 1 | 700 | 0 | 1306 |
| Assembled_892500-893200 | Reference_892500-893200 | 99.57 | 700 | 3 | 0 | 1 | 700 | 1 | 700 | 0 | 1380 |
| Assembled_900200-900900 | Reference_900200-900900 | 99.86 | 700 | 1 | 0 | 1 | 700 | 1 | 700 | 0 | 1380 |
| Assembled_907900-908600 | Reference_907900-908600 | 99.86 | 700 | 1 | 0 | 1 | 700 | 1 | 700 | 0 | 1380 |
| Assembled_915600-916300 | Reference_915600-916300 | 96.86 | 700 | 22 | 0 | 1 | 700 | 1 | 700 | 0 | 1232 |
| Assembled_923300-924000 | Reference_923300-924000 | 98.14 | 700 | 13 | 0 | 1 | 700 | 1 | 700 | 0 | 1201 |
| Assembled_931000-931700 | Reference_931000-931700 | 100 | 700 | 0 | 0 | 1 | 700 | 1 | 700 | 0 | 1388 |
| Assembled_938700-939400 | Reference_938700-939400 | 93.84 | 698 | 43 | 0 | 1 | 698 | 1 | 698 | 0 | 1216 |
| Assembled_946400-947100 | Reference_946400-947100 | 98.86 | 700 | 8 | 0 | 1 | 700 | 1 | 700 | 0 | 1366 |
| Assembled_954100-954800 | Reference_954100-954800 | 96.57 | 700 | 24 | 0 | 1 | 700 | 1 | 700 | 0 | 1319 |
| Assembled_961800-962500 | Reference_961800-962500 | 99.71 | 700 | 2 | 0 | 1 | 700 | 1 | 700 | 0 | 1377 |
| Assembled_969500-970200 | Reference_969500-970200 | 98 | 700 | 14 | 0 | 1 | 700 | 1 | 700 | 0 | 1297 |
| Assembled_977200-977900 | Reference_977200-977900 | 99.71 | 700 | 2 | 0 | 1 | 700 | 1 | 700 | 0 | 1294 |
| Assembled_984900-985600 | Reference_984900-985600 | 99.14 | 700 | 6 | 0 | 1 | 700 | 1 | 700 | 0 | 1342 |
| Assembled_992600-993300 | Reference_992600-993300 | 99.86 | 700 | 1 | 0 | 1 | 700 | 1 | 700 | 0 | 1338 |
| Assembled_1000300-1001000 | Reference_1000300-1001000 | 99.71 | 700 | 2 | 0 | 1 | 700 | 1 | 700 | 0 | 1372 |
| Assembled_1008000-1008700 | Reference_1008000-1008700 | 100 | 700 | 0 | 0 | 1 | 700 | 1 | 700 | 0 | 1388 |
| Assembled_1015700-1016400 | Reference_1015700-1016400 | 99.85 | 660 | 1 | 0 | 1 | 660 | 1 | 660 | 0 | 1300 |
| Assembled_1023400-1024100 | Reference_1023400-1024100 | 99.71 | 700 | 2 | 0 | 1 | 700 | 1 | 700 | 0 | 1338 |
| Assembled_1031100-1031800 | Reference_1031100-1031800 | 99.86 | 700 | 1 | 0 | 1 | 700 | 1 | 700 | 0 | 1380 |
| Assembled_1038800-1039500 | Reference_1038800-1039500 | 100 | 700 | 0 | 0 | 1 | 700 | 1 | 700 | 0 | 1388 |
| Assembled_1046500-1047200 | Reference_1046500-1047200 | 99.43 | 700 | 4 | 0 | 1 | 700 | 1 | 700 | 0 | 1356 |
| Assembled_1054200-1054900 | Reference_1054200-1054900 | 99.43 | 700 | 4 | 0 | 1 | 700 | 1 | 700 | 0 | 1377 |
| Assembled_1061900-1062600 | Reference_1061900-1062600 | 99.57 | 700 | 3 | 0 | 1 | 700 | 1 | 700 | 0 | 1380 |
| Assembled_1069600-1070300 | Reference_1069600-1070300 | 99.71 | 700 | 2 | 0 | 1 | 700 | 1 | 700 | 0 | 1377 |
| Assembled_1077300-1078000 | Reference_1077300-1078000 | 99.14 | 700 | 6 | 0 | 1 | 700 | 1 | 700 | 0 | 1340 |
| Assembled_1085000-1085700 | Reference_1085000-1085700 | 98.86 | 700 | 8 | 0 | 1 | 700 | 1 | 700 | 0 | 1314 |
| Assembled_1092700-1093400 | Reference_1092700-1093400 | 99.29 | 700 | 5 | 0 | 1 | 700 | 1 | 700 | 0 | 1358 |
| Assembled_1100400-1101100 | Reference_1100400-1101100 | 99.86 | 700 | 1 | 0 | 1 | 700 | 1 | 700 | 0 | 1380 |
| Assembled_1108100-1108800 | Reference_1108100-1108800 | 99.43 | 700 | 4 | 0 | 1 | 700 | 1 | 700 | 0 | 1356 |
| Assembled_1115800-1116500 | Reference_1115800-1116500 | 96.8 | 437 | 14 | 0 | 1 | 437 | 1 | 437 | 0 | 754 |
| Assembled_1123500-1124200 | Reference_1123500-1124200 | 99.43 | 700 | 4 | 0 | 1 | 700 | 1 | 700 | 0 | 1367 |
| Assembled_1131200-1131900 | Reference_1131200-1131900 | 100 | 700 | 0 | 0 | 1 | 700 | 1 | 700 | 0 | 1340 |
| Assembled_1138900-1139600 | Reference_1138900-1139600 | 99 | 700 | 7 | 0 | 1 | 700 | 1 | 700 | 0 | 1332 |
| Assembled_1146600-1147300 | Reference_1146600-1147300 | 99.29 | 700 | 5 | 0 | 1 | 700 | 1 | 700 | 0 | 1306 |
| Assembled_1154300-1155000 | Reference_1154300-1155000 | 97.26 | 219 | 6 | 0 | 1 | 219 | 1 | 219 | 8e-109 | 387 |
| Assembled_1162000-1162700 | Reference_1162000-1162700 | 99.71 | 700 | 2 | 0 | 1 | 700 | 1 | 700 | 0 | 1335 |
| Assembled_1169700-1170400 | Reference_1169700-1170400 | 96.85 | 698 | 22 | 0 | 1 | 698 | 1 | 698 | 0 | 1269 |
| Assembled_1177400-1178100 | Reference_1177400-1178100 | 98.71 | 700 | 9 | 0 | 1 | 700 | 1 | 700 | 0 | 1327 |
| Assembled_1185100-1185800 | Reference_1185100-1185800 | 99.86 | 700 | 1 | 0 | 1 | 700 | 1 | 700 | 0 | 1380 |
| Assembled_1192800-1193500 | Reference_1192800-1193500 | 97.15 | 562 | 16 | 0 | 139 | 700 | 139 | 700 | 0 | 930 |
| Assembled_1200500-1201200 | Reference_1200500-1201200 | 99.71 | 700 | 2 | 0 | 1 | 700 | 1 | 700 | 0 | 1377 |
| Assembled_1208200-1208900 | Reference_1208200-1208900 | 98.43 | 699 | 11 | 0 | 1 | 699 | 1 | 699 | 0 | 1310 |
| Assembled_1215900-1216600 | Reference_1215900-1216600 | 99.29 | 700 | 5 | 0 | 1 | 700 | 1 | 700 | 0 | 1306 |
| Assembled_1223600-1224300 | Reference_1223600-1224300 | 100 | 700 | 0 | 0 | 1 | 700 | 1 | 700 | 0 | 1388 |
| Assembled_1231300-1232000 | Reference_1231300-1232000 | 98.71 | 700 | 9 | 0 | 1 | 700 | 1 | 700 | 0 | 1275 |
| Assembled_1239000-1239700 | Reference_1239000-1239700 | 99.86 | 700 | 1 | 0 | 1 | 700 | 1 | 700 | 0 | 1380 |
| Assembled_1246700-1247400 | Reference_1246700-1247400 | 99.57 | 700 | 3 | 0 | 1 | 700 | 1 | 700 | 0 | 1374 |
| Assembled_1254400-1255100 | Reference_1254400-1255100 | 97.86 | 700 | 15 | 0 | 1 | 700 | 1 | 700 | 0 | 1324 |
| Assembled_1262100-1262800 | Reference_1262100-1262800 | 99.14 | 700 | 6 | 0 | 1 | 700 | 1 | 700 | 0 | 1340 |
| Assembled_1269800-1270500 | Reference_1269800-1270500 | 100 | 700 | 0 | 0 | 1 | 700 | 1 | 700 | 0 | 1388 |
| Assembled_1277500-1278200 | Reference_1277500-1278200 | 99.43 | 700 | 4 | 0 | 1 | 700 | 1 | 700 | 0 | 1377 |
| Assembled_1285200-1285900 | Reference_1285200-1285900 | 100 | 700 | 0 | 0 | 1 | 700 | 1 | 700 | 0 | 1388 |
| Assembled_1292900-1293600 | Reference_1292900-1293600 | 99.86 | 700 | 1 | 0 | 1 | 700 | 1 | 700 | 0 | 1380 |
| Assembled_1300600-1301300 | Reference_1300600-1301300 | 100 | 700 | 0 | 0 | 1 | 700 | 1 | 700 | 0 | 1334 |
| Assembled_1308300-1309000 | Reference_1308300-1309000 | 99.86 | 700 | 1 | 0 | 1 | 700 | 1 | 700 | 0 | 1380 |
| Assembled_1316000-1316700 | Reference_1316000-1316700 | 99.57 | 700 | 3 | 0 | 1 | 700 | 1 | 700 | 0 | 1322 |
| Assembled_1323700-1324400 | Reference_1323700-1324400 | 99.86 | 700 | 1 | 0 | 1 | 700 | 1 | 700 | 0 | 1380 |
| Assembled_1331400-1332100 | Reference_1331400-1332100 | 99.43 | 700 | 4 | 0 | 1 | 700 | 1 | 700 | 0 | 1377 |
| Assembled_1339100-1339800 | Reference_1339100-1339800 | 100 | 700 | 0 | 0 | 1 | 700 | 1 | 700 | 0 | 1388 |
| Assembled_1346800-1347500 | Reference_1346800-1347500 | 99.57 | 700 | 3 | 0 | 1 | 700 | 1 | 700 | 0 | 1374 |
| Assembled_1354500-1355200 | Reference_1354500-1355200 | 100 | 700 | 0 | 0 | 1 | 700 | 1 | 700 | 0 | 1388 |
| Assembled_1362200-1362900 | Reference_1362200-1362900 | 96.29 | 700 | 26 | 0 | 1 | 700 | 1 | 700 | 0 | 1306 |
| Assembled_1369900-1370600 | Reference_1369900-1370600 | 96.71 | 700 | 23 | 0 | 1 | 700 | 1 | 700 | 0 | 1094 |
| Assembled_1377600-1378300 | Reference_1377600-1378300 | 98.71 | 700 | 9 | 0 | 1 | 700 | 1 | 700 | 0 | 1275 |
| Assembled_1385300-1386000 | Reference_1385300-1386000 | 100 | 700 | 0 | 0 | 1 | 700 | 1 | 700 | 0 | 1388 |
| Assembled_1393000-1393700 | Reference_1393000-1393700 | 96.94 | 490 | 15 | 0 | 1 | 490 | 1 | 490 | 0 | 927 |
| Assembled_1400700-1401400 | Reference_1400700-1401400 | 100 | 338 | 0 | 0 | 1 | 338 | 1 | 338 | 0 | 629 |
| Assembled_1408400-1409100 | Reference_1408400-1409100 | 97.86 | 700 | 15 | 0 | 1 | 700 | 1 | 700 | 0 | 1269 |
| Assembled_1416100-1416800 | Reference_1416100-1416800 | 84.71 | 700 | 107 | 0 | 1 | 700 | 1 | 700 | 0 | 977 |
| Assembled_1423800-1424500 | Reference_1423800-1424500 | 93.57 | 653 | 42 | 0 | 48 | 700 | 48 | 700 | 0 | 1062 |
| Assembled_1431500-1432200 | Reference_1431500-1432200 | 99.57 | 691 | 3 | 0 | 10 | 700 | 10 | 700 | 0 | 1346 |
| Assembled_1439200-1439900 | Reference_1439200-1439900 | 99 | 700 | 7 | 0 | 1 | 700 | 1 | 700 | 0 | 1369 |
| Assembled_1446900-1447600 | Reference_1446900-1447600 | 99.86 | 700 | 1 | 0 | 1 | 700 | 1 | 700 | 0 | 1291 |
| Assembled_1454600-1455300 | Reference_1454600-1455300 | 99.71 | 700 | 2 | 0 | 1 | 700 | 1 | 700 | 0 | 1382 |
| Assembled_1462300-1463000 | Reference_1462300-1463000 | 98 | 700 | 14 | 0 | 1 | 700 | 1 | 700 | 0 | 1350 |
| Assembled_1470000-1470700 | Reference_1470000-1470700 | 98.86 | 700 | 8 | 0 | 1 | 700 | 1 | 700 | 0 | 1356 |
| Assembled_1477700-1478400 | Reference_1477700-1478400 | 100 | 700 | 0 | 0 | 1 | 700 | 1 | 700 | 0 | 1388 |
| Assembled_1485400-1486100 | Reference_1485400-1486100 | 100 | 700 | 0 | 0 | 1 | 700 | 1 | 700 | 0 | 1346 |
| Assembled_1493100-1493800 | Reference_1493100-1493800 | 100 | 700 | 0 | 0 | 1 | 700 | 1 | 700 | 0 | 1388 |
| Assembled_1500800-1501500 | Reference_1500800-1501500 | 98.57 | 700 | 10 | 0 | 1 | 700 | 1 | 700 | 0 | 1308 |
| Assembled_1508500-1509200 | Reference_1508500-1509200 | 99.71 | 700 | 2 | 0 | 1 | 700 | 1 | 700 | 0 | 1382 |
| Assembled_1516200-1516900 | Reference_1516200-1516900 | 99.57 | 700 | 3 | 0 | 1 | 700 | 1 | 700 | 0 | 1327 |
| Assembled_1523900-1524600 | Reference_1523900-1524600 | 99.43 | 700 | 4 | 0 | 1 | 700 | 1 | 700 | 0 | 1377 |
| Assembled_1531600-1532300 | Reference_1531600-1532300 | 100 | 700 | 0 | 0 | 1 | 700 | 1 | 700 | 0 | 1388 |
| Assembled_1539300-1540000 | Reference_1539300-1540000 | 100 | 700 | 0 | 0 | 1 | 700 | 1 | 700 | 0 | 1388 |
| Assembled_1547000-1547700 | Reference_1547000-1547700 | 99.71 | 700 | 2 | 0 | 1 | 700 | 1 | 700 | 0 | 1372 |
| Assembled_1554700-1555400 | Reference_1554700-1555400 | 99.43 | 700 | 4 | 0 | 1 | 700 | 1 | 700 | 0 | 1366 |
| Assembled_1562400-1563100 | Reference_1562400-1563100 | 100 | 700 | 0 | 0 | 1 | 700 | 1 | 700 | 0 | 1388 |
| Assembled_1570100-1570800 | Reference_1570100-1570800 | 98.57 | 700 | 10 | 0 | 1 | 700 | 1 | 700 | 0 | 1256 |
| Assembled_1577800-1578500 | Reference_1577800-1578500 | 99.57 | 700 | 3 | 0 | 1 | 700 | 1 | 700 | 0 | 1316 |
| Assembled_1585500-1586200 | Reference_1585500-1586200 | 99.86 | 700 | 1 | 0 | 1 | 700 | 1 | 700 | 0 | 1380 |
| Assembled_1593200-1593900 | Reference_1593200-1593900 | 99.71 | 700 | 2 | 0 | 1 | 700 | 1 | 700 | 0 | 1300 |
| Assembled_1600900-1601600 | Reference_1600900-1601600 | 99.29 | 700 | 5 | 0 | 1 | 700 | 1 | 700 | 0 | 1333 |
| Assembled_1608600-1609300 | Reference_1608600-1609300 | 96.13 | 698 | 27 | 0 | 3 | 700 | 3 | 700 | 0 | 1285 |
| Assembled_1616300-1617000 | Reference_1616300-1617000 | 100 | 700 | 0 | 0 | 1 | 700 | 1 | 700 | 0 | 1251 |
| Assembled_1624000-1624700 | Reference_1624000-1624700 | 82.45 | 695 | 122 | 0 | 2 | 696 | 2 | 696 | 0 | 944 |
| Assembled_1631700-1632400 | Reference_1631700-1632400 | 99.57 | 700 | 3 | 0 | 1 | 700 | 1 | 700 | 0 | 1369 |
| Assembled_1639400-1640100 | Reference_1639400-1640100 | 100 | 700 | 0 | 0 | 1 | 700 | 1 | 700 | 0 | 1346 |
| Assembled_1647100-1647800 | Reference_1647100-1647800 | 100 | 700 | 0 | 0 | 1 | 700 | 1 | 700 | 0 | 1388 |
| Assembled_1654800-1655500 | Reference_1654800-1655500 | 99.29 | 700 | 5 | 0 | 1 | 700 | 1 | 700 | 0 | 1369 |
| Assembled_1662500-1663200 | Reference_1662500-1663200 | 99.71 | 700 | 2 | 0 | 1 | 700 | 1 | 700 | 0 | 1372 |
| Assembled_1670200-1670900 | Reference_1670200-1670900 | 99.86 | 700 | 1 | 0 | 1 | 700 | 1 | 700 | 0 | 1380 |
| Assembled_1677900-1678600 | Reference_1677900-1678600 | 99.49 | 391 | 2 | 0 | 1 | 391 | 1 | 391 | 0 | 770 |
| Assembled_1685600-1686300 | Reference_1685600-1686300 | 99.57 | 700 | 3 | 0 | 1 | 700 | 1 | 700 | 0 | 1364 |
| Assembled_1693300-1694000 | Reference_1693300-1694000 | 99.71 | 700 | 2 | 0 | 1 | 700 | 1 | 700 | 0 | 1330 |
| Assembled_1701000-1701700 | Reference_1701000-1701700 | 100 | 700 | 0 | 0 | 1 | 700 | 1 | 700 | 0 | 1346 |
| Assembled_1708700-1709400 | Reference_1708700-1709400 | 100 | 700 | 0 | 0 | 1 | 700 | 1 | 700 | 0 | 1346 |
| Assembled_1716400-1717100 | Reference_1716400-1717100 | 99.71 | 700 | 2 | 0 | 1 | 700 | 1 | 700 | 0 | 1372 |
| Assembled_1724100-1724800 | Reference_1724100-1724800 | 99.86 | 700 | 1 | 0 | 1 | 700 | 1 | 700 | 0 | 1385 |
| Assembled_1731800-1732500 | Reference_1731800-1732500 | 99.86 | 700 | 1 | 0 | 1 | 700 | 1 | 700 | 0 | 1254 |
| Assembled_1739500-1740200 | Reference_1739500-1740200 | 100 | 700 | 0 | 0 | 1 | 700 | 1 | 700 | 0 | 1388 |
| Assembled_1747200-1747900 | Reference_1747200-1747900 | 99.86 | 700 | 1 | 0 | 1 | 700 | 1 | 700 | 0 | 1385 |
| Assembled_1754900-1755600 | Reference_1754900-1755600 | 95.71 | 700 | 30 | 0 | 1 | 700 | 1 | 700 | 0 | 1258 |
| Assembled_1762600-1763300 | Reference_1762600-1763300 | 99.43 | 700 | 4 | 0 | 1 | 700 | 1 | 700 | 0 | 1356 |
| Assembled_1770300-1771000 | Reference_1770300-1771000 | 98.5 | 468 | 7 | 0 | 233 | 700 | 233 | 700 | 0 | 901 |
| Assembled_1778000-1778700 | Reference_1778000-1778700 | 100 | 700 | 0 | 0 | 1 | 700 | 1 | 700 | 0 | 1388 |
| Assembled_1785700-1786400 | Reference_1785700-1786400 | 96.14 | 700 | 27 | 0 | 1 | 700 | 1 | 700 | 0 | 1230 |
| Assembled_1793400-1794100 | Reference_1793400-1794100 | 100 | 700 | 0 | 0 | 1 | 700 | 1 | 700 | 0 | 1388 |
| Assembled_1801100-1801800 | Reference_1801100-1801800 | 100 | 700 | 0 | 0 | 1 | 700 | 1 | 700 | 0 | 1388 |
| Assembled_1808800-1809500 | Reference_1808800-1809500 | 99.71 | 700 | 2 | 0 | 1 | 700 | 1 | 700 | 0 | 1335 |
| Assembled_1816500-1817200 | Reference_1816500-1817200 | 93.85 | 699 | 43 | 0 | 1 | 699 | 1 | 699 | 0 | 1209 |
| Assembled_1824200-1824900 | Reference_1824200-1824900 | 100 | 700 | 0 | 0 | 1 | 700 | 1 | 700 | 0 | 1388 |
| Assembled_1831900-1832600 | Reference_1831900-1832600 | 100 | 693 | 0 | 0 | 8 | 700 | 8 | 700 | 0 | 1374 |
| Assembled_1839600-1840300 | Reference_1839600-1840300 | 100 | 700 | 0 | 0 | 1 | 700 | 1 | 700 | 0 | 1346 |
| Assembled_1847300-1848000 | Reference_1847300-1848000 | 99.86 | 700 | 1 | 0 | 1 | 700 | 1 | 700 | 0 | 1380 |
| Assembled_1855000-1855700 | Reference_1855000-1855700 | 98.86 | 700 | 8 | 0 | 1 | 700 | 1 | 700 | 0 | 1356 |
| Assembled_1862700-1863400 | Reference_1862700-1863400 | 98.14 | 700 | 13 | 0 | 1 | 700 | 1 | 700 | 0 | 1211 |
| Assembled_1870400-1871100 | Reference_1870400-1871100 | 100 | 700 | 0 | 0 | 1 | 700 | 1 | 700 | 0 | 1388 |
| Assembled_1878100-1878800 | Reference_1878100-1878800 | 100 | 529 | 0 | 0 | 1 | 529 | 1 | 529 | 0 | 1049 |
| Assembled_1885800-1886500 | Reference_1885800-1886500 | 100 | 700 | 0 | 0 | 1 | 700 | 1 | 700 | 0 | 1388 |
| Assembled_1893500-1894200 | Reference_1893500-1894200 | 100 | 96 | 0 | 0 | 162 | 257 | 162 | 257 | 1e-49 | 190 |
| Assembled_1901200-1901900 | Reference_1901200-1901900 | 100 | 700 | 0 | 0 | 1 | 700 | 1 | 700 | 0 | 1346 |
| Assembled_1908900-1909600 | Reference_1908900-1909600 | 96 | 700 | 28 | 0 | 1 | 700 | 1 | 700 | 0 | 1297 |
| Assembled_1916600-1917300 | Reference_1916600-1917300 | 100 | 700 | 0 | 0 | 1 | 700 | 1 | 700 | 0 | 1388 |
| Assembled_1924300-1925000 | Reference_1924300-1925000 | 100 | 700 | 0 | 0 | 1 | 700 | 1 | 700 | 0 | 1388 |
| Assembled_1932000-1932700 | Reference_1932000-1932700 | 99.57 | 700 | 3 | 0 | 1 | 700 | 1 | 700 | 0 | 1380 |
| Assembled_1939700-1940400 | Reference_1939700-1940400 | 98 | 700 | 14 | 0 | 1 | 700 | 1 | 700 | 0 | 1291 |
| Assembled_1947400-1948100 | Reference_1947400-1948100 | 97.43 | 700 | 18 | 0 | 1 | 700 | 1 | 700 | 0 | 1239 |
| Assembled_1955100-1955800 | Reference_1955100-1955800 | 99.57 | 700 | 3 | 0 | 1 | 700 | 1 | 700 | 0 | 1364 |
| Assembled_1962800-1963500 | Reference_1962800-1963500 | 99.14 | 700 | 6 | 0 | 1 | 700 | 1 | 700 | 0 | 1372 |
| Assembled_1970500-1971200 | Reference_1970500-1971200 | 100 | 700 | 0 | 0 | 1 | 700 | 1 | 700 | 0 | 1346 |
| Assembled_1978200-1978900 | Reference_1978200-1978900 | 99.43 | 700 | 4 | 0 | 1 | 700 | 1 | 700 | 0 | 1356 |
| Assembled_1985900-1986600 | Reference_1985900-1986600 | 97 | 700 | 21 | 0 | 1 | 700 | 1 | 700 | 0 | 1231 |
| Assembled_1993600-1994300 | Reference_1993600-1994300 | 98.57 | 700 | 10 | 0 | 1 | 700 | 1 | 700 | 0 | 1361 |
| Assembled_2001300-2002000 | Reference_2001300-2002000 | 100 | 700 | 0 | 0 | 1 | 700 | 1 | 700 | 0 | 1388 |
| Assembled_2009000-2009700 | Reference_2009000-2009700 | 89.86 | 700 | 71 | 0 | 1 | 700 | 1 | 700 | 0 | 1012 |
| Assembled_2016700-2017400 | Reference_2016700-2017400 | 94.57 | 700 | 38 | 0 | 1 | 700 | 1 | 700 | 0 | 1219 |
| Assembled_2024400-2025100 | Reference_2024400-2025100 | 99.35 | 460 | 3 | 0 | 241 | 700 | 241 | 700 | 0 | 893 |
| Assembled_2032100-2032800 | Reference_2032100-2032800 | 99.61 | 519 | 2 | 0 | 71 | 589 | 71 | 589 | 0 | 916 |
| Assembled_2039800-2040500 | Reference_2039800-2040500 | 99.71 | 347 | 1 | 0 | 354 | 700 | 354 | 700 | 0 | 633 |
| Assembled_2047500-2048200 | Reference_2047500-2048200 | 96.71 | 700 | 23 | 0 | 1 | 700 | 1 | 700 | 0 | 1241 |
| Assembled_2055200-2055900 | Reference_2055200-2055900 | 99 | 700 | 7 | 0 | 1 | 700 | 1 | 700 | 0 | 1366 |
| Assembled_2062900-2063600 | Reference_2062900-2063600 | 100 | 700 | 0 | 0 | 1 | 700 | 1 | 700 | 0 | 1346 |
| Assembled_2070600-2071300 | Reference_2070600-2071300 | 100 | 700 | 0 | 0 | 1 | 700 | 1 | 700 | 0 | 1388 |
| Assembled_2078300-2079000 | Reference_2078300-2079000 | 98 | 700 | 14 | 0 | 1 | 700 | 1 | 700 | 0 | 1291 |
| Assembled_2086000-2086700 | Reference_2086000-2086700 | 99.43 | 699 | 4 | 0 | 2 | 700 | 2 | 700 | 0 | 1176 |
| Assembled_2093700-2094400 | Reference_2093700-2094400 | 99.71 | 698 | 2 | 0 | 3 | 700 | 3 | 700 | 0 | 1381 |
| Assembled_2101400-2102100 | Reference_2101400-2102100 | 93.71 | 700 | 44 | 0 | 1 | 700 | 1 | 700 | 0 | 1025 |
| Assembled_2109100-2109800 | Reference_2109100-2109800 | 98.71 | 698 | 9 | 0 | 1 | 698 | 1 | 698 | 0 | 1312 |
| Assembled_2116800-2117500 | Reference_2116800-2117500 | 97.86 | 700 | 15 | 0 | 1 | 700 | 1 | 700 | 0 | 1215 |
| Assembled_2124500-2125200 | Reference_2124500-2125200 | 99.14 | 700 | 6 | 0 | 1 | 700 | 1 | 700 | 0 | 1347 |
| Assembled_2132200-2132900 | Reference_2132200-2132900 | 99.86 | 700 | 1 | 0 | 1 | 700 | 1 | 700 | 0 | 1338 |
| Assembled_2139900-2140600 | Reference_2139900-2140600 | 100 | 700 | 0 | 0 | 1 | 700 | 1 | 700 | 0 | 1388 |
| Assembled_2147600-2148300 | Reference_2147600-2148300 | 96.44 | 562 | 20 | 0 | 125 | 686 | 125 | 686 | 0 | 940 |
| Assembled_2155300-2156000 | Reference_2155300-2156000 | 89.93 | 695 | 70 | 0 | 6 | 700 | 6 | 700 | 0 | 952 |
| Assembled_2163000-2163700 | Reference_2163000-2163700 | 92.43 | 700 | 53 | 0 | 1 | 700 | 1 | 700 | 0 | 1089 |
| Assembled_2170700-2171400 | Reference_2170700-2171400 | 97.86 | 700 | 15 | 0 | 1 | 700 | 1 | 700 | 0 | 1337 |
| Assembled_2178400-2179100 | Reference_2178400-2179100 | 99.14 | 697 | 6 | 0 | 1 | 697 | 1 | 697 | 0 | 1360 |
| Assembled_2186100-2186800 | Reference_2186100-2186800 | 99.57 | 700 | 3 | 0 | 1 | 700 | 1 | 700 | 0 | 1322 |
| Assembled_2193800-2194500 | Reference_2193800-2194500 | 99.29 | 700 | 5 | 0 | 1 | 700 | 1 | 700 | 0 | 1348 |
| Assembled_2201500-2202200 | Reference_2201500-2202200 | 100 | 487 | 0 | 0 | 214 | 700 | 214 | 700 | 0 | 965 |
| Assembled_2209200-2209900 | Reference_2209200-2209900 | 99.29 | 700 | 5 | 0 | 1 | 700 | 1 | 700 | 0 | 1348 |
| Assembled_2216900-2217600 | Reference_2216900-2217600 | 99.43 | 700 | 4 | 0 | 1 | 700 | 1 | 700 | 0 | 1314 |
| Assembled_2224600-2225300 | Reference_2224600-2225300 | 98.43 | 700 | 11 | 0 | 1 | 700 | 1 | 700 | 0 | 1332 |
| Assembled_2232300-2233000 | Reference_2232300-2233000 | 97.43 | 700 | 18 | 0 | 1 | 700 | 1 | 700 | 0 | 1302 |
| Assembled_2240000-2240700 | Reference_2240000-2240700 | 98.71 | 700 | 9 | 0 | 1 | 700 | 1 | 700 | 0 | 1279 |
| Assembled_2263100-2263800 | Reference_2263100-2263800 | 97.8 | 546 | 12 | 0 | 1 | 546 | 1 | 546 | 0 | 987 |
| Assembled_2270800-2271500 | Reference_2270800-2271500 | 99.43 | 700 | 4 | 0 | 1 | 700 | 1 | 700 | 0 | 1314 |
| Assembled_2278500-2279200 | Reference_2278500-2279200 | 100 | 700 | 0 | 0 | 1 | 700 | 1 | 700 | 0 | 1346 |
| Assembled_2286200-2286900 | Reference_2286200-2286900 | 99.71 | 683 | 2 | 0 | 18 | 700 | 18 | 700 | 0 | 1338 |
| Assembled_2293900-2294600 | Reference_2293900-2294600 | 99.86 | 700 | 1 | 0 | 1 | 700 | 1 | 700 | 0 | 1380 |
| Assembled_2301600-2302300 | Reference_2301600-2302300 | 99.71 | 700 | 2 | 0 | 1 | 700 | 1 | 700 | 0 | 1372 |
| Assembled_2309300-2310000 | Reference_2309300-2310000 | 100 | 87 | 0 | 0 | 462 | 548 | 462 | 548 | 2e-44 | 172 |
| Assembled_2317000-2317700 | Reference_2317000-2317700 | 98.71 | 700 | 9 | 0 | 1 | 700 | 1 | 700 | 0 | 1295 |
| Assembled_2324700-2325400 | Reference_2324700-2325400 | 99.71 | 700 | 2 | 0 | 1 | 700 | 1 | 700 | 0 | 1372 |
| Assembled_2332400-2333100 | Reference_2332400-2333100 | 99.28 | 699 | 5 | 0 | 2 | 700 | 2 | 700 | 0 | 1346 |
| Assembled_2340100-2340800 | Reference_2340100-2340800 | 99.86 | 700 | 1 | 0 | 1 | 700 | 1 | 700 | 0 | 1380 |
| Assembled_2347800-2348500 | Reference_2347800-2348500 | 89.94 | 696 | 70 | 0 | 5 | 700 | 5 | 700 | 0 | 1162 |
| Assembled_2355500-2356200 | Reference_2355500-2356200 | 94.39 | 410 | 23 | 0 | 1 | 410 | 1 | 410 | 0 | 692 |
| Assembled_2363200-2363900 | Reference_2363200-2363900 | 100 | 700 | 0 | 0 | 1 | 700 | 1 | 700 | 0 | 1388 |
| Assembled_2370900-2371600 | Reference_2370900-2371600 | 99.43 | 700 | 4 | 0 | 1 | 700 | 1 | 700 | 0 | 1356 |
| Assembled_2378600-2379300 | Reference_2378600-2379300 | 99.29 | 700 | 5 | 0 | 1 | 700 | 1 | 700 | 0 | 1348 |
| Assembled_2386300-2387000 | Reference_2386300-2387000 | 100 | 700 | 0 | 0 | 1 | 700 | 1 | 700 | 0 | 1346 |
| Assembled_2394000-2394700 | Reference_2394000-2394700 | 86.43 | 700 | 95 | 0 | 1 | 700 | 1 | 700 | 0 | 1005 |
| Assembled_2401700-2402400 | Reference_2401700-2402400 | 99.57 | 700 | 3 | 0 | 1 | 700 | 1 | 700 | 0 | 1364 |
| Assembled_2409400-2410100 | Reference_2409400-2410100 | 99.71 | 700 | 2 | 0 | 1 | 700 | 1 | 700 | 0 | 1372 |
| Assembled_2417100-2417800 | Reference_2417100-2417800 | 100 | 84 | 0 | 0 | 617 | 700 | 617 | 700 | 1e-42 | 167 |
| Assembled_2424800-2425500 | Reference_2424800-2425500 | 99.86 | 700 | 1 | 0 | 1 | 700 | 1 | 700 | 0 | 1296 |
| Assembled_2432500-2433200 | Reference_2432500-2433200 | 99.14 | 700 | 6 | 0 | 1 | 700 | 1 | 700 | 0 | 1340 |
| Assembled_2440200-2440900 | Reference_2440200-2440900 | 100 | 700 | 0 | 0 | 1 | 700 | 1 | 700 | 0 | 1388 |
| Assembled_2447900-2448600 | Reference_2447900-2448600 | 99.86 | 700 | 1 | 0 | 1 | 700 | 1 | 700 | 0 | 1385 |
| Assembled_2455600-2456300 | Reference_2455600-2456300 | 99.71 | 700 | 2 | 0 | 1 | 700 | 1 | 700 | 0 | 1372 |
| Assembled_2463300-2464000 | Reference_2463300-2464000 | 99.86 | 700 | 1 | 0 | 1 | 700 | 1 | 700 | 0 | 1343 |
| Assembled_2471000-2471700 | Reference_2471000-2471700 | 99.49 | 586 | 3 | 0 | 1 | 586 | 1 | 586 | 0 | 1138 |
| Assembled_2478700-2479400 | Reference_2478700-2479400 | 100 | 700 | 0 | 0 | 1 | 700 | 1 | 700 | 0 | 1388 |
| Assembled_2486400-2487100 | Reference_2486400-2487100 | 99.86 | 700 | 1 | 0 | 1 | 700 | 1 | 700 | 0 | 1338 |
| Assembled_2494100-2494800 | Reference_2494100-2494800 | 100 | 700 | 0 | 0 | 1 | 700 | 1 | 700 | 0 | 1388 |
| Assembled_2501800-2502500 | Reference_2501800-2502500 | 99 | 700 | 7 | 0 | 1 | 700 | 1 | 700 | 0 | 1306 |
| Assembled_2509500-2510200 | Reference_2509500-2510200 | 97.57 | 700 | 17 | 0 | 1 | 700 | 1 | 700 | 0 | 1263 |
| Assembled_2517200-2517900 | Reference_2517200-2517900 | 97.86 | 700 | 15 | 0 | 1 | 700 | 1 | 700 | 0 | 1269 |
| Assembled_2524900-2525600 | Reference_2524900-2525600 | 99.57 | 700 | 3 | 0 | 1 | 700 | 1 | 700 | 0 | 1333 |
| Assembled_2532600-2533300 | Reference_2532600-2533300 | 100 | 175 | 0 | 0 | 1 | 175 | 1 | 175 | 7e-97 | 347 |
| Assembled_2540300-2541000 | Reference_2540300-2541000 | 99.57 | 700 | 3 | 0 | 1 | 700 | 1 | 700 | 0 | 1380 |
| Assembled_2548000-2548700 | Reference_2548000-2548700 | 99.14 | 700 | 6 | 0 | 1 | 700 | 1 | 700 | 0 | 1366 |
| Assembled_2555700-2556400 | Reference_2555700-2556400 | 99.29 | 700 | 5 | 0 | 1 | 700 | 1 | 700 | 0 | 1256 |
| Assembled_2563400-2564100 | Reference_2563400-2564100 | 99 | 700 | 7 | 0 | 1 | 700 | 1 | 700 | 0 | 1251 |
| Assembled_2571100-2571800 | Reference_2571100-2571800 | 99.71 | 700 | 2 | 0 | 1 | 700 | 1 | 700 | 0 | 1377 |
| Assembled_2578800-2579500 | Reference_2578800-2579500 | 99.29 | 700 | 5 | 0 | 1 | 700 | 1 | 700 | 0 | 1265 |
| Assembled_2586500-2587200 | Reference_2586500-2587200 | 100 | 700 | 0 | 0 | 1 | 700 | 1 | 700 | 0 | 1340 |
| Assembled_2594200-2594900 | Reference_2594200-2594900 | 99 | 700 | 7 | 0 | 1 | 700 | 1 | 700 | 0 | 1353 |
| Assembled_2601900-2602600 | Reference_2601900-2602600 | 100 | 700 | 0 | 0 | 1 | 700 | 1 | 700 | 0 | 1126 |
| Assembled_2609600-2610300 | Reference_2609600-2610300 | 100 | 700 | 0 | 0 | 1 | 700 | 1 | 700 | 0 | 1388 |
| Assembled_2617300-2618000 | Reference_2617300-2618000 | 100 | 700 | 0 | 0 | 1 | 700 | 1 | 700 | 0 | 1298 |
| Assembled_2625000-2625700 | Reference_2625000-2625700 | 94.29 | 700 | 40 | 0 | 1 | 700 | 1 | 700 | 0 | 1277 |
| Assembled_2632700-2633400 | Reference_2632700-2633400 | 97.14 | 700 | 20 | 0 | 1 | 700 | 1 | 700 | 0 | 1334 |
| Assembled_2640400-2641100 | Reference_2640400-2641100 | 99.29 | 700 | 5 | 0 | 1 | 700 | 1 | 700 | 0 | 1358 |
| Assembled_2648100-2648800 | Reference_2648100-2648800 | 99.14 | 700 | 6 | 0 | 1 | 700 | 1 | 700 | 0 | 1314 |
| Assembled_2655800-2656500 | Reference_2655800-2656500 | 99.86 | 700 | 1 | 0 | 1 | 700 | 1 | 700 | 0 | 1380 |
| Assembled_2663500-2664200 | Reference_2663500-2664200 | 100 | 700 | 0 | 0 | 1 | 700 | 1 | 700 | 0 | 1388 |
| Assembled_2671200-2671900 | Reference_2671200-2671900 | 99.14 | 700 | 6 | 0 | 1 | 700 | 1 | 700 | 0 | 1340 |
| Assembled_2678900-2679600 | Reference_2678900-2679600 | 99.43 | 700 | 4 | 0 | 1 | 700 | 1 | 700 | 0 | 1295 |
| Assembled_2686600-2687300 | Reference_2686600-2687300 | 99.29 | 700 | 5 | 0 | 1 | 700 | 1 | 700 | 0 | 1348 |
| Assembled_2694300-2695000 | Reference_2694300-2695000 | 100 | 700 | 0 | 0 | 1 | 700 | 1 | 700 | 0 | 1388 |
| Assembled_2702000-2702700 | Reference_2702000-2702700 | 100 | 700 | 0 | 0 | 1 | 700 | 1 | 700 | 0 | 1388 |
| Assembled_2709700-2710400 | Reference_2709700-2710400 | 100 | 700 | 0 | 0 | 1 | 700 | 1 | 700 | 0 | 1388 |
| Assembled_2717400-2718100 | Reference_2717400-2718100 | 99.57 | 700 | 3 | 0 | 1 | 700 | 1 | 700 | 0 | 1364 |
| Assembled_2725100-2725800 | Reference_2725100-2725800 | 99.43 | 700 | 4 | 0 | 1 | 700 | 1 | 700 | 0 | 1377 |
| Assembled_2732800-2733500 | Reference_2732800-2733500 | 94 | 700 | 42 | 0 | 1 | 700 | 1 | 700 | 0 | 1175 |
| Assembled_2740500-2741200 | Reference_2740500-2741200 | 95.29 | 700 | 33 | 0 | 1 | 700 | 1 | 700 | 0 | 1177 |
| Assembled_2748200-2748900 | Reference_2748200-2748900 | 100 | 700 | 0 | 0 | 1 | 700 | 1 | 700 | 0 | 1388 |
| Assembled_2755900-2756600 | Reference_2755900-2756600 | 100 | 44 | 0 | 0 | 109 | 152 | 109 | 152 | 1e-18 | 87.7 |
| Assembled_2763600-2764300 | Reference_2763600-2764300 | 94.27 | 698 | 40 | 0 | 3 | 700 | 3 | 700 | 0 | 1253 |
| Assembled_2771300-2772000 | Reference_2771300-2772000 | 99.14 | 700 | 6 | 0 | 1 | 700 | 1 | 700 | 0 | 1372 |
| Assembled_2779000-2779700 | Reference_2779000-2779700 | 98 | 700 | 14 | 0 | 1 | 700 | 1 | 700 | 0 | 1339 |
| Assembled_2786700-2787400 | Reference_2786700-2787400 | 99.71 | 700 | 2 | 0 | 1 | 700 | 1 | 700 | 0 | 1332 |
| Assembled_2794400-2795100 | Reference_2794400-2795100 | 98.26 | 688 | 12 | 0 | 1 | 688 | 1 | 688 | 0 | 1279 |
| Assembled_2802100-2802800 | Reference_2802100-2802800 | 98.57 | 700 | 10 | 0 | 1 | 700 | 1 | 700 | 0 | 1329 |
| Assembled_2809800-2810500 | Reference_2809800-2810500 | 96.7 | 698 | 23 | 0 | 1 | 698 | 1 | 698 | 0 | 1317 |
| Assembled_2817500-2818200 | Reference_2817500-2818200 | 96.86 | 700 | 22 | 0 | 1 | 700 | 1 | 700 | 0 | 1286 |
| Assembled_2825200-2825900 | Reference_2825200-2825900 | 100 | 700 | 0 | 0 | 1 | 700 | 1 | 700 | 0 | 1388 |
| Assembled_2832900-2833600 | Reference_2832900-2833600 | 99.57 | 700 | 3 | 0 | 1 | 700 | 1 | 700 | 0 | 1364 |
| Assembled_2840600-2841300 | Reference_2840600-2841300 | 98.86 | 700 | 8 | 0 | 1 | 700 | 1 | 700 | 0 | 1345 |
| Assembled_2848300-2849000 | Reference_2848300-2849000 | 98.57 | 700 | 10 | 0 | 1 | 700 | 1 | 700 | 0 | 1319 |
| Assembled_2856000-2856700 | Reference_2856000-2856700 | 99.57 | 700 | 3 | 0 | 1 | 700 | 1 | 700 | 0 | 1369 |
| Assembled_2863700-2864400 | Reference_2863700-2864400 | 99.57 | 700 | 3 | 0 | 1 | 700 | 1 | 700 | 0 | 1364 |
| Assembled_2871400-2872100 | Reference_2871400-2872100 | 99.14 | 700 | 6 | 0 | 1 | 700 | 1 | 700 | 0 | 1298 |
| Assembled_2879100-2879800 | Reference_2879100-2879800 | 99.71 | 700 | 2 | 0 | 1 | 700 | 1 | 700 | 0 | 1341 |
| Assembled_2886800-2887500 | Reference_2886800-2887500 | 99.43 | 700 | 4 | 0 | 1 | 700 | 1 | 700 | 0 | 1314 |
| Assembled_2894500-2895200 | Reference_2894500-2895200 | 98.14 | 700 | 13 | 0 | 1 | 700 | 1 | 700 | 0 | 1311 |
| Assembled_2902200-2902900 | Reference_2902200-2902900 | 100 | 700 | 0 | 0 | 1 | 700 | 1 | 700 | 0 | 1388 |
| Assembled_2909900-2910600 | Reference_2909900-2910600 | 98.42 | 379 | 6 | 0 | 322 | 700 | 322 | 700 | 0 | 723 |
| Assembled_2917600-2918300 | Reference_2917600-2918300 | 99.71 | 700 | 2 | 0 | 1 | 700 | 1 | 700 | 0 | 1341 |
| Assembled_2925300-2926000 | Reference_2925300-2926000 | 98.43 | 700 | 11 | 0 | 1 | 700 | 1 | 700 | 0 | 1300 |
| Assembled_2933000-2933700 | Reference_2933000-2933700 | 98.57 | 700 | 10 | 0 | 1 | 700 | 1 | 700 | 0 | 1308 |
| Assembled_2940700-2941400 | Reference_2940700-2941400 | 100 | 700 | 0 | 0 | 1 | 700 | 1 | 700 | 0 | 1388 |
| Assembled_2948400-2949100 | Reference_2948400-2949100 | 95.29 | 700 | 33 | 0 | 1 | 700 | 1 | 700 | 0 | 1281 |
| Assembled_2956100-2956800 | Reference_2956100-2956800 | 99.71 | 700 | 2 | 0 | 1 | 700 | 1 | 700 | 0 | 1382 |
| Assembled_2963800-2964500 | Reference_2963800-2964500 | 99.71 | 700 | 2 | 0 | 1 | 700 | 1 | 700 | 0 | 1324 |
| Assembled_2971500-2972200 | Reference_2971500-2972200 | 99.86 | 700 | 1 | 0 | 1 | 700 | 1 | 700 | 0 | 1380 |
| Assembled_2979200-2979900 | Reference_2979200-2979900 | 98.14 | 700 | 13 | 0 | 1 | 700 | 1 | 700 | 0 | 1353 |
| Assembled_2986900-2987600 | Reference_2986900-2987600 | 98.57 | 700 | 10 | 0 | 1 | 700 | 1 | 700 | 0 | 1267 |
| Assembled_2994600-2995300 | Reference_2994600-2995300 | 98.14 | 700 | 13 | 0 | 1 | 700 | 1 | 700 | 0 | 1353 |
| Assembled_3002300-3003000 | Reference_3002300-3003000 | 99.86 | 699 | 1 | 0 | 2 | 700 | 2 | 700 | 0 | 1378 |
| Assembled_3010000-3010700 | Reference_3010000-3010700 | 99.14 | 700 | 6 | 0 | 1 | 700 | 1 | 700 | 0 | 1356 |
| Assembled_3017700-3018400 | Reference_3017700-3018400 | 99.57 | 700 | 3 | 0 | 1 | 700 | 1 | 700 | 0 | 1338 |
| Assembled_3025400-3026100 | Reference_3025400-3026100 | 98.43 | 700 | 11 | 0 | 1 | 700 | 1 | 700 | 0 | 1300 |
| Assembled_3033100-3033800 | Reference_3033100-3033800 | 99.57 | 700 | 3 | 0 | 1 | 700 | 1 | 700 | 0 | 1369 |
| Assembled_3040800-3041500 | Reference_3040800-3041500 | 99.14 | 697 | 6 | 0 | 3 | 699 | 3 | 699 | 0 | 1355 |
| Assembled_3048500-3049200 | Reference_3048500-3049200 | 99.14 | 700 | 6 | 0 | 1 | 700 | 1 | 700 | 0 | 1356 |
| Assembled_3056200-3056900 | Reference_3056200-3056900 | 100 | 700 | 0 | 0 | 1 | 700 | 1 | 700 | 0 | 1388 |
| Assembled_3063900-3064600 | Reference_3063900-3064600 | 99.86 | 700 | 1 | 0 | 1 | 700 | 1 | 700 | 0 | 1385 |
| Assembled_3071600-3072300 | Reference_3071600-3072300 | 99.86 | 700 | 1 | 0 | 1 | 700 | 1 | 700 | 0 | 1296 |
| Assembled_3079300-3080000 | Reference_3079300-3080000 | 93.53 | 696 | 45 | 0 | 2 | 697 | 2 | 697 | 0 | 1171 |
| Assembled_3087000-3087700 | Reference_3087000-3087700 | 99.86 | 700 | 1 | 0 | 1 | 700 | 1 | 700 | 0 | 1385 |
| Assembled_3094700-3095400 | Reference_3094700-3095400 | 99.71 | 700 | 2 | 0 | 1 | 700 | 1 | 700 | 0 | 1299 |
| Assembled_3102400-3103100 | Reference_3102400-3103100 | 99.14 | 700 | 6 | 0 | 1 | 700 | 1 | 700 | 0 | 1274 |
| Assembled_3110100-3110800 | Reference_3110100-3110800 | 98.45 | 645 | 10 | 0 | 56 | 700 | 56 | 700 | 0 | 1199 |
| Assembled_3117800-3118500 | Reference_3117800-3118500 | 97.69 | 692 | 16 | 0 | 1 | 692 | 1 | 692 | 0 | 1250 |
| Assembled_3125500-3126200 | Reference_3125500-3126200 | 99.86 | 700 | 1 | 0 | 1 | 700 | 1 | 700 | 0 | 1380 |
| Assembled_3133200-3133900 | Reference_3133200-3133900 | 100 | 700 | 0 | 0 | 1 | 700 | 1 | 700 | 0 | 1388 |
| Assembled_3140900-3141600 | Reference_3140900-3141600 | 99.84 | 623 | 1 | 0 | 78 | 700 | 78 | 700 | 0 | 1179 |
| Assembled_3148600-3149300 | Reference_3148600-3149300 | 100 | 700 | 0 | 0 | 1 | 700 | 1 | 700 | 0 | 1298 |
| Assembled_3156300-3157000 | Reference_3156300-3157000 | 99.43 | 700 | 4 | 0 | 1 | 700 | 1 | 700 | 0 | 1356 |
| Assembled_3164000-3164700 | Reference_3164000-3164700 | 99.86 | 700 | 1 | 0 | 1 | 700 | 1 | 700 | 0 | 1380 |
| Assembled_3171700-3172400 | Reference_3171700-3172400 | 99.43 | 700 | 4 | 0 | 1 | 700 | 1 | 700 | 0 | 1356 |
| Assembled_3179400-3180100 | Reference_3179400-3180100 | 99.71 | 700 | 2 | 0 | 1 | 700 | 1 | 700 | 0 | 1372 |
| Assembled_3187100-3187800 | Reference_3187100-3187800 | 99.57 | 700 | 3 | 0 | 1 | 700 | 1 | 700 | 0 | 1380 |
| Assembled_3194800-3195500 | Reference_3194800-3195500 | 99.14 | 700 | 6 | 0 | 1 | 700 | 1 | 700 | 0 | 1372 |
| Assembled_3202500-3203200 | Reference_3202500-3203200 | 99.43 | 700 | 4 | 0 | 1 | 700 | 1 | 700 | 0 | 1325 |
| Assembled_3210200-3210900 | Reference_3210200-3210900 | 99.86 | 700 | 1 | 0 | 1 | 700 | 1 | 700 | 0 | 1338 |
| Assembled_3217900-3218600 | Reference_3217900-3218600 | 99.86 | 700 | 1 | 0 | 1 | 700 | 1 | 700 | 0 | 1380 |
| Assembled_3225600-3226300 | Reference_3225600-3226300 | 99.57 | 700 | 3 | 0 | 1 | 700 | 1 | 700 | 0 | 1364 |
| Assembled_3233300-3234000 | Reference_3233300-3234000 | 99.29 | 700 | 5 | 0 | 1 | 700 | 1 | 700 | 0 | 1312 |
| Assembled_3241000-3241700 | Reference_3241000-3241700 | 99.82 | 557 | 1 | 0 | 1 | 557 | 1 | 557 | 0 | 1096 |
| Assembled_3248700-3249400 | Reference_3248700-3249400 | 100 | 700 | 0 | 0 | 1 | 700 | 1 | 700 | 0 | 1346 |
| Assembled_3256400-3257100 | Reference_3256400-3257100 | 99.71 | 700 | 2 | 0 | 1 | 700 | 1 | 700 | 0 | 1330 |
| Assembled_3264100-3264800 | Reference_3264100-3264800 | 100 | 700 | 0 | 0 | 1 | 700 | 1 | 700 | 0 | 1388 |
| Assembled_3271800-3272500 | Reference_3271800-3272500 | 99.86 | 700 | 1 | 0 | 1 | 700 | 1 | 700 | 0 | 1338 |
| Assembled_3279500-3280200 | Reference_3279500-3280200 | 100 | 700 | 0 | 0 | 1 | 700 | 1 | 700 | 0 | 1388 |
| Assembled_3287200-3287900 | Reference_3287200-3287900 | 97.86 | 700 | 15 | 0 | 1 | 700 | 1 | 700 | 0 | 1268 |
| Assembled_3294900-3295600 | Reference_3294900-3295600 | 97.71 | 698 | 16 | 0 | 1 | 698 | 1 | 698 | 0 | 1342 |
| Assembled_3302600-3303300 | Reference_3302600-3303300 | 99.71 | 341 | 1 | 0 | 1 | 341 | 1 | 341 | 0 | 626 |
| Assembled_3310300-3311000 | Reference_3310300-3311000 | 94.12 | 697 | 41 | 0 | 1 | 697 | 1 | 697 | 0 | 1263 |
| Assembled_3318000-3318700 | Reference_3318000-3318700 | 99.29 | 700 | 5 | 0 | 1 | 700 | 1 | 700 | 0 | 1322 |
| Assembled_3325700-3326400 | Reference_3325700-3326400 | 99.71 | 700 | 2 | 0 | 1 | 700 | 1 | 700 | 0 | 1377 |
| Assembled_3333400-3334100 | Reference_3333400-3334100 | 100 | 700 | 0 | 0 | 1 | 700 | 1 | 700 | 0 | 1388 |
| Assembled_3341100-3341800 | Reference_3341100-3341800 | 100 | 700 | 0 | 0 | 1 | 700 | 1 | 700 | 0 | 1346 |
| Assembled_3348800-3349500 | Reference_3348800-3349500 | 99.57 | 700 | 3 | 0 | 1 | 700 | 1 | 700 | 0 | 1374 |
| Assembled_3356500-3357200 | Reference_3356500-3357200 | 99.86 | 700 | 1 | 0 | 1 | 700 | 1 | 700 | 0 | 1338 |
| Assembled_3364200-3364900 | Reference_3364200-3364900 | 97.86 | 700 | 15 | 0 | 1 | 700 | 1 | 700 | 0 | 1331 |
| Assembled_3371900-3372600 | Reference_3371900-3372600 | 98.71 | 700 | 9 | 0 | 1 | 700 | 1 | 700 | 0 | 1316 |
| Assembled_3379600-3380300 | Reference_3379600-3380300 | 99.86 | 700 | 1 | 0 | 1 | 700 | 1 | 700 | 0 | 1380 |
| Assembled_3387300-3388000 | Reference_3387300-3388000 | 99.78 | 457 | 1 | 0 | 244 | 700 | 244 | 700 | 0 | 899 |
| Assembled_3395000-3395700 | Reference_3395000-3395700 | 99.29 | 700 | 5 | 0 | 1 | 700 | 1 | 700 | 0 | 1300 |
| Assembled_3402700-3403400 | Reference_3402700-3403400 | 99.71 | 700 | 2 | 0 | 1 | 700 | 1 | 700 | 0 | 1377 |
| Assembled_3410400-3411100 | Reference_3410400-3411100 | 100 | 383 | 0 | 0 | 1 | 383 | 1 | 383 | 0 | 677 |
| Assembled_3418100-3418800 | Reference_3418100-3418800 | 100 | 485 | 0 | 0 | 1 | 485 | 1 | 485 | 0 | 961 |
| Assembled_3425800-3426500 | Reference_3425800-3426500 | 99.43 | 698 | 4 | 0 | 1 | 698 | 1 | 698 | 0 | 1352 |
| Assembled_3433500-3434200 | Reference_3433500-3434200 | 99 | 700 | 7 | 0 | 1 | 700 | 1 | 700 | 0 | 1322 |
| Assembled_3441200-3441900 | Reference_3441200-3441900 | 90.9 | 659 | 60 | 0 | 42 | 700 | 42 | 700 | 0 | 972 |
| Assembled_3448900-3449600 | Reference_3448900-3449600 | 99.57 | 700 | 3 | 0 | 1 | 700 | 1 | 700 | 0 | 1364 |
| Assembled_3456600-3457300 | Reference_3456600-3457300 | 98.14 | 700 | 13 | 0 | 1 | 700 | 1 | 700 | 0 | 1342 |
| Assembled_3464300-3465000 | Reference_3464300-3465000 | 98.26 | 576 | 10 | 0 | 125 | 700 | 125 | 700 | 0 | 1047 |
| Assembled_3472000-3472700 | Reference_3472000-3472700 | 100 | 700 | 0 | 0 | 1 | 700 | 1 | 700 | 0 | 1388 |
| Assembled_3479700-3480400 | Reference_3479700-3480400 | 99.57 | 700 | 3 | 0 | 1 | 700 | 1 | 700 | 0 | 1380 |
| Assembled_3487400-3488100 | Reference_3487400-3488100 | 98 | 700 | 14 | 0 | 1 | 700 | 1 | 700 | 0 | 1350 |
| Assembled_3495100-3495800 | Reference_3495100-3495800 | 97.65 | 681 | 16 | 0 | 20 | 700 | 20 | 700 | 0 | 1253 |
| Assembled_3502800-3503500 | Reference_3502800-3503500 | 100 | 700 | 0 | 0 | 1 | 700 | 1 | 700 | 0 | 1215 |
| Assembled_3510500-3511200 | Reference_3510500-3511200 | 100 | 700 | 0 | 0 | 1 | 700 | 1 | 700 | 0 | 1298 |
| Assembled_3518200-3518900 | Reference_3518200-3518900 | 99.71 | 700 | 2 | 0 | 1 | 700 | 1 | 700 | 0 | 1247 |
| Assembled_3525900-3526600 | Reference_3525900-3526600 | 98 | 700 | 14 | 0 | 1 | 700 | 1 | 700 | 0 | 1302 |
| Assembled_3533600-3534300 | Reference_3533600-3534300 | 99.86 | 700 | 1 | 0 | 1 | 700 | 1 | 700 | 0 | 1380 |
| Assembled_3541300-3542000 | Reference_3541300-3542000 | 99.43 | 700 | 4 | 0 | 1 | 700 | 1 | 700 | 0 | 1361 |
| Assembled_3549000-3549700 | Reference_3549000-3549700 | 100 | 700 | 0 | 0 | 1 | 700 | 1 | 700 | 0 | 1388 |
| Assembled_3556700-3557400 | Reference_3556700-3557400 | 99.43 | 700 | 4 | 0 | 1 | 700 | 1 | 700 | 0 | 1314 |
| Assembled_3564400-3565100 | Reference_3564400-3565100 | 100 | 690 | 0 | 0 | 11 | 700 | 11 | 700 | 0 | 1326 |
| Assembled_3572100-3572800 | Reference_3572100-3572800 | 100 | 700 | 0 | 0 | 1 | 700 | 1 | 700 | 0 | 1334 |
| Assembled_3579800-3580500 | Reference_3579800-3580500 | 98.71 | 699 | 9 | 0 | 2 | 700 | 2 | 700 | 0 | 1323 |
| Assembled_3587500-3588200 | Reference_3587500-3588200 | 99.86 | 700 | 1 | 0 | 1 | 700 | 1 | 700 | 0 | 1380 |
| Assembled_3595200-3595900 | Reference_3595200-3595900 | 100 | 683 | 0 | 0 | 1 | 683 | 1 | 683 | 0 | 1312 |
| Assembled_3602900-3603600 | Reference_3602900-3603600 | 99.81 | 537 | 1 | 0 | 164 | 700 | 164 | 700 | 0 | 1057 |
| Assembled_3610600-3611300 | Reference_3610600-3611300 | 96.57 | 700 | 24 | 0 | 1 | 700 | 1 | 700 | 0 | 1317 |
| Assembled_3618300-3619000 | Reference_3618300-3619000 | 99.86 | 700 | 1 | 0 | 1 | 700 | 1 | 700 | 0 | 1380 |
| Assembled_3626000-3626700 | Reference_3626000-3626700 | 100 | 700 | 0 | 0 | 1 | 700 | 1 | 700 | 0 | 1388 |
| Assembled_3633700-3634400 | Reference_3633700-3634400 | 100 | 700 | 0 | 0 | 1 | 700 | 1 | 700 | 0 | 1346 |
| Assembled_3641400-3642100 | Reference_3641400-3642100 | 100 | 372 | 0 | 0 | 1 | 372 | 1 | 372 | 0 | 737 |
| Assembled_3649100-3649800 | Reference_3649100-3649800 | 98.71 | 700 | 9 | 0 | 1 | 700 | 1 | 700 | 0 | 1348 |
| Assembled_3656800-3657500 | Reference_3656800-3657500 | 100 | 700 | 0 | 0 | 1 | 700 | 1 | 700 | 0 | 1388 |
| Assembled_3664500-3665200 | Reference_3664500-3665200 | 99.43 | 700 | 4 | 0 | 1 | 700 | 1 | 700 | 0 | 1314 |
| Assembled_3672200-3672900 | Reference_3672200-3672900 | 92.62 | 461 | 34 | 0 | 240 | 700 | 240 | 700 | 0 | 767 |
| Assembled_3679900-3680600 | Reference_3679900-3680600 | 99.26 | 677 | 5 | 0 | 24 | 700 | 24 | 700 | 0 | 1302 |
| Assembled_3687600-3688300 | Reference_3687600-3688300 | 99.43 | 700 | 4 | 0 | 1 | 700 | 1 | 700 | 0 | 1325 |
| Assembled_3695300-3696000 | Reference_3695300-3696000 | 100 | 349 | 0 | 0 | 1 | 349 | 1 | 349 | 0 | 650 |
| Assembled_3703000-3703700 | Reference_3703000-3703700 | 99.71 | 700 | 2 | 0 | 1 | 700 | 1 | 700 | 0 | 1372 |
| Assembled_3710700-3711400 | Reference_3710700-3711400 | 100 | 700 | 0 | 0 | 1 | 700 | 1 | 700 | 0 | 1388 |
| Assembled_3718400-3719100 | Reference_3718400-3719100 | 99.71 | 700 | 2 | 0 | 1 | 700 | 1 | 700 | 0 | 1335 |
| Assembled_3726100-3726800 | Reference_3726100-3726800 | 99.14 | 700 | 6 | 0 | 1 | 700 | 1 | 700 | 0 | 1340 |
| Assembled_3733800-3734500 | Reference_3733800-3734500 | 100 | 700 | 0 | 0 | 1 | 700 | 1 | 700 | 0 | 1388 |
| Assembled_3741500-3742200 | Reference_3741500-3742200 | 100 | 700 | 0 | 0 | 1 | 700 | 1 | 700 | 0 | 1304 |
| Assembled_3749200-3749900 | Reference_3749200-3749900 | 99.57 | 700 | 3 | 0 | 1 | 700 | 1 | 700 | 0 | 1327 |
| Assembled_3756900-3757600 | Reference_3756900-3757600 | 98.29 | 700 | 12 | 0 | 1 | 700 | 1 | 700 | 0 | 1350 |
| Assembled_3764600-3765300 | Reference_3764600-3765300 | 99.86 | 700 | 1 | 0 | 1 | 700 | 1 | 700 | 0 | 1296 |
| Assembled_3772300-3773000 | Reference_3772300-3773000 | 98.86 | 700 | 8 | 0 | 1 | 700 | 1 | 700 | 0 | 1356 |
| Assembled_3780000-3780700 | Reference_3780000-3780700 | 99.14 | 700 | 6 | 0 | 1 | 700 | 1 | 700 | 0 | 1356 |
| Assembled_3787700-3788400 | Reference_3787700-3788400 | 99.57 | 700 | 3 | 0 | 1 | 700 | 1 | 700 | 0 | 1364 |
| Assembled_3795400-3796100 | Reference_3795400-3796100 | 99.43 | 700 | 4 | 0 | 1 | 700 | 1 | 700 | 0 | 1356 |
| Assembled_3803100-3803800 | Reference_3803100-3803800 | 99.43 | 700 | 4 | 0 | 1 | 700 | 1 | 700 | 0 | 1356 |
| Assembled_3810800-3811500 | Reference_3810800-3811500 | 99.86 | 700 | 1 | 0 | 1 | 700 | 1 | 700 | 0 | 1380 |
| Assembled_3818500-3819200 | Reference_3818500-3819200 | 99.43 | 700 | 4 | 0 | 1 | 700 | 1 | 700 | 0 | 1267 |
| Assembled_3826200-3826900 | Reference_3826200-3826900 | 99 | 700 | 7 | 0 | 1 | 700 | 1 | 700 | 0 | 1311 |
| Assembled_3833900-3834600 | Reference_3833900-3834600 | 99.71 | 700 | 2 | 0 | 1 | 700 | 1 | 700 | 0 | 1324 |
| Assembled_3841600-3842300 | Reference_3841600-3842300 | 99.57 | 700 | 3 | 0 | 1 | 700 | 1 | 700 | 0 | 1374 |
| Assembled_3849300-3850000 | Reference_3849300-3850000 | 99.57 | 700 | 3 | 0 | 1 | 700 | 1 | 700 | 0 | 1322 |
| Assembled_3857000-3857700 | Reference_3857000-3857700 | 99.86 | 700 | 1 | 0 | 1 | 700 | 1 | 700 | 0 | 1380 |
| Assembled_3864700-3865400 | Reference_3864700-3865400 | 98.86 | 700 | 8 | 0 | 1 | 700 | 1 | 700 | 0 | 1361 |
| Assembled_3872400-3873100 | Reference_3872400-3873100 | 99.14 | 700 | 6 | 0 | 1 | 700 | 1 | 700 | 0 | 1372 |
| Assembled_3880100-3880800 | Reference_3880100-3880800 | 99.57 | 700 | 3 | 0 | 1 | 700 | 1 | 700 | 0 | 1327 |
| Assembled_3887800-3888500 | Reference_3887800-3888500 | 99.57 | 700 | 3 | 0 | 1 | 700 | 1 | 700 | 0 | 1322 |
| Assembled_3895500-3896200 | Reference_3895500-3896200 | 98.76 | 564 | 7 | 0 | 1 | 564 | 1 | 564 | 0 | 973 |
| Assembled_3903200-3903900 | Reference_3903200-3903900 | 99 | 700 | 7 | 0 | 1 | 700 | 1 | 700 | 0 | 1309 |
| Assembled_3910900-3911600 | Reference_3910900-3911600 | 99.86 | 700 | 1 | 0 | 1 | 700 | 1 | 700 | 0 | 1380 |
| Assembled_3918600-3919300 | Reference_3918600-3919300 | 99.29 | 700 | 5 | 0 | 1 | 700 | 1 | 700 | 0 | 1348 |
| Assembled_3926300-3927000 | Reference_3926300-3927000 | 99.86 | 700 | 1 | 0 | 1 | 700 | 1 | 700 | 0 | 1380 |
| Assembled_3934000-3934700 | Reference_3934000-3934700 | 94.14 | 700 | 41 | 0 | 1 | 700 | 1 | 700 | 0 | 1082 |
| Assembled_3941700-3942400 | Reference_3941700-3942400 | 98.85 | 435 | 5 | 0 | 266 | 700 | 266 | 700 | 0 | 823 |
| Assembled_3949400-3950100 | Reference_3949400-3950100 | 100 | 700 | 0 | 0 | 1 | 700 | 1 | 700 | 0 | 1388 |
| Assembled_3957100-3957800 | Reference_3957100-3957800 | 99.86 | 700 | 1 | 0 | 1 | 700 | 1 | 700 | 0 | 1380 |
| Assembled_3964800-3965500 | Reference_3964800-3965500 | 99.86 | 700 | 1 | 0 | 1 | 700 | 1 | 700 | 0 | 1380 |
| Assembled_3972500-3973200 | Reference_3972500-3973200 | 99.86 | 700 | 1 | 0 | 1 | 700 | 1 | 700 | 0 | 1380 |
| Assembled_3980200-3980900 | Reference_3980200-3980900 | 98.99 | 494 | 5 | 0 | 207 | 700 | 207 | 700 | 0 | 950 |
| Assembled_3987900-3988600 | Reference_3987900-3988600 | 95.14 | 700 | 34 | 0 | 1 | 700 | 1 | 700 | 0 | 1294 |
| Assembled_3995600-3996300 | Reference_3995600-3996300 | 100 | 178 | 0 | 0 | 523 | 700 | 523 | 700 | 1e-98 | 353 |
| Assembled_4003300-4004000 | Reference_4003300-4004000 | 99.57 | 700 | 3 | 0 | 1 | 700 | 1 | 700 | 0 | 1338 |
| Assembled_4011000-4011700 | Reference_4011000-4011700 | 99.71 | 700 | 2 | 0 | 1 | 700 | 1 | 700 | 0 | 1377 |
| Assembled_4018700-4019400 | Reference_4018700-4019400 | 99.57 | 700 | 3 | 0 | 1 | 700 | 1 | 700 | 0 | 1369 |
| Assembled_4026400-4027100 | Reference_4026400-4027100 | 99.14 | 700 | 6 | 0 | 1 | 700 | 1 | 700 | 0 | 1340 |
| Assembled_4034100-4034800 | Reference_4034100-4034800 | 92.71 | 700 | 51 | 0 | 1 | 700 | 1 | 700 | 0 | 1219 |
| Assembled_4041800-4042500 | Reference_4041800-4042500 | 98.6 | 429 | 6 | 0 | 272 | 700 | 272 | 700 | 0 | 822 |
| Assembled_4049500-4050200 | Reference_4049500-4050200 | 99.71 | 700 | 2 | 0 | 1 | 700 | 1 | 700 | 0 | 1372 |
| Assembled_4057200-4057900 | Reference_4057200-4057900 | 98.57 | 700 | 10 | 0 | 1 | 700 | 1 | 700 | 0 | 1319 |
| Assembled_4064900-4065600 | Reference_4064900-4065600 | 97.71 | 700 | 16 | 0 | 1 | 700 | 1 | 700 | 0 | 1266 |
| Assembled_4072600-4073300 | Reference_4072600-4073300 | 99.57 | 700 | 3 | 0 | 1 | 700 | 1 | 700 | 0 | 1322 |
| Assembled_4080300-4081000 | Reference_4080300-4081000 | 99.57 | 700 | 3 | 0 | 1 | 700 | 1 | 700 | 0 | 1322 |
| Assembled_4088000-4088700 | Reference_4088000-4088700 | 95.29 | 700 | 33 | 0 | 1 | 700 | 1 | 700 | 0 | 1161 |
| Assembled_4095700-4096400 | Reference_4095700-4096400 | 99.4 | 336 | 2 | 0 | 365 | 700 | 365 | 700 | 0 | 650 |
| Assembled_4103400-4104100 | Reference_4103400-4104100 | 100 | 700 | 0 | 0 | 1 | 700 | 1 | 700 | 0 | 1388 |
| Assembled_4111100-4111800 | Reference_4111100-4111800 | 97.57 | 700 | 17 | 0 | 1 | 700 | 1 | 700 | 0 | 1284 |
| Assembled_4118800-4119500 | Reference_4118800-4119500 | 99.86 | 700 | 1 | 0 | 1 | 700 | 1 | 700 | 0 | 1380 |
| Assembled_4126500-4127200 | Reference_4126500-4127200 | 98.71 | 700 | 9 | 0 | 1 | 700 | 1 | 700 | 0 | 1316 |
| Assembled_4134200-4134900 | Reference_4134200-4134900 | 96.71 | 700 | 23 | 0 | 1 | 700 | 1 | 700 | 0 | 1188 |
| Assembled_4141900-4142600 | Reference_4141900-4142600 | 99 | 700 | 7 | 0 | 1 | 700 | 1 | 700 | 0 | 1332 |
| Assembled_4149600-4150300 | Reference_4149600-4150300 | 99.86 | 700 | 1 | 0 | 1 | 700 | 1 | 700 | 0 | 1337 |
| Assembled_4157300-4158000 | Reference_4157300-4158000 | 99.86 | 700 | 1 | 0 | 1 | 700 | 1 | 700 | 0 | 1380 |
| Assembled_4165000-4165700 | Reference_4165000-4165700 | 100 | 700 | 0 | 0 | 1 | 700 | 1 | 700 | 0 | 1132 |
| Assembled_4172700-4173400 | Reference_4172700-4173400 | 99.57 | 700 | 3 | 0 | 1 | 700 | 1 | 700 | 0 | 1333 |
| Assembled_4180400-4181100 | Reference_4180400-4181100 | 100 | 277 | 0 | 0 | 1 | 277 | 1 | 277 | 1e-157 | 549 |
| Assembled_4188100-4188800 | Reference_4188100-4188800 | 99.57 | 700 | 3 | 0 | 1 | 700 | 1 | 700 | 0 | 1364 |
| Assembled_4195800-4196500 | Reference_4195800-4196500 | 99.09 | 330 | 3 | 0 | 297 | 626 | 297 | 626 | 0 | 644 |
| Assembled_4203500-4204200 | Reference_4203500-4204200 | 99.29 | 700 | 5 | 0 | 1 | 700 | 1 | 700 | 0 | 1358 |
| Assembled_4211200-4211900 | Reference_4211200-4211900 | 98.43 | 700 | 11 | 0 | 1 | 700 | 1 | 700 | 0 | 1300 |
| Assembled_4218900-4219600 | Reference_4218900-4219600 | 99.71 | 700 | 2 | 0 | 1 | 700 | 1 | 700 | 0 | 1330 |
| Assembled_4226600-4227300 | Reference_4226600-4227300 | 98.71 | 700 | 9 | 0 | 1 | 700 | 1 | 700 | 0 | 1275 |
| Assembled_4234300-4235000 | Reference_4234300-4235000 | 100 | 700 | 0 | 0 | 1 | 700 | 1 | 700 | 0 | 1340 |
| Assembled_4242000-4242700 | Reference_4242000-4242700 | 99.86 | 700 | 1 | 0 | 1 | 700 | 1 | 700 | 0 | 1338 |
| Assembled_4249700-4250400 | Reference_4249700-4250400 | 99.29 | 700 | 5 | 0 | 1 | 700 | 1 | 700 | 0 | 1369 |
| Assembled_4257400-4258100 | Reference_4257400-4258100 | 97 | 700 | 21 | 0 | 1 | 700 | 1 | 700 | 0 | 1293 |
| Assembled_4265100-4265800 | Reference_4265100-4265800 | 99.57 | 700 | 3 | 0 | 1 | 700 | 1 | 700 | 0 | 1233 |
| Assembled_4272800-4273500 | Reference_4272800-4273500 | 99.71 | 700 | 2 | 0 | 1 | 700 | 1 | 700 | 0 | 1372 |
| Assembled_4280500-4281200 | Reference_4280500-4281200 | 99.14 | 700 | 6 | 0 | 1 | 700 | 1 | 700 | 0 | 1281 |
| Assembled_4288200-4288900 | Reference_4288200-4288900 | 99.86 | 700 | 1 | 0 | 1 | 700 | 1 | 700 | 0 | 1380 |
| Assembled_4295900-4296600 | Reference_4295900-4296600 | 99.71 | 700 | 2 | 0 | 1 | 700 | 1 | 700 | 0 | 1382 |
| Assembled_4303600-4304300 | Reference_4303600-4304300 | 99.86 | 700 | 1 | 0 | 1 | 700 | 1 | 700 | 0 | 1343 |
| Assembled_4311300-4312000 | Reference_4311300-4312000 | 96.76 | 617 | 20 | 0 | 1 | 617 | 1 | 617 | 0 | 1098 |
| Assembled_4319000-4319700 | Reference_4319000-4319700 | 77.14 | 700 | 160 | 0 | 1 | 700 | 1 | 700 | 0 | 807 |
| Assembled_4326700-4327400 | Reference_4326700-4327400 | 89.73 | 224 | 23 | 0 | 2 | 225 | 2 | 225 | 7e-73 | 267 |
| Assembled_4342100-4342800 | Reference_4342100-4342800 | 99.57 | 700 | 3 | 0 | 1 | 700 | 1 | 700 | 0 | 1380 |
| Assembled_4349800-4350500 | Reference_4349800-4350500 | 99.29 | 700 | 5 | 0 | 1 | 700 | 1 | 700 | 0 | 1369 |
| Assembled_4357500-4358200 | Reference_4357500-4358200 | 99.86 | 700 | 1 | 0 | 1 | 700 | 1 | 700 | 0 | 1380 |
| Assembled_4365200-4365900 | Reference_4365200-4365900 | 96.14 | 700 | 27 | 0 | 1 | 700 | 1 | 700 | 0 | 1214 |
| Assembled_4372900-4373600 | Reference_4372900-4373600 | 99.14 | 700 | 6 | 0 | 1 | 700 | 1 | 700 | 0 | 1345 |
| Assembled_4380600-4381300 | Reference_4380600-4381300 | 99.71 | 700 | 2 | 0 | 1 | 700 | 1 | 700 | 0 | 1294 |

The result is shown in the m8 format of the BLAST output. Query name represents the position of the gaps inserted in the simulated draft genome and subject name refers to the same positions in the reference genome. Aligned length represents the number of bases (out of 700) which were replaced by known bases in the reconstructed genome. Percentage identities represents the identity with which the gaps are replaced with the known bases.
