# Supplementary material for: Reconstruction of Bacterial and Viral Genomes from Multiple Metagenomes
Source: Front Microbiol. 2016 Apr 12;7:469. doi: 10.3389/fmicb.2016.00469 (PMC4828583; doi:10.3389/fmicb.2016.00469)
Supplement: Supplementary file 14 [file Table14.DOCX]

**Table S14. Percentage of assembly achieved after the addition of 'left over reads'.** 'Initial % assembly' represents the percentage of assembly achieved before using Flash rejected reads. 'left over reads' contains the 'Initially rejected reads' and 'Flash rejected reads'.

| **Genus** | **Initial % assembly** | **# Reads Used** | **% Assembly after addition of left over reads** | **# Reads Used** |
| --- | --- | --- | --- | --- |
| *Akkermansia* | 98.71 | 1258928 | 98.71 | 1258928 |
| *Bacteroides* | 98.89 | 10692762 | 98.89 | 10692762 |
| *Bifdobacterium* | 97.14 | 459827 | 97.14 | 459827 |
| *Escherichia* | 95.72 | 1045845 | 95.72 | 1045845 |
| *Eubacterium* | 95.07 | 4316322 | 96.36 | 7369124 |
| *Odoribacter* | 99.17 | 2785796 | 98.57 | 2785796 |
| *Parabacteroides* | 98.69 | 6132555 | 98.10 | 6132555 |
| *Roseburia* | 97.79 | 2913425 | 97.56 | 2912635 |
