# Supplementary material for: Reconstruction of Bacterial and Viral Genomes from Multiple Metagenomes
Source: Front Microbiol. 2016 Apr 12;7:469. doi: 10.3389/fmicb.2016.00469 (PMC4828583; doi:10.3389/fmicb.2016.00469)
Supplement: Supplementary file 15 [file DataSheet1.DOCX]

**Text S1**

*Improving draft assemblies using leftover reads*

The paired-end metagenomic reads data used in this study was processed using two steps, i) Q30 quality filtering, and ii) FLASH assembly. Due to Q30 filtering, 0.03% of the total reads, which belonged to either forward or reverse read of a paired-end read, could not qualify and was kept in a separate file labelled as 'Initially rejected reads'. The filtered paired-end reads were concatenated using FLASH into a single read and were used further for the above presented analysis. However, in the Flash assembly process a major fraction (56.6%) of reads was discarded and was labelled as 'Flash rejected reads'. It is to be noted that these rejected reads are completely non-erroneous, since in the former case, the mate pair for the read was missing and in the latter case, the minimum (4 bp) overlap was not observed among the mate pairs. The reads which failed the assembly using FLASH could not be used since the read length of a singleton is 75 bp which is too short for taxonomic classification by Kraken or any other available method. However, these reads can also be used for improving the assembly and coverage in genome reconstruction.

To examine if an improvement could be achieved in the coverage of the draft genomes of the seven top most abundant species which were earlier reconstructed in this study, the 'Initially rejected reads' and 'Flash rejected reads' were added to their genus-pool of reads. It was observed that after the addition of 'Initially rejected reads' and 'Flash rejected reads' the coverage did not show any improvement (Table S14). Also, the number of reads required to attain the highest coverage remained similar in almost all the cases (Table S14), further indicating that the addition of the 'Initially rejected reads' and 'Flash rejected reads' did not take part in improving the assembly process. This indicates that the number of reads present for each of the genome was sufficient to reach the saturation in assembly and further addition of reads did not improve the alignment coverage.
