# Supplementary figures and images for: Reconstruction of Bacterial and Viral Genomes from Multiple Metagenomes
Source: Front Microbiol. 2016 Apr 12;7:469. doi: 10.3389/fmicb.2016.00469 (PMC4828583; doi:10.3389/fmicb.2016.00469)

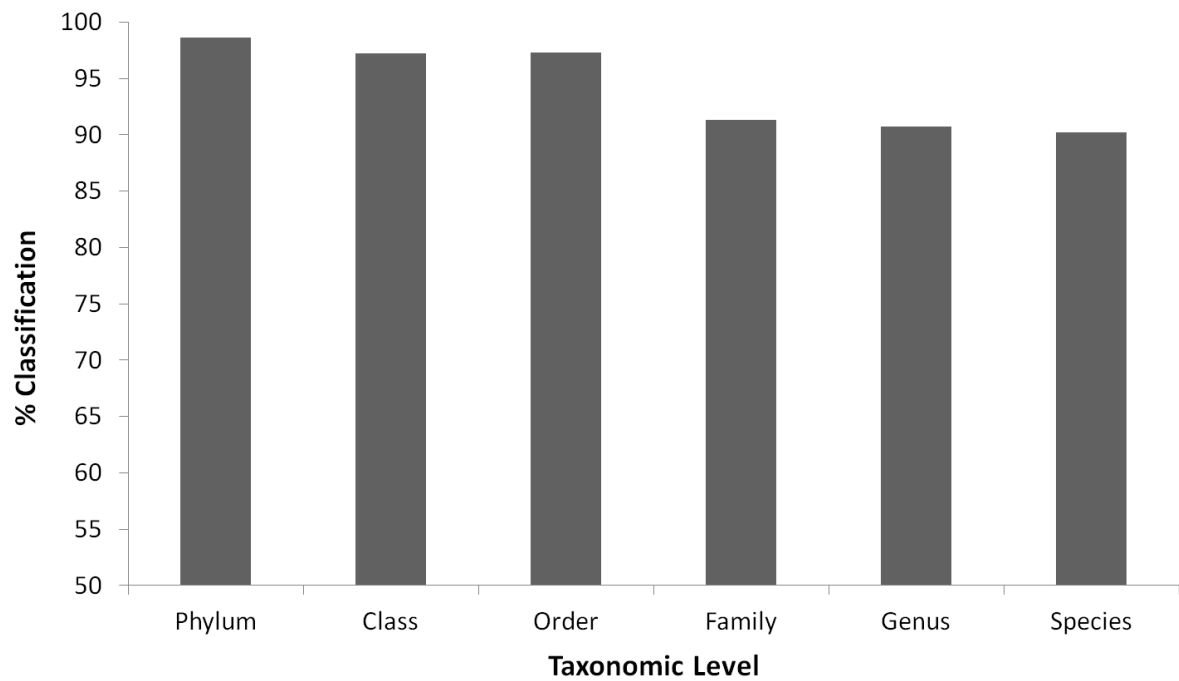

**Figure S1. Percentage of reads classified at different taxonomic levels by Kraken.**

Supplement: Supplementary file 16 [file Image1.PDF]

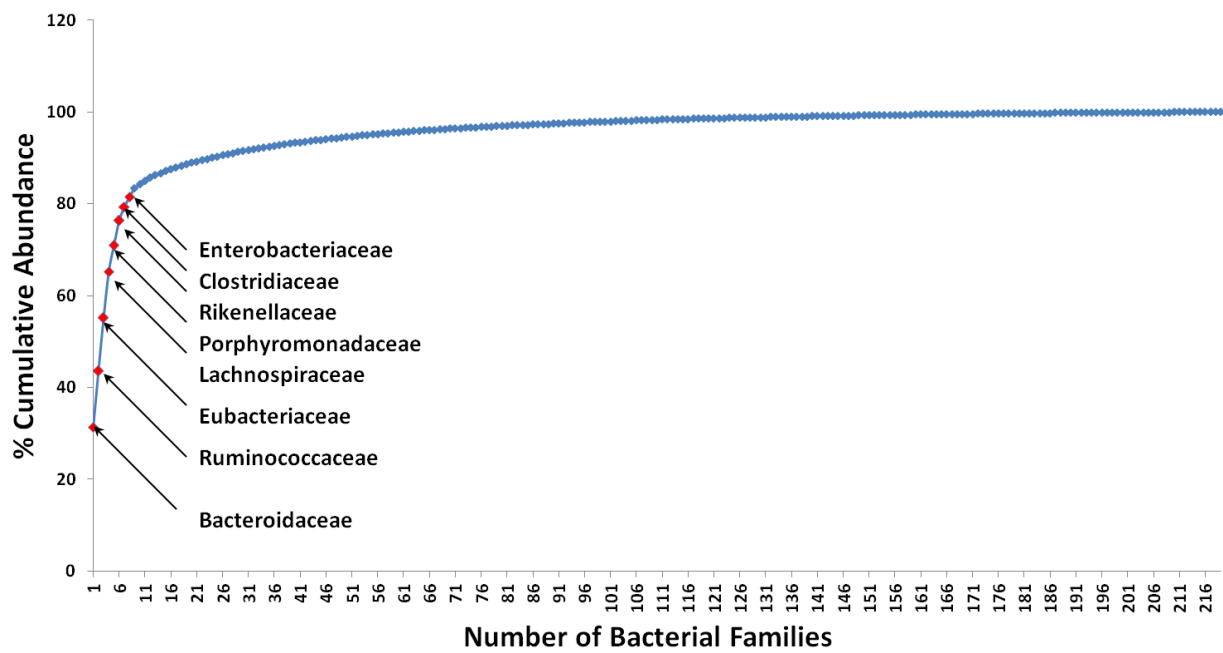

**Figure S2. Top eight families contributing more than 80% of the total reads.**

Supplement: Supplementary file 17 [file Image2.PDF]
