# Supplementary material for: Reconstruction of Bacterial and Viral Genomes from Multiple Metagenomes
Source: Front Microbiol. 2016 Apr 12;7:469. doi: 10.3389/fmicb.2016.00469 (PMC4828583; doi:10.3389/fmicb.2016.00469)
Supplement: Supplementary file 18 [file Image3.PDF]

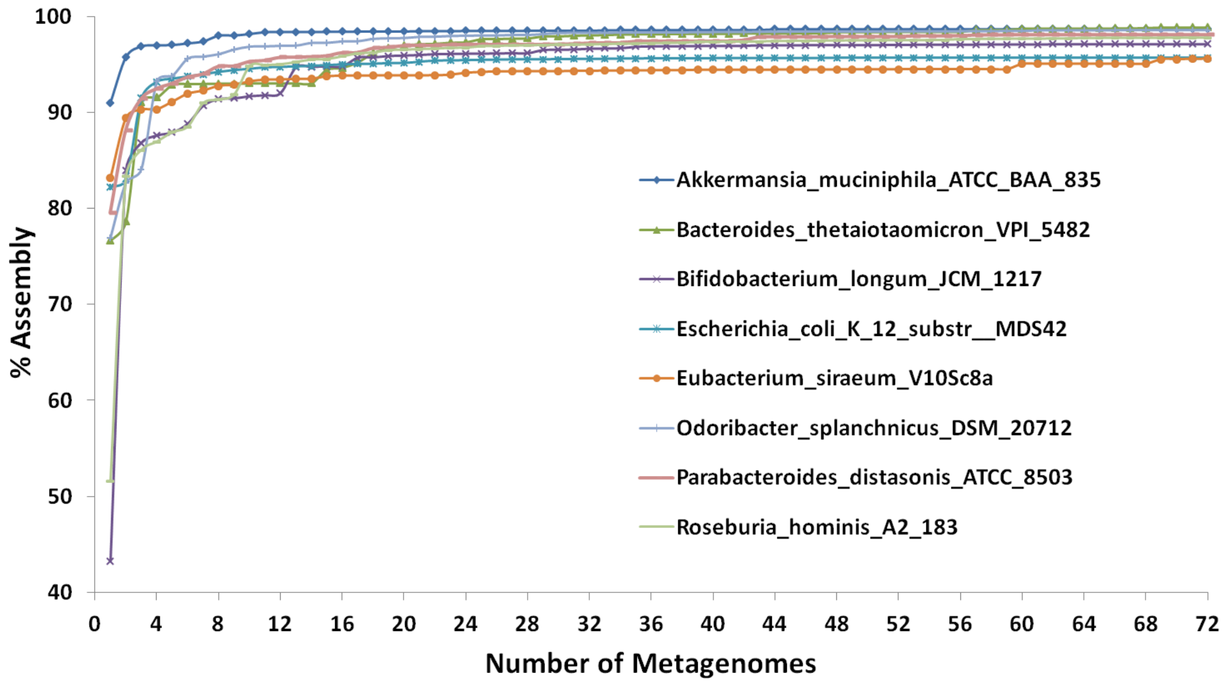

Figure S3. Incremental addition of reads from 72 metagenomes based on the respective genus abundance for eight selected bacterial genomes.
